# Radical trifluoromethoxylation of fluorinated alkenes for accessing difluoro(trifluoromethoxy)methyl groups†

**DOI:** 10.1039/d4sc07788a

**Published:** 2025-01-13

**Authors:** Koki Kawai, Mai Usui, Sota Ikawa, Naoyuki Hoshiya, Yosuke Kishikawa, Norio Shibata

**Affiliations:** a Department of Nanopharmaceutical Sciences, Nagoya Institute of Technology Gokiso, Showa-ku Nagoya 466-8555 Japan nozshiba@nitech.ac.jp; b Department of Engineering, Nagoya Institute of Technology Gokiso, Showa-ku Nagoya 466-8555 Japan; c Technology and Innovation Center, DAIKIN Industries, Ltd 1-1 Nishi-Hitotsuya, Settsu Osaka 566-8585 Japan

## Abstract

In this study, we explore the potential of the difluoro(trifluoromethoxy)methyl group, CF_2_–O–CF_3_, an underexplored but promising structural analog of the trifluoromethoxy group (OCF_3_). This moiety offers unique electronic properties and enhanced chemical stability due to its multiple C–F bonds, along with the added advantage of C–O bond cleavage, making it an attractive option in fluorine chemistry. We have succeeded in synthesizing difluoro(trifluoromethoxy)methyl compounds *via* radical amino- and hydroxy-trifluoromethoxylations of β,β-difluorostyrenes. Control experiments, including radical clock experiments, support a free radical mechanism. The synthetic utility of the resulting difluoro(trifluoromethoxy)methyl compounds is also demonstrated through transformations into bioactive analogs, such as pyrrole derivatives, fendiline analogs, and carpropamid analogs, highlighting their potential in drug development.

## Abstract 2

In this study, we explore the potential of the difluoro(trifluoromethoxy)methyl group, CF_2_–O–CF_3_, an underexplored but promising structural analog of the trifluoromethoxy group (OCF_3_).

## Graphical abstract

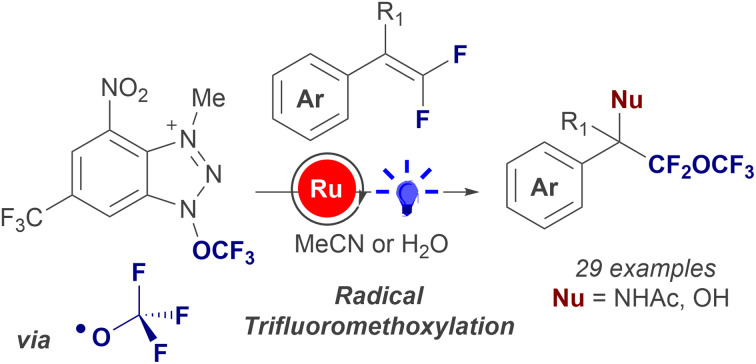

## Introduction

**Fig. 1:**
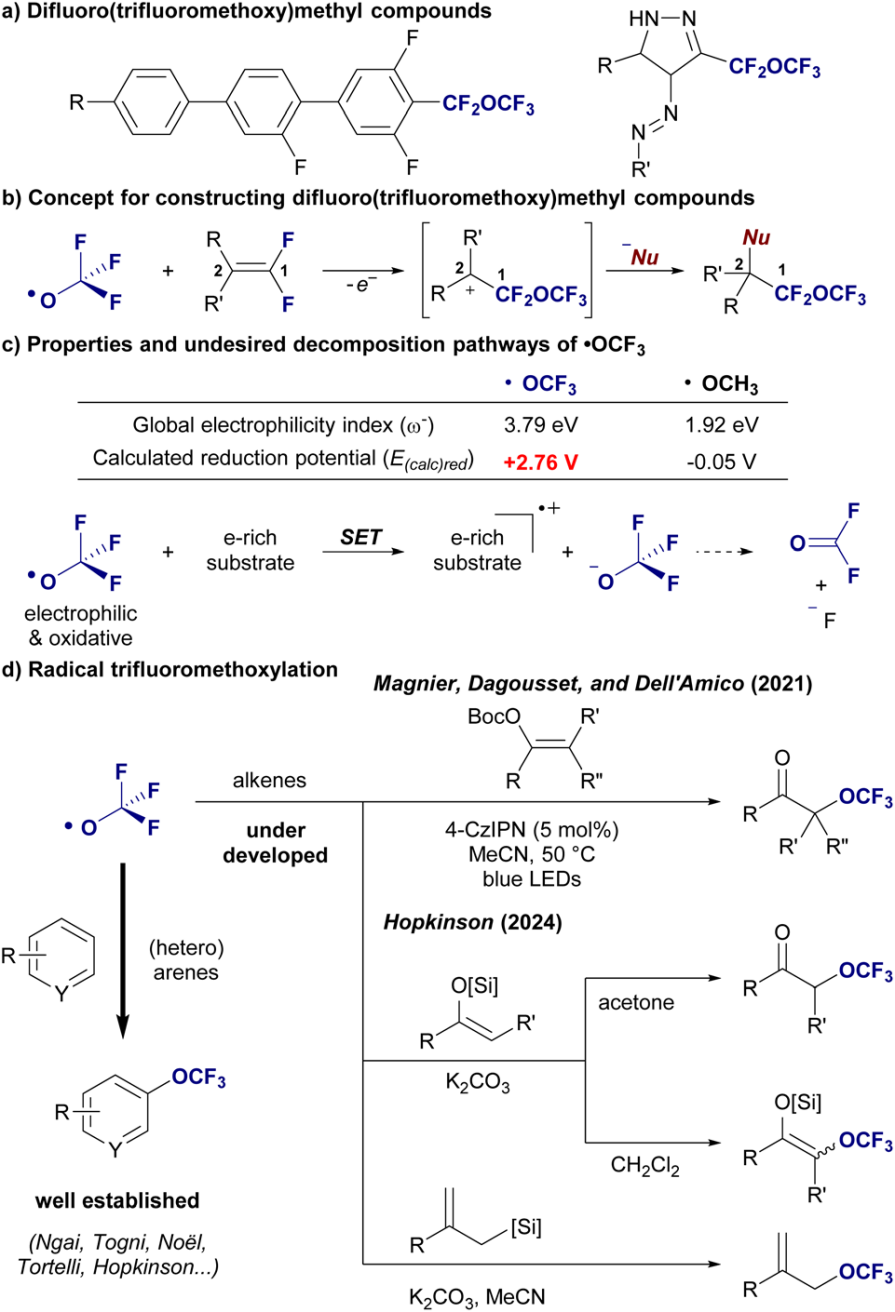
(a) Examples of compounds with the difluoro(trifluoromethoxy)methyl (–CF_2_–O–CF_3_) group. (b) Concept for constructing difluoro(trifluoromethoxy)methyl compounds (this work). (c) Properties and decomposition pathways of the OCF_3_ radical. (d) Radical trifluoromethoxylation (previous studies).

The development of new functional motifs has long been a cornerstone of the evolution of molecular design, particularly in the fields of drug discovery^1^ and materials science.^2^ Fluorinated functional groups have attracted significant attention owing to their unique chemical properties, including their ability to modulate lipophilicity, metabolic stability, and bioavailability.^3^ Among these, the trifluoromethoxy (OCF_3_) group is well established and is known to enhance the metabolic stability and improve the physicochemical properties of bioactive molecules.^4^ However, its structural analog, the difluoro(trifluoromethoxy)methyl group, –CF_2_–O–CF_3_, has been rarely examined, representing a largely untapped frontier in fluorine chemistry (Fig. 1a).^5^

The introduction of the –CF_2_–O–CF_3_ moiety provides a novel opportunity to explore its potential as a terminal functional group in molecular design. This group is distinct from the well-studied –OCF_3_ group due to the addition of the difluoromethylene (–CF_2_–) unit, which may offer unique lipophilic, electronic and steric effects that can significantly impact molecular interactions and reactivity.^6^ The presence of multiple C–F bonds in this structure is expected to impart high electronegativity and chemical stability, which are both highly desirable traits in drug candidates and advanced materials. In addition to its desirable electronic and steric properties, the –CF_2_–O–CF_3_ moiety presents another unique property. According to ref. 5, owing to the oxygen atom embedded within the perfluoroalkyl unit, the –CF_2_–O–CF_3_ structure is susceptible to C–O bond cleavage under UV irradiation conditions.^7^ Thus, the CF_2_–O–CF_3_ group is a unique functional group that combines strong fluorine-based attributes with a potential property of bond cleavage under specific conditions, making this functional group an attractive option in the design of organofluorine compounds.

The synthetic methods for difluoro(trifluoromethoxy)methyl molecules are not well established. DesMarteau^8^ reported the addition reactions of trifluoromethoxy hypohalite, CF_3_OX (X = F, Cl), with fluoroalkenes to give difluoro(trifluoromethoxy)methyl compounds. However, CF_3_OX poses significant challenges due to its strong oxidizing power, high reactivity, and gaseous nature, making it difficult to handle. Therefore, the development of practical methods to access difluoro(trifluoromethoxy)methyl molecules is highly desirable. In this study, we present an investigation into the synthesis of difluoro(trifluoromethoxy)methyl-molecules through radical trifluoromethoxylation of β,β-fluorinated alkenes. This approach not only introduces a new fluorinated functional group^9^ but also opens new pathways for the design of molecules with enhanced fluorine-induced properties and controlled degradation potential. Our strategy for constructing difluoro(trifluoromethoxy)methyl compounds is based on the site selective radical trifluoromethoxylation at the C1-position of difluorinated alkenes, followed by nucleophilic functionalization at the C2-position (Fig. 1b). However, this process presents significant challenges due to the highly reactive and electrophilic nature of the trifluoromethoxy radical, ˙OCF_3_. The trifluoromethoxy radical exhibits exceptional electrophilicity, as indicated by its high global electrophilicity index (*ω*^−^ = 3.79 eV), and is characterized by strong oxidative power, with a calculated reduction potential of *E*_(calc)red_ = +2.76 V. These features contrast sharply with those of the much milder methoxy radical, ˙OCH_3_, (*E*_(calc)red_ = −0.05 V), underscoring the unruly reactivity profile of the trifluoromethoxy radical (Fig. 1c).^10^ This pronounced electrophilicity makes the trifluoromethoxy radical highly selective for electron-rich substrates, where it readily engages in radical addition. However, this same reactivity also increases the likelihood of undesired oxidative pathways, including single-electron transfer (SET) processes, which significantly restrict the range of compatible substrates. Indeed, the combination of strong oxidative potential and the propensity for side reactions limits the utility of this radical in many contexts. Over the past few years, several radical trifluoromethoxylation reagents have been developed, with the majority focused on trifluoromethoxylation of aromatic systems.^11^ However, these methods have predominantly been limited to electron-deficient arenes, where oxidative side reactions can be controlled. Magnier, Dagousset, and Dell'Amico (2021) demonstrated the radical trifluoromethoxylation of enol carbonates using the Togni OCF_3_ reagent, offering a rare example of radical trifluoromethoxylation beyond the aromatic ring.^12^ More recently, in 2024, Hopkinson expanded the substrate scope by applying the bis(trifluoromethyl)peroxide (BTMP) reagent to the radical trifluoromethoxylation of silyl enol ethers (Fig. 1d).^13^ These advancements mark significant progress, yet the scope remains largely confined to specific functionalized alkenes and electron-poor arenes, limiting broader applicability (Fig. 1c). Thus, the inherent difficulty lies in controlling the strong electrophilic and oxidizing nature of trifluoromethoxy radicals, which often results in undesirable side reactions, such as SET oxidative pathways, and thus narrows the substrate scope.

## Results and discussion

We designed the photocatalytic amino-trifluoromethoxylation of fluorinated alkenes, using acetonitrile as the nucleophile, exploiting the Ritter reaction mechanism.^14^ Initially, three trifluoromethoxy reagents, namely 1a and 1b (by Ngai)^11*c*,*d*^ and 1c (by Togni)^11*b*^ were evaluated with the treatment of 4-chloro-(β,β-difluoro)-styrene (2a, 5.0 equiv.) with Ru(bpy)_3_(PF_6_)_2_ (1 mol%), and H_2_O (1.0 equiv.) in acetonitrile under blue LED irradiation at rt for 1 h (entries 1–3, Table 1). As expected, the Ritter-type amino trifluoromethoxylation proceeded well, especially with reagent 1a, yielding the desired product 3a in 52% yield, along with a hydroxylated by-product 4a (9%, entry 1). To suppress the formation of 4a, we explored the use of acid additives. The addition of triflic acid showed no improvement (3a, 51% and 4a, 9%, entry 4). However, using bis(trifluoromethanesulfonyl)imide (Tf_2_NH) resulted in a more selective transformation, yielding 3a in 64% (entry 5), which we adopted as our standard condition. We next performed control experiments to further optimize the reaction. Reducing the amount of difluorostyrene 2a led to a decreased yield of 3a (54%, entry 6). The use of excess substrate 2a was necessary due to the high oxidative power of the trifluoromethoxy radical, a challenge that is consistent with previous reports on radical trifluoromethoxylation reactions. The yield decreased to 35% when the ratio of reactants 1a/2a = 2/1 (entry 7). Substituting Ru(bpy)_3_(PF_6_)_2_ with other photocatalysts, such as [Ir(dFCF_3_ppy)_2_-(5,5′-dCF_3_bpy)]PF_6_ or 4CzIPN, resulted in significantly lower yields (9% and 22%, respectively; entries 8 and 9), underscoring the importance of the ruthenium photocatalyst. Increasing the water content in the reaction mixture had a detrimental effect, reducing both the yield and selectivity (3a, 23% and 4a, 15%, entry 10). In contrast, switching the solvent to acetone led to the selective formation of the hydroxy-trifluoromethoxylated product 4a (19%, entry 11), with yields further improving in the absence of TF_2_NH (4a, 45%, entry 12). Control experiments confirmed the necessity of both photo-irradiation and the photocatalyst, as no reaction occurred without either component (entries 13 and 14).

**Table 1:** Amino-trifluoromethoxylation of β,β-difluorostyrene (2a)^a^

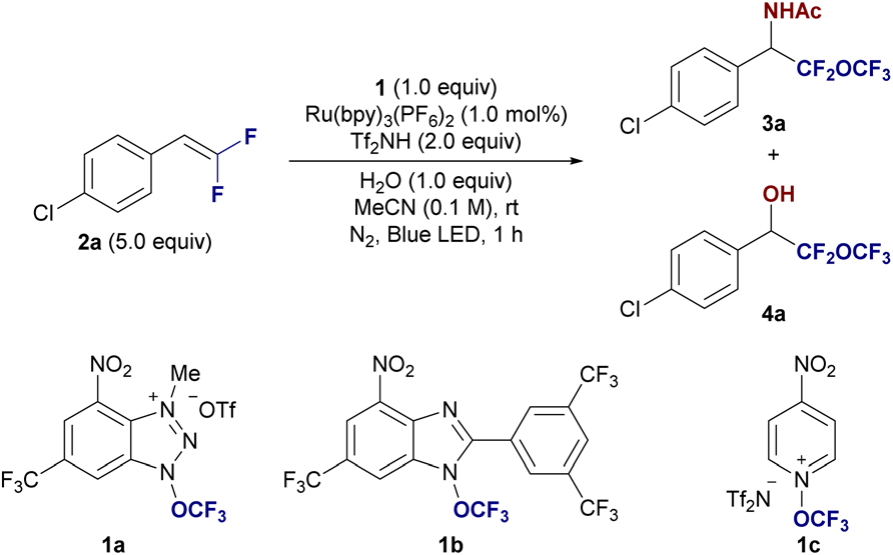			
Entry	1	Deviations from standard conditions	Yield 3a (%)^b^/4a (%)^b^
1	1a	Without Tf_2_NH	52/9
2	1b	Without Tf_2_NH	0/0
3	1c	Without Tf_2_NH	28/0
4	1a	TfOH instead of Tf_2_NH	51/9
5	1a	None	64/0
6	1a	2a (1.0 equiv.)	54/0
7	1a	1a (2.0 equiv.), 2a (1.0 equiv.)	35/0^c^
8	1a	[Ir(dFCF_3_ppy)_2_-(5,5′-dCF_3_bpy)]PF_6_ (1.0 mol%)	9/0
9	1a	4CzIPN (1.0 mol%)	22/0
10	1a	H_2_O (50 equiv.)	23/15
11	1a	Acetone instead of MeCN	0/19
12	1a	Acetone (0.1 M), without Tf_2_NH	0/45
13	1a	No light	0/0
14	1a	No photocatalyst	0/0

a Unless otherwise noted, the standard conditions refer to 1 (0.1 mmol), 2a (0.5 mmol), Tf_2_NH (0.2 mmol), H_2_O (0.1 mmol), and Ru(bpy)_3_(PF_6_)_2_ (1.0 mol%) in MeCN irradiated at rt for 1 h.

b Determined by ^19^F NMR spectroscopy using C_6_F_6_ as the internal standard.

c Yields based on 2a.

**Fig. 2:**
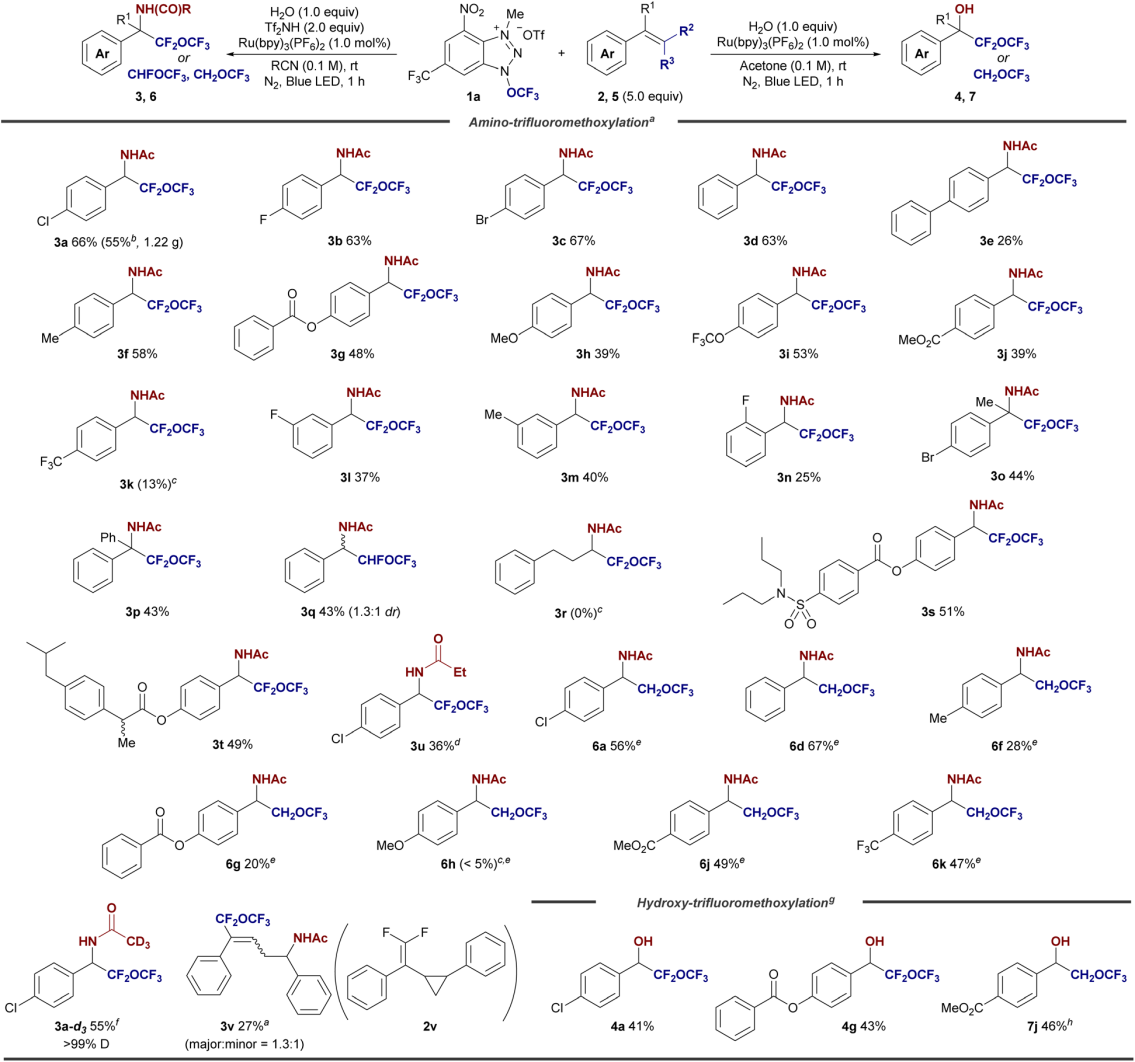
Substrate scope of the amino and hydroxy-perfluoroalkoxylation of styrenes 2. ^*a*^ Reaction conditions for the amino-trifluoromethoxylation: 1a (0.3 mmol), 2 (1.5 mmol), H_2_O (0.3 mmol), Tf_2_NH (0.6 mmol), and Ru(bpy)_3_(PF_6_)_2_ (1.0 mol%) in MeCN (3.0 mL), under N_2_ and blue LED (450 nm) irradiation for 1 h at room temperature. Isolated yields were shown. ^*b*^ Gram scale reaction. 1a (7.0 mmol, 3.36 g) was used. ^*c*^ Crude reaction mixture was measured by ^19^F NMR. ^*d*^ EtCN as a solvent. ^*e*^ Without Tf_2_NH. ^*f*^ CD_3_CN as a solvent. ^*g*^ Reaction conditions for the hydroxy-perfluoroalkoxylation: 1a (0.3 mmol), 2 (1.5 mmol), H_2_O (0.3 mmol), and Ru(bpy)_3_(PF_6_)_2_ (1.0 mol%) in acetone (3.0 mL), under N_2_ and blue LED (450 nm) irradiation for 1 h at room temperature. Isolated yields were shown. ^*h*^ Na_3_PO_4_ (1.0 equiv.) was added.

With the optimized reaction conditions in hand, we explored the generality of the amino-trifluoromethoxylation of difluoroalkenes 2 with 1a in acetonitrile. As illustrated in Fig. 2, a broad range of β,β-difluorostyrenes 2 containing both electron-donating and electron-withdrawing groups on the aryl ring were successfully employed, yielding the desired α-difluoro(trifluoromethoxy)methyl benzylamine derivatives 3 in good yields. Halogen-substituted β,β-difluorostyrenes at the *para*-position (2a: Cl, 2b: F, 2c: Br) provided the corresponding difluoro(trifluoromethoxy)methyl benzylamines 3a–c in 63–67% yields. Styrene (2d) was also converted to simple α-difluoro(trifluoromethoxy)methyl benzylamine 3d in 63% yield. Additionally, difluorinated biphenyl styrene (2e) was converted to 3e in 26% yield. Electron-donating groups (2f: Me, 2g: OCOPh, and 2h: OMe) and electron-withdrawing groups (2i: OCF_3_ and 2j: CO_2_Me) on the *para*-position at the aryl ring of β,β-difluorostyrenes afforded the corresponding products 3f–j in 39–58% yields, while the *p*-CF_3_-substituted styrene 2k lowered the yield of 3k to 13%. The alkene moiety in 2k would be more electrophilic. This reduces the reactivity towards the electrophilic trifluoromethoxy radical. The *meta*-substituted styrene derivatives (F, 2l; Me, 2m) yielded 3l and 3m in 40% and 37% yields, respectively. However, the *ortho*-fluorinated styrene 2n provided a lower yield of 3n (25%), likely due to steric hindrance at the *ortho* position. Interestingly, the reaction also accommodated α-methyl-substituted β,β-difluorostyrene (2o) and α,α-diphenyl β,β-difluorostyrene (2p), giving the corresponding amines 3o and 3p in 44% and 43% yields, respectively. When β-monofluorostyrene (2q) was subjected to the standard conditions, it delivered the α-fluoro(trifluoromethoxy)methyl, –CFH–O–CF_3_, containing benzylamine derivative 3q in 43% yield with a diastereomeric ratio of 1.3 : 1. However, the aliphatic difluoroalkene 2r failed to produce the desired product 3r, highlighting a limitation of the method. We further explored the reaction with β,β-difluorostyrenes derived from drug molecules. Probenecid- (2s) and ibuprofen-derived (2t) difluorostyrenes underwent amino-trifluoromethoxylation smoothly, yielding α-difluoro(trifluoromethoxy)methylated drug candidates 3s and 3t in 51% and 49% yields, respectively. Notably, difluoro(trifluoromethoxy)methylated ethyl amide 3u was produced in propionitrile as the solvent with a yield of 36%. A gram-scale reaction using 7.0 mmol (3.36 g) of 1a under optimized conditions resulted in the isolation of α-difluoro(trifluoromethoxy)methyl benzylamine derivative 3a in 55% yield (1.22 g isolated).

To further expand the scope of this reaction, we also investigated non-fluorinated styrenes 5. Without the use of Tf_2_NH, various styrenes substituted with functional groups, including halogens, electron-donating, and electron-withdrawing groups (5a: Cl, 5d: H, 5f: Me, 5g: OCOPh, 5j: CO_2_Me, and 5k: CF_3_), successfully yielded the corresponding (trifluoromethoxy)methyl products 6 in 20–67% yields. However, the electron-rich styrene with a *para*-methoxy (OMe) group (5h) failed to produce the desired product 6h, likely due to single-electron transfer between the trifluoromethoxy radical and the electron-rich aromatic system.

The method also proves effective for the synthesis of deuterium-labeled products, which are of particular interest in drug design due to the kinetic isotope effect. Deuterium-containing drugs often exhibit reduced metabolic rates, leading to a longer half-life.^15^ By using deuterated acetonitrile (CD_3_CN) instead of regular acetonitrile under optimized conditions, we successfully isolated the deuterated product 3a-*d*_3_ in 55% yield. From a mechanistic perspective, we also investigated the reaction using cyclopropyl-containing difluorostyrene 2v under standard conditions. Interestingly, the 1,4-amino-trifluoromethoxylation reaction proceeded *via* ring-opening of the cyclopropyl group, yielding the vinyl difluoro(trifluoromethoxy)methyl product 3v as an *E*/*Z* mixture (major = 1.3 : 1) in 27% yield. This outcome supports the involvement of a free radical process in the amino-trifluoromethoxylation reaction.

We next turned our attention to the hydroxy-trifluoromethoxylation of fluorinated styrenes 2 for providing (trifluoromethoxy)methyl benzyl alcohol derivatives 4. The reaction proceeded stably when β,β-difluorostyrenes (2a and 2g) were treated with 1a, Ru(bpy)_3_(PF_6_)_2_ (1 mol%), and H_2_O (1 equiv.) in acetone, yielding 4a and 4g in 41% and 43% yields, respectively. Similarly, non-fluorinated styrene 5j afforded the (trifluoromethoxy)methyl benzyl alcohol 7j in 46% yield when Na_3_PO_4_ (1 equiv.) was used.

**Fig. 3:**
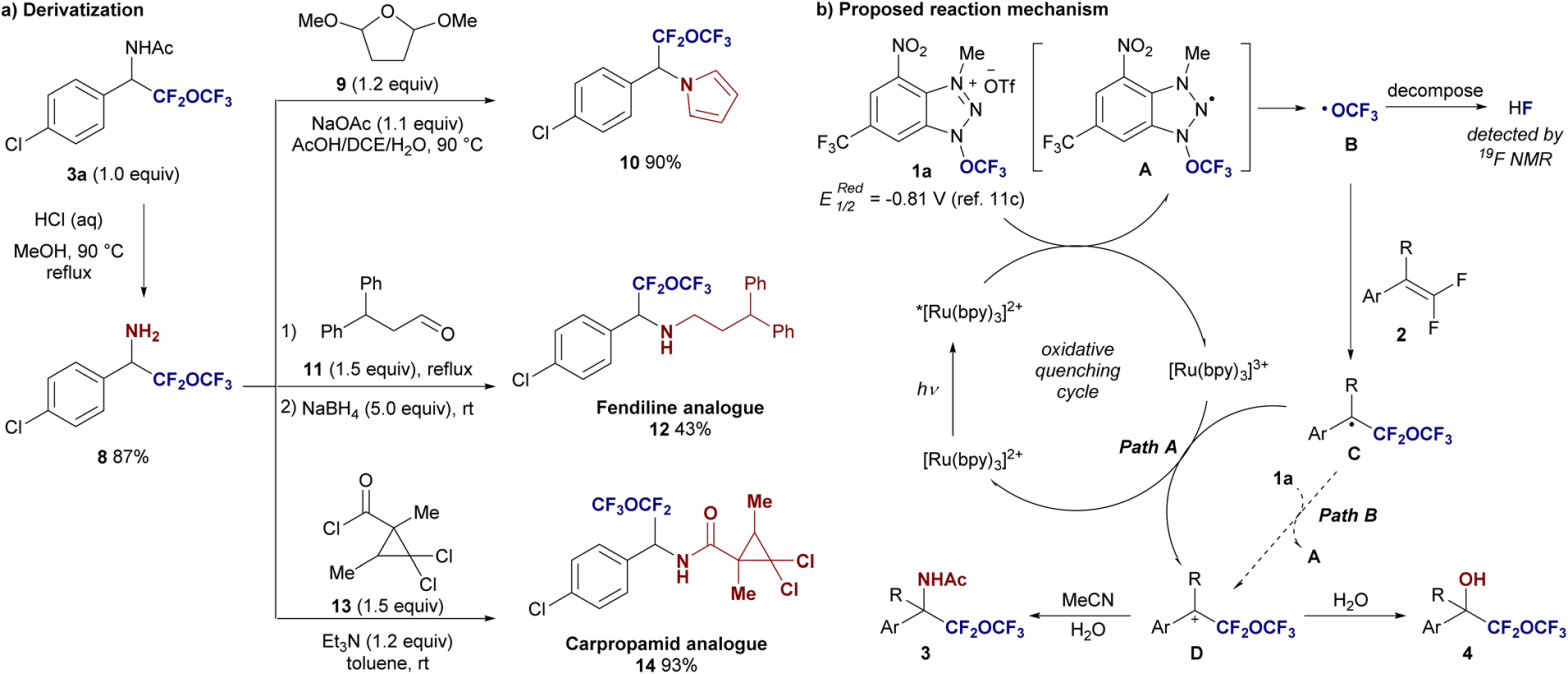
(a) Synthetic application and (b) proposed reaction mechanism.

To demonstrate the synthetic utility of the obtained difluoro(trifluoromethoxy)methyl benzyl amine derivatives 3, we first removed the acetyl group from compound 3a under acidic conditions, yielding the free amine 8 in high yield (Fig. 3). This transformation allows the amine functionality to be accessible for various subsequent modifications. Moreover, this fact indicated that the –CF_2_–O–CF_3_ unit is stable under acidic conditions with heated. In a Clauson–Kass type reaction, difluoro(trifluoromethoxy)methyl benzyl amine 8 was reacted with 2,5-dimethoxytetrahydrofuran 9 at 90 °C, affording the corresponding difluoro(trifluoromethoxy)methyl benzylpyrrole derivative 10 in an excellent 90% yield. In another transformation, amine 8 was reacted with aldehyde 11 in the presence of triethylamine (Et_3_N), followed by NaBH_4_ reduction, yielding the *N*-alkylated fendiline analogue 12 in 43% yield over two steps (fendiline is a nonselective calcium channel blocker). Additionally, treating amine 8 with a suitably designed acyl chloride 13 provided the carpropamid analogue 14 in 93% yield (carpropamid is a melanin-inhibiting fungicide). These results highlight the versatility of difluoro(trifluoromethoxy)methyl benzyl amines and their potential for generating bioactive molecules, demonstrating the synthetic value of this functional group in medicinal chemistry.

Based on these experimental results, along with precedents from reported radical alkoxylation mechanisms, we propose the mechanism shown in Fig. 3b. The reaction begins with the photoexcitation of the Ru(ii) catalyst, which reduces the trifluoromethoxylation reagent 1a to form the OCF_3_ radical B*via* intermediate A. The OCF_3_ radical B then undergoes addition to the alkene 2, generating the stable benzyl radical intermediate C. The formation of the stable benzyl radical intermediate C is critical to this transformation, as the aliphatic 2r has not been converted to the adduct 3r. This radical intermediate C is subsequently oxidized by the Ru(iii) catalyst (path A), producing the benzyl cation intermediate D. The cationic intermediate D can be trapped by acetonitrile in a Ritter-type reaction, leading to the formation of an amide product 3. Alternatively, when the cation is trapped by water, a hydroxylated product 4 is formed. The moderate yields observed in all cases can be attributed to the competitive decomposition of the OCF_3_ radical into HF, as evidenced by the presence of an HF peak in the crude reaction mixture in the 1^9^F NMR spectrum and the substantial amount of unreacted starting material 2. To investigate whether the reaction proceeds *via* a radical chain mechanism (path B), we conducted a light/dark experiment. The experiment demonstrated that the reaction does not occur under dark conditions (see the ESI†). While this result suggests a dependence on light, it does not definitively rule out the possibility that the trifluoromethoxylation reaction involves a radical chain mechanism.^16^

## Conclusions

In this work, we have presented the design and synthesis of difluoro(trifluoromethoxy)methyl compounds, a novel functional group with potential applications in both pharmaceuticals and materials. Using a radical trifluoromethoxylation approach, we selectively functionalized β,β-difluorostyrenes to introduce the –CF_2_–O–CF_3_ moiety, which imparts unique electronic and steric properties compared to its well-known trifluoromethoxy counterpart (–OCF_3_). Our optimization studies highlighted the challenges associated with controlling the highly electrophilic trifluoromethoxy radical, particularly in preventing side reactions such as single-electron transfer. We achieved site-selective amino-trifluoromethoxylation using Ru(bpy)_3_(PF_6_)_2_ as a photocatalyst and explored various β,β-difluorostyrenes, including those derived from drug molecules, expanding the substrate scope significantly. Hydroxy-trifluoromethoxylation was also achieved. This work highlights the –CF_2_–O–CF_3_ group as a valuable addition to fluorine chemistry, offering both unique reactivity and properties for advanced molecular design. Further studies on the application of difluoro(trifluoromethoxy)methyl compounds as drug candidates and functional materials are currently underway in our laboratory.

## Data availability

The data that support the findings of this study are available within the article and the ESI.† Details about materials and methods, experimental procedures, characterization data, and NMR spectra are included.

## Author contributions

KK optimized the reaction conditions. KK, MU and SI surveyed the substrate scope, analyzed the data, and then discussed the results with NH, YK and NS. KK and NS wrote the manuscript. NS supervised the project. All authors contributed to the manuscript and have approved the final version of the manuscript.

## Conflicts of interest

There are no conflicts to declare.

## Supplementary Material

SC-016-D4SC07788A-s001
