# Supplementary material for: Radical trifluoromethoxylation of fluorinated alkenes for accessing difluoro(trifluoromethoxy)methyl groups
Source: Chem Sci. 2025 Jan 13;16(6):2830–6. doi: 10.1039/d4sc07788a (PMC11726583; doi:10.1039/d4sc07788a)

Supplementary Information (SI) for Chemical Science.
This journal is © The Royal Society of Chemistry 2025

# Electronic Supplementary Information

# Radical trifluoromethoxylation of fluorinated alkenes for accessing difluoro(trifluoromethoxy)methyl groups

Koki Kawai[1], Mai Usui[2], Sota Ikawa[2], Naoyuki Hoshiya[3], Yosuke Kishikawa[3], and Norio Shibata[1,2]

[1] Department of Nanopharmaceutical Sciences, Nagoya Institute of Technology, Gokiso, Showa-ku, Nagoya 466-8555, Japan.

[2] Department of Engineering, Nagoya Institute of Technology Gokiso, Showa-ku, Nagoya 466-8555, Japan

[3] Technology and Innovation Center DAIKIN Industries, Ltd. 1-1 Nishi-Hitotsuya, Settsu, Osaka 566-8585, Japan

*E-mail: nozshiba@nitech.ac.jp

## CONTENTS

1. General information ........................................ 1
2. Additional optimization data ........................................ 2
3. Preparation of alkenes 2, 5 ........................................ 3
4. Amino–trifluoromethoxylation of 2 ........................................ 4
5. Hydroxy–trifluoromethoxylation of 2 ........................................ 10
6. Amino–trifluoromethoxylation of 5 ........................................ 11
7. Hydroxy–trifluoromethoxylation of 5 ........................................ 13
8. Procedure for synthesis of compound 8 ........................................ 13
9. Procedure for synthesis of compound 10 ........................................ 14
10. Procedure for synthesis of compound 12 ........................................ 14
11. Procedure for synthesis of compound 14 ........................................ 15
12. Light/Dark experiment ........................................ 16
13. Reference ........................................ 16
14. NMR data ........................................ 17

## 1. General information

All reactions were performed in oven-dried glassware under positive pressure of nitrogen unless otherwise mentioned. Solvents were transferred via syringe and were introduced into the reaction vessels though a rubberseptum. All of the reactions were monitored by thin-layer chromatography (TLC) carried out on 0.25 mm Merck silica gel (60-$F_{254}$). The TLC plates were visualized with UV light and $KMnO_4$ in water/heat. Column chromatography was carried out on columns packed with silica gel (60N spherical neutral size 63-210 nm). The $^1$H NMR (500 MHz, 700 MHz), $^{19}$F NMR (282 MHz), $^{13}$C NMR (126 MHz) spectra for solution in $CDCl_3$ were recorded on a Varian 300 and a Buruker Avance 500 and JEOL ECZ700R. Chemical shifts (δ) are expressed in ppm downfield from TMS (δ = 0.00 ppm for $^1$H NMR), $C_6F_6$ (δ = −162.2 ppm for $^{19}$F NMR) or $CDCl_3$ (77.16 ppm for $^{13}$C NMR) as an internal standard. Mass spectra were recorded on a SHIMAZU LCMS-2020 (ESI-MS) and JEOL JMS-700 (FAB-MS). Melting points were recorded on Buchi M-565. Infrared spectra were recorded on a JASCO FT/IR-4100 spectrometer. Chemicals were purchased and used without further purification unless otherwise noted.

## 2. Additional optimization data

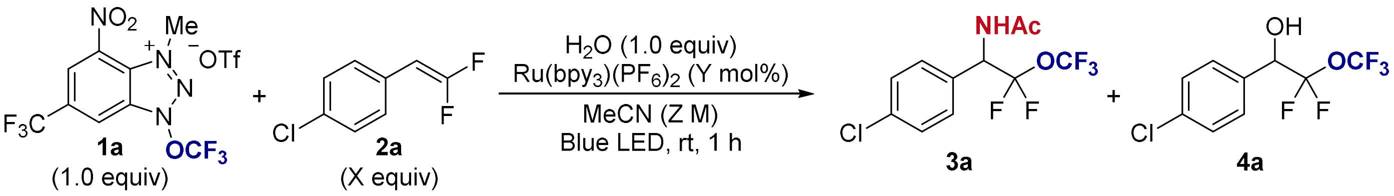

| Entry | **2** (X equiv) | Ru(bpy)$_3$(PF$_6$)$_2$ (Y mol%) | MeCN (Z M) | **3a** (%) | **4a** (%) | **3a+4a** (%) |
|---|---|---|---|---|---|---|
| 1 | 5.0 | 2.0 | 0.1 | 48 | 12 | 60 |
| 2 | 5.0 | 1.0 | 0.1 | 50 | 10 | 60 |
| 3 | 5.0 | 0.5 | 0.1 | 35 | 18 | 53 |
| 4 | 10 | 2.0 | 0.05 | 50 | 13 | 63 |

Yields were determined by $^{19}F$ NMR using $C_6F_6$ as an internal standard.

**Table S1**

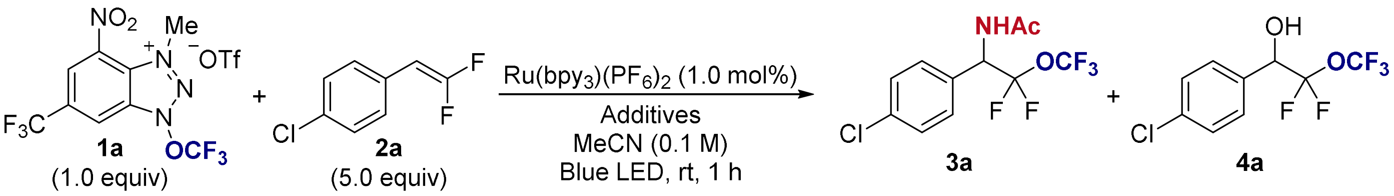

| Entry | Adidives | **3a** (%) | **4a** (%) | **3a+4a** (%) |
|---|---|---|---|---|
| 1 | $K_2HPO_4 \cdot 3H_2O$ (1.0 equiv) | 44 | 16 | 60 |
| 2 | $H_2O$ (1.0 equiv) | 50 | 10 | 60 |
| 3 | none | 30 | 8 | 38 |
| 4 | KF (2.0 equiv) | 40 | 22 | 62 |
| 5 | TfOH (2.0 equiv), $H_2O$ (1.0 equiv) | 51 | 9 | 60 |
| 6 | $Tf_2NH$ (2.0 equiv), $H_2O$ (1.0 equiv) | 63 | trace | 63 |

Yields were determined by $^{19}F$ NMR using $C_6F_6$ as an internal standard.

**Table S2**

## 3. Preparation of alkenes 2, 5

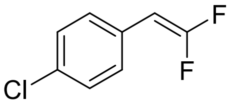
**2a**

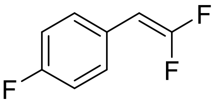
**2b**

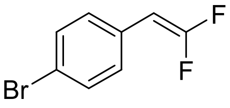
**2c**

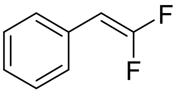
**2d**

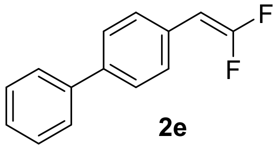
**2e**

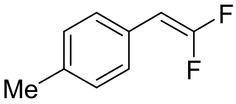
**2f**

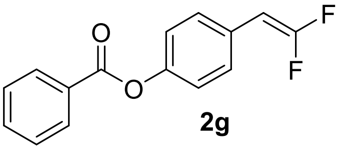
**2g**

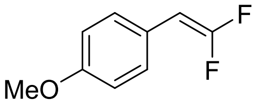
**2h**

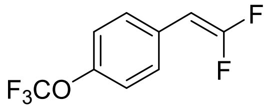
**2i**

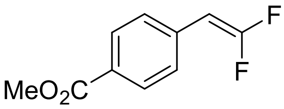
**2j**

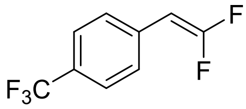
**2k**

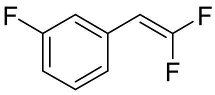
**2l**

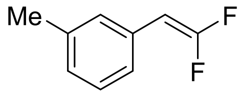
**2m**

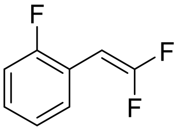
**2n**

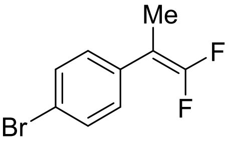
**2o**

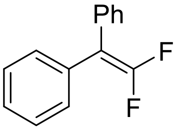
**2p**

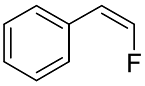
**2q**

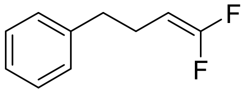
**2r**

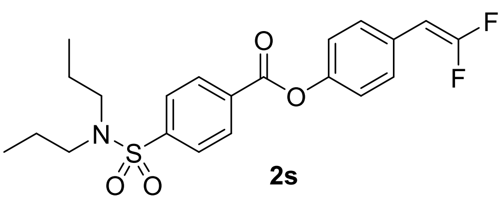
**2s**

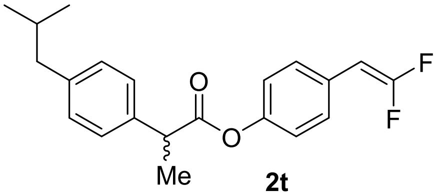
**2t**

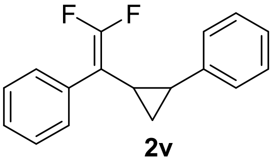
**2v**

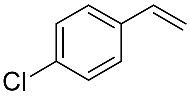
**5a**

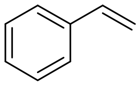
**5d**

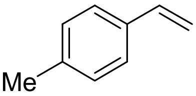
**5f**

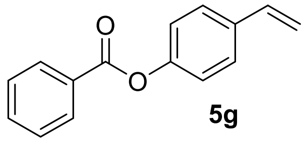
**5g**

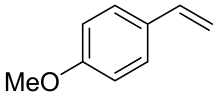
**5h**

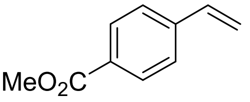
**5j**

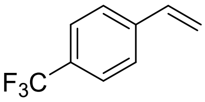
**5k**

Alkenes **2a–2r**, **2t**, **5a–5k** are known compounds. Alkenes **2s**, **2v** are new compounds and were synthesised by the following procedure.

**Preparation of 4-(2,2-difluorovinyl)phenyl 4-(*N*,*N*-dipropylsulfamoyl)benzoate (2s)**

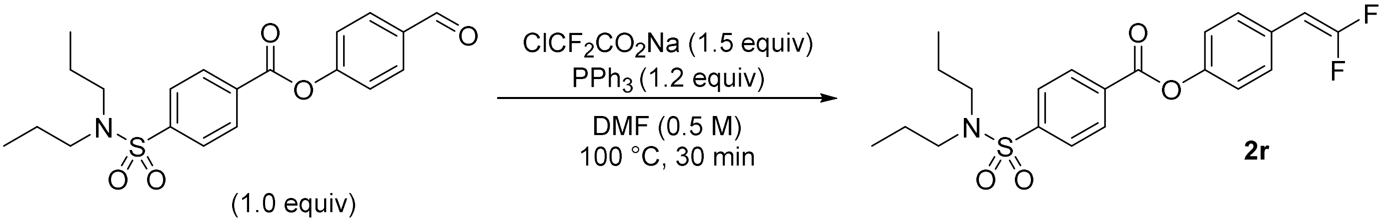


The difluoroalkene **2s** was prepared by procedure according to literature.[1] Added Sodium chlorodifluoroacetate (1.8 g, 12.0 mmol, 1.5 equiv,) in the mixture of 4-formylphenyl 4-(*N*,*N*-dipropylsulfamoyl)benzoate (3.1 g, 8.0 mmol, 1.0 equiv.) in DMF (16.0 mL, 0.5 M). Added triphenyl phosphine (2.5 g, 9.6 mmol, 1.2 equiv,). Heated the reaction at 100 °C and kept at this temperature. After 30 min, cooled to room temperature. Added water to the reaction slowly and extract the mixture with $Et_2O$. Dried the combine

organic layer over $Na_2SO_4$, filter and concentrate in vacuo. The resulting mixture was purified by silica gel chromatography to obtain the product **2s** (2.1 g, 5.0 mmol, 62% yield).

White solid. **Mp**. = 78.4 – 80.1 °C (Chloroform). **HRMS** (ESI) *m/z*: $[M + H]^+$ calculated for $C_{21}H_{24}F_2NO_4S$ 424.1394; found: 424.1403. **$^1$H NMR** (500 MHz, $CDCl_3$) δ 8.33-8.31 (m, 2H), 7.96-7.94 (m, 2H), 7.42-7.39 (m, 2H), 7.22-7.19 (m, 2H), 5.31 (dd, *J* = 25.9, 3.7 Hz, 1H), 3.15-3.12 (m, 4H), 1.61-1.55 (m, 4H), 0.89 (t, *J* = 7.3 Hz, 6H). **$^{13}$C NMR** (126 MHz, $CDCl_3$) δ 163.9, 156.4 (dd, J = 297.9, 288.8 Hz), 149.5, 145.1, 132.8, 130.9 (2C), 128.9 (q, *J* = 2.5 Hz, 2C), 128.6 (t, *J* = 6.3 Hz), 127.3 (2C), 121.9 (2C), 81.6 (q, *J* = 14.2 Hz), 50.0, 22.0, 11.3. **$^{19}$F NMR** (282 MHz, $CDCl_3$) δ -82.1 to -82.3 (m, 1F), -83.7 to -83.8 (m, 1F). **IR (KBr)**: 3461, 2962, 2663, 2140, 1913, 1723, 1348, 1214, 982, 711 $cm^{-1}$.

**Preparation of 4-(2,2-difluorovinyl)phenyl 4-(*N*,*N*-dipropylsulfamoyl)benzoate (2v)**

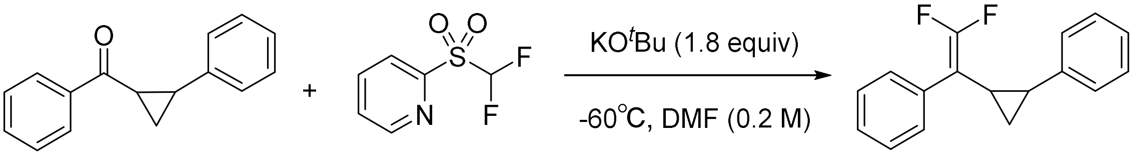


(1.0 equiv) (1.2 equiv) **2u**

The difluoroalkene **2v** was prepared by procedure according to literature.[2] Phenyl(2-phenylcyclopropyl)methanone (2.22 g, 10.0 mmol, 1.0 equiv) and 2-((difluoromethyl)sulfinyl)pyridine (2.32 g, 12.0 mmol, 1.2 equiv) were added to a round bottom flask and purged with nitrogen. DMF (50 mL) was then added, and the reaction was cooled to -60 °C. KO$^t$Bu (2.02 g, 18.0 mmol, 1.8 equiv, 0.5 M in THF) was added via syringe pump over 2 h. The temperature was maintained between -55 and -65 °C. After addition, the reaction mixture was further stirred for 60 min at -60 °C. Saturated $NH_4Cl$ (5 mL) was then added, and the reaction mixture was stirred for 5 min at -60 °C before 6 M HCl (5 mL) was added. After 5 min of stirring at -60 °C, the reaction mixture was warmed to room temp and then to 65 °C for 1 h. After being cooled, the reaction mixture was diluted with brine, extracted (3 x 50 mL) with ethyl acetate, and washed with brine (10 mL). Dried the combine organic layer over $Na_2SO_4$, filter and concentrate in vacuo. The resulting mixture was purified by silica gel chromatography to obtain the product **2v** (1.03 g, 4.0 mmol, 40% yield).

Colorless oil. **HRMS** (ESI) *m/z*: $[M + Na]^+$ calculated for $C_{17}H_{14}F_2Na$ 279.0961; found: 279.0947. **$^1$H NMR** (500 MHz, $CDCl_3$) δ 7.38-7.32 (m, 4H), 7.29-7.26 (m, 3H), 7.20-7.16 (m, 1H), 7.11-7.09 (m, 2H), 1.96-1.88 (m, 2H), 1.29-1.25 (m, 1H), 1.12-1.08 (m, 1H). **$^{13}$C NMR** (126 MHz, $CDCl_3$) δ 154.9 (t, *J* = 292.3 Hz), 141.9, 133.1 (t, *J* = 3.8 Hz), 128.9 (t, *J* = 2.5 Hz, 2C), 128.5 (2C), 128.4 (2C), 127.4, 125.9 (2C), 93.2 (t, *J* = 17.6 Hz), 23.8, 20.9 (d, *J* = 2.5 Hz), 15.5 (d, *J* = 3.8 Hz). **$^{19}$F NMR** (282 MHz, $CDCl_3$) -87.5 (dd, *J* = 576.7, 38.8 Hz, 2F). **IR (KBr)**: 3417, 3028, 2470, 1948, 1713, 1497, 1243, 1110, 864, 695 $cm^{-1}$.

## 4. Amino–trifluoromethoxylation of 2

**General procedure A**

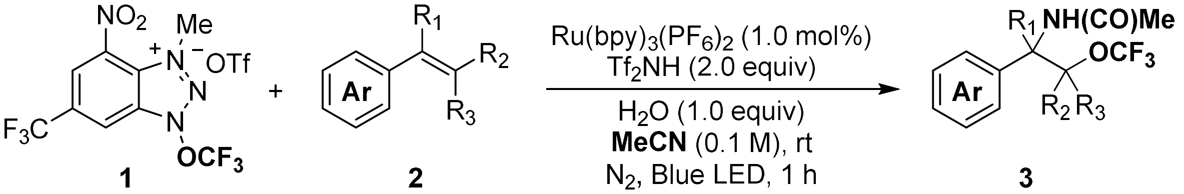


**1** (1.0 equiv) **2** (5.0 equiv) **3**

In a glovebox, to an oven-dried screw cap vial with a magnetic stir bar was added $Ru(bpy)_3(PF_6)_2$ (2.58 mg, 3.0 μmol, 1.0 mol%), **1a** (144 mg, 0.3 mmol, 1.0 equiv), and $Tf_2NH$ (169 mg, 0.6 mmol, 2.0 equiv). Then MeCN (3.0 mL, 0.1 M), difluoroalkene **2** (1.5 mmol, 5.0 equiv), and $H_2O$ (5.4 μL, 0.3 mmol, 1.0 equiv) were added. The vial was capped and taken out of the glovebox. The reaction mixture was then stirred and irradiated with blue LED at room temperature. After 1 h, the resulting mixture was poured into sat. aq. $NaHCO_3$ and extracted with $Et_2O$ three times. The organic layer was washed with brine, and dried over $Na_2SO_4$, then

filtered and concentrated *in vacuo* to give a residue. The residue was purified with silica gel column chromatography to provide pure product.

***N*-(1-(4-chlorophenyl)-2,2-difluoro-2-(trifluoromethoxy)ethyl)acetamide (3a)**

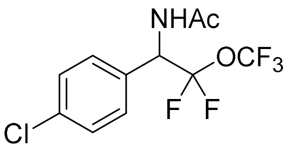


**3a** (63.3 mg, 0.199 mmol, 66% yield) was obtained by **General procedure A** using **1a** (144 mg, 0.3 mmol, 1.0 equiv), alkene **2a** (262 mg, 1.5 mmol, 5.0 equiv). The crude **3a** was purified by flash chromatography on silica gel (Hexane/EtOAc = 9:1 to 6:1).

White solid. **Mp**. = 101.1 – 101.9 °C (Chloroform). **HRMS** (ESI) *m/z*: $[M + H]^+$ calculated for $C_{11}H_{10}F_5NO_2Cl$ 318.0320; found: 318.0319. **$^1$H NMR** (500 MHz, $CDCl_3$) δ 7.39-7.31 (m, 4H), 6.51 (d, $J$ = 9.5 Hz, 1H), 5.69 (q, $J$ = 9.1 Hz, 1H), 2.08 (s, 3H). **$^{13}$C NMR** (126 MHz, $CDCl_3$) δ 169.7, 135.6, 131.4, 129.5-129.3 (m, 4C), 121.6 (t, $J$ = 281.1 Hz), δ 119.5 (q, $J$ = 266.4 Hz), 55.0 (t, $J$ = 28.6 Hz), 23.2. **$^{19}$F NMR** (282 MHz, $CDCl_3$) δ -55.6 (t, $J$ = 9.5 Hz, 3F), -79.7 to -79.9 (m, 2F). **IR (KBr)**: 3247, 3071, 1671, 1547, 1496, 1336, 1280, 1193, 836, 781 $cm^{-1}$.

***N*-(2,2-difluoro-1-(4-fluorophenyl)-2-(trifluoromethoxy)ethyl)acetamide (3b)**

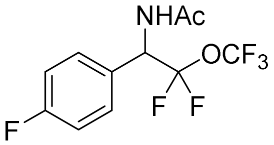


**3b** (58.5 mg, 0.194 mmol, 63% yield) was obtained by **General procedure A** using **1a** (144 mg, 0.3 mmol, 1.0 equiv), alkene **2b** (237 mg, 1.5 mmol, 5.0 equiv). The crude **3b** was purified by flash chromatography on silica gel (Hexane/EtOAc = 9:1 to 6:1).

Pale orange solid. **Mp**. = 88.8 – 89.7 °C (Chloroform). **HRMS** (ESI) *m/z*: $[M + H]^+$ calculated for $C_{11}H_{10}F_6NO_2$ 302.0616; found: 302.0615. **$^1$H NMR** (500 MHz, $CDCl_3$) δ 7.39-7.36 (m, 2H), 7.09-7.05 (m, 2H), 6.93 (d, $J$ = 9.2 Hz, 1H), 5.69 (q, $J$ = 9.1 Hz, 1H), 2.08 (s, 3H). **$^{13}$C NMR** (126 MHz, $CDCl_3$) δ 170.1, 163.2 (d, $J$ = 249.7 Hz), 130.1 (d, $J$ = 9.1 Hz, 2C), 128.8 (d, $J$ = 2.7 Hz), 121.7 (t, $J$ = 281.1 Hz), δ 119.5 (q, $J$ = 266.1 Hz), 116.1 (d, $J$ = 21.8 Hz, 2C), 55.0 (t, $J$ = 29.1 Hz), 23.0. **$^{19}$F NMR** (282 MHz, $CDCl_3$) δ -55.6 (t, J = 9.5 Hz, 3F), -79.9 to -80.0 (m, 2F), -112.2 (s, 1F). **IR (KBr)**: 3294, 3067, 2652, 2263, 1905, 1696, 1379, 1090, 831, 600 $cm^{-1}$.

***N*-(1-(4-bromophenyl)-2,2-difluoro-2-(trifluoromethoxy)ethyl)acetamide (3c)**

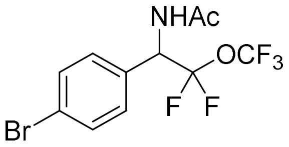


**3c** (73.0 mg, 0.202 mmol, 67% yield) was obtained by **General procedure A** using **1a** (144 mg, 0.3 mmol, 1.0 equiv), alkene **2c** (329 mg, 1.5 mmol, 5.0 equiv). The crude **3c** was purified by flash chromatography on silica gel (Hexane/EtOAc = 9:1 to 6:1).

White solid. **Mp**. = 115.8 – 116.5 °C (Chloroform). **HRMS** (ESI) *m/z*: $[M + H]^+$ calculated for $C_{11}H_{10}F_5NO_2Br$ 361.9815; found: 361.9816. **$^1$H NMR** (500 MHz, $CDCl_3$) δ 7.53-7.52 (m, 2H), 7.26-7.25 (m, 2H), 6.61-6.47 (m, 1H), 5.67 (q, $J$ = 9.0 Hz, 1H), 2.08 (s, 3H). **$^{13}$C NMR** (126 MHz, $CDCl_3$) δ 169.8, 132.3 (2C), 131.9, 129.8 (2C), 123.7, δ 121.5 (t, $J$ = 281.5 Hz), 119.5 (q, $J$ = 266.4 Hz), 55.1 (t, $J$ = 29.1 Hz), 23.2. **$^{19}$F NMR** (282 MHz, $CDCl_3$) δ -55.6 (t, $J$ = 9.5 Hz, 3F), -79.7 to -79.9 (m, 2F). **IR (KBr)**: 3294, 3072, 2976, 2822, 1670, 1550, 1375, 1129, 1010, 779 $cm^{-1}$.

***N*-(2,2-difluoro-1-phenyl-2-(trifluoromethoxy)ethyl)acetamide (3d)**

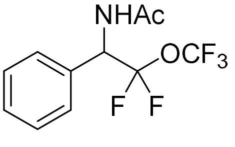


**3d** (53.3 mg, 0.188 mmol, 63% yield) was obtained by **General procedure A** using **1a** (144 mg, 0.3 mmol, 1.0 equiv), alkene **2d** (210 mg, 1.5 mmol, 5.0 equiv). The crude **3d** was purified by flash chromatography on silica gel (Hexane/EtOAc = 9:1 to 6:1).

White solid. **Mp**. = 85.3 – 86.0 °C (Chloroform). **HRMS** (ESI) *m/z*: $[M + H]^+$ calculated for $C_{11}H_{11}F_5NO_2$ 284.0710; found: 284.0712. **$^1$H NMR** (500 MHz, $CDCl_3$) δ 7.40-7.37 (m, 5H), 6.65 (d, $J$ = 9.2 Hz, 1H), 5.71 (q, $J$ = 9.1 Hz, 1H), 2.07 (s, 3H). **$^{13}$C NMR** (126 MHz, $CDCl_3$) δ 169.8, 132.9, 129.4, 129.0 (2C), 128.2 (2C), δ 121.8 (t, $J$ = 281.5 Hz), 119.5 (q, $J$ = 265.8 Hz), 55.5 (t,

$J$ = 28.6 Hz), 23.2. **$^{19}$F NMR** (282 MHz, $CDCl_3$) δ -55.6 (t, $J$ = 9.9 Hz, 3F), -79.7 to -79.9 (m, 2F). **IR (KBr)**: 3323, 3073, 2822, 2359, 1958, 1659, 1430, 1163, 1039, 799 $cm^{-1}$.

***N*-(1-([1,1'-biphenyl]-4-yl)-2,2-difluoro-2-(trifluoromethoxy)ethyl)acetamide (3e)**

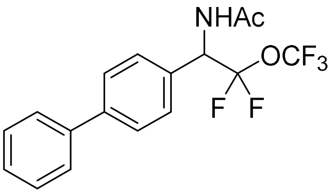


**3e** (28.4 mg, 0.079 mmol, 26% yield) was obtained by **General procedure A** using **1a** (144 mg, 0.3 mmol, 1.0 equiv), alkene **2e** (324 mg, 1.5 mmol, 5.0 equiv). The crude **3e** was purified by flash chromatography on silica gel (Hexane/EtOAc = 9:1 to 6:1).

White solid. **Mp**. = 188.6 – 189.2 °C (Chloroform). **HRMS** (ESI) *m/z*: [M + H]$^+$ calculated for $C_{17}H_{15}F_5NO_2$ 360.1023; found: 360.1028. **$^1$H NMR** (500 MHz, $CDCl_3$) δ 7.63-7.61 (m, 2H), 7.59-7.57 (m, 2H), 7.47-7.44 (m, 4H), 7.39-7.36 (m, 1H), 6.18 (d, $J$ = 9.5 Hz, 1H), 5.76 (q, $J$ = 9.1 Hz, 1H), 2.11 (s, 3H). **$^{13}$C NMR** (126 MHz, $CDCl_3$) δ 169.4, 142.4, 140.2, 131.8, 129.0 (2C), 128.6 (2C), 127.9, 127.8 (2C), 127.3 (2C), 121.8 (t, $J$ = 281.5 Hz), 119.6 (q, $J$ = 266.1 Hz), 55.2 (t, $J$ = 28.6 Hz), 23.4. **$^{19}$F NMR** (282 MHz, $CDCl_3$) δ -55.6 (t, $J$ = 9.5 Hz, 3F), -79.7 to -79.8 (m, 2F). **IR (KBr)**: 3307, 3065, 2344, 1917, 1665, 1545, 1277, 1123, 972, 720 $cm^{-1}$.

***N*-(2,2-difluoro-1-(p-tolyl)-2-(trifluoromethoxy)ethyl)acetamide (3f)**

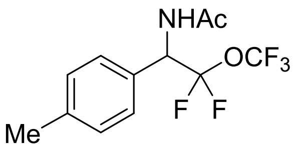


**3f** (51.7 mg, 0.174 mmol, 58% yield) was obtained by **General procedure A** using **1a** (144 mg, 0.3 mmol, 1.0 equiv), alkene **2f** (231 mg, 1.5 mmol, 5.0 equiv). The crude **3f** was purified by flash chromatography on silica gel (Hexane/EtOAc = 9:1 to 6:1).

White solid. **Mp**. = 109.9 – 110.8 °C (Chloroform). **HRMS** (ESI) *m/z*: [M + H]$^+$ calculated for $C_{12}H_{13}F_5NO_2$ 298.0866; found: 298.0865. **$^1$H NMR** (500 MHz, $CDCl_3$) δ 7.27-7.26 (m, 2H), 7.19-7.18 (m, 2H), 6.71 (d, $J$ = 9.5 Hz, 1H), 5.67 (q, $J$ = 9.1 Hz, 1H), 2.35 (s, 3H), 2.05 (s, 3H). **$^{13}$C NMR** (126 MHz, $CDCl_3$) δ 169.8, 139.4, 129.9, 129.7 (2C), 128.1 (2C), 121.9 (t, $J$ = 280.6 Hz), δ 119.5 (q, $J$ = 266.1 Hz), 55.2 (t, $J$ = 28.6 Hz), 23.1, 21.3. **$^{19}$F NMR** (282 MHz, $CDCl_3$) δ -55.6 (t, $J$ = 9.9 Hz, 3F), -79.8 to -79.9 (m, 2F). **IR (KBr)**: 3335, 3072, 2826, 2523, 1914, 1655, 1425, 1121, 974, 734 $cm^{-1}$.

**4-(1-acetamido-2,2-difluoro-2-(trifluoromethoxy)ethyl)phenyl benzoate (3g)**

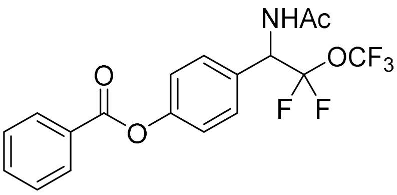


**3g** (57.7 mg, 0.143 mmol, 48% yield) was obtained by **General procedure A** using **1a** (144 mg, 0.3 mmol, 1.0 equiv), alkene **2g** (390 mg, 1.5 mmol, 5.0 equiv). The crude **3g** was purified by flash chromatography on silica gel (Hexane/EtOAc = 9:1 to 6:1).

White solid. **Mp**. = 159.8 – 160.4 °C (Chloroform). **HRMS** (ESI) *m/z*: [M + Na]$^+$ calculated for $C_{18}H_{14}F_5NO_4Na$ 426.0741; found: 426.0748. **$^1$H NMR** (500 MHz, $CDCl_3$) δ 8.20-8.19 (m, 2H), 7.67-7.64 (m, 1H), 7.54-7.51 (m, 2H), 7.46-7.44 (m, 2H), 7.27-7.25 (m, 2H), 6.40 (d, $J$ = 9.5 Hz, 1H), 5.76 (q, $J$ = 9.1 Hz, 1H), 2.08 (s, 3H). **$^{13}$C NMR** (126 MHz, $CDCl_3$) δ 169.6, 165.1, 151.7, 134.0, 130.6, 130.4 (2C), 129.5 (2C), 129.3, 128.8 (2C), 122.5 (2C), 121.7 (t, $J$ = 282.2 Hz), 119.5 (q, $J$ = 267.1 Hz), 55.0 (t, $J$ = 29.0 Hz), 23.2. **$^{19}$F NMR** (282 MHz, $CDCl_3$) δ -55.5 (t, $J$ = 8.2 Hz, 3F), -79.7 (t, $J$ = 8.6 Hz, 2F). **IR (KBr)**: 3311, 2926, 2344, 1911, 1731, 1545, 1185, 1024, 907, 782 $cm^{-1}$

***N*-(2,2-difluoro-1-(4-methoxyphenyl)-2-(trifluoromethoxy)ethyl)acetamide (3h)**

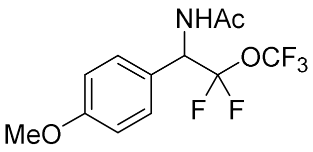


**3h** (36.3 mg, 0.116 mmol, 39% yield) was obtained by **General procedure A** using **1a** (144 mg, 0.3 mmol, 1.0 equiv), alkene **2h** (255 mg, 1.5 mmol, 5.0 equiv). The crude **3h** was purified by flash chromatography on silica gel (Hexane/EtOAc = 9:1 to 7:3).

White solid. **Mp**. = 115.6 – 116.4 °C (Chloroform). **HRMS** (ESI) *m/z*: [M + Na]$^+$ calculated for $C_{12}H_{12}F_5NO_3Na$ 336.0635; found: 336.0637. **$^1$H NMR** (500 MHz, $CDCl_3$) δ 7.30-7.28 (m, 2H), 6.93-6.90 (m, 2H), 6.24 (d, $J$ = 9.5 Hz, 1H), 5.65 (q, $J$ = 9.1 Hz, 1H), 3.82 (s, 3H), 2.07 (s, 3H). **$^{13}$C NMR** (126 MHz, $CDCl_3$) δ 169.4, 160.3, 129.4 (2C), 124.9, 121.9

(t, $J$ = 286.7 Hz), 119.5 (q, $J$ = 268.4 Hz), 114.4, 54.9 (t, $J$ = 28.9 Hz), 23.3. **$^{19}$F NMR** (282 MHz, $CDCl_3$) δ -54.6 (t, $J$ = 9.3 Hz, 3F), -79.0 to -79.1 (m, 2F). **IR (KBr)**: 3324, 3079, 2345, 2840, 1905, 1680, 1345, 1032, 846, 665 $cm^{-1}$.

***N*-(2,2-difluoro-2-(trifluoromethoxy)-1-(4-(trifluoromethoxy)phenyl)ethyl)acetamide (3i)**

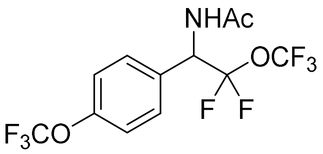


**3i** (59.2 mg, 0.160 mmol, 53% yield) was obtained by **General procedure A** using **1a** (144 mg, 0.3 mmol, 1.0 equiv), alkene **2i** (336 mg, 1.5 mmol, 5.0 equiv). The crude **3i** was purified by flash chromatography on silica gel (Hexane/EtOAc = 9:1 to 6:1).

Pale yellow solid. **Mp**. = 82.1 – 82.9 °C (Chloroform). **HRMS** (ESI) *m*/*z*: $[M + Na]^+$ calculated for $C_{12}H_{10}F_8NO_3$ 368.0533; found: 368.0542. **$^1$H NMR** (500 MHz, $CDCl_3$) δ 7.43-7.41 (m, 2H), 7.25-7.23 (m, 2H), 6.52 (d, $J$ = 9.5 Hz, 1H), 5.73 (q, $J$ = 9.0 Hz, 1H), 2.09 (s, 3H). **$^{13}$C NMR** (126 MHz, $CDCl_3$) δ 169.7, 149.9, 131.6, 129.8 (2C), 123.8-116.3 (m, 3C), 121.4 (2C), 54.9 (t, $J$ = 29.0 Hz), 23.2. **$^{19}$F NMR** (282 MHz, $CDCl_3$) δ -55.6 (t, $J$ = 9.1 Hz, 3F), -58.3 (s, 3F), -79.7 to -79.8 (m, 2F). **IR (KBr)**: 3324, 3075, 2819, 2481, 1914, 1674, 1274, 1043, 973, 670 $cm^{-1}$.

**methyl 4-(1-acetamido-2,2-difluoro-2-(trifluoromethoxy)ethyl)benzoate (3j)**

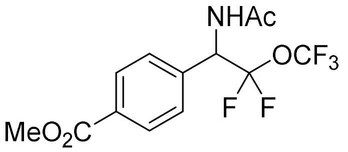


**3j** (39.5 mg, 0.116 mmol, 39% yield) was obtained by **General procedure A** using **1a** (144 mg, 0.3 mmol, 1.0 equiv), alkene **2j** (297 mg, 1.5 mmol, 5.0 equiv). The crude **3j** was purified by flash chromatography on silica gel (Hexane/EtOAc = 9:1 to 6:1).

White solid. **Mp**. = 114.5 – 115.3 °C (Chloroform). **HRMS** (ESI) *m*/*z*: $[M + H]^+$ calculated for $C_{13}H_{13}F_5NO_4$ 342.0765; found: 342.0768. **$^1$H NMR** (500 MHz, $CDCl_3$) δ 8.07-8.05 (m, 2H), 7.48-7.46 (m, 2H), 6.59 (d, $J$ = 9.8 Hz, 1H), 5.78 (q, $J$ = 9.0 Hz, 1H), 3.93 (s, 3H), 2.10 (s, 3H). **$^{13}$C NMR** (126 MHz, $CDCl_3$) δ 169.7, 166.5, 137.6, 131.2, 130.2 (2C), 128.3 (2C), 121.5 (t, $J$ = 281.1 Hz), 119.5 (q, $J$ = 266.4 Hz), 55.3 (t, $J$ = 29.1 Hz), 52.5, 23.2. **$^{19}$F NMR** (282 MHz, $CDCl_3$) δ -55.6 (t, $J$ = 9.9 Hz, 3F), -79.4 to -79.6 (m, 2F). **IR (KBr)**: 3323, 3080, 2850, 2537, 2365, 1938, 1671, 1401, 1113, 816 $cm^{-1}$.

***N*-(2,2-difluoro-1-(3-fluorophenyl)-2-(trifluoromethoxy)ethyl)acetamide (3l)**

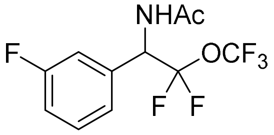


**3l** (33.4 mg, 0.111 mmol, 37% yield) was obtained by **General procedure A** using **1a** (144 mg, 0.3 mmol, 1.0 equiv), alkene **2l** (237 mg, 1.5 mmol, 5.0 equiv). The crude **3l** was purified by flash chromatography on silica gel (Hexane/EtOAc = 9:1 to 6:1).

White solid. **Mp**. = 67.4 – 68.0 °C (Chloroform). **HRMS** (ESI) *m*/*z*: $[M + H]^+$ calculated for $C_{11}H_{10}F_6NO_2$ 302.0616; found: 302.0623. **$^1$H NMR** (500 MHz, $CDCl_3$) δ 7.39-7.35 (m, 1H), 7.18-7.05 (m, 3H), 6.69 (d, $J$ = 9.5 Hz, 1H), 5.71 (q, $J$ = 9.0 Hz, 1H), 2.09 (s, 3H). **$^{13}$C NMR** (126 MHz, $CDCl_3$) δ 169.9, 163.0 (d, $J$ = 248.2 Hz), 132.7 (d, $J$ = 7.5 Hz), 130.5 (d, $J$ = 8.8 Hz), 124.2, 121.6 (t, $J$ = 280.9 Hz), 119.6 (q, $J$ = 265.9 Hz), 116.6 (d, $J$ = 21.4 Hz), 115.3 (d, $J$ = 22.7 Hz), 55.1 (t, $J$ = 28.9 Hz), 23.1. **$^{19}$F NMR** (282 MHz, $CDCl_3$) δ -55.6 (t, $J$ = 9.9 Hz, 3F), -79.7 to -79.8 (m, 2F), -112.1 (q, $J$ = 8.0 Hz, 1F). **IR (KBr)**: 3297, 2927, 2524, 1943, 1596, 1222, 1009, 821, 698, 575 $cm^{-1}$.

***N*-(2,2-difluoro-1-(m-tolyl)-2-(trifluoromethoxy)ethyl)acetamide (3m)**

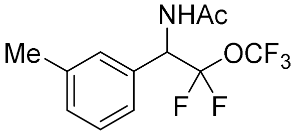


**3m** (35.8 mg, 0.120 mmol, 40% yield) was obtained by **General procedure A** using **1a** (144 mg, 0.3 mmol, 1.0 equiv), alkene **2m** (231 mg, 1.5 mmol, 5.0 equiv). The crude **3m** was purified by flash chromatography on silica gel (Hexane/EtOAc = 9:1 to 6:1).

White solid. **Mp**. = 87.0 – 87.8 °C (Chloroform). **HRMS** (ESI) *m*/*z*: $[M + Na]^+$ calculated for $C_{12}H_{12}F_5NO_2Na$ 320.0686; found: 320.0687. **$^1$H NMR** (500 MHz, $CDCl_3$) δ 7.28-7.26 (m, 1H), 7.21-7.18 (m, 3H), 6.42 (d, $J$ = 9.5 Hz, 1H), 5.67 (q, $J$ = 9.2 Hz, 1H), 2.36 (s, 3H), 2.07 (s, 3H) **$^{13}$C NMR** (126 MHz, $CDCl_3$) δ 169.6, 138.9, 132.8, 130.2, 129.0, 128.9, 125.1, 121.9 (t, $J$ = 281.0 Hz),

119.5 (q, $J$ = 267.1 Hz), 55.5 (t, J = 29.0 Hz), 23.3, 21.5. **$^{19}$F NMR** (282 MHz, $CDCl_3$) δ -55.6 (t, $J$ = 9.5 Hz, 3F), -79.7 to -79.8 (m, 2F). **IR (KBr)**: 3309, 3235, 3073, 1659, 1538, 1377, 1239, 1201, 1106, 889 $cm^{-1}$.

***N*-(2,2-difluoro-1-(2-fluorophenyl)-2-(trifluoromethoxy)ethyl)acetamide (3n)**

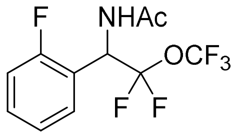


**3n** (22.6 mg, 0.075 mmol, 25% yield) was obtained by **General procedure A** using **1a** (144 mg, 0.3 mmol, 1.0 equiv), alkene **2n** (237 mg, 1.5 mmol, 5.0 equiv). The crude **3n** was purified by flash chromatography on silica gel (Hexane/EtOAc = 9:1 to 6:1).

White solid. **Mp**. = 85.1 – 85.9 °C (Chloroform). **HRMS** (ESI) *m/z*: $[M + H]^+$ calculated for $C_{11}H_{10}F_6NO_2$ 302.0616; found: 302.0624. **$^1$H NMR** (500 MHz, $CDCl_3$) δ 7.41-7.35 (m, 2H), 7.20-7.17 (m, 1H), 7.15-7.11 (m, 1H), 6.54 (d, $J$ = 9.5 Hz, 1H), 5.97 (q, $J$ = 9.2 Hz, 1H), 2.10 (s, 3H). **$^{13}$C NMR** (126 MHz, $CDCl_3$) δ 169.5, 161.2 (d, $J$ = 249.5 Hz) 131.4 (d, $J$ = 8.8 Hz), 130.5 (d, $J$ = 2.5 Hz), 124.8 (d, $J$ = 3.7 Hz), 121.5 (t, $J$ = 282.2 Hz), 119.9 (d, $J$ = 12.6 Hz), 119.4 (q, $J$ = 265.9 Hz), 116.5 (d, $J$ = 22.6 Hz), 51.6 (t, $J$ = 30.2 Hz), 23.3. **$^{19}$F NMR** (282 MHz, $CDCl_3$) δ -55.7 (t, $J$ = 9.5 Hz, 3F), -80.5 to -80.7 (m, 2F), -116.1 (s, 1F). **IR (KBr)**: 3333, 3056, 2345, 1961, 1652, 1253, 1004, 930, 796, 616 $cm^{-1}$.

***N*-(2-(4-bromophenyl)-1,1-difluoro-1-(trifluoromethoxy)propan-2-yl)acetamide (3o)**

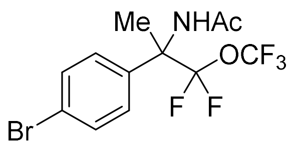


**3o** (49.5 mg, 0.131 mmol, 44% yield) was obtained by **General procedure A** using **1a** (144 mg, 0.3 mmol, 1.0 equiv), alkene **2o** (350 mg, 1.5 mmol, 5.0 equiv). The crude **3o** was purified by flash chromatography on silica gel (Hexane/EtOAc = 9:1 to 6:1).

Pale orange solid. **Mp**. = 113.9 – 114.5 °C (Chloroform). **HRMS** (ESI) *m/z*: $[M + H]^+$ calculated for $C_{12}H_{12}F_5NO_2Br$ 375.9972; found: 375.9974. **$^1$H NMR** (500 MHz, $CDCl_3$) δ 7.49-7.48 (m, 2H), 7.29-7.27 (m, 2H), 6.32 (br s, 1H) 2.02 (s, 6H). **$^{13}$C NMR** (126 MHz, $CDCl_3$) δ 169.2, 135.3, 131.5 (2C), 128.9 (2C), 122.6 (t, $J$ = 283.8 Hz), δ 122.9, 119.6 (q, $J$ = 267.0 Hz), 63.1 (t, $J$ = 25.0 Hz), 24.1, 19.6. **$^{19}$F NMR** (282 MHz, $CDCl_3$) δ -55.6 (t, $J$ = 9.5 Hz, 3F), -84.9 to -85.1 (m, 2F). **IR (KBr)**: 3287, 3068, 2539, 2355, 1912, 1671, 1558, 1221, 977, 823 $cm^{-1}$.

***N*-(2,2-difluoro-1,1-diphenyl-2-(trifluoromethoxy)ethyl)acetamide (3p)**

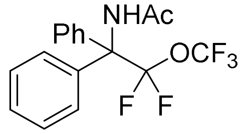


**3p** (46.2 mg, 0.129 mmol, 43% yield) was obtained by **General procedure A** using **1a** (144 mg, 0.3 mmol, 1.0 equiv), alkene **2p** (324 mg, 1.5 mmol, 5.0 equiv). The crude **3p** was purified by flash chromatography on silica gel (Hexane/EtOAc = 95:5 to 8:2).

White solid. **Mp**. = 143.1 – 143.8 °C (Chloroform). **HRMS** (ESI) *m/z*: $[M + H]^+$ calculated for $C_{17}H_{15}NO_2F_5$ 360.1023; found: 360.1028. **$^1$H NMR** (500 MHz, $CDCl_3$) δ 7.37-7.35 (m, 10H), 6.40 (s, 1H), 2.03 (s, 3H). **$^{13}$C NMR** (126 MHz, $CDCl_3$) δ 168.5, 136.8(2C), 128.6(2C), 128.5 (4C), 128.2 (4C), 124.0 (q, $J$ = 320.0 Hz), 120.7 (t, $J$ = 277.0 Hz), 69.9 (t, $J$ = 25.0 Hz), 24.5. **$^{19}$F NMR** (282 MHz, $CDCl_3$) δ -55.4 (t, $J$ = 9.5 Hz, 3F), -73.8 to -73.8 (m, 2F). **IR (KBr)**: 3257, 3201, 3030, 2332, 1965, 1678, 1532, 1273, 1095, 765 $cm^{-1}$.

***N*-(2-fluoro-1-phenyl-2-(trifluoromethoxy)ethyl)acetamide (3q)**

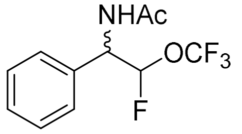


**3q** (34.6 mg, 0.13 mmol, 43% yield) was obtained by **General procedure A** using **1a** (144 mg, 0.3 mmol, 1.0 equiv), alkene **2q** (183.2 mg, 1.5 mmol, 5.0 equiv). The crude **3q** was purified by flash chromatography on silica gel (Hexane/EtOAc = 9:1 to 8:2).

White solid. **Mp**. = 66.8 – 67.5 °C (Chloroform). **HRMS** (ESI) *m/z*: $[M + Na]^+$ calculated for $C_{11}H_{11}F_4NO_2Na$ 288.0624; found: 288.0623. **$^1$H NMR** (500 MHz, $CDCl_3$) major: δ 7.40-7.33 (m, 5H), 6.41 (s, 1H), 5.98 (q, $J$ = 2.6 Hz, 1H), 5.44-5.38 (m, 1H), 2.06 (s, 2H). minor: δ 7.40-7.33 (m, 5H), 6.41 (s, 1H), 6.09 (t, $J$ = 3.1 Hz, 1H), 5.44-5.38 (m, 1H), 2.06 (s, 3H). **$^{13}$C NMR** (126 MHz, $CDCl_3$) δ 170.1 (2C), 134.0, 133.9-133.9 (m, 1C), 129.0 (4C), 128.0 (4C), 124.3-117.9 (m, 4C), 105.8-105.7 (m, 1C), 104.0-103.9

(m, 1C), 55.0-54.5 (m, 2C), 23.2, 23.2. **$^{19}$F NMR** (282 MHz, $CDCl_3$) δ-59.7 to -59.7 (m, 3F), -135.9 to -136.5 (m, 1F). **IR (KBr)**: 3321, 3068, 2853, 2468, 2125, 1669, 1368, 1150, 949, 764 $cm^{-1}$.

**4-(1-acetamido-2,2-difluoro-2-(trifluoromethoxy)ethyl)phenyl 4-(*N,N*-diethylsulfamoyl)benzoate (3s)**

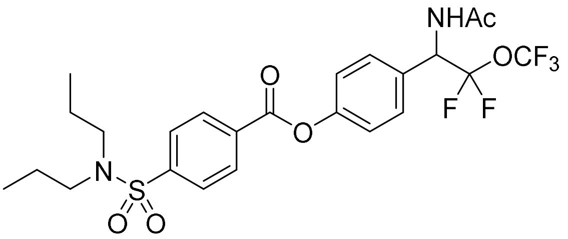


**3s** (86.8 mg, 0.153 mmol, 51% yield) was obtained by **General procedure A** using **1a** (144 mg, 0.3 mmol, 1.0 equiv), alkene **2s** (635 mg, 1.5 mmol, 5.0 equiv). The crude **3s** was purified by flash chromatography on silica gel (Hexane/EtOAc = 3:1 to 2:1).

Orange oil. **HRMS** (ESI) *m/z*: $[M + Na]^+$ calculated for $C_{24}H_{27}F_5N_2O_6SNa$ 589.1408; found: 589.1420. **$^1$H NMR** (500 MHz, $CDCl_3$) δ 8.32-8.30 (m, 2H), 7.96-7.94 (m, 2H), 7.49-7.47 (m, 2H), 7.26-7.24 (m, 2H), 6.72 (d, *J* = 9.5 Hz, 1H), 5.77 (q, *J* = 9.0 Hz, 1H), 3.15-3.12 (m, 4H), 2.09 (s, 3H), 1.61-1.55 (m, 4H), 0.89 (t, *J* = 7.3 Hz, 6H). **$^{13}$C NMR** (126 MHz, $CDCl_3$) δ 169.8, 163.7, 151.2, 145.2, 132.6, 131.0, 129.7, 127.3, 122.2, 121.7 (t, *J* = 281.0 Hz), 119.5 (q, *J* = 267.0 Hz), 54.9 (t, *J* = 29.0 Hz), 50.1, 23.1, 22.0, 11.3, (1C overlap). **$^{19}$F NMR** (282 MHz, $CDCl_3$) δ -55.5 (t, *J* = 8.6 Hz, 3F), -79.7 to -79.7 (m, 2F). **IR (KBr)**: 3304, 3068, 2968, 2255, 1943, 1747, 1513, 1210, 993, 742 $cm^{-1}$.

**4-(1-acetamido-2,2-difluoro-2-(trifluoromethoxy)ethyl)phenyl 2-(4-isobutylphenyl)propanoate (3t)**

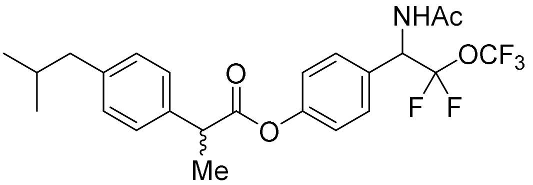


**3t** (71.4 mg, 0.146 mmol, 49% yield) was obtained by **General procedure A** using **1a** (144 mg, 0.3 mmol, 1.0 equiv), alkene **2t** (517 mg, 1.5 mmol, 5.0 equiv). The crude **3t** was purified by flash chromatography on silica gel (Hexane/EtOAc = 9:1 to 6:1).

Pink amorphous. **HRMS** (ESI) *m/z*: $[M + Na]^+$ calculated for $C_{24}H_{26}F_5NO_4Na$ 510.1680; found: 510.1680. **$^1$H NMR** (500 MHz, $CDCl_3$) δ 7.35-7.28 (m, 4H), 7.15-7.13 (m, 2H), 7.06-7.03 (m, 2H), 6.05 (d, *J* = 9.5 Hz, 1H), 5.69 (q, *J* = 9.0 Hz, 1H), 3.98-3.89 (m, 1H), 2.47 (d, *J* = 7.3 Hz, 2H), 2.07 (s, 3H), 1.60 (d, *J* = 7.3 Hz, 3H), 0.91 (d, *J* = 6.7 Hz, 6H). **$^{13}$C NMR** (126 MHz, $CDCl_3$) δ 173.1, 170.0, 151.4, 141.0, 137.0, 130.5, 129.6 (2C), 129.5 (2C), 127.2 (2C), 121.8 (2C), 121.7 (t, *J* = 282.2 Hz), 119.4 (q, *J* = 267.1 Hz), 54.8 (t, *J* = 25.2 Hz), 45.3, 45.1, 40.9, 30.2, 22.9, 22.4 (2C), 18.5. **$^{19}$F NMR** (282 MHz, $CDCl_3$) δ -55.6 (t, *J* = 9.1 Hz, 3F), -79.8 to -79.9 (m, 2F). **IR (KBr)**: 3123, 3008, 2819, 2390, 1786, 1712, 1587, 1401, 1031, 647 $cm^{-1}$.

***N*-(1-(4-chlorophenyl)-2,2-difluoro-2-(trifluoromethoxy)ethyl)propionamide (3u)**

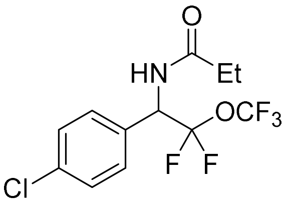


**3u** (36.1 mg, 0.109 mmol, 36% yield) was obtained by **General procedure A** using **1a** (144 mg, 0.3 mmol, 1.0 equiv), alkene **2a** (262 mg, 1.5 mmol, 5.0 equiv). EtCN was used as solvent instead of MeCN. The crude **3u** was purified by flash chromatography on silica gel (Hexane/EtOAc = 9:1 to 6:1). White solid. **Mp**. = 89.2 – 90.0 °C (Chloroform). **HRMS** (ESI) *m/z*: $[M + H]^+$ calculated for $C_{12}H_{12}F_5NO_2Cl$ 332.0477; found: 332.0475. **$^1$H NMR** (500 MHz, $CDCl_3$) δ 7.38-7.36 (m, 2H), 7.32-7.31 (m, 2H), 6.27 (d, *J* = 9.5 Hz, 1H), 5.70 (q, *J* = 9.0 Hz, 1H), 2.36-2.28 (m, 2H), 1.17 (t, J = 7.6 Hz, 3H). **$^{13}$C NMR** (126 MHz, $CDCl_3$) δ 173.3, 135.5, 131.5, 129.5 (2C), 129.3 (2C), 121.6 (t, *J* = 281.0 Hz), 119.5 (q, *J* = 267.1 Hz), 54.9 (t, *J* = 30.2 Hz), 29.6, 9.6. **$^{19}$F NMR** (282 MHz, $CDCl_3$) δ -55.6 (t, *J* = 9.1 Hz, 3F), -79.8 to -79.9 (m, 2F). **IR (KBr)**: 3310, 3065, 2986, 2766, 1915, 1675, 1546, 1251, 1015, 820 $cm^{-1}$.

***N*-(1-(4-chlorophenyl)-2,2-difluoro-2-(trifluoromethoxy)ethyl)acetamide-2,2,2-*d$_3$* (3a-d3)**

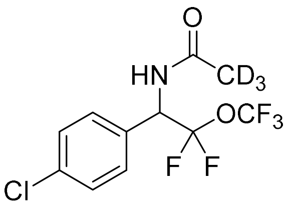


**3a-d3** (52.8 mg, 0.165 mmol, 55% yield) was obtained by **General procedure A** using **1a** (144 mg, 0.3 mmol, 1.0 equiv), alkene **2a** (262 mg, 1.5 mmol, 5.0 equiv). $CD_3CN$ was used as solvent instead of MeCN. The crude **3a-d3** was purified by flash chromatography on silica gel (Hexane/EtOAc = 9:1 to 6:1).

White solid. **Mp**. = 99.3 – 100.0 °C (Chloroform). **HRMS** (ESI) *m/z*: $[M + H]^+$ calculated for $C_{11}H_7D_3F_5NO_2Cl$ 321.0509; found: 321.0509. **$^1$H NMR** (500 MHz, $CDCl_3$) δ 7.38-7.31 (m, 4H), 6.67 (d, $J$ = 9.5 Hz, 1H), 5.69 (q, $J$ = 9.0 Hz, 1H). **$^{13}$C NMR** (126 MHz, $CDCl_3$) δ 169.8, 135.6, 131.4, 129.6-129.3 (m, 4C), 121.6 (t, $J$ = 281.1 Hz), 119.5 (q, $J$ = 266.4 Hz), 55.0 (t, $J$ = 29.1 Hz). **$^{19}$F NMR** (282 MHz, $CDCl_3$) δ -55.6 (t, J = 9.1 Hz, 3F), -79.8 to -79.9 (m, 2F). **IR (KBr)**: 3295, 3067, 2853, 2366, 1918, 1669, 1340, 1119, 968, 778 $cm^{-1}$.

***N*-(5,5-difluoro-1,4-diphenyl-5-(trifluoromethoxy)pent-3-en-1-yl)acetamide (3v)**

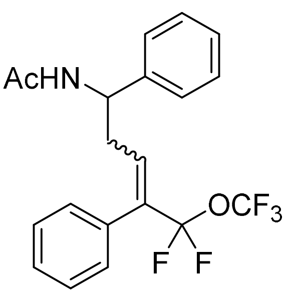


**3v** (32.1 mg, 0.080 mmol, 27% yield) was obtained by **General procedure A** using **1a** (144 mg, 0.3 mmol, 1.0 equiv), alkene **2v** (384 mg, 1.5 mmol, 5.0 equiv). The crude **3v** was purified by flash chromatography on silica gel (Hexane/EtOAc = 95:5 to 90:10 to 70:30).

Orange oil. **HRMS** (ESI) *m/z*: $[M + Na]^+$ calculated for $C_{20}H_{18}F_5NO_2Na$ 422.1155; found: 422.1158. **$^1$H NMR** (500 MHz, $CDCl_3$) δ 7.38-7.36 (m, 2H), 7.33-7.31 (m, 6H), 7.22-7.19 (m, 2H), 5.99 (t, $J$ = 7.6 Hz, 1H), 5.74 (d, $J$ = 8.5 Hz, 1H), 5.26-5.21 (m, 1H), 3.08-3.01 (m, 1H), 2.97-2.90 (m, 1H), 2.01 (s, 3H). **$^{13}$C NMR** (126 MHz, $CDCl_3$) δ 169.6, 140.8, 137.7, 134.4 (t, $J$ = 26.4 Hz), 129.1 (2C), 128.9, 128.5 (2C), 128.4 (2C), 128.1, 127.5, 126.7 (2C), 121.2 (t, $J$ = 277.2 Hz), 119.4 (q, $J$ = 267.1 Hz), 52.8, 29.9, 23.5. **$^{19}$F NMR** (282 MHz, $CDCl_3$) δ -54.6 (t, $J$ = 10.0 Hz, 3F), -62.9 to -63.0 (m, 2F). **IR (KBr)**: 3289, 3087, 2935, 2867, 1954, 1652, 1555, 1237, 1078, 700 $cm^{-1}$.

## 5. Hydroxy–trifluoromethoxylation of 2

**General procedure B**

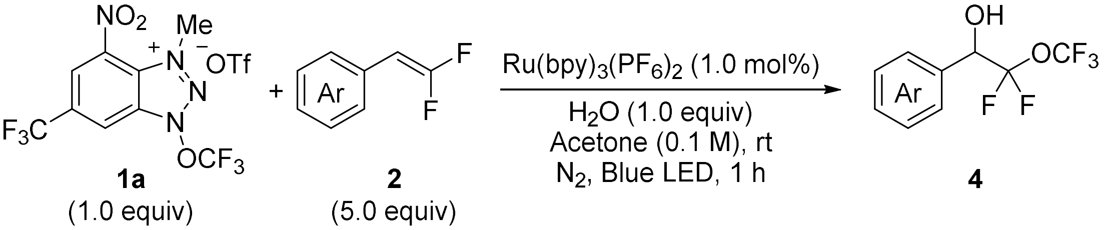


In a glovebox, to an oven-dried screw cap vial with a magnetic stir bar was added $Ru(bpy)_3(PF_6)_2$ (2.58 mg, 3.0 µmol, 1.0 mol%), and **1a** (144 mg, 0.3 mmol, 1.0 equiv). Then acetone (3.0 mL, 0.1 M), styrene **2** (1.5 mmol, 5.0 equiv), and $H_2O$ (5.4 µL, 0.3 mmol, 1.0 equiv) were added. The vial was capped and taken out of the glovebox. The reaction mixture was then stirred and irradiated with blue LED at room temperature. After 1 h, the resulting mixture was poured into sat. aq. $NaHCO_3$ and extracted with $Et_2O$ three times. The organic layer was washed with brine, and dried over $Na_2SO_4$, then filtered and concentrated *in vacuo* to give a residue. The residue was purified with silica gel column chromatography to provide pure product.

**1-(4-chlorophenyl)-2,2-difluoro-2-(trifluoromethoxy)ethan-1-ol (4a)**

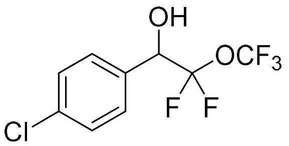


**4a** (34.2 mg, 0.124 mmol, 41% yield) was obtained by **General procedure B** using **1a** (144 mg, 0.3 mmol, 1.0 equiv), alkene **2a** (0.21 mL, 1.5 mmol, 5.0 equiv). The crude **4a** was purified by flash chromatography on silica gel (Hexane/EtOAc = 99:1 to 9:1).

Orange oil. **HRMS** (ESI) *m/z*: $[M - H]^-$ calculated for $C_9H_5F_5O_2Cl$ 274.9898; found: 274.9899. **$^1$H NMR** (500 MHz, $CDCl_3$) δ 7.43-7.38 (m, 4H), 4.99 (t, J = 7.5 Hz, 1H), 2.91 (s, 1H). **$^{13}$C NMR** (126 MHz, $CDCl_3$) δ 135.7, 132.2,

129.1-128.9 (m, 4C), 121.3 (t, $J$ = 281.5 Hz), 119.6 (q, $J$ = 265.8 Hz), 73.3 (t, $J$ = 29.5 Hz). $^{19}$**F NMR** (282 MHz, $CDCl_3$) δ -55.6 (t, $J$ = 9.5 Hz, 3F), -83.9 to -85.4 (m, 2F). **IR (KBr)**: 3420, 2928, 2546, 1912, 1600, 1495, 1217, 1110, 906, 757 $cm^{-1}$.

**4-(2,2-difluoro-1-hydroxy-2-(trifluoromethoxy)ethyl)phenyl benzoate (4g)**

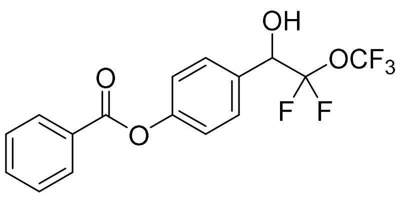


**4g** (46 mg, 0.127 mmol, 43% yield) was obtained by **General procedure B** using **1a** (144 mg, 0.3 mmol, 1.0 equiv), alkene **2g** (390 mg, 1.5 mmol, 5.0 equiv). The crude **4g** was purified by flash chromatography on silica gel (Hexane/EtOAc = 30:1)

White solid. **Mp**. = 93.1 – 93.8 °C (Chloroform). **HRMS** (ESI) *m/z*: $[M + H]^+$ calculated for $C_{16}H_{12}F_5O_4$ 363.0656; found: 363.0658. $^1$**H NMR** (500 MHz, $CDCl_3$) δ 8.21-8.19 (m, 2H), 7.67-7.64 (m, 1H), 7.55-7.51 (m, 4H), 7.28-7.26 (m, 2H), 5.00 (t, $J$ = 7.3 Hz, 1H). $^{13}$**C NMR** (126 MHz, $CDCl_3$) δ 165.2, 151.8, 134.0, 131.5, 130.4 (2C), 129.3, 129.1 (2C), 128.8 (2C), 122.0 (2C), 121.5 (t, $J$ = 282.2 Hz), 119.7 (q, $J$ = 264.6 Hz), 73.4 (t, $J$ = 30.2 Hz). $^{19}$**F NMR** (282 MHz, $CDCl_3$) δ -55.6 (t, J = 9.5 Hz, 3F), -83.7 to -85.4 (m, 2F). **IR (KBr)**: 3448, 2924, 2653, 2169, 1972, 1718, 1602, 1219, 940, 754 $cm^{-1}$.

## 6. Amino–trifluoromethoxylation of 5

**General procedure C**

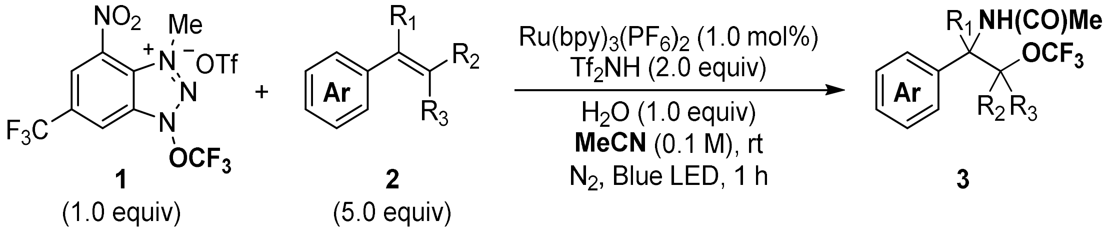


In a glovebox, to an oven-dried screw cap vial with a magnetic stir bar was added $Ru(bpy)_3(PF_6)_2$ (2.58 mg, 3.0 μmol, 1.0 mol%), and **1a** (144 mg, 0.3 mmol, 1.0 equiv). Then MeCN (3.0 mL, 0.1 M), styrene **5** (1.5 mmol, 5.0 equiv), and $H_2O$ (5.4 μL, 0.3 mmol, 1.0 equiv) were added. The vial was capped and taken out of the glovebox. The reaction mixture was then stirred and irradiated with blue LED at room temperature. After 1 h, the resulting mixture was poured into sat. aq. $NaHCO_3$ and extracted with $Et_2O$ three times. The organic layer was washed with brine, and dried over $Na_2SO_4$, then filtered and concentrated *in vacuo* to give a residue. The residue was purified with silica gel column chromatography to provide pure product.

***N*-(1-(4-chlorophenyl)-2-(trifluoromethoxy)ethyl)acetamide (6a)**

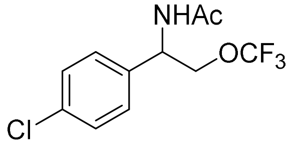


**6a** (46.9 mg, 0.167 mmol, 56% yield) was obtained by **General procedure C** using **1a** (144 mg, 0.3 mmol, 1.0 equiv), alkene **5a** (208 mg, 1.5 mmol, 5.0 equiv). The crude **6a** was purified by flash chromatography on silica gel (Hexane/EtOAc = 9:1 to 6:1).

White solid. **Mp**. = 105.8 – 106.5 °C (Chloroform). **HRMS** (ESI) *m/z*: $[M + H]^+$ calculated for $C_{11}H_{12}F_3NO_2Cl$ 282.0509; found: 282.0509. $^1$**H NMR** (500 MHz, $CDCl_3$) δ 7.34-7.24 (m, 4H), 6.41 (s, 1H), 5.30-5.26 (m, 1H), 4.22-4.16 (m, 2H), 2.03 (s, 3H). $^{13}$**C NMR** (126 MHz, $CDCl_3$) δ 170.0, 136.1, 134.3, 129.2 (2C), 128.4 (2C), 121.6 (q, $J$ = 255.5 Hz), 68.5, 51.3, 23.2. $^{19}$**F NMR** (282 MHz, $CDCl_3$) δ -61.3 (s, 3F). **IR (KBr)**: 3312, 3072, 2852, 2493, 1908, 1661, 1378, 1199, 970, 705 $cm^{-1}$.

***N*-(1-phenyl-2-(trifluoromethoxy)ethyl)acetamide (6d)**

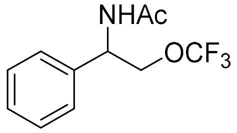


**6d** (49.7 mg, 0.20 mmol, 67% yield) was obtained by **General procedure C** using **1a** (144 mg, 0.3 mmol, 1.0 equiv), alkene **5d** (0.17 mL, 1.5 mmol, 5.0 equiv). The crude **6d** was purified by flash chromatography on silica gel (Hexane/EtOAc = 9:1 to 7:3).

Pale yellow solid. **Mp**. = 67.5 – 68.4 °C (Chloroform). **HRMS** (ESI) *m/z*: $[M + H]^+$ calculated for $C_{11}H_{13}F_3NO_2$ 248.0898; found: 288.0903. **$^1$H NMR** (500 MHz, $CDCl_3$) δ 7.38-7.30 (m, 5H), 6.26 (s, 1H), 5.33-5.29 (m, 1H), 4.23 (t, J = 4.7 Hz, 2H), 2.03 (s, 3H). **$^{13}$C NMR** (126 MHz, $CDCl_3$) δ 169.9, 137.6, 128.9 (2C), 128.4, 127.0 (2C), 121.6 (q, *J* = 255.5 Hz), 68.7, 51.8, 23.3. **$^{19}$F NMR** (282 MHz, $CDCl_3$) δ -61.3 (s, 3F). **IR (KBr)**: 3303, 3081, 2927, 2855, 2486, 2069, 1954, 1640, 1107, 723 $cm^{-1}$.

**_N_-(1-(p-tolyl)-2-(trifluoromethoxy)ethyl)acetamide (6f)**

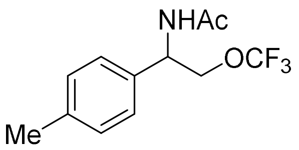


**6f** (21.6 mg, 0.083 mmol, 28% yield) was obtained by **General procedure C** using **1a** (144 mg, 0.3 mmol, 1.0 equiv), alkene **5f** (177 mg, 1.5 mmol, 5.0 equiv). The crude **6f** was purified by flash chromatography on silica gel (Hexane/EtOAc = 9:1 to 6:1).

Pale yellow solid. **Mp**. = 107.0 – 107.7 °C (Chloroform). **HRMS** (ESI) *m/z*: $[M + Na]^+$ calculated for $C_{12}H_{14}F_3NO_2Na$ 284.0874; found: 284.0878. **$^1$H NMR** (500 MHz, $CDCl_3$) δ 7.22-7.15 (m, 4H), 6.10 (d, *J* = 7.3 Hz, 1H), 5.29-5.26 (m, 1H), 4.29-4.15 (m, 2H), 2.34 (s, 3H), 2.03 (s, 3H). **$^{13}$C NMR** (126 MHz, $CDCl_3$) δ 169.8, 138.2, 134.6, 129.7 (2C), 126.9 (2C), 121.7 (q, *J* = 255.5 Hz), 68.7, 51.6, 23.4, 21.2. **$^{19}$F NMR** (282 MHz, $CDCl_3$) δ -61.3 (s, 3F). **IR (KBr)**: 3289, 2970, 2482, 2143, 1900, 1641, 1415, 1120, 1259, 981 $cm^{-1}$.

**4-(1-acetamido-2-(trifluoromethoxy)ethyl)phenyl benzoate (6g)**

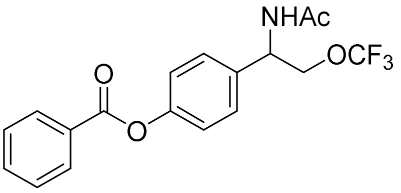


**6g** (22.3 mg, 0.061 mmol, 20% yield) was obtained by **General procedure C** using **1a** (144 mg, 0.3 mmol, 1.0 equiv), alkene **5g** (336 mg, 1.5 mmol, 5.0 equiv). The crude **6g** was purified by flash chromatography on silica gel (Hexane/$Et_2O$ = 30:1).

Pale orange solid. **Mp**. = 116.3 – 117.1 °C (Chloroform). **HRMS** (ESI) *m/z*: $[M + Na]^+$ calculated for $C_{18}H_{16}F_3NO_4Na$ 390.0929; found: 390.0938. **$^1$H NMR** (500 MHz, $CDCl_3$) δ 8.20-8.18 (m, 2H), 7.66-7.62 (m, 1H), 7.53-7.50 (m, 2H), 7.39-7.37 (m, 2H), 7.22-7.21 (m, 2H), 6.24 (d, J = 7.9 Hz, 1H), 5.36-5.32 (m, 1H), 4.27-4.21 (m, 2H), 2.04 (s, 3H). **$^{13}$C NMR** (126 MHz, $CDCl_3$) δ 169.9, 165.3, 150.9, 135.3, 133.9, 130.3 (2C), 129.4, 128.8 (2C), 128.3 (2C), 121.6 (q, *J* = 255.5 Hz), δ 122.2-122.5 (2C) 68.5, 51.3, 23.3. **$^{19}$F NMR** (282 MHz, $CDCl_3$) δ -61.3 (s, 3F). **IR (KBr)**: 3292, 3065, 2924, 2344, 1906, 1642, 1418, 1182, 910, 713 $cm^{-1}$.

**methyl 4-(1-acetamido-2-(trifluoromethoxy)ethyl)benzoate (6j)**

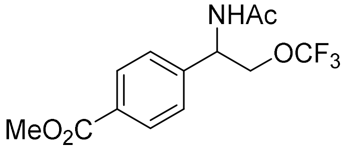


**6j** (44.6 mg, 0.146 mmol, 49% yield) was obtained by **General procedure C** using **1a** (144 mg, 0.3 mmol, 1.0 equiv), alkene **5j** (243 mg, 1.5 mmol, 5.0 equiv). The crude **6j** was purified by flash chromatography on silica gel (Hexane/EtOAc = 3:1).

White solid. **Mp**. = 145.6 – 146.4 °C (Chloroform). **HRMS** (ESI) *m/z*: $[M + H]^+$ calculated for $C_{13}H_{15}F_3NO_4$ 306.0953; found: 306.0952. **$^1$H NMR** (500 MHz, $CDCl_3$) δ 8.05-8.02 (m, 2H), 7.40-7.39 (m, 2H), 6.25 (d, *J* = 6.7 Hz, 1H), 5.40 - 5.37 (m, 1H), 4.28 - 4.23 (m, 2H), 3.92 (s, 3H), 2.08 (s, 3H). **$^{13}$C NMR** (126 MHz, $CDCl_3$) δ 169.9, 166.7, 142.6, 130.3 (2C), 130.2, 127.0 (2C), 121.6 (q, *J* = 257 Hz), 68.6, 52.4, 51.6, 23.3. **$^{19}$F NMR** (282 MHz, $CDCl_3$) δ -61.5 (s, 3F). **IR (KBr)**: 3310, 3079, 2840, 2547, 1905, 1681, 1276, 1034, 962, 787 $cm^{-1}$.

**_N_-(2-(trifluoromethoxy)-1-(4-(trifluoromethyl)phenyl)ethyl)acetamide (6k)**

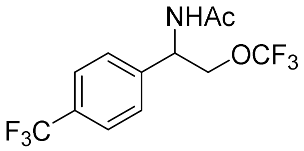


**6k** (44.7 mg, 0.142 mmol, 47% yield) was obtained by **General procedure C** using **1a** (144 mg, 0.3 mmol, 1.0 equiv), alkene **5k** (258 mg, 1.5 mmol, 5.0 equiv). The crude **6k** was purified by flash chromatography on silica gel (Hexane/EtOAc = 9:1 to 7:3).

Pale yellow solid. **Mp**. = 82.9 – 83.7 °C (Chloroform). **HRMS** (ESI) *m/z*: $[M + H]^+$ calculated for $C_{12}H_{12}F_6NO_2$ 316.0772; found: 316.0772. **$^1$H NMR** (500 MHz, $CDCl_3$) δ 7.63-7.61 (m, 2H), 7.45-7.43 (m, 2H), 6.46 (d, *J* = 7.9 Hz,

1H), 5.39-5.35 (m, 1H), 4.26-4.20 (m, 2H), 2.05 (s, 3H). **$^{13}$C NMR** (126 MHz, $CDCl_3$) δ 170.1, 141.6, 130.7 (q, $J$ = 32.7 Hz), 127.4 (2C), 126.0 (d, $J$ = 5.0 Hz, 2C), 124.0 (q, $J$ = 272.2 Hz), 121.5 (q, $J$ = 255.8 Hz), 68.5, 51.6, 23.2. **$^{19}$F NMR** (282 MHz, $CDCl_3$) δ -60.4 (s, 3F), -62.1 (s, 3F). **IR (KBr)**: 3266, 3082, 2484, 2093, 1930, 1653, 1267, 1038, 839, 613 $cm^{-1}$.

## 7. Hydroxy–trifluoromethoxylation of 5

**General procedure D**

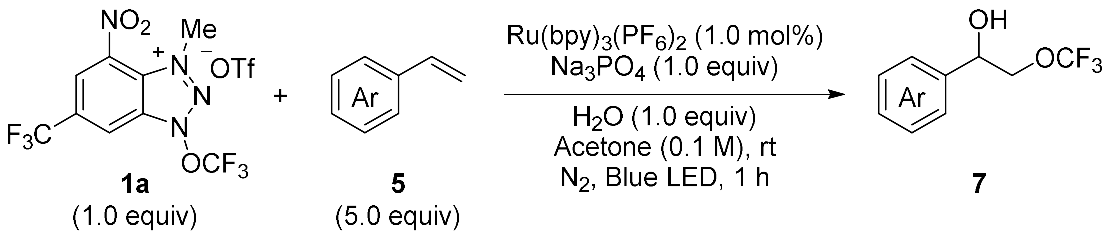


In a glovebox, to an oven-dried screw cap vial with a magnetic stir bar was added $Ru(bpy)_3(PF_6)_2$ (2.58 mg, 3.0 µmol, 1.0 mol%), **1a** (144 mg, 0.3 mmol, 1.0 equiv), and $Na_3PO_4$ (49.2 mg, 0.3 mmol, 1.0 equiv). Then acetone (3.0 mL, 0.1 M), styrene **5** (1.5 mmol, 5.0 equiv), and $H_2O$ (5.4 µL, 0.3 mmol, 1.0 equiv) were added. The vial was capped and taken out of the glovebox. The reaction mixture was then stirred and irradiated with blue LED at room temperature. After 1 h, the resulting mixture was poured into sat. aq. $NaHCO_3$ and extracted with $Et_2O$ three times. The organic layer was washed with brine, and dried over $Na_2SO_4$, then filtered and concentrated *in vacuo* to give a residue. The residue was purified with silica gel column chromatography to provide pure product.

**methyl 4-(1-hydroxy-2-(trifluoromethoxy)ethyl)benzoate (7j)**

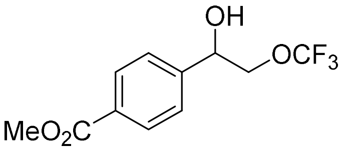


**7j** (36.1 mg, 0.136 mmol, 46% yield) was obtained by **General procedure D** using **1a** (144 mg, 0.3 mmol, 1.0 equiv), alkene **5j** (243 mg, 1.5 mmol, 5.0 equiv). The crude **7j** was purified by flash chromatography on silica gel (Hexane/EtOAc = 95:5 to 90:10 to 80:20).

Yellow oil. **HRMS** (ESI) *m/z*: $[M + H]^+$ calculated for $C_{11}H_{12}F_3O_4$ 265.0688; found: 265.0691. **$^1$H NMR** (500 MHz, $CDCl_3$) δ 8.05-8.03 (m, 2H), 7.48-7.47 (m, 2H), 5.07-5.06 (m, 1H), 4.10-4.08 (m, 1H), 4.04-4.00 (m, 1H), 3.92 (s, 3H). **$^{13}$C NMR** (126 MHz, $CDCl_3$) δ 166.9, 143.5 (d, $J$ = 5.0 Hz), 130.4 (d, $J$ = 2.5 Hz), 130.1 (2C), 126.3 (2C), 121.7 (q, $J$ = 255.8 Hz), 71.5, 52.4, (1C over lap). **$^{19}$F NMR** (282 MHz, $CDCl_3$) δ -60.7 (s, 3F). **IR (KBr)**: 3412, 2960, 2352, 1712, 1612, 1440, 1271, 1144, 1022, 705 $cm^{-1}$.

## 8. Procedure for synthesis of compound 8

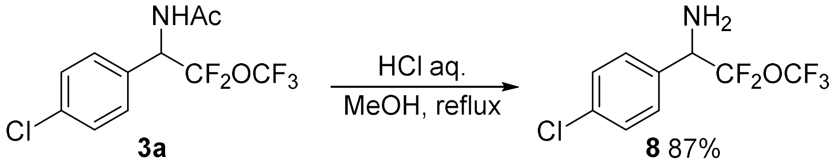


In a 30 mL round bottom flask equiped with condensor, *N*-(1-(4-chlorophenyl)-2,2-difluoro-2-(trifluoromethoxy)ethyl)acetamide **3a** (1.0 equiv, 794 mg, 2.50 mmol) was dissolved in MeOH (8.3 mL, 0.3M). Then, 2M HCl aq. (1.0 mL) was added to the solution at rt. The reaction mixture was stirred and heated under reflux for 14 h. After compelition, the solution was cooled to 0 ºC and quenched with sat. $NaHCO_3$ aq. The mixture was extracted with ethyl acetate 3 times and washed with Brine. The organic phase was dried over anhydrous $Na_2SO_4$ and concentrated under reduced pressure. The crude product was purified by silica gel chromatography (hexane : ethyl acetate = 95:5 to 90:10) to obtain 1-(4-chlorophenyl)-2,2-difluoro-2-(trifluoromethoxy)ethan-1-amine **8** as yellow oil (596.7 mg, 2.17 mmol, 87% yield).

**HRMS** (ESI) *m/z*: $[M + H]^+$ calculated for $C_9H_8F_5NOCl$ 276.0215; found: 276.0218. **$^1$H NMR** (500 MHz, $CDCl_3$) δ 7.37 (s, 4H), 4.36 (t, $J$ = 8.1 Hz, 1H). **$^{13}$C NMR** (126 MHz, $CDCl_3$) δ 135.1, 133.7, 129.5 (2C), 129.0 (2C), 122.7 (t, $J$ = 281.0 Hz), 119.7 (q, $J$ =

267.1 Hz), 58.8 (t, $J = 27.7$ Hz). **$^{19}$F NMR** (282 MHz, $CDCl_3$) δ -55.6 (s, 3F), -82.1 to -83.5 (m, 2F). **IR (KBr)**: 3405, 2929, 2344, 1905, 1598, 1494, 1218, 1094, 830, 675 $cm^{-1}$.

## 9. Procedure for synthesis of compound 10

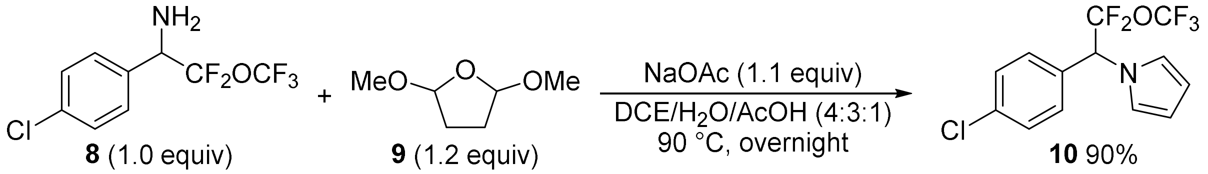


In a screw cap vial, NaOAc (1.1 equiv, 18 mg, 0.22 mmol) was dissolved in a mixture solvent of 1,2-Dichloroethane (0.2 mL), $H_2O$ (0.15 mL) and AcOH (0.5 mL). Then, 1-(4-chlorophenyl)-2,2-difluoro-2-(trifluoromethoxy)ethan-1-amine **8** (1.0 equiv, 55.1 mg, 0.20 mmol) was added to the solution at rt. The solution was stirred and heated to 90 ºC for 5 min. before the addition of 2,5-Dimethoxytetrahydrofuran **9** (1.2 equiv, 0.24 mmol, 31 µL). The solution was stirred at 90 ºC for overnight. After compelition, the solution was cooled to rt, dilluted with $Et_2O$ and washed with sat. $NH_4Cl$ aq. The aqueous layer was extracted with $Et_2O$ 3 times and washed with Brine. The organic phase was dried over anhydrous $Na_2SO_4$ and concentrated under reduced pressure. The crude product was purified by silica gel chromatography (hexane) to obtain 1-(1-(4-chlorophenyl)-2,2-difluoro-2-(trifluoromethoxy)ethyl)-1*H*-pyrrole **10** as colourless oil (58.5 mg, 0.18 mmol, 90% yield).

**HRMS** (ESI) *m/z*: $[M + H]^+$ calculated for $C_{13}H_{10}F_5NOCl$ 326.0371; found: 326.0382. **$^1$H NMR** (500 MHz, $CDCl_3$) δ 7.38-7.31 (m, 4H), 6.77 (s, 2H), 6.23 (s, 2H), 5.52 (t, $J = 9.2$ Hz, 1H). **$^{13}$C NMR** (126 MHz, $CDCl_3$) δ 135.7, 130.8, 129.7-129.4 (m, 4C), 121.3-121.2 (m, 2C), 121.2 (t, $J = 282.0$ Hz), 119.5 (q, $J = 267.0$ Hz), 109.9 (2C), 65.0 (t, $J = 29.1$ Hz). **$^{19}$F NMR** (282 MHz, $CDCl_3$) δ-55.5 (t, $J = 9.5$ Hz, 3F), -76.5 (td, $J = 18.1$, 9.2 Hz, 2F). **IR (KBr)**: 3106, 2929, 2359, 1907, 1720, 1496, 1214, 1096, 915, 725 $cm^{-1}$.

## 10. Procedure for synthesis of compound 12

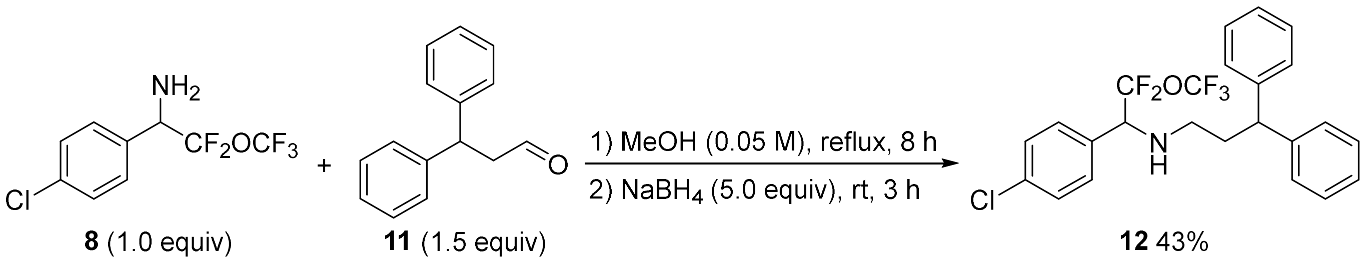


In a 10 mL round bottom flask equiped with condensor, 1-(4-chlorophenyl)-2,2-difluoro-2-(trifluoromethoxy)ethan-1-amine (**8**: 1.0 equiv, 55.1 mg, 0.20 mmol) and 3,3-diphenylpropanal **11** (1.5 equiv, 0.3 mmol, 65 mg) was dissolved in MeOH (0.05 M, 4.0 mL). The reaction mixture was stirred and heated under reflux for 8 h. After compelition, the solution was cooled to rt. Then, $NaBH_4$ (5.0 equiv, 1.0 mmol, 37.8 mg) was added in small portions. The reaction mixture was stirred at rt for 3 h. After compelition, the reaction mixture was quenched with sat. $NH_4Cl$ aq. and extracted with ethyl acetate 3 times. The organic phase was washed with Brine, dried over anhydrous $Na_2SO_4$, and concentrated under reduced pressure. The crude product was purified by silica gel chromatography (hexane : ethyl acetate = 95:5 to 90:10) to obtain *N*-(1-(4-chlorophenyl)-2,2-difluoro-2-(trifluoromethoxy)ethyl)-3,3-diphenylpropan-1-amine **12** as white amorphous (40.6 mg, 0.086 mmol, 43% yield).

**HRMS** (ESI) *m/z*: $[M + H]^+$ calculated for $C_{24}H_{22}F_5NOCl$ 470.1310; found: 470.1325. **$^1$H NMR** (500 MHz, $CDCl_3$) δ 7.31-7.15 (m, 15H), 4.02-3.97 (m, 2H), 2.50-2.47 (m, 2H), 2.27-2.15 (m, 2H). **$^{13}$C NMR** (126 MHz, $CDCl_3$) δ 144.5 (d, $J = 39.1$ Hz, 2C), 135.0, 132.8, 130.1 (2C), 128.9 (2C), 128.7 (4C), 127.9 (d, $J = 10.1$ Hz, 4C), 126.5 (d, $J = 2.5$ Hz, 2C), 122.3 (t, $J = 282.2$ Hz), 119.6 (q, $J = 265.9$ Hz) , 65.3 (t, $J = 26.4$ Hz), 48.7, 45.8, 35.7 **$^{19}$F NMR** (282 MHz, $CDCl_3$) δ -55.5 (t, $J = 9.5$ Hz, 3F), -79.6 to -80.8 (m, 2F). **IR (KBr)**: 3346, 2929, 1946, 1725, 1598, 1493, 1217, 1089, 700, 501 $cm^{-1}$.

## 11. Procedure for synthesis of compound 14

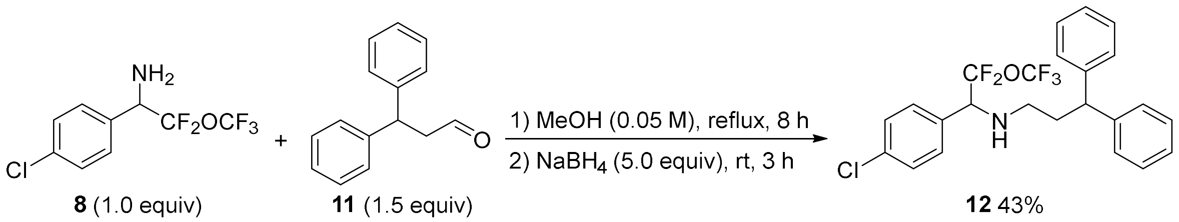


Under $N_2$ atmosphere, in a 10 mL round bottom flask, 1-(4-chlorophenyl)-2,2-difluoro-2-(trifluoromethoxy)ethan-1-amine **8** (1.0 equiv, 55.1 mg, 0.20 mmol) and $Et_3N$ (1.2 equiv, 0.24 mmol, 17.3 µL) was dissolved in toluene (1.0 mL). The reaction mixture was stirred at rt for 5 min., then 2,2-dichloro-1,3-dimethylcyclopropane-1-carbonyl chloride **13** (1.5 equiv, 0.3 mmol) in 1 mL toluene was added slowly at rt. The reaction mixture was stirred at rt for 2 h. After compelition, the reaction mixture was quenched with water and extracted with ethyl acetate 3 times. The organic phase was washed with Brine, dried over anhydrous $Na_2SO_4$, and concentrated under reduced pressure. The crude product was purified by silica gel chromatography (hexane : ethyl acetate = 95:5 to 90:10) to obtain 2,2-dichloro-*N*-(1-(4-chlorophenyl)-2,2-difluoro-2-(trifluoromethoxy)ethyl)-1,3-dimethylcyclopropane-1-carboxamide **14** as white solid (81.6 mg, 0.185 mmol, 93% yield, dr mixture).

**Mp**. = 143.0 – 143.9 °C (Chloroform). **HRMS** (ESI) *m/z*: $[M + H]^+$ calculated for $C_{15}H_{14}F_5NO_2Cl_3$ 440.0010; found: 440.0015. **$^1$H NMR** (500 MHz, $CDCl_3$) δ 7.41-7.33 (m, 4H), 6.34 (dd, *J* = 31.1, 9.5 Hz, 1H), 5.70 (q, J = 9.0 Hz, 1H), 2.35-2.28 (m, 1H), 1.33-1.47 (m, 3H), 1.19 (dd, *J* = 6.6, 4.4 Hz, 3H) **$^{13}$C NMR** (126 MHz, $CDCl_3$) δ 169.1, 168.8, 135.7, 135.7, 131.2, 130.8, 129.6 (2C), 129.5 (2C), 129.4 (2C), 129.3 (2C), 121.6 (t, *J* = 281.0 Hz), 121.4 (t, *J* = 281.0 Hz), 119.5 (q, *J* = 267.1 Hz), 119.5 (q, *J* = 268.3 Hz), 66.7, 66.6, 55.6 (t, *J* = 29.0 Hz), 55.5 (t, *J* = 30.2 Hz), 37.8, 37.8, 30.4, 30.2, 13.4, 13.3, 9.0, 9.0. **$^{19}$F NMR** (282 MHz, $CDCl_3$) δ -55.5 to -55.6 (m, 3F), -79.0 to -80.2 (m, 2F). **IR (KBr)**: 3303, 2972, 2736, 2320, 1909, 1540, 1325, 1122, 877, 679 $cm^{-1}$.

## 12. Light/Dark experiment

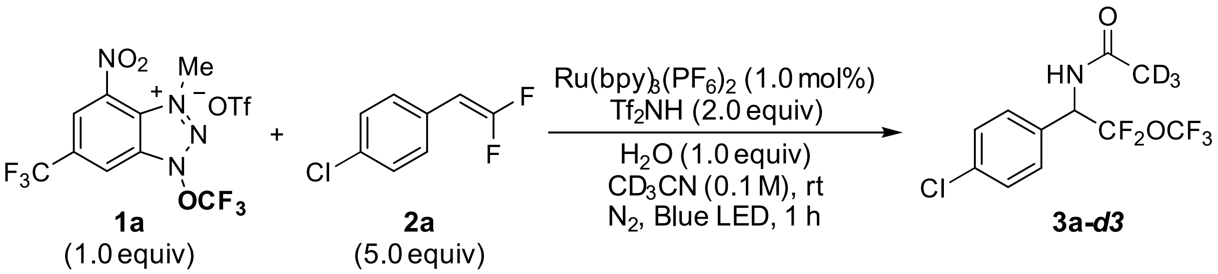


In a glovebox, to an oven-dried screw cap vial with a magnetic stir bar was added $Ru(bpy)_3(PF_6)_2$ (0.86 mg, 1.0 µmol, 1.0 mol%), **1a** (48 mg, 0.1 mmol, 1.0 equiv), and $Tf_2NH$ (56.2 mg, 0.2 mmol, 2.0 equiv). Then $CD_3CN$ (1.0 mL, 0.1 M), difluoroalkene **2a** (0.5 mmol, 5.0 equiv), $H_2O$ (1.8 µL, 0.1 mmol, 1.0 equiv), $C_6F_6$ (1.8 µL, 0.1 mmol, 1.0 equiv) were added. The homogeneous solution was transferred to NMR Tube and taken out of the glovebox. The NMR tube was placed in the photoreactor and irradiated with blue light for 1 min and the crude yield was determined by $^{19}F$ NMR. Another measurement was performed 5 min after the first one. The NMR sample is then irradiated again and process is repeated as previously indicated. The subsequent irradiation times are 1, 1, 2, 2, 5 and 5 min. Note: When not irradiated, the NMR sample is carefully protected from any light source with aluminum foil.

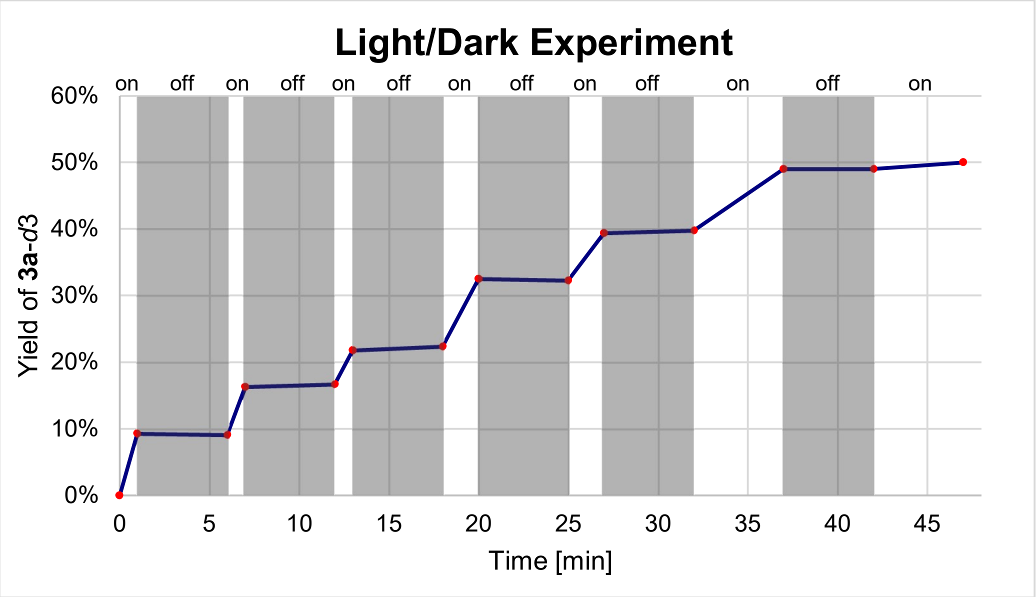


## 13. Reference


1. Hu, J.; Han, X.; Yuan, Y.; Shi, Z. *Angew. Chem. Int. Ed.* **2017**, *56*, 13342 –13346.
2. Gao, P.; Wang, G.; Xi, L.; Wang, M.; Li, Z.; *Chin. J. Chem.*, **2019**, *37*, 1009-1014.

## 14. NMR data

**$^{1}$H NMR** (500 MHz, $CDCl_3$) : **2s**

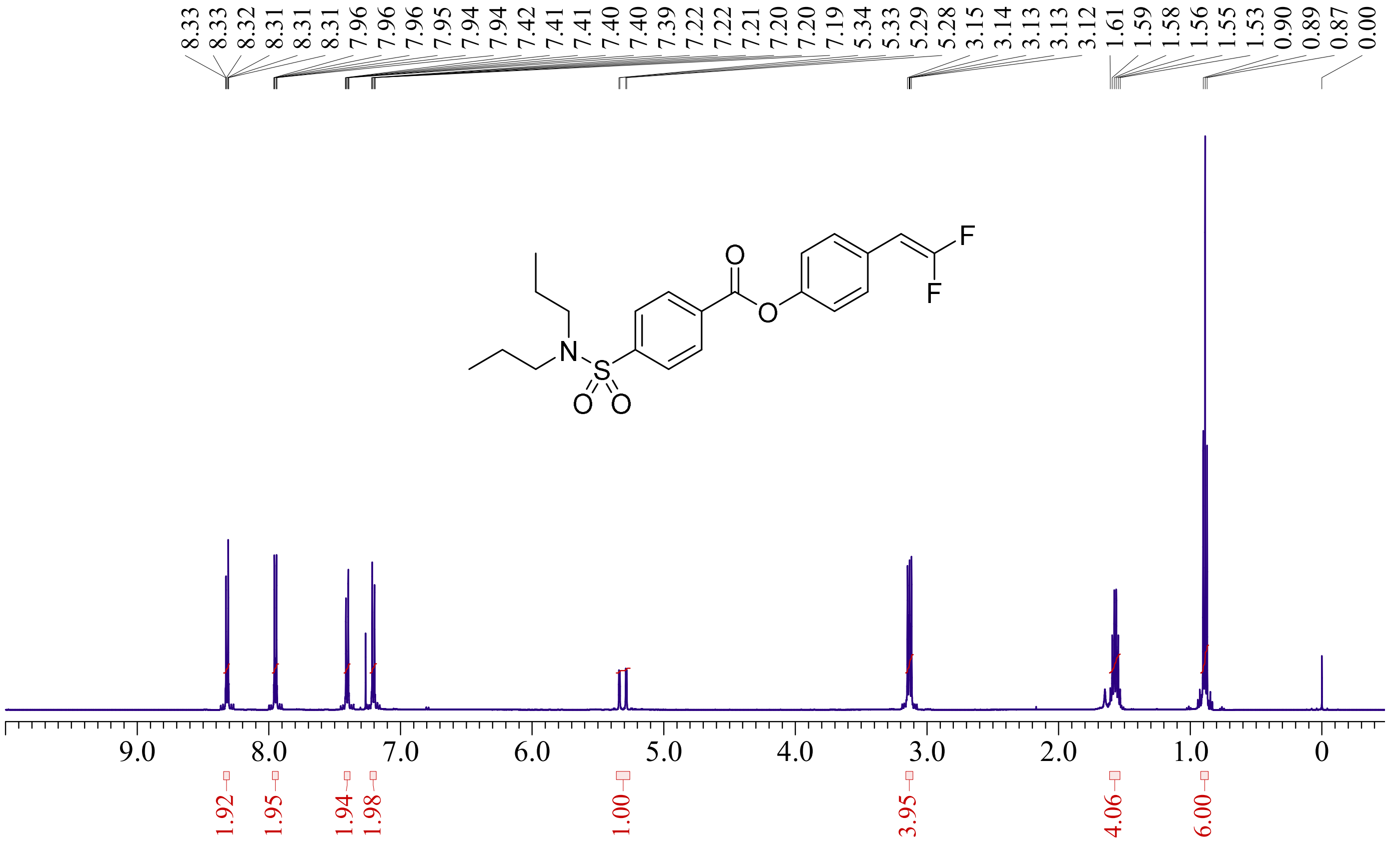


**$^{13}$C NMR** (126 MHz, $CDCl_3$) : **2s**

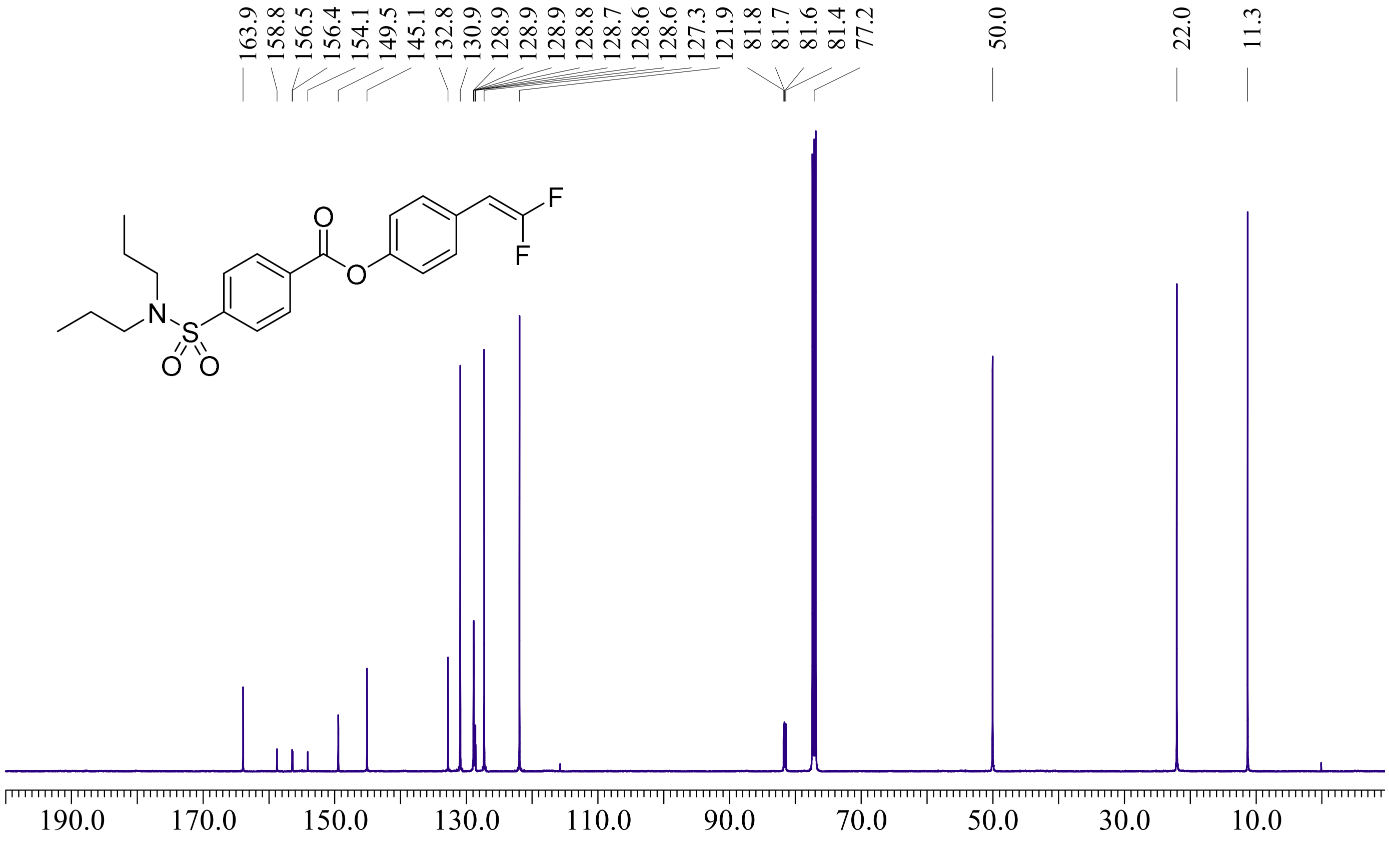

**$^{19}$F NMR** (282 MHz, CDCl3) : **2s**

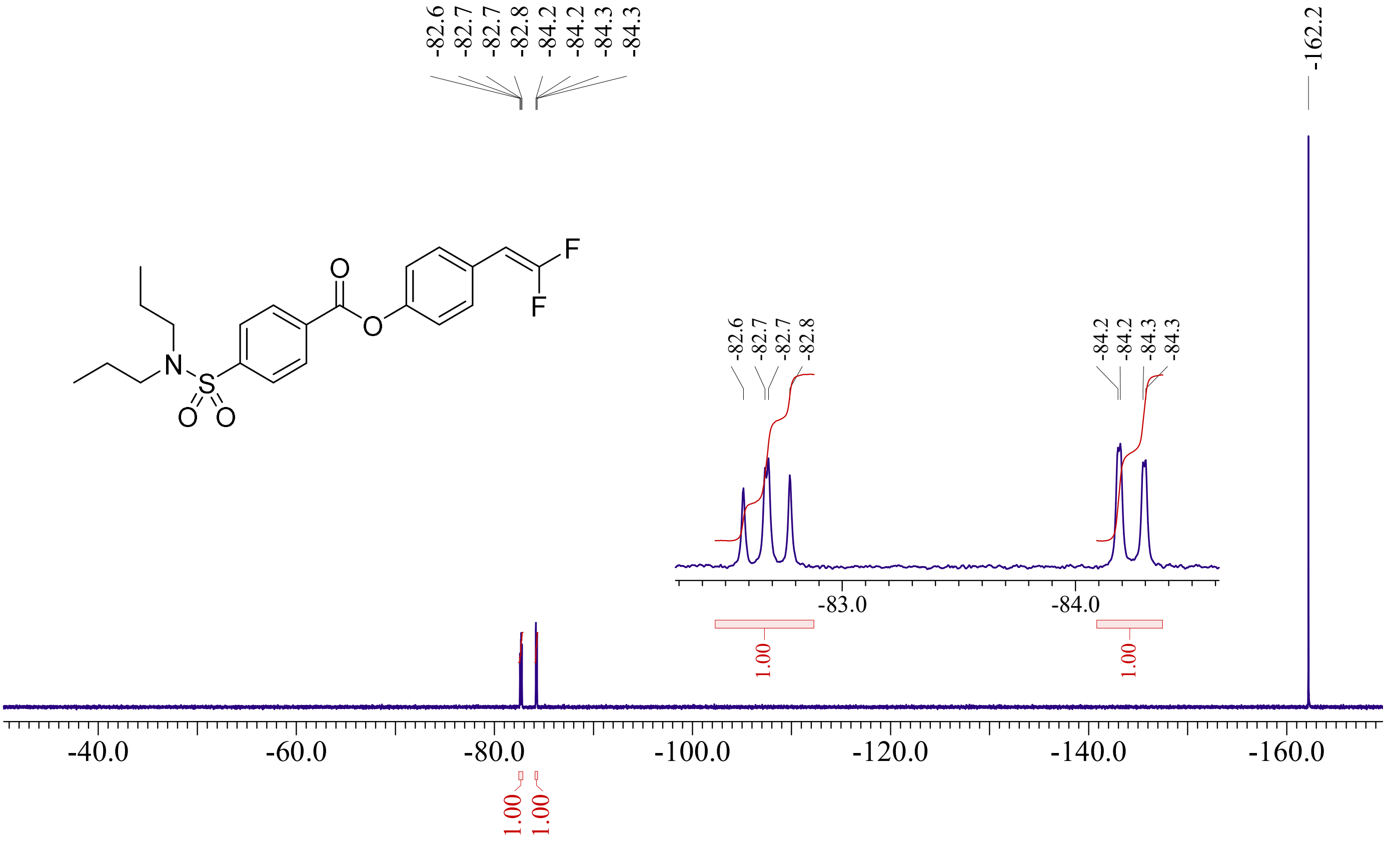

**$^{1}$H NMR** (500 MHz, CDCl3) : **2v**

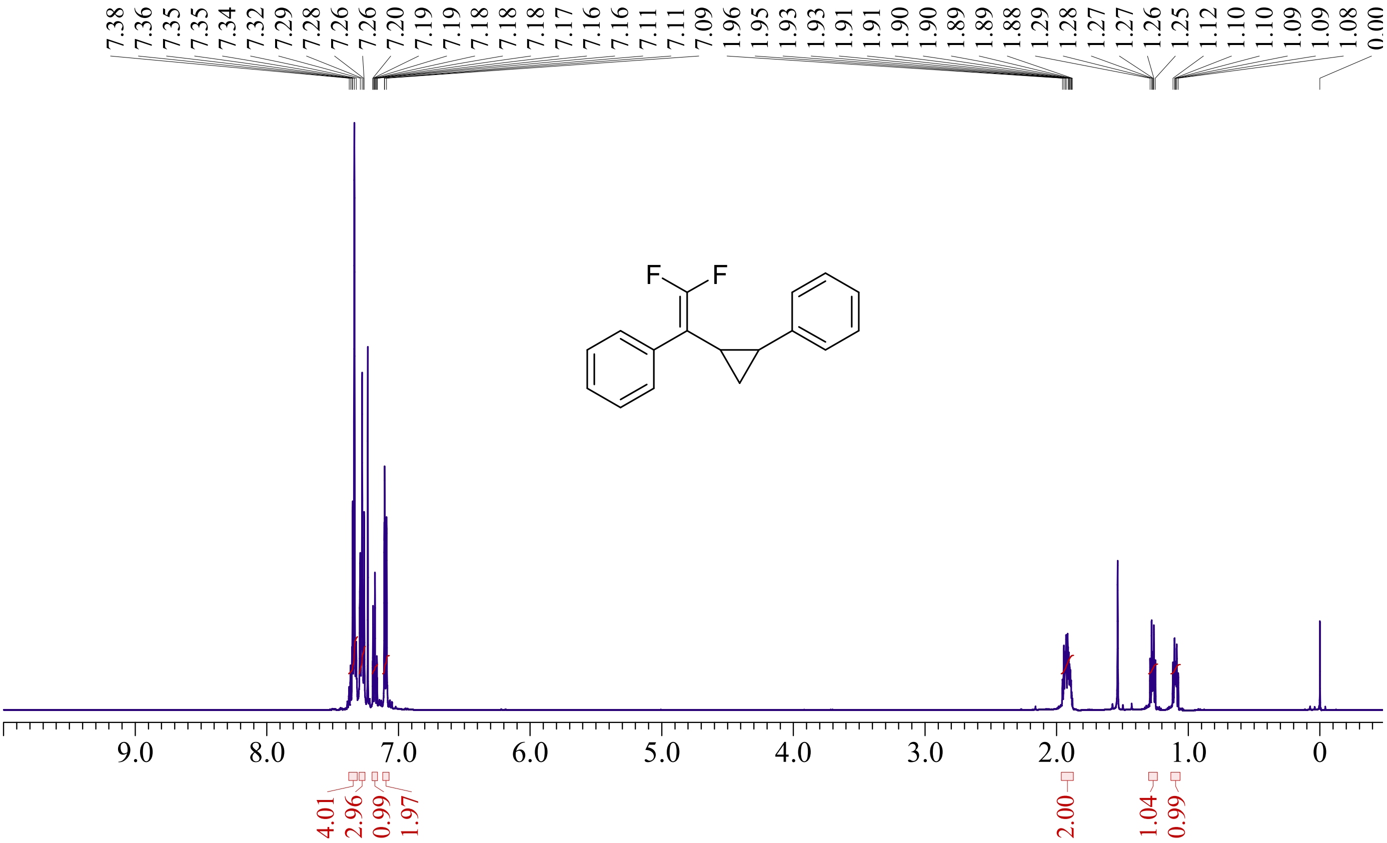

**$^{13}$C NMR** (126 MHz, $CDCl_3$) : **2v**

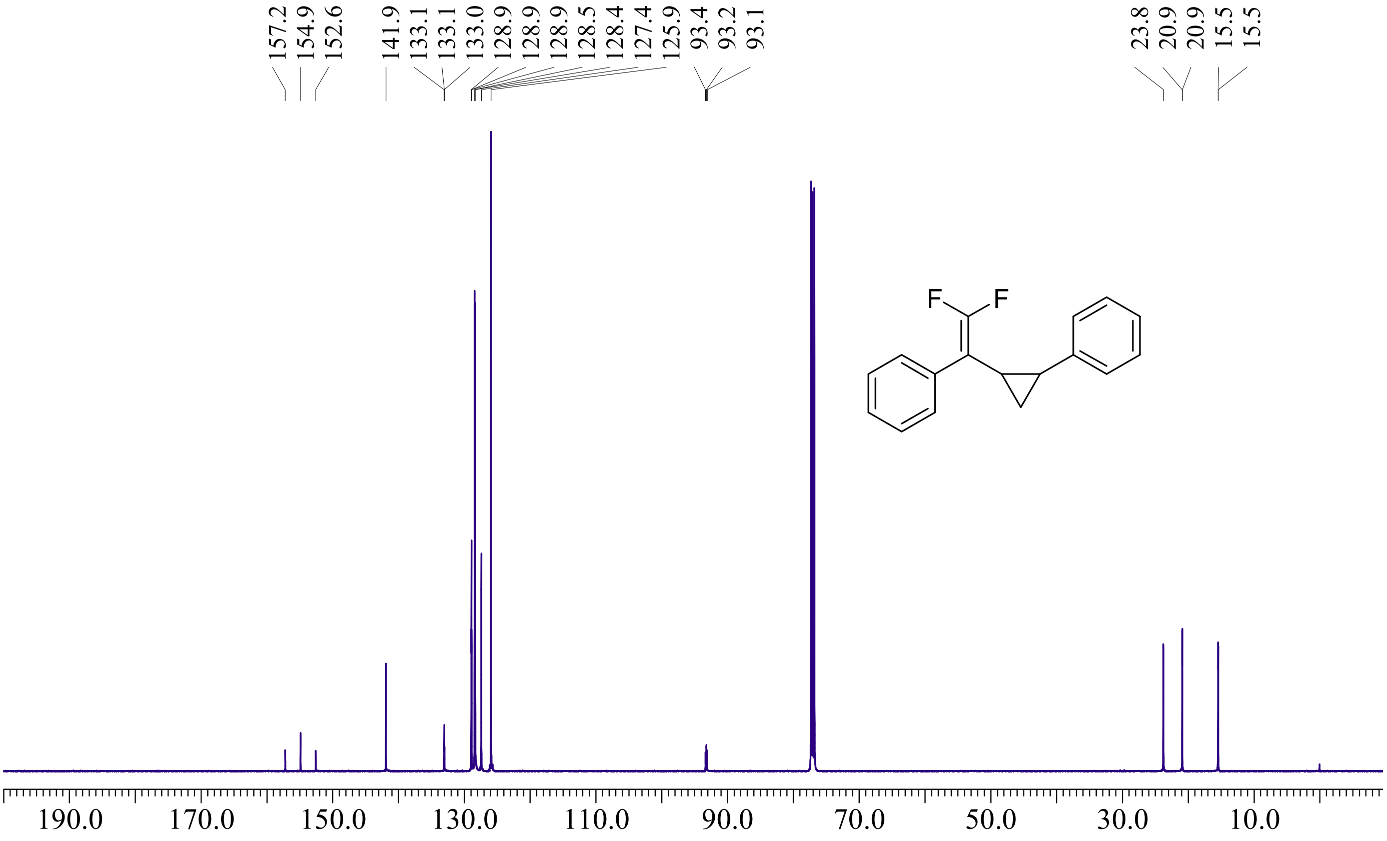


**$^{19}$F NMR** (282 MHz, $CDCl_3$) : **2v**

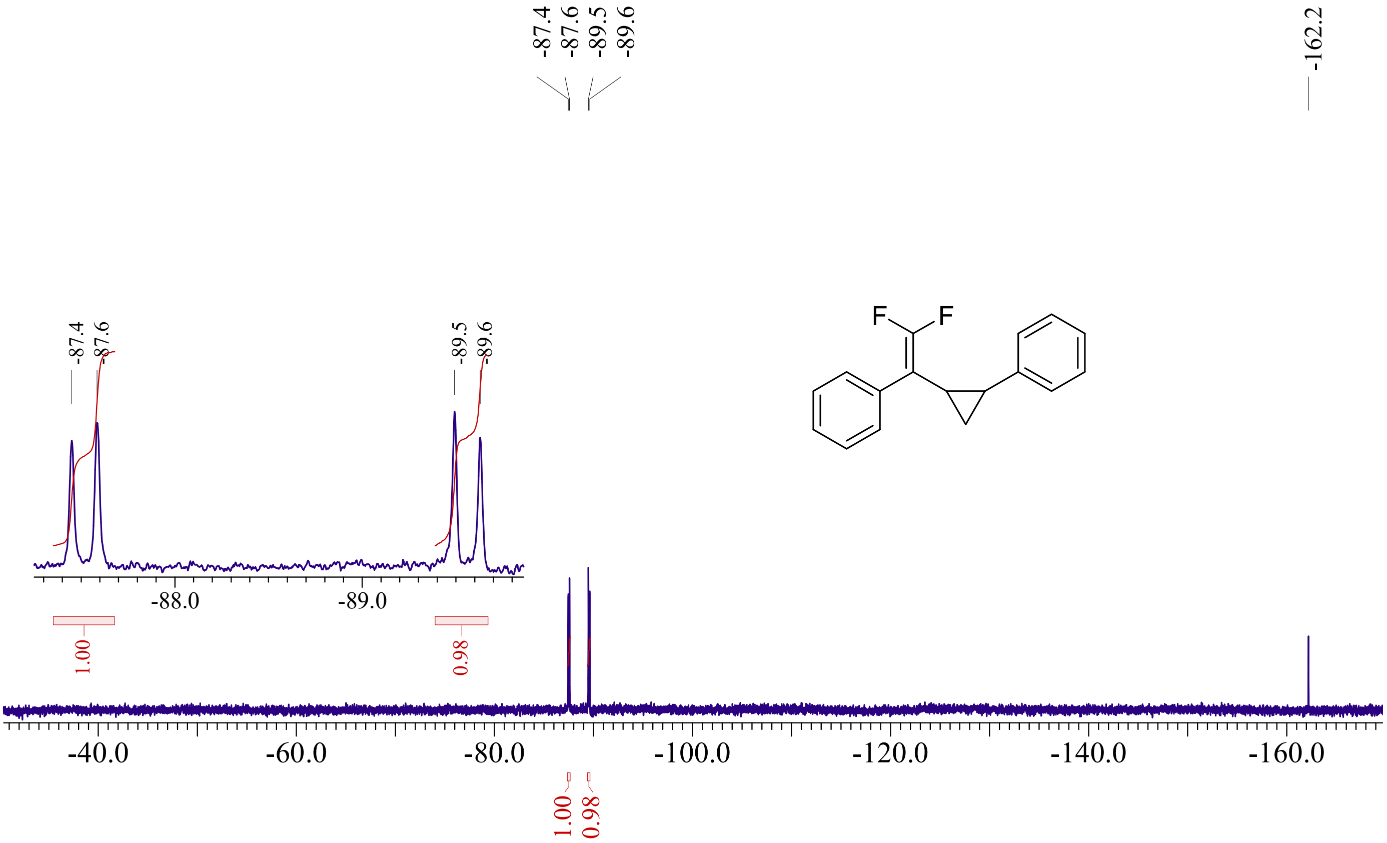

**$^{1}$H NMR** (500 MHz, $CDCl_3$) : **3a**

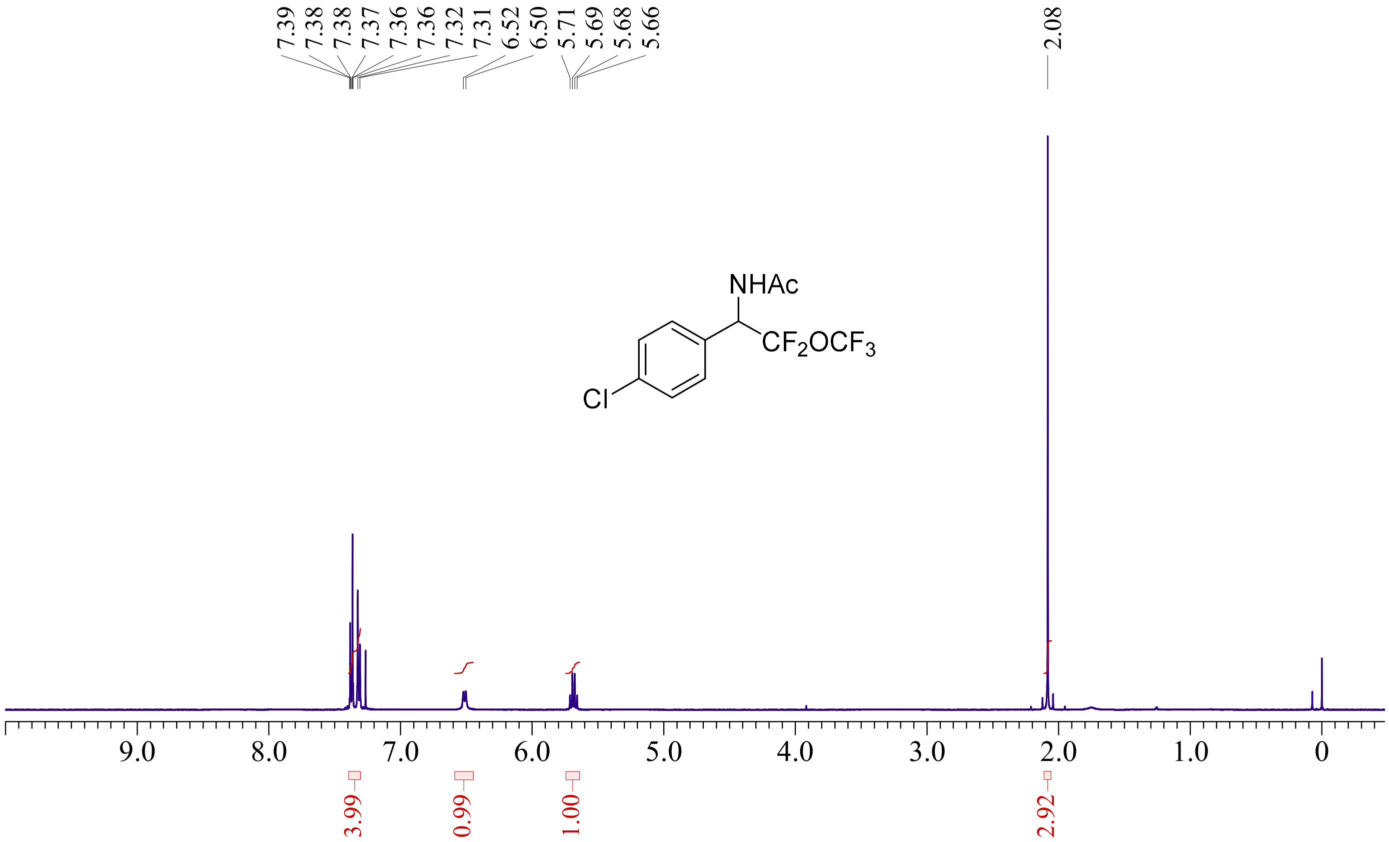


**$^{13}$C NMR** (126 MHz, $CDCl_3$) : **3a**

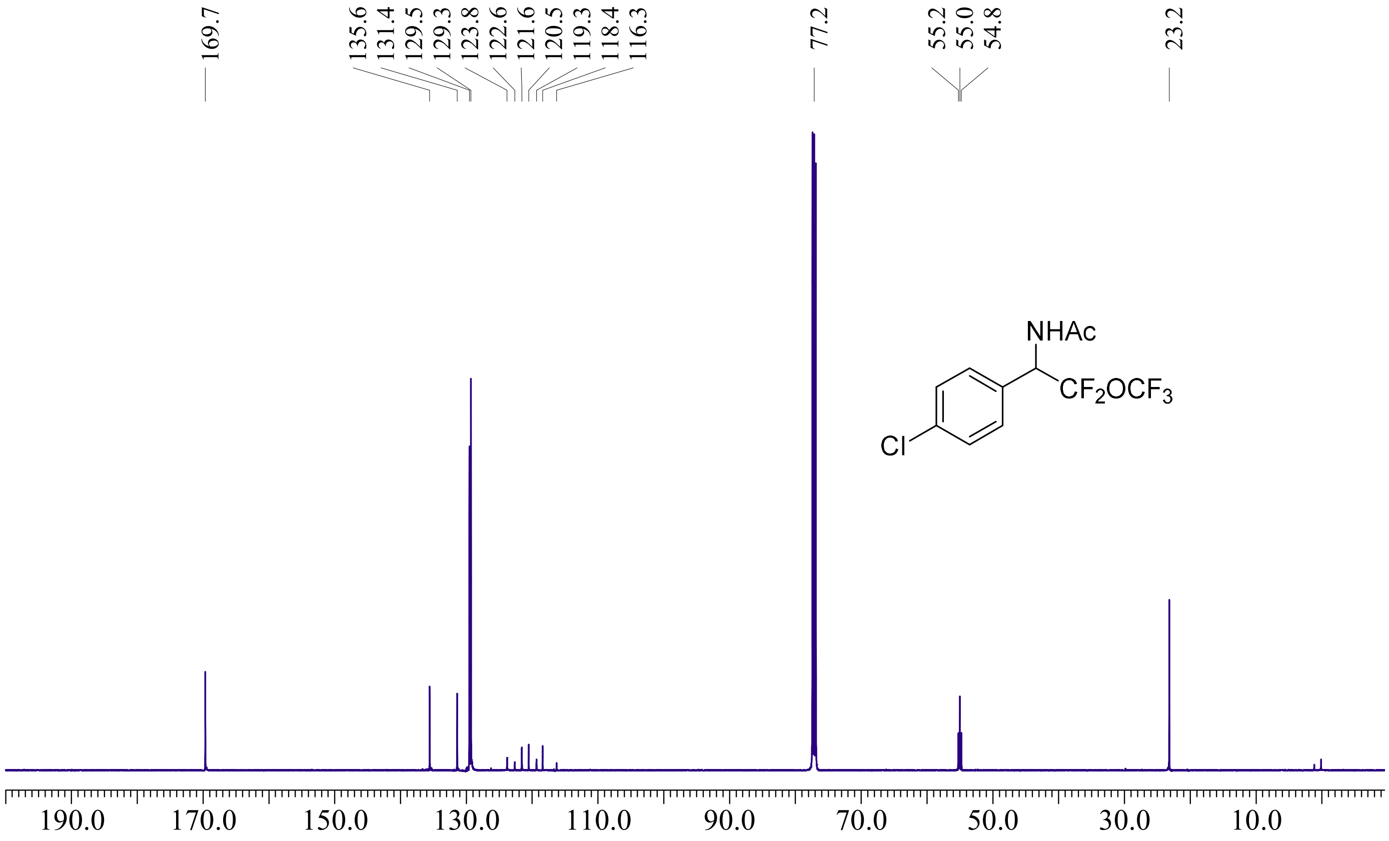

$^{19}$F NMR (282 MHz, $CDCl_3$) : **3a**

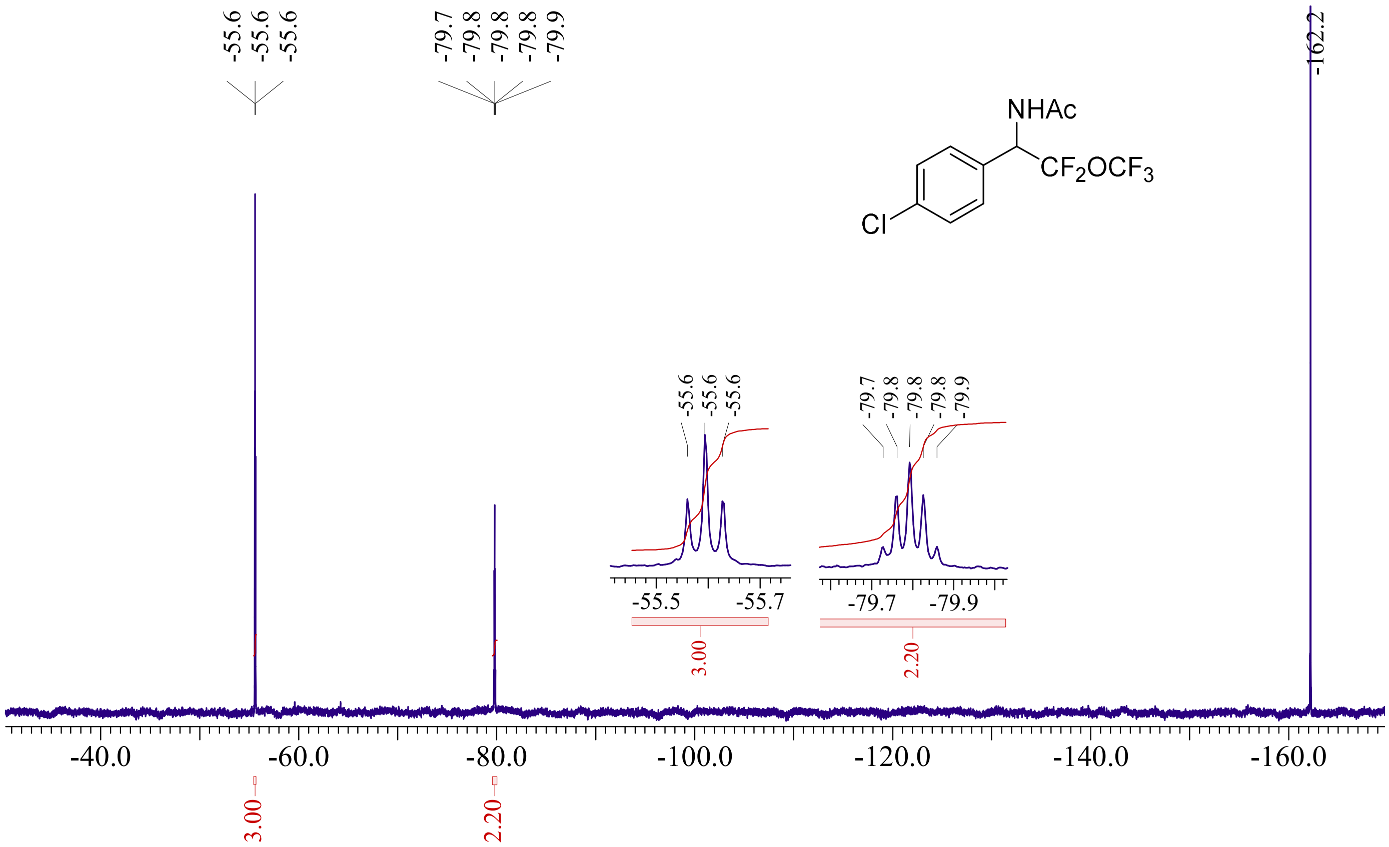


$^{1}$H NMR (500 MHz, $CDCl_3$) : **3b**

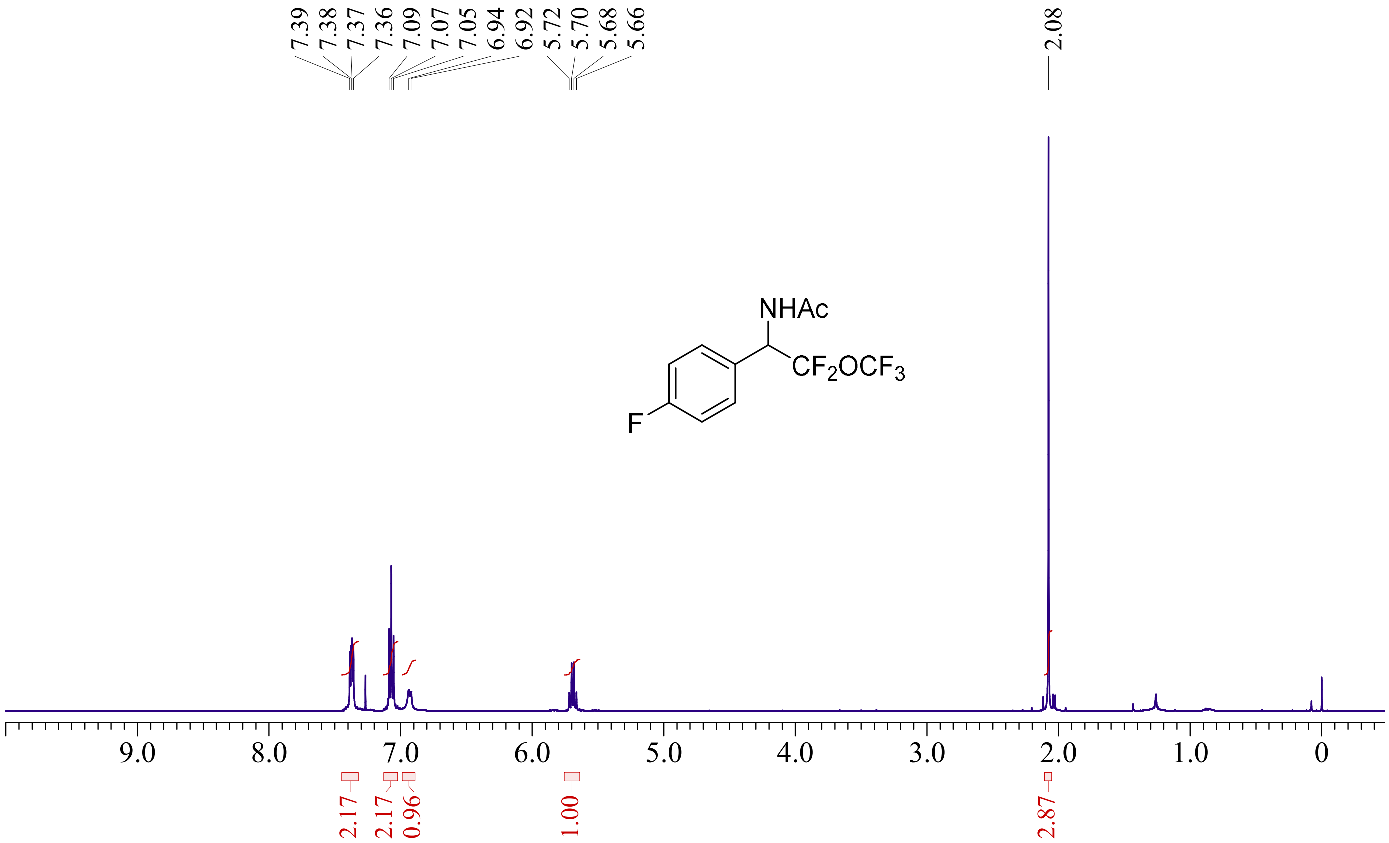

**$^{13}$C NMR** (126 MHz, CDCl$_3$) : **3b**

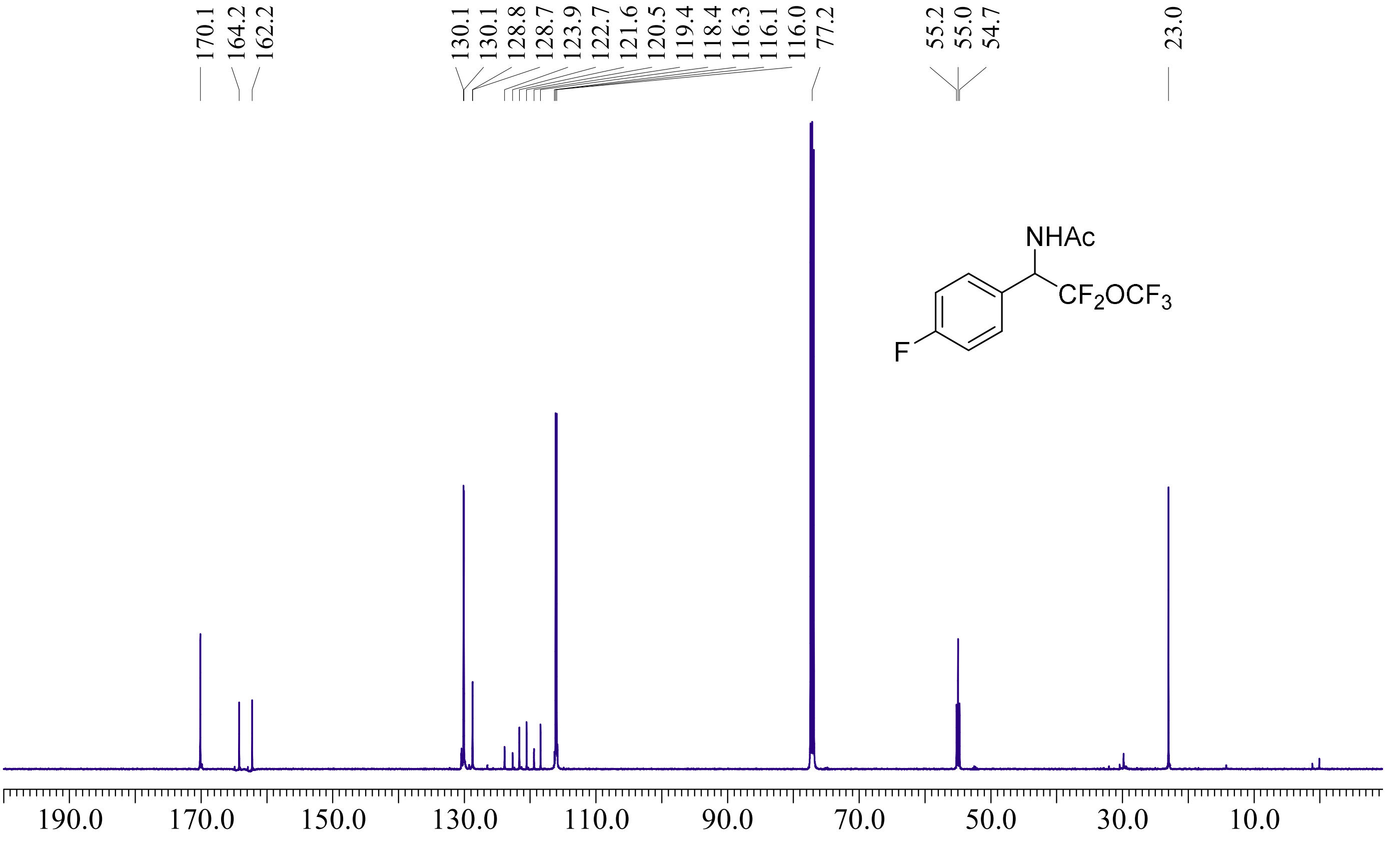


**$^{19}$F NMR** (282 MHz, CDCl$_3$) : **3b**

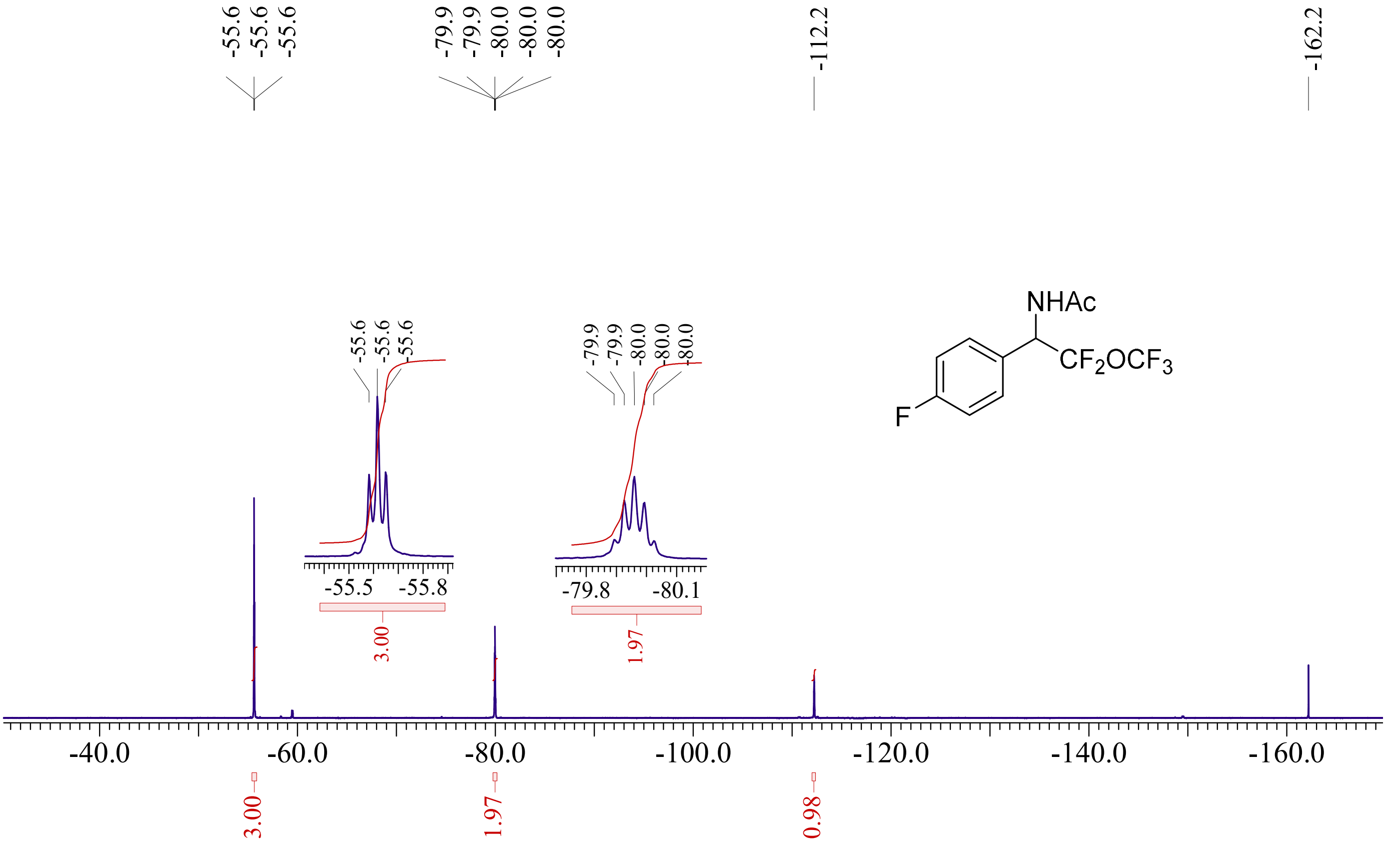

**$^{1}$H NMR** (500 MHz, $CDCl_3$) : **3c**

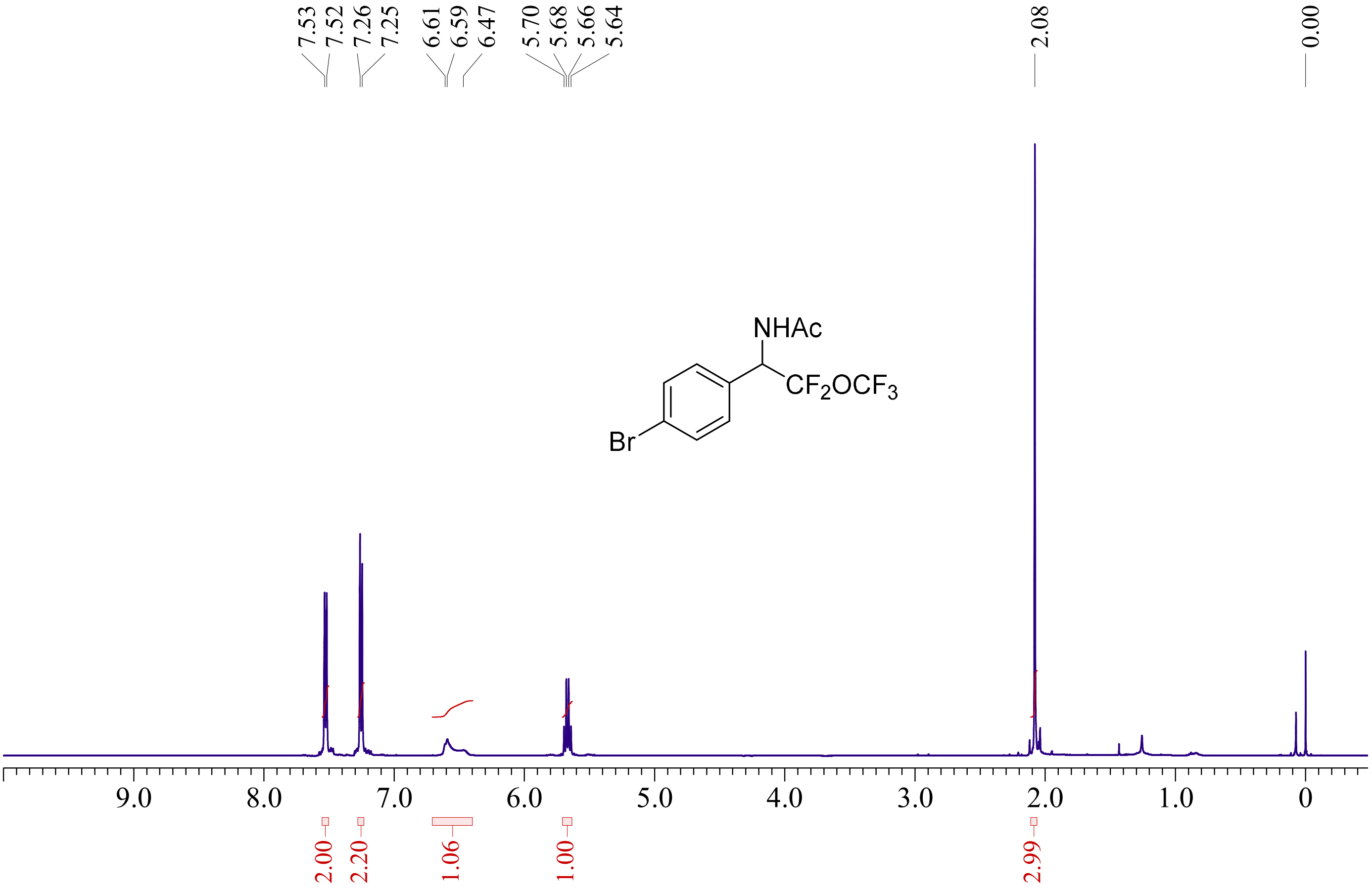


**$^{13}$C NMR** (126 MHz, $CDCl_3$) : **3c**

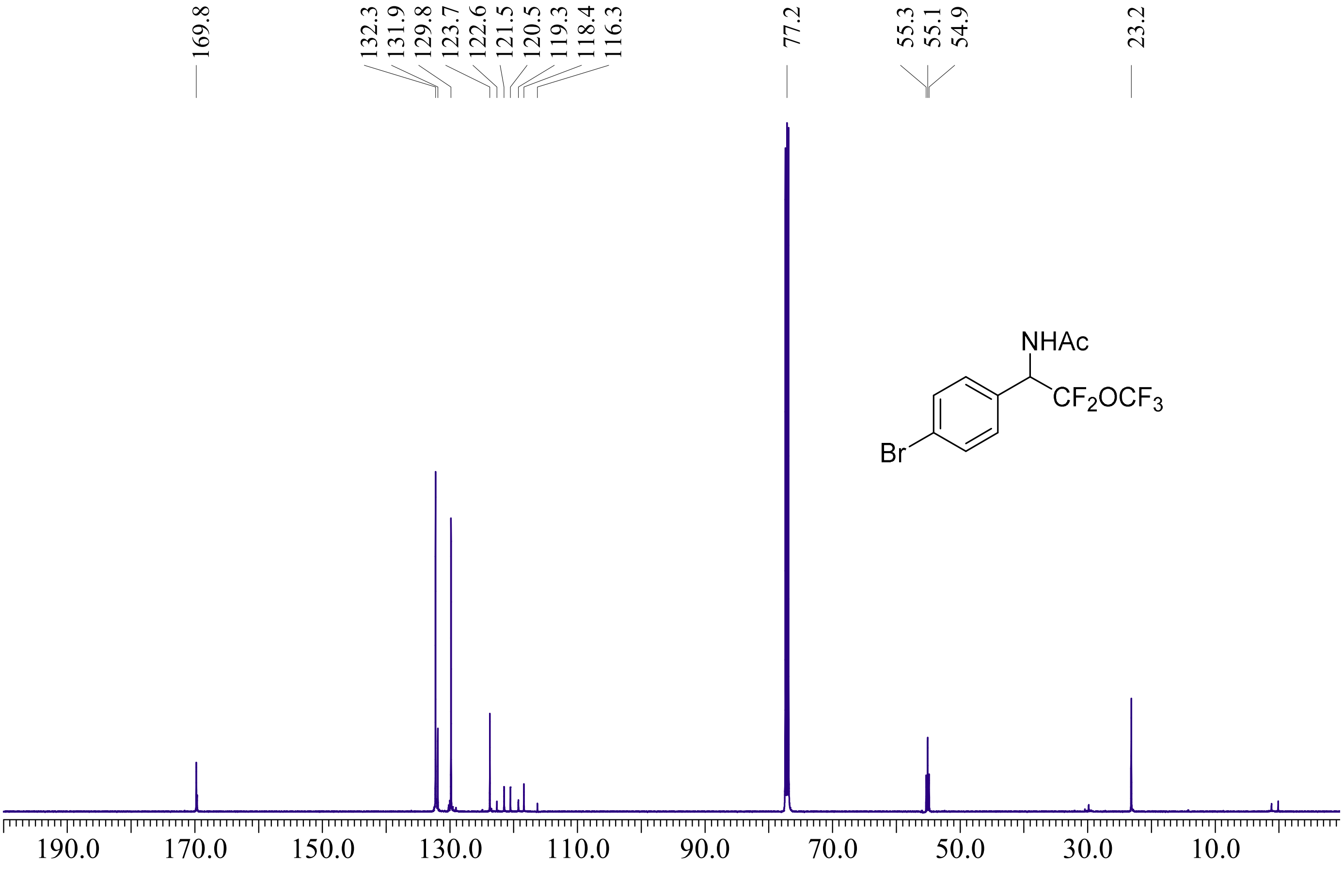

**$^{19}$F NMR** (282 MHz, CDCl3) : **3c**

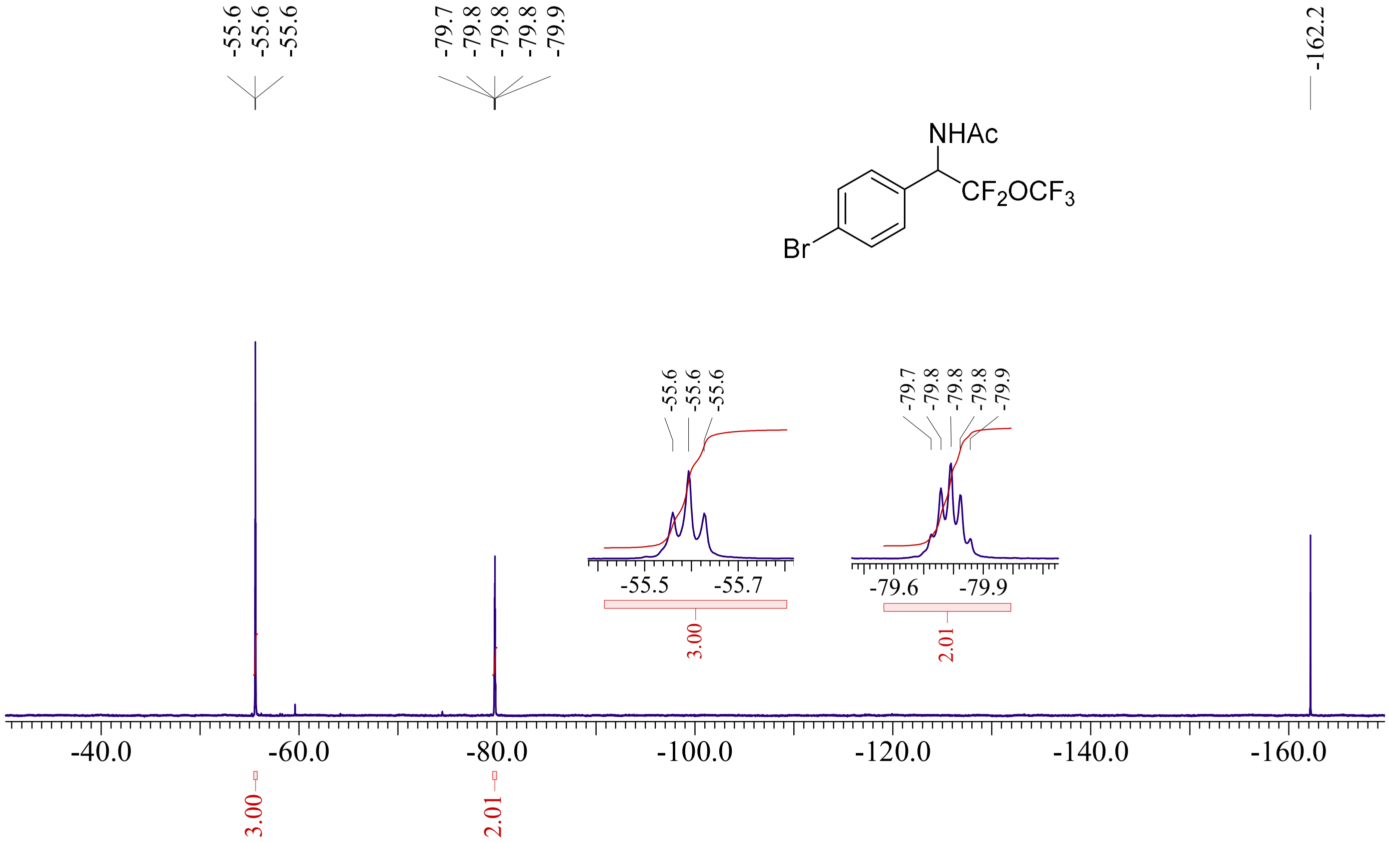


**$^{1}$H NMR** (500 MHz, CDCl3) : **3d**

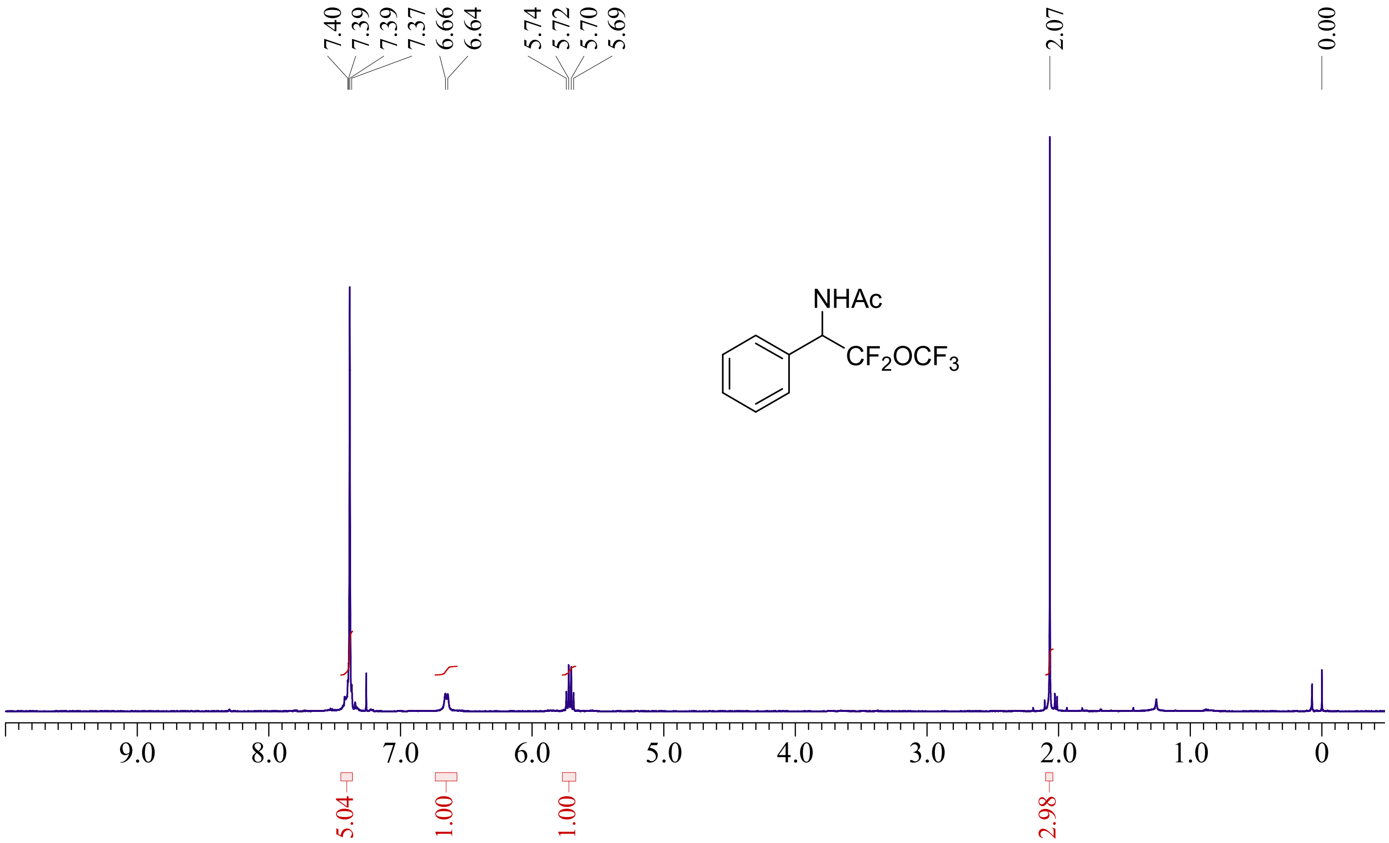

$^{13}$C NMR (126 MHz, $CDCl_3$) : **3d**

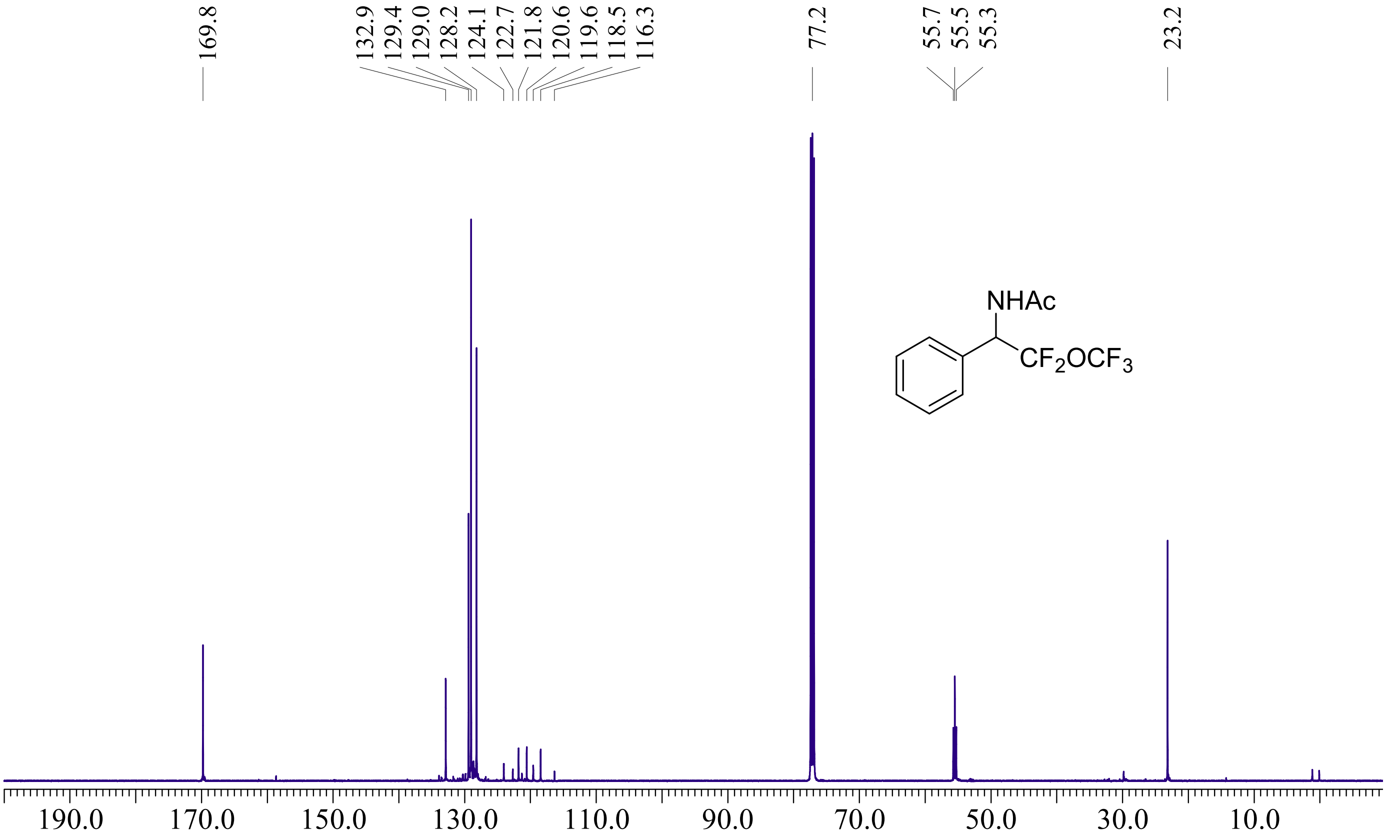


$^{19}$F NMR (282 MHz, $CDCl_3$) : **3d**

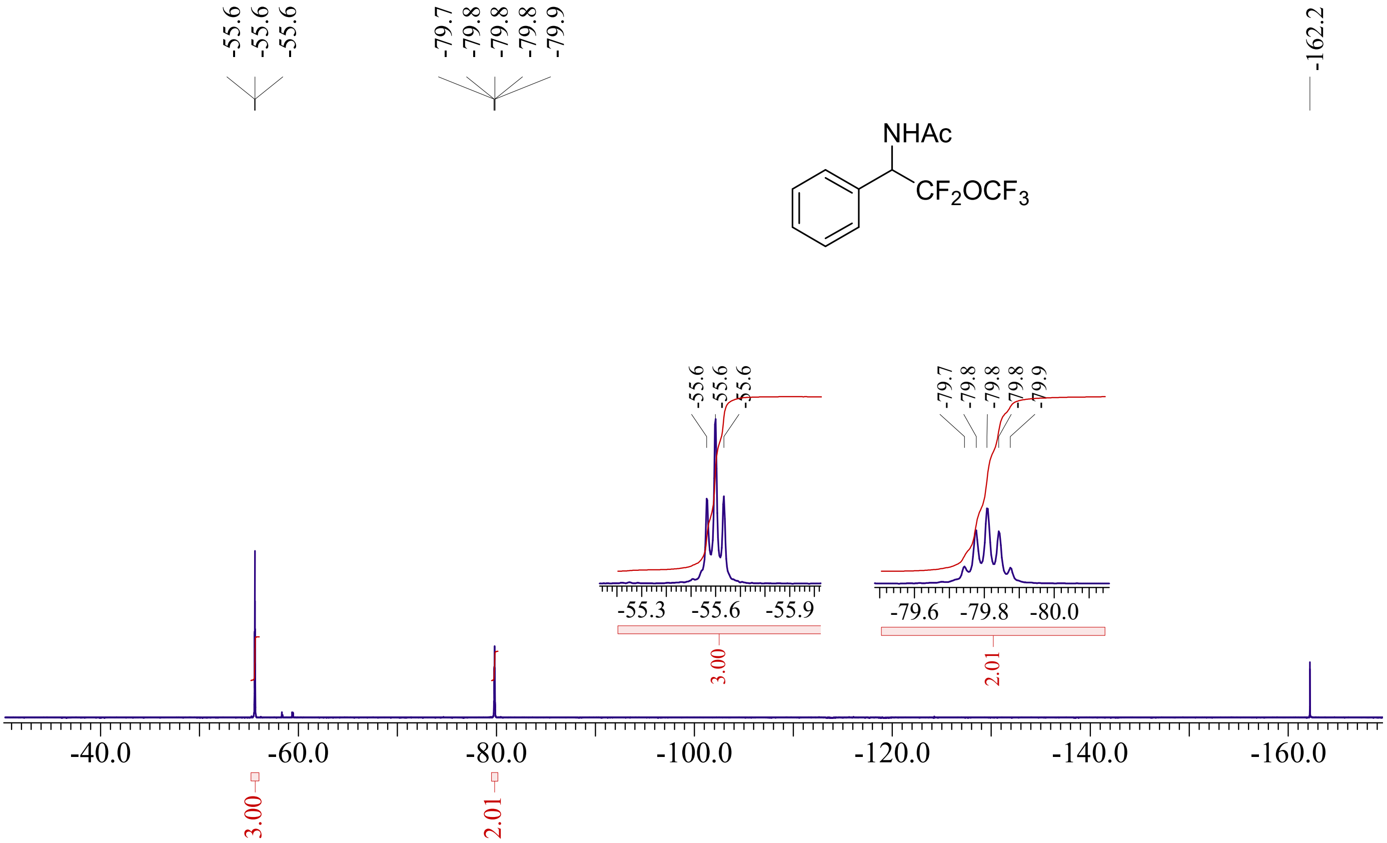

**$^{1}$H NMR** (500 MHz, $CDCl_3$) : **3e**

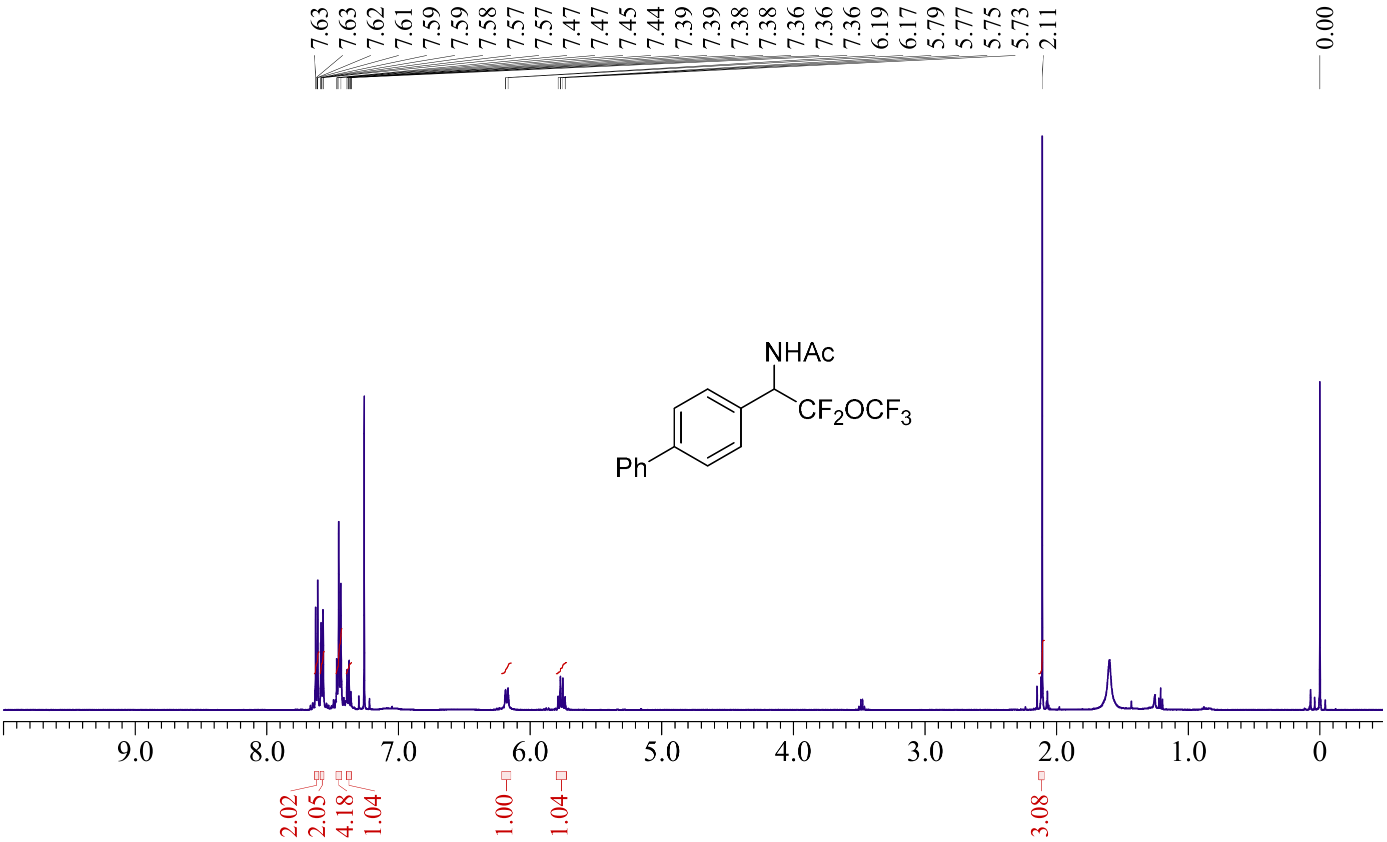


**$^{13}$C NMR** (126 MHz, $CDCl_3$) : **3e**

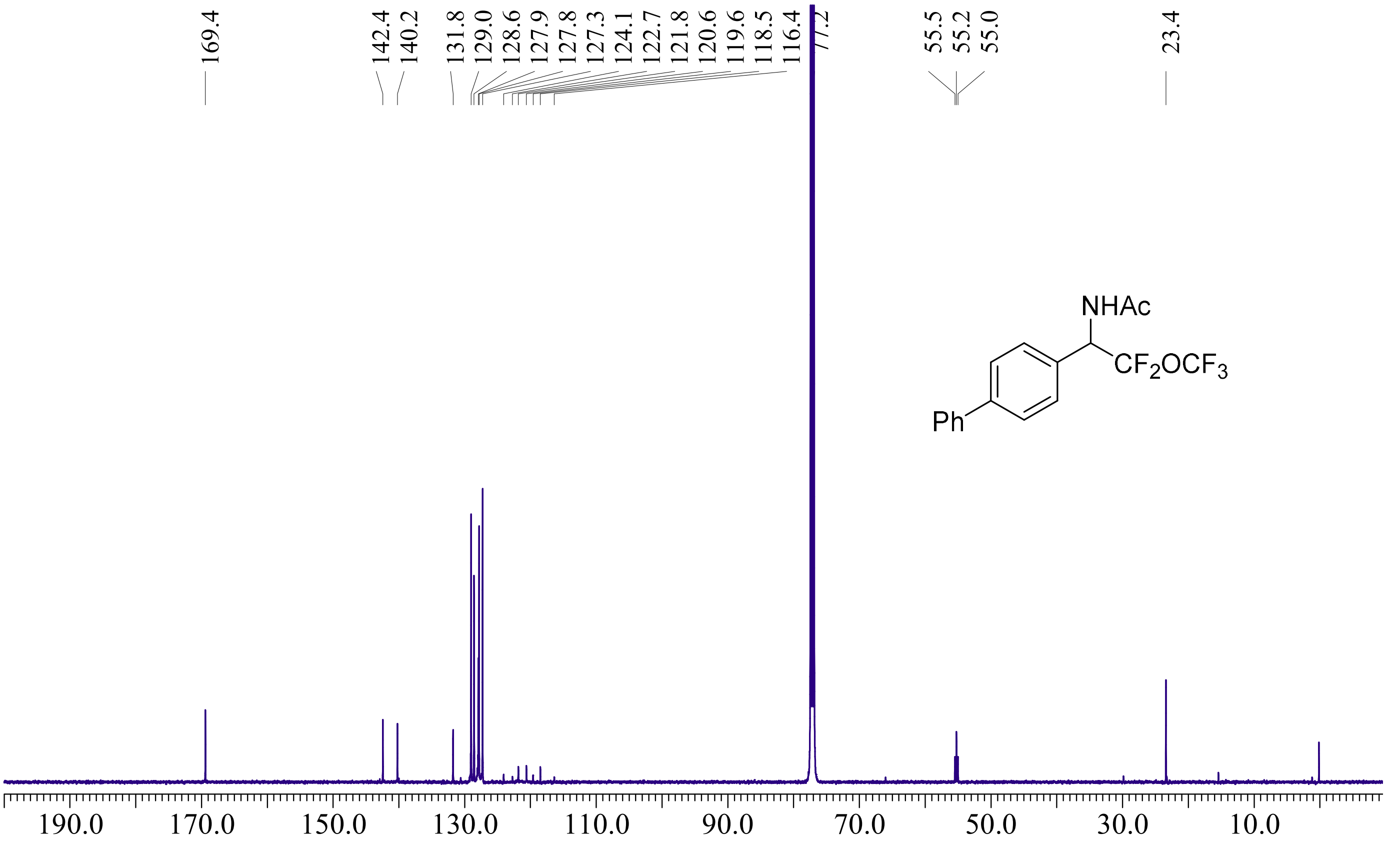

**$^{19}$F NMR** (282 MHz, $CDCl_3$) : **3e**

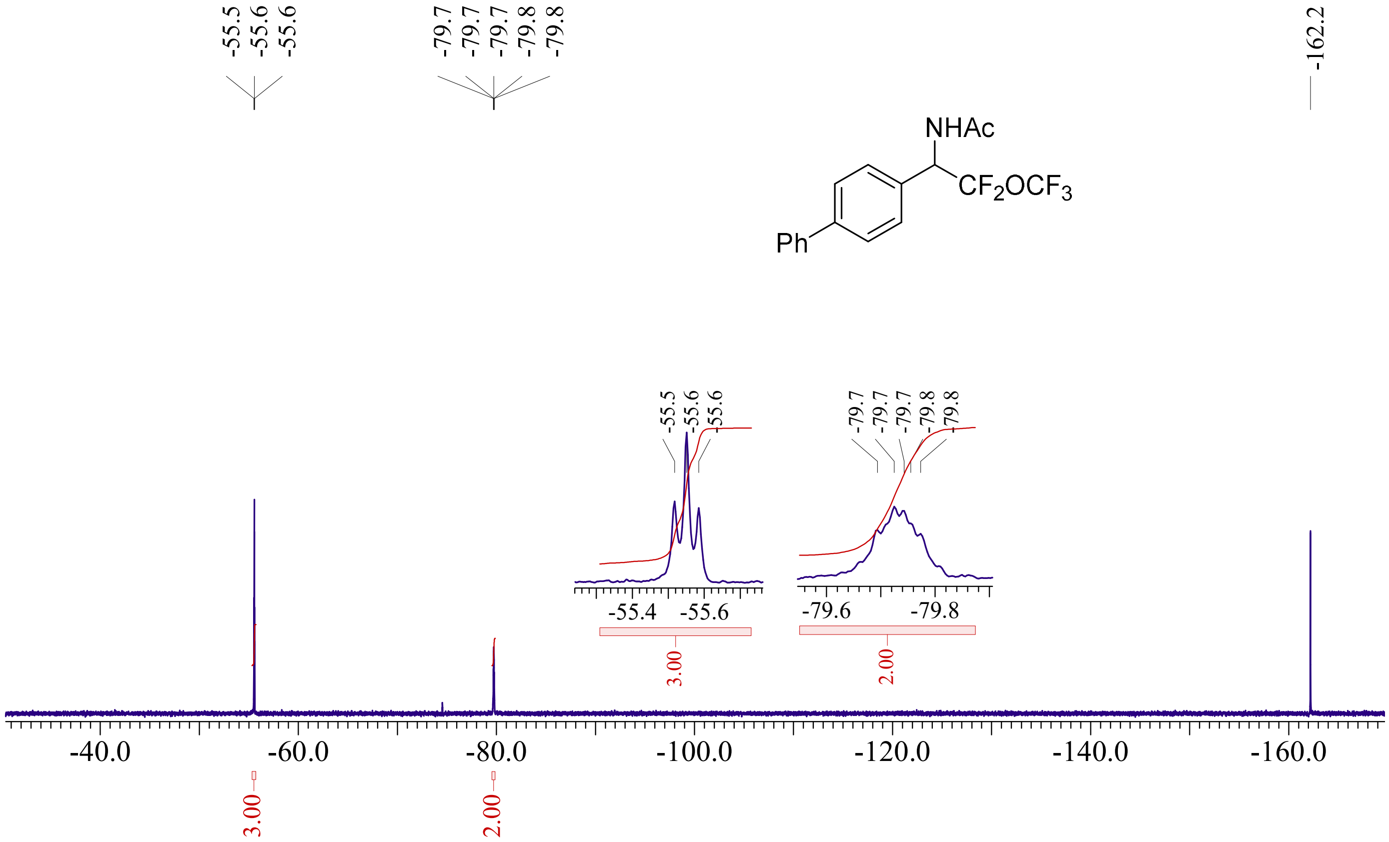


**$^{1}$H NMR** (500 MHz, $CDCl_3$) : **3f**

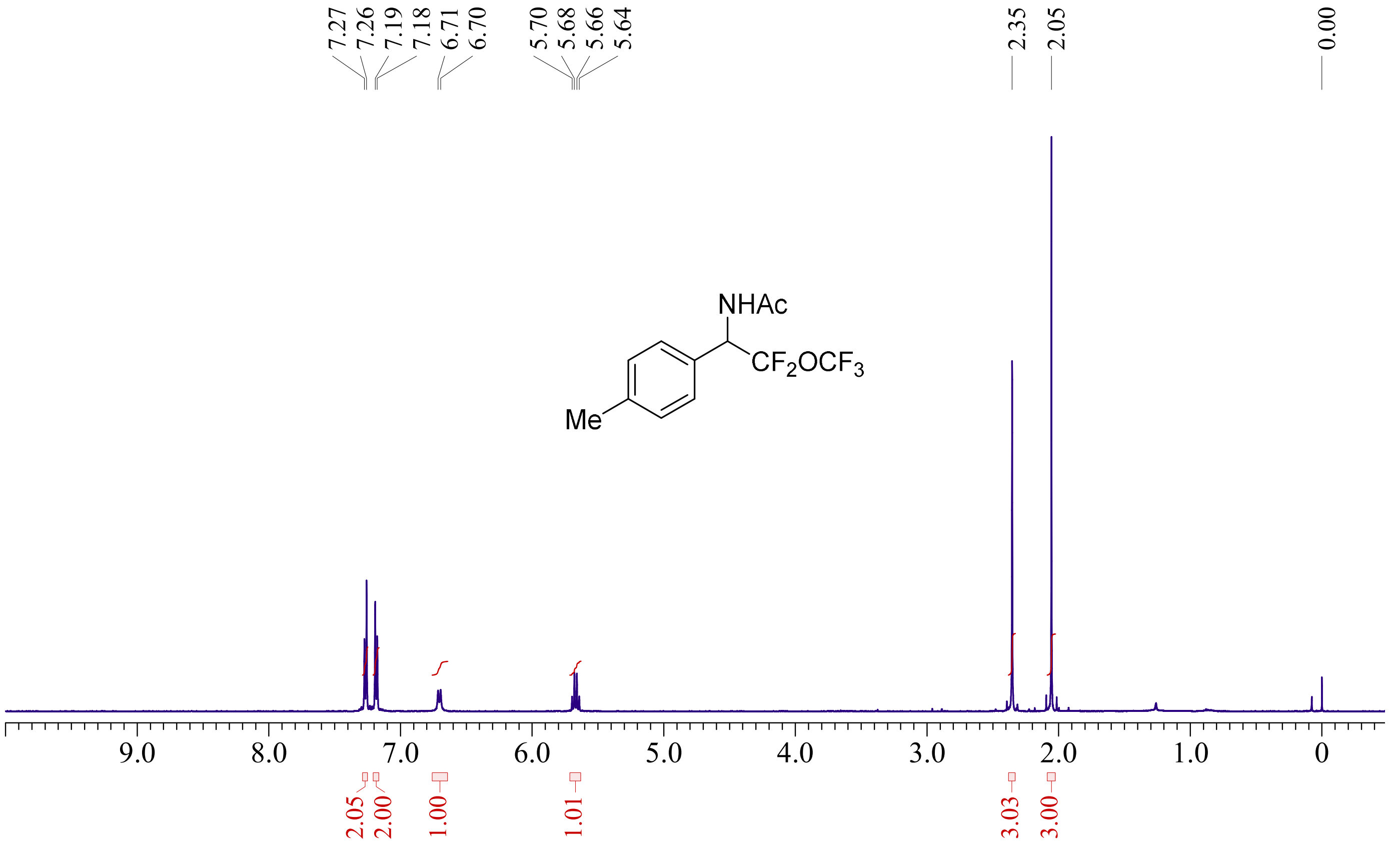

**$^{13}$C NMR** (126 MHz, $CDCl_3$) : **3f**

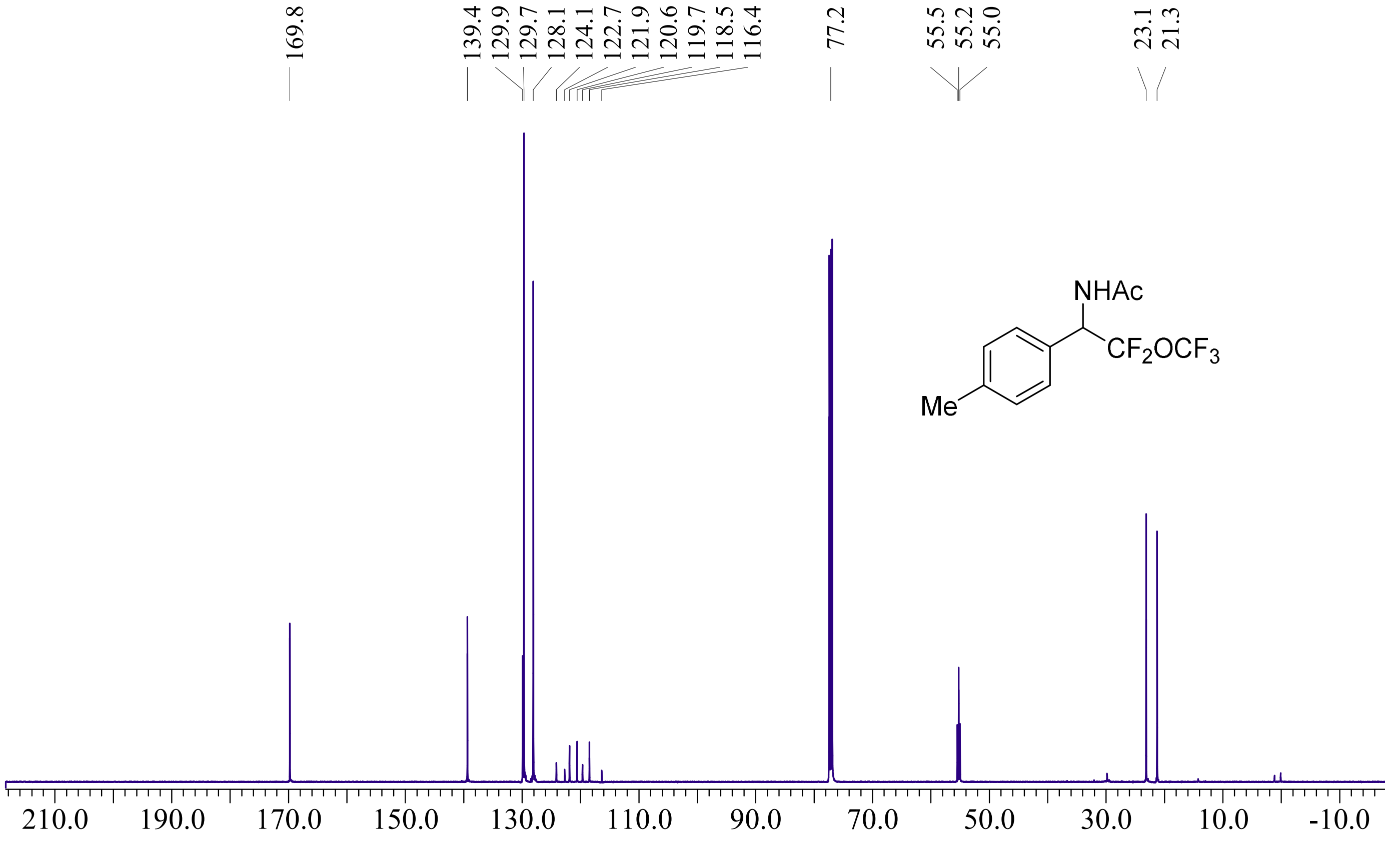


**$^{19}$F NMR** (282 MHz, $CDCl_3$) : **3f**

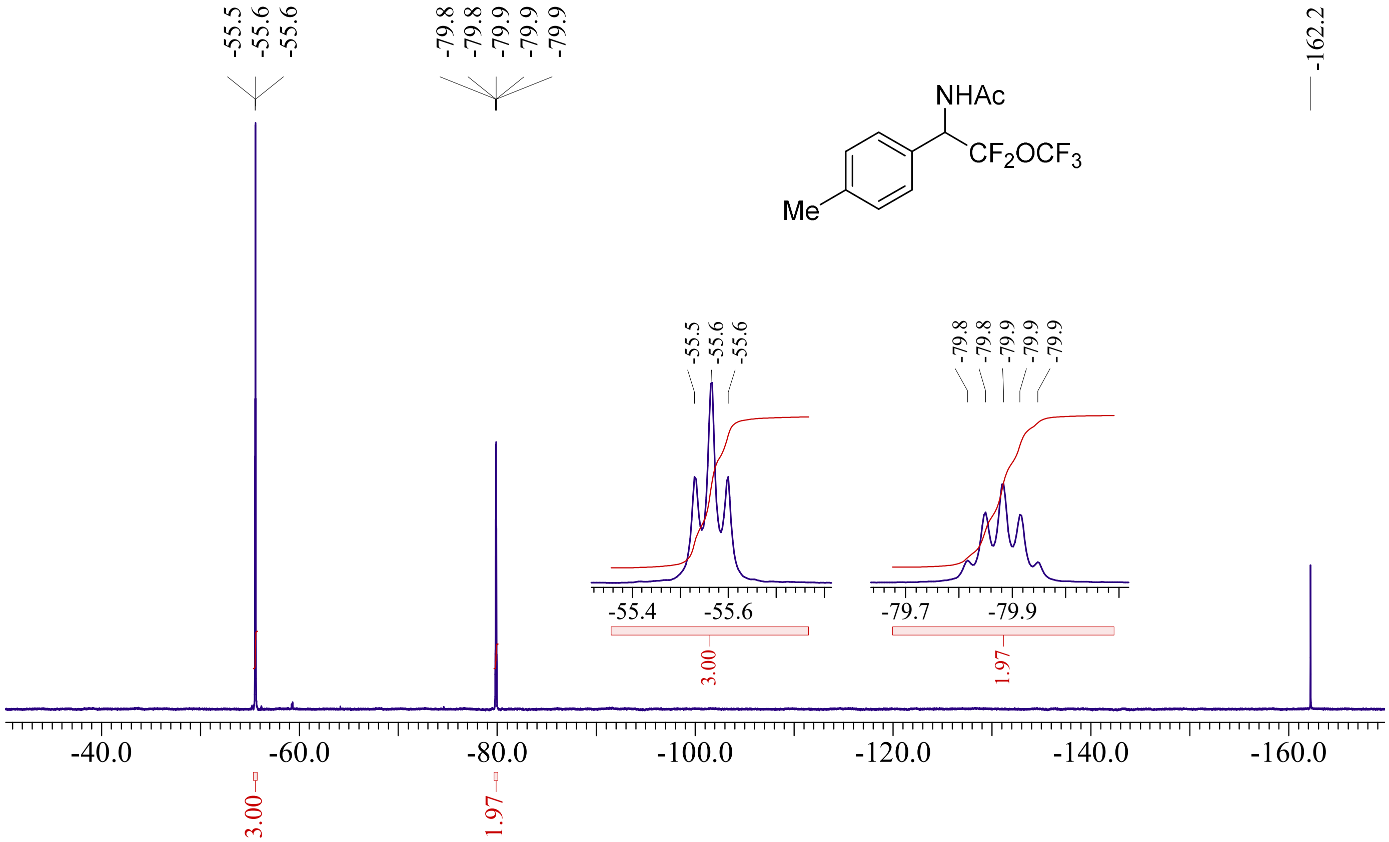

**¹H NMR** (500 MHz, $CDCl_3$) : **3g**

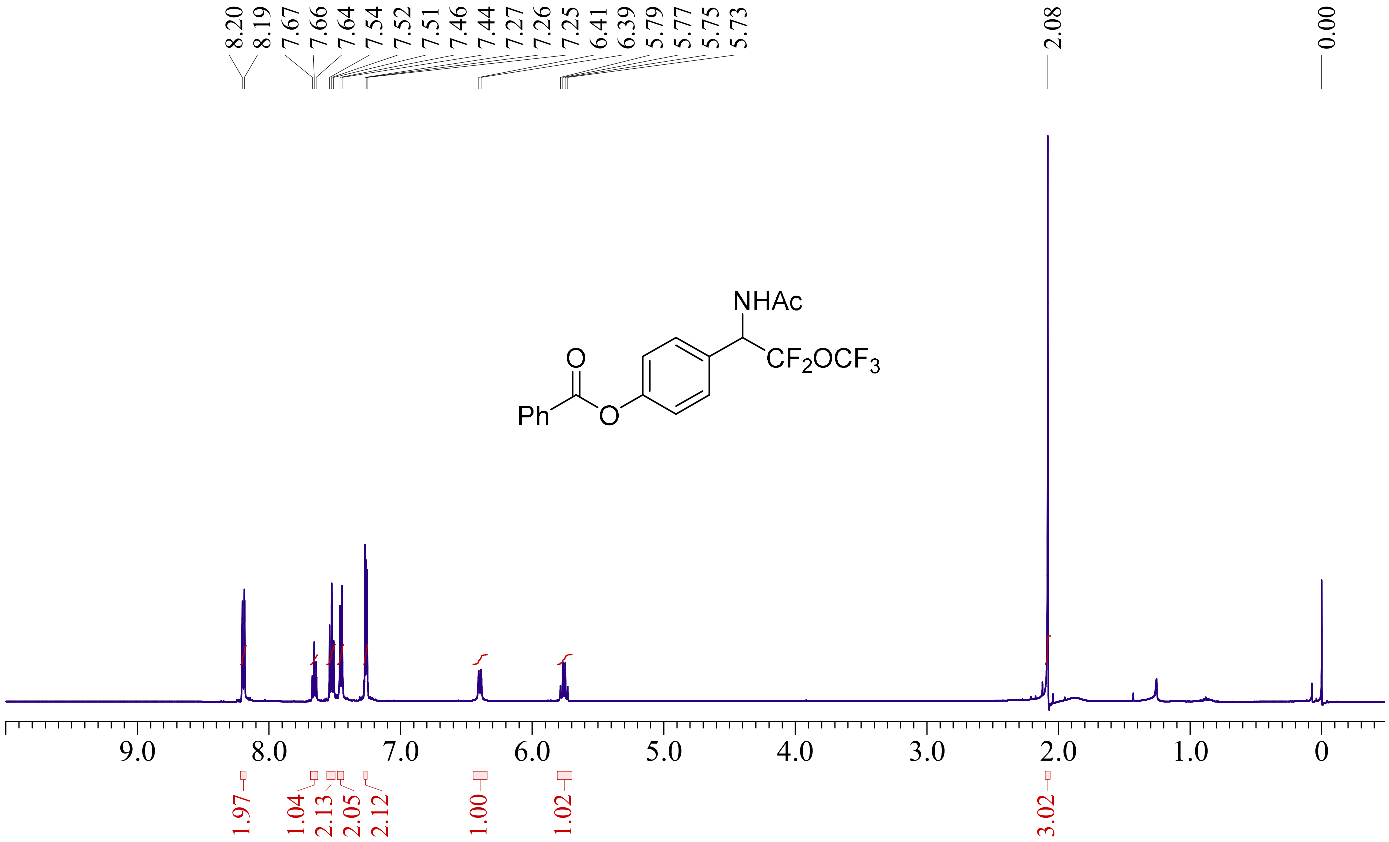


**¹³C NMR** (126 MHz, $CDCl_3$) : **3g**

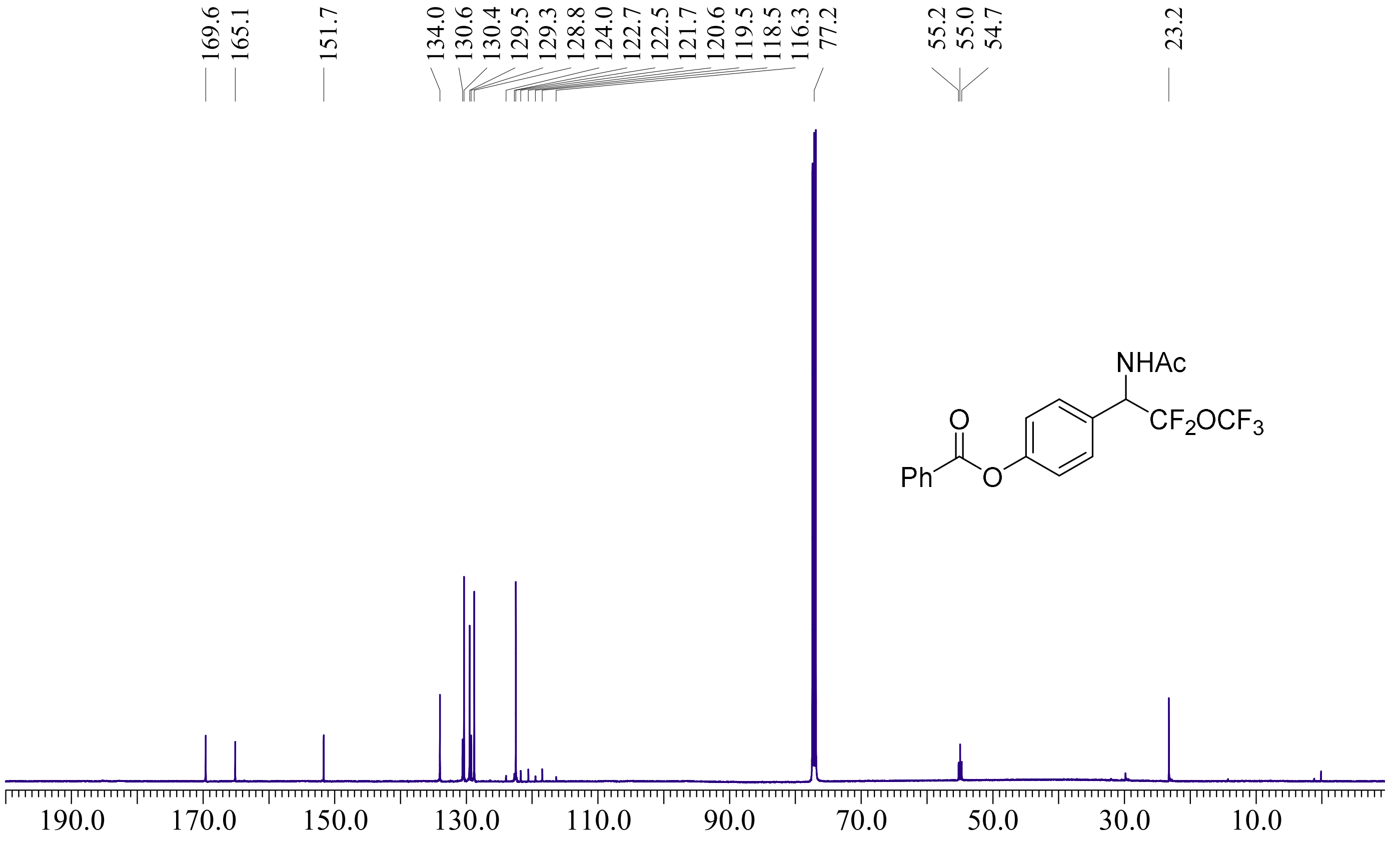

[19]**F NMR** (282 MHz, CDCl$_3$) : **3g**

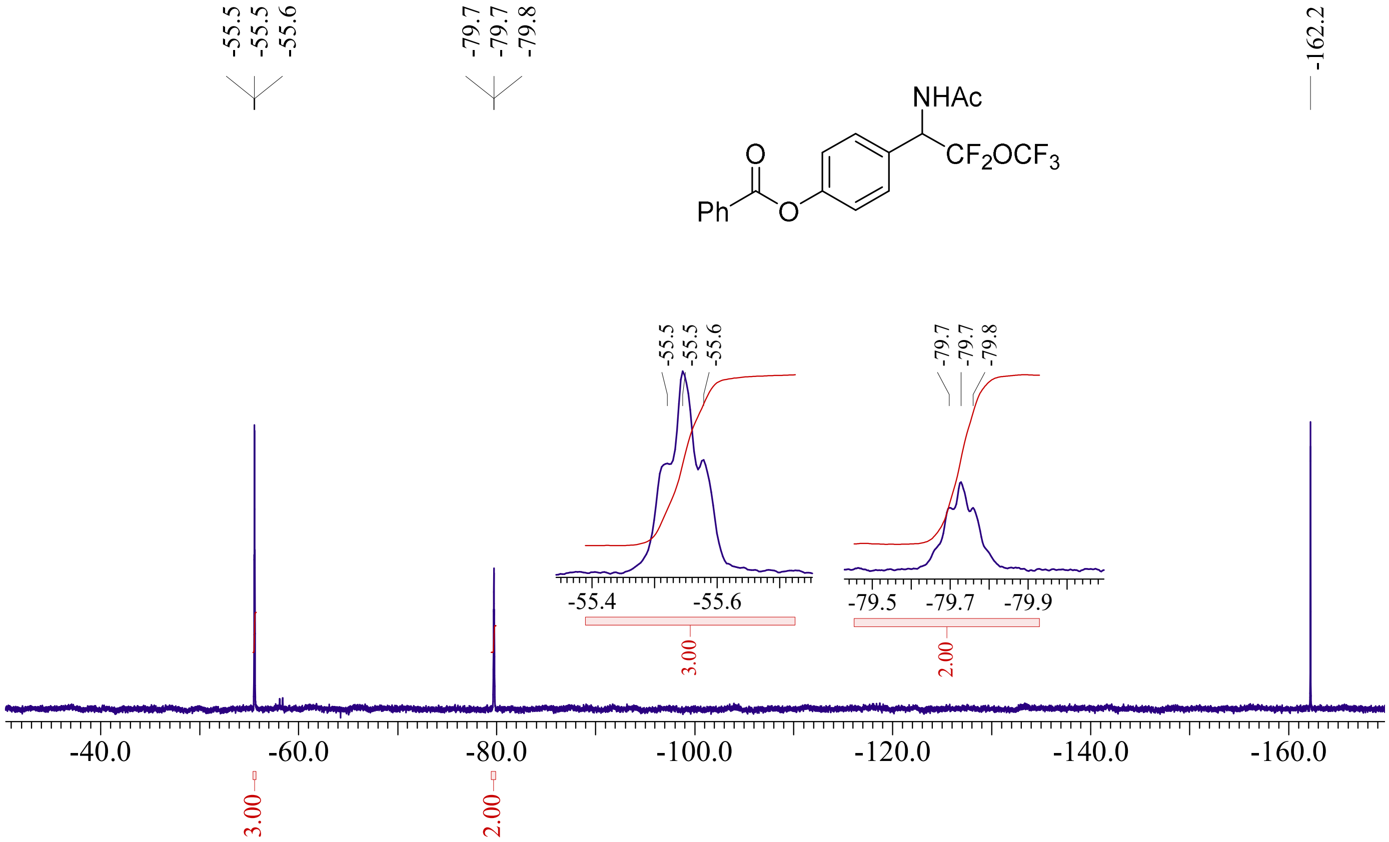

[1]**H NMR** (500 MHz, CDCl$_3$) : **3h**

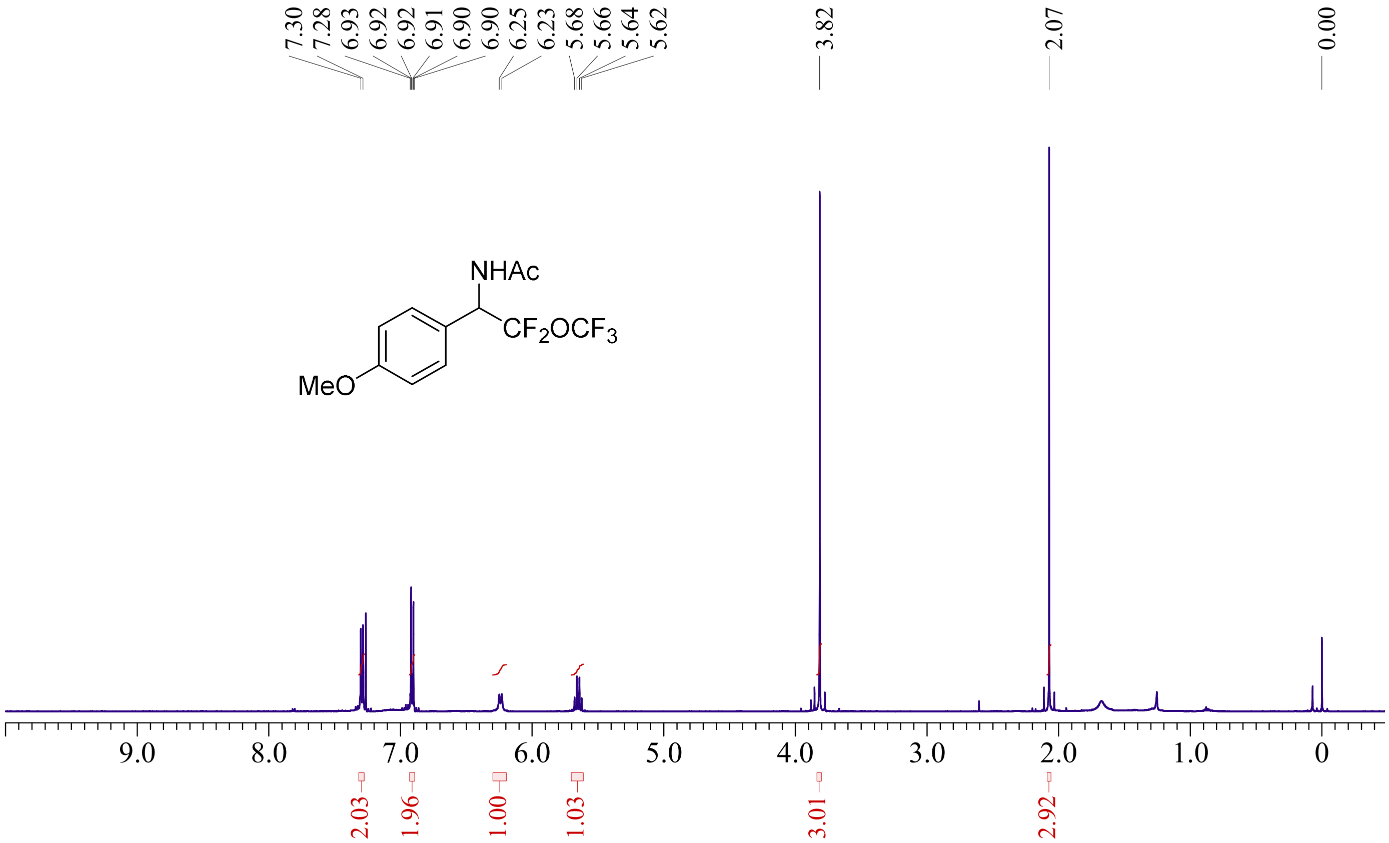

**$^{13}$C NMR** (126 MHz, $CDCl_3$) : **3h**

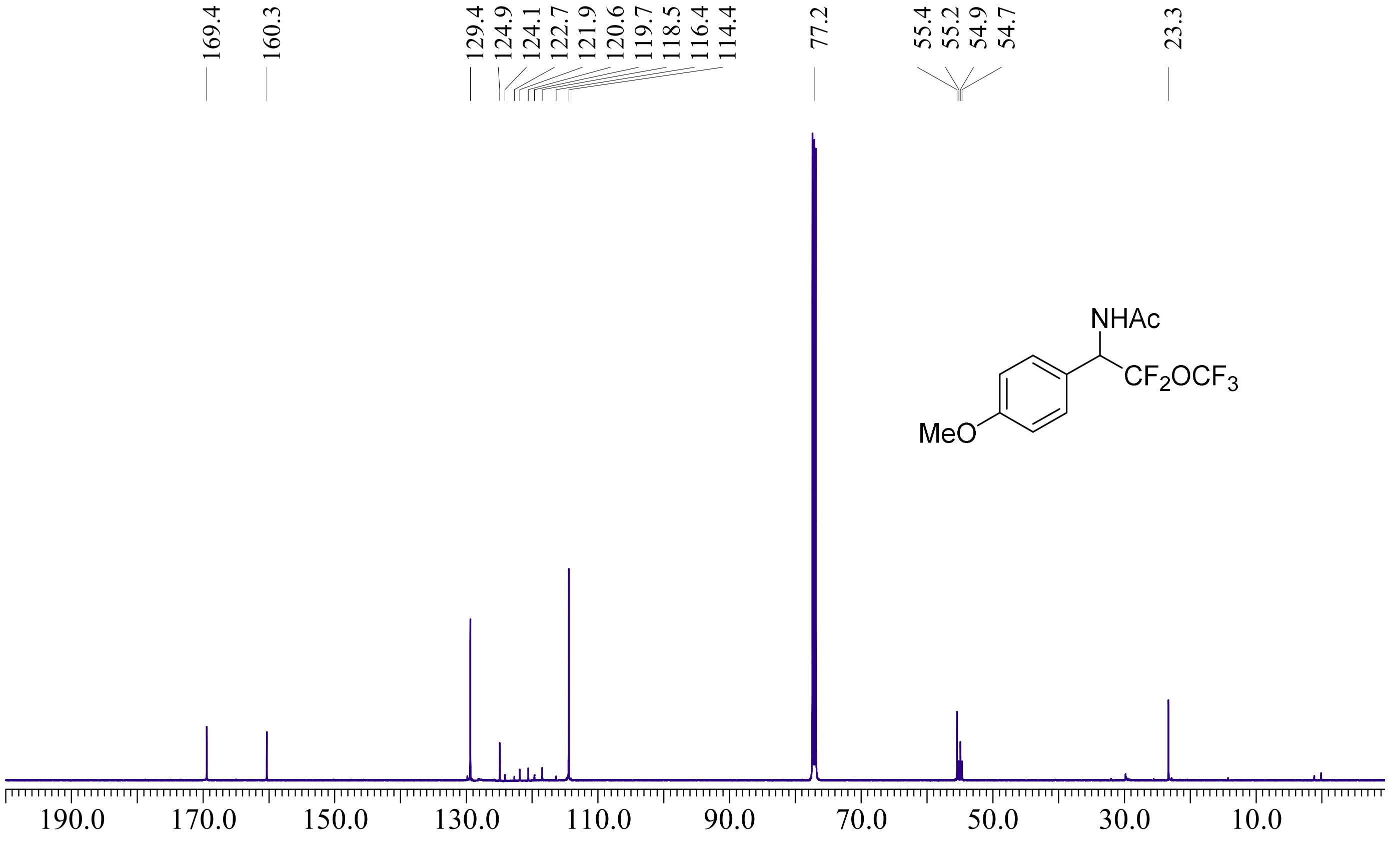


**$^{19}$F NMR** (282 MHz, $CDCl_3$) : **3h**

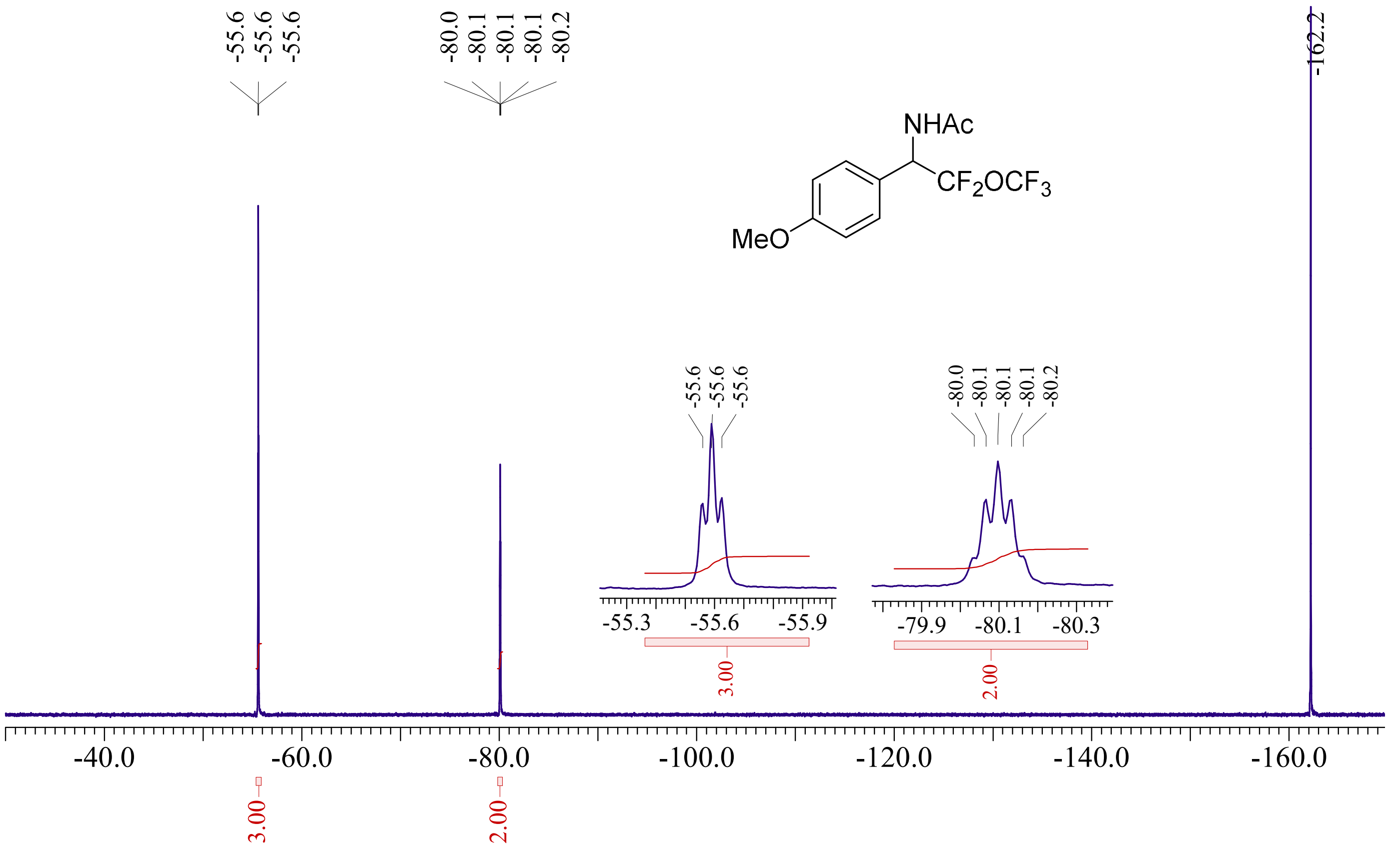

**$^{1}$H NMR** (500 MHz, $CDCl_3$) : **3i**

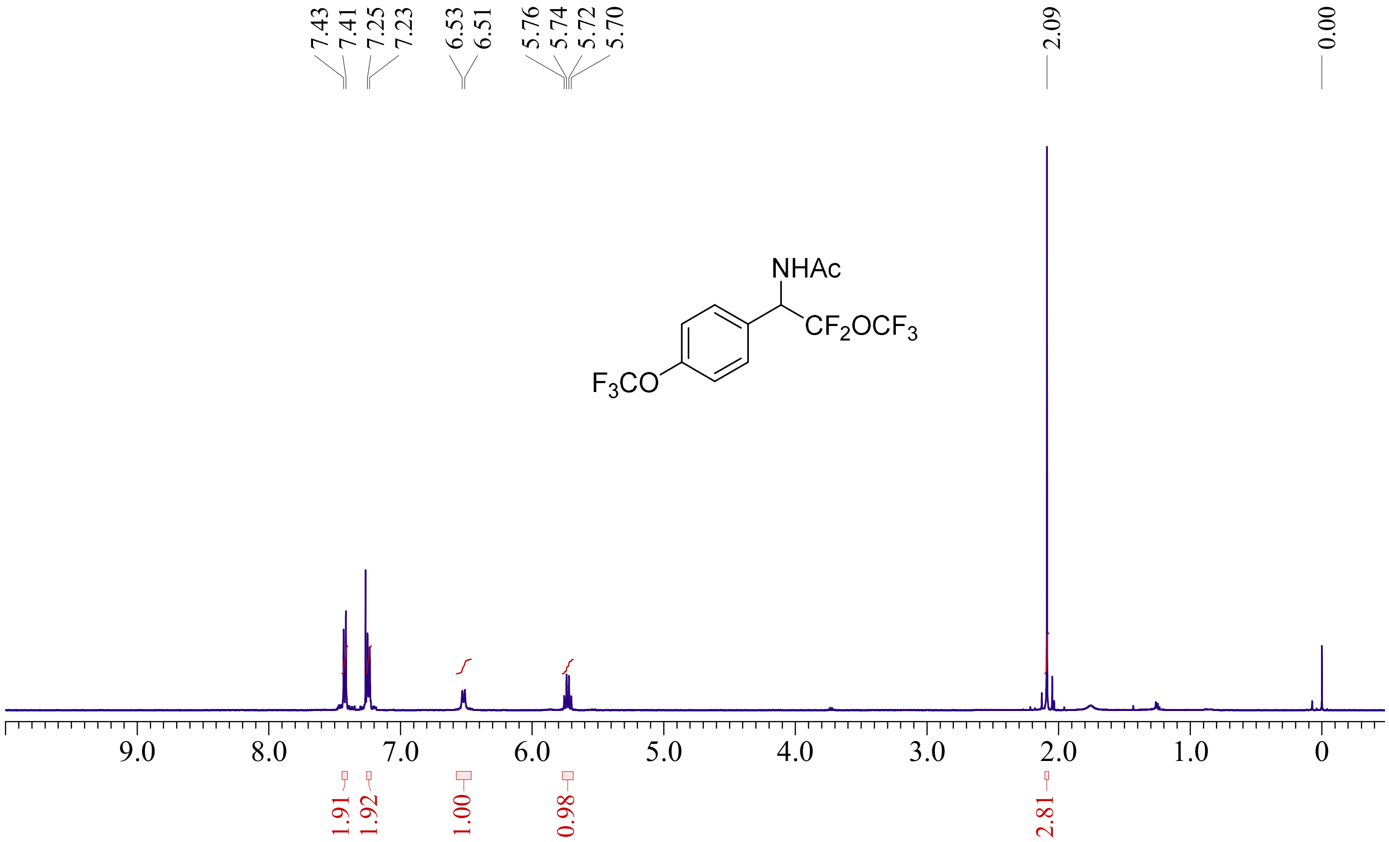


**$^{13}$C NMR** (126 MHz, $CDCl_3$) : **3i**

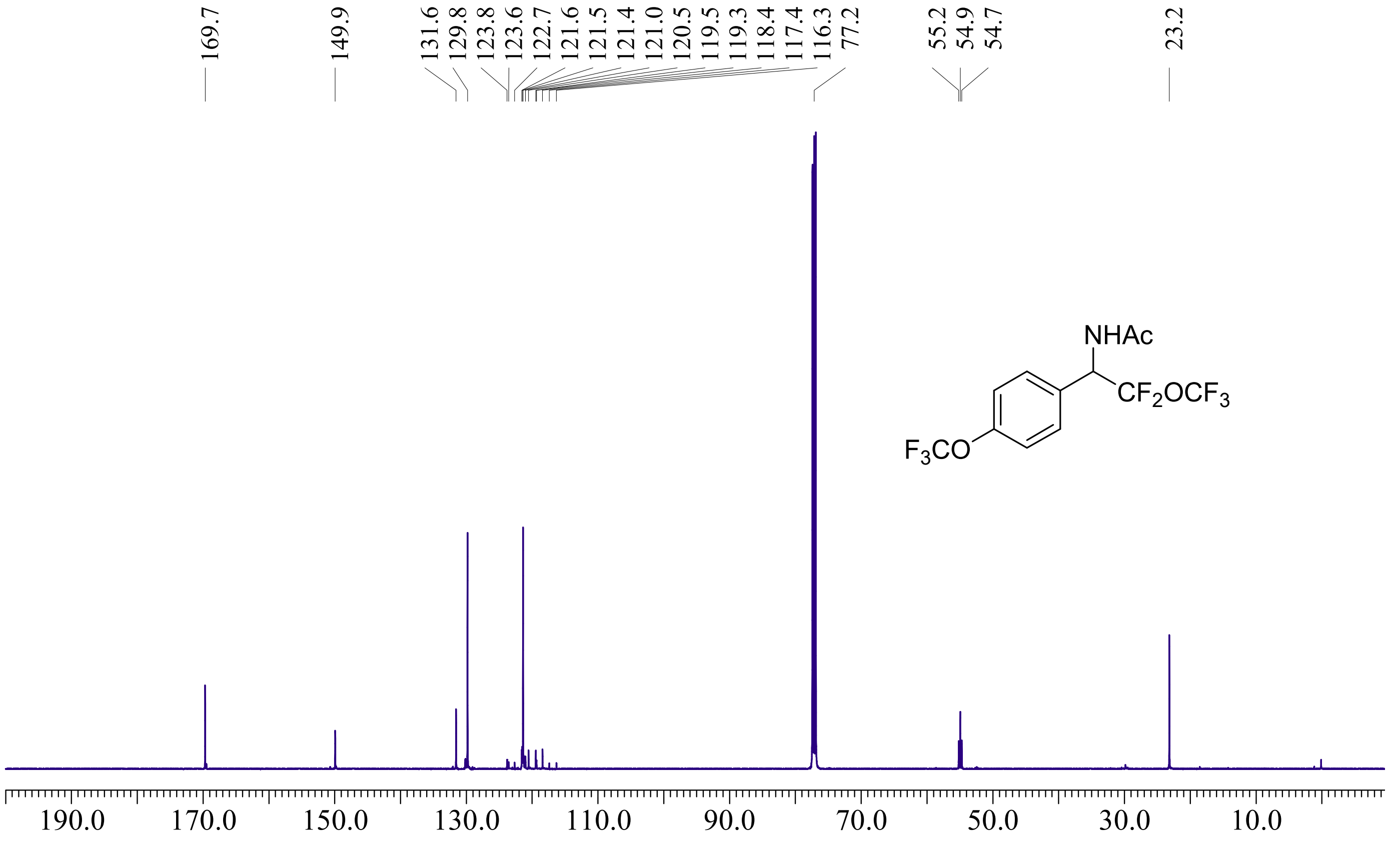

**$^{19}$F NMR** (282 MHz, $CDCl_3$) : **3i**

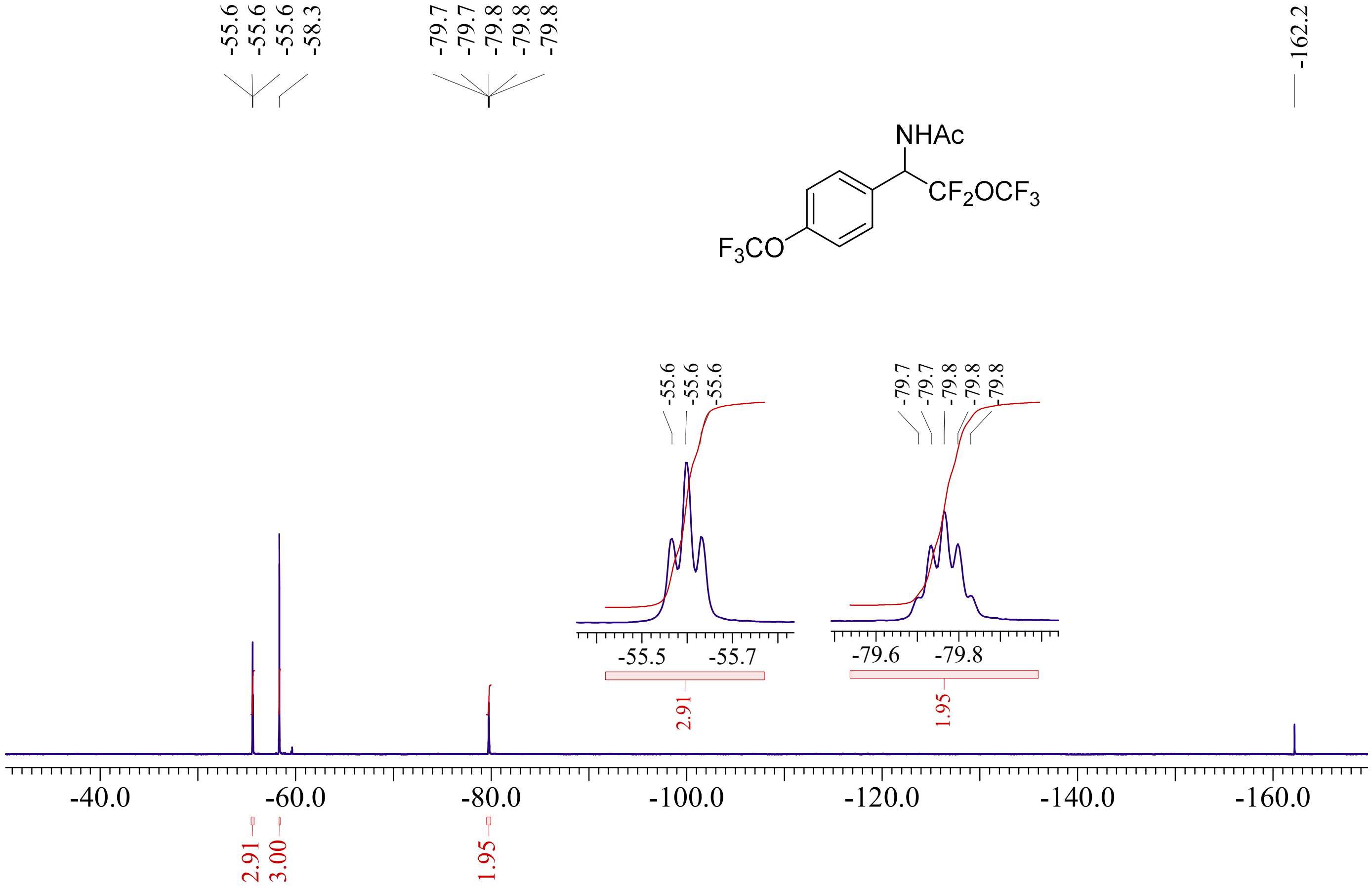


**$^{1}$H NMR** (500 MHz, $CDCl_3$) : **3j**

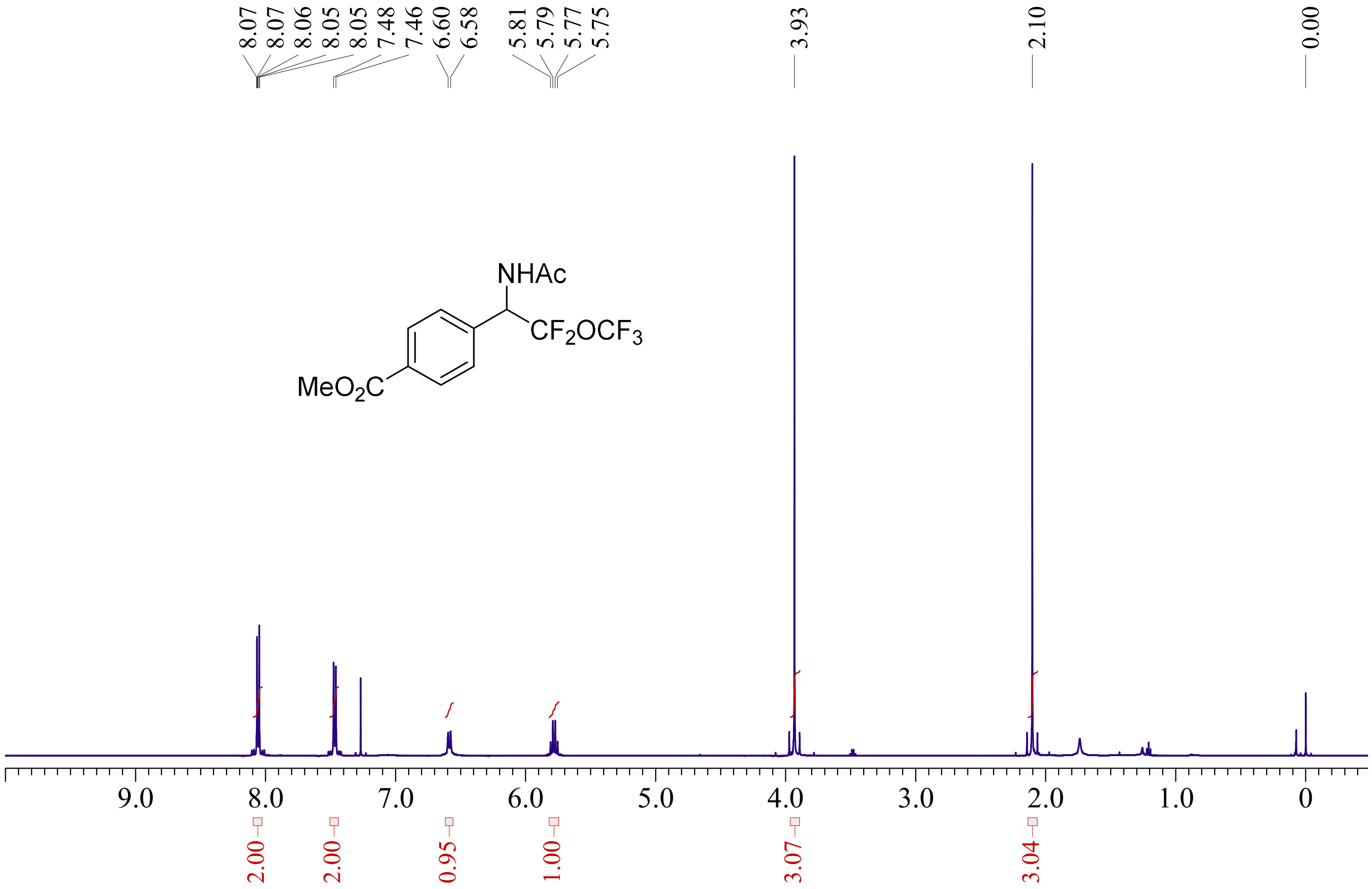

**$^{13}$C NMR** (126 MHz, $CDCl_3$) : **3j**

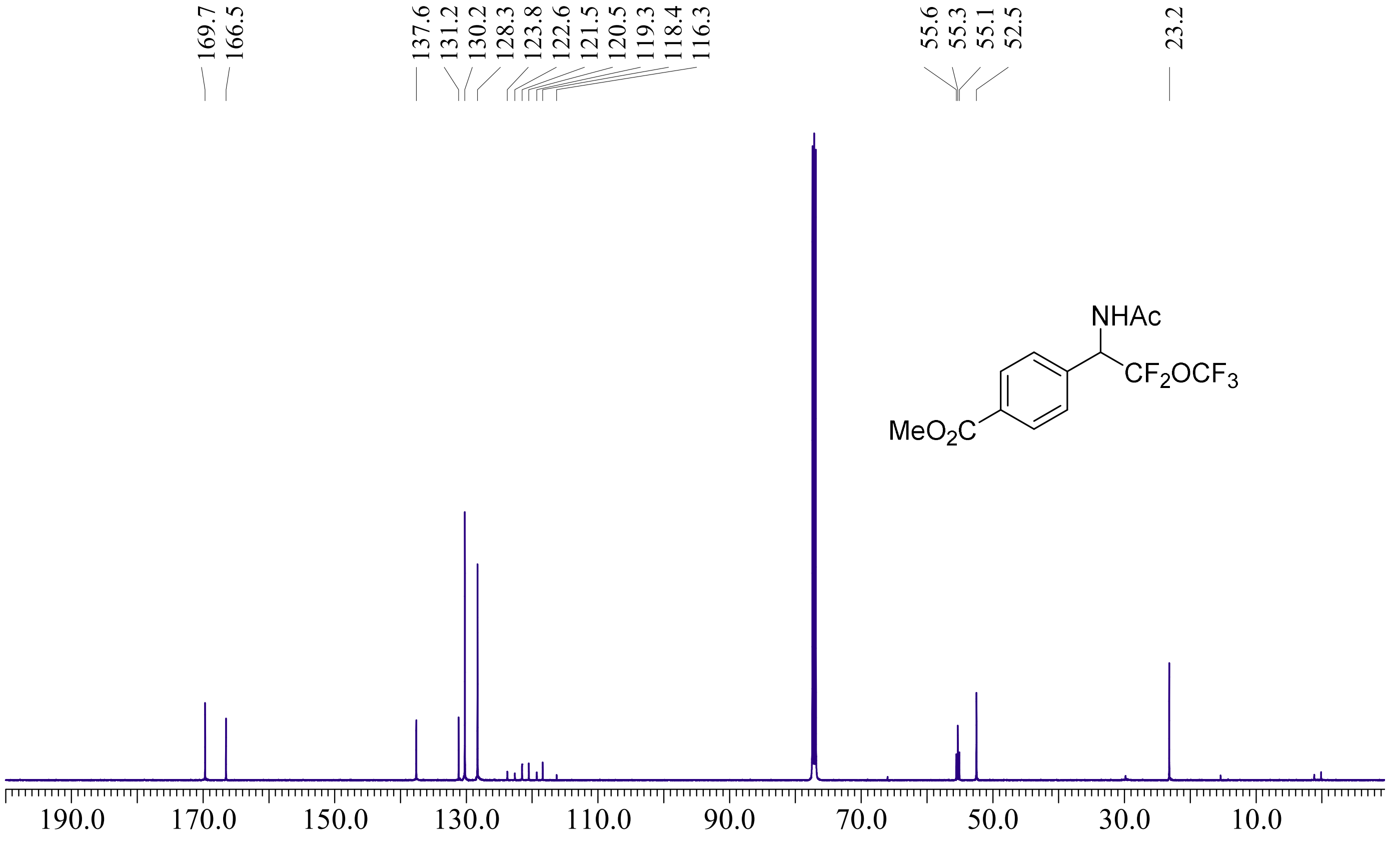


**$^{19}$F NMR** (282 MHz, $CDCl_3$) : **3j**

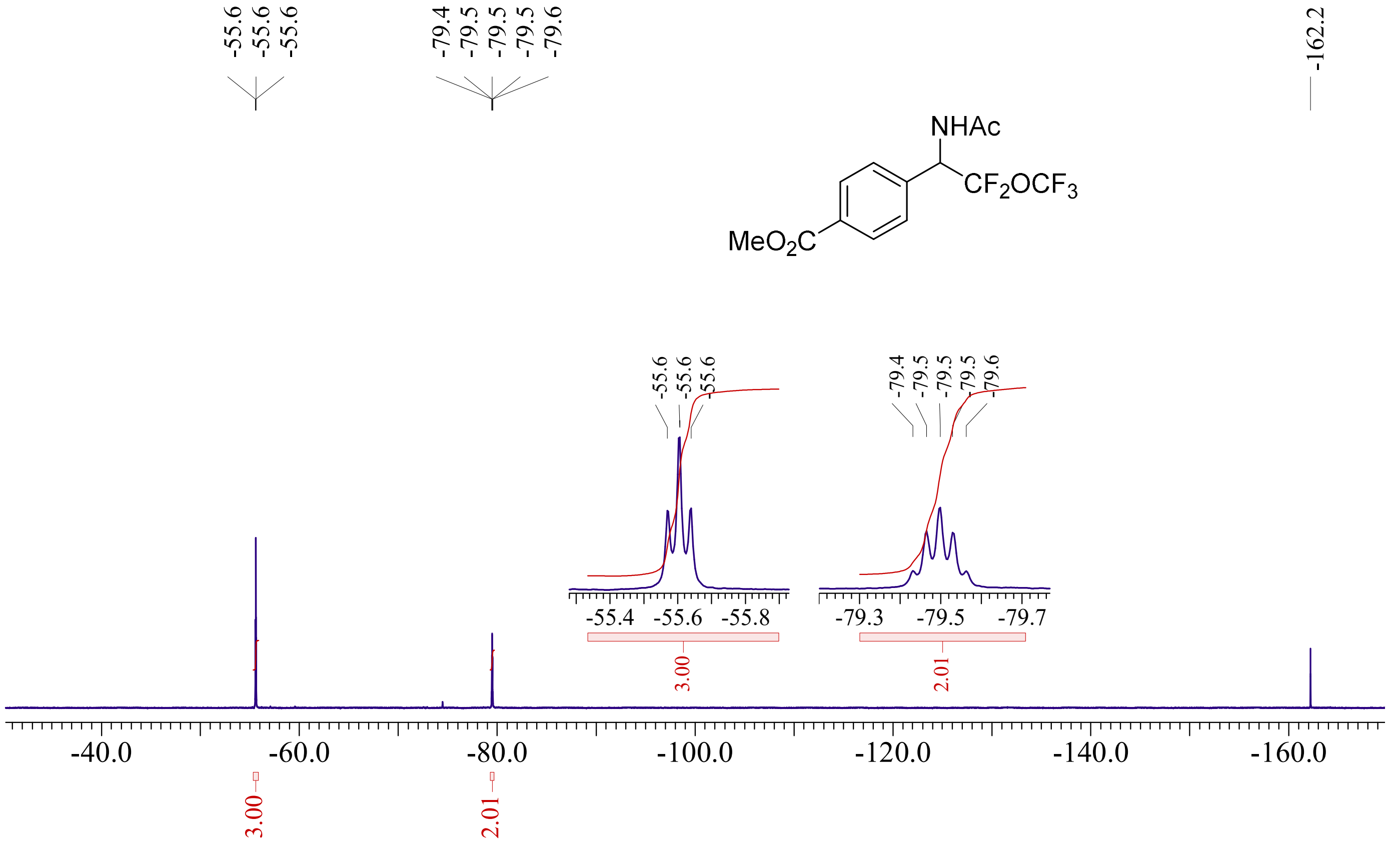

**$^{1}$H NMR** (500 MHz, $CDCl_3$) : **3l**

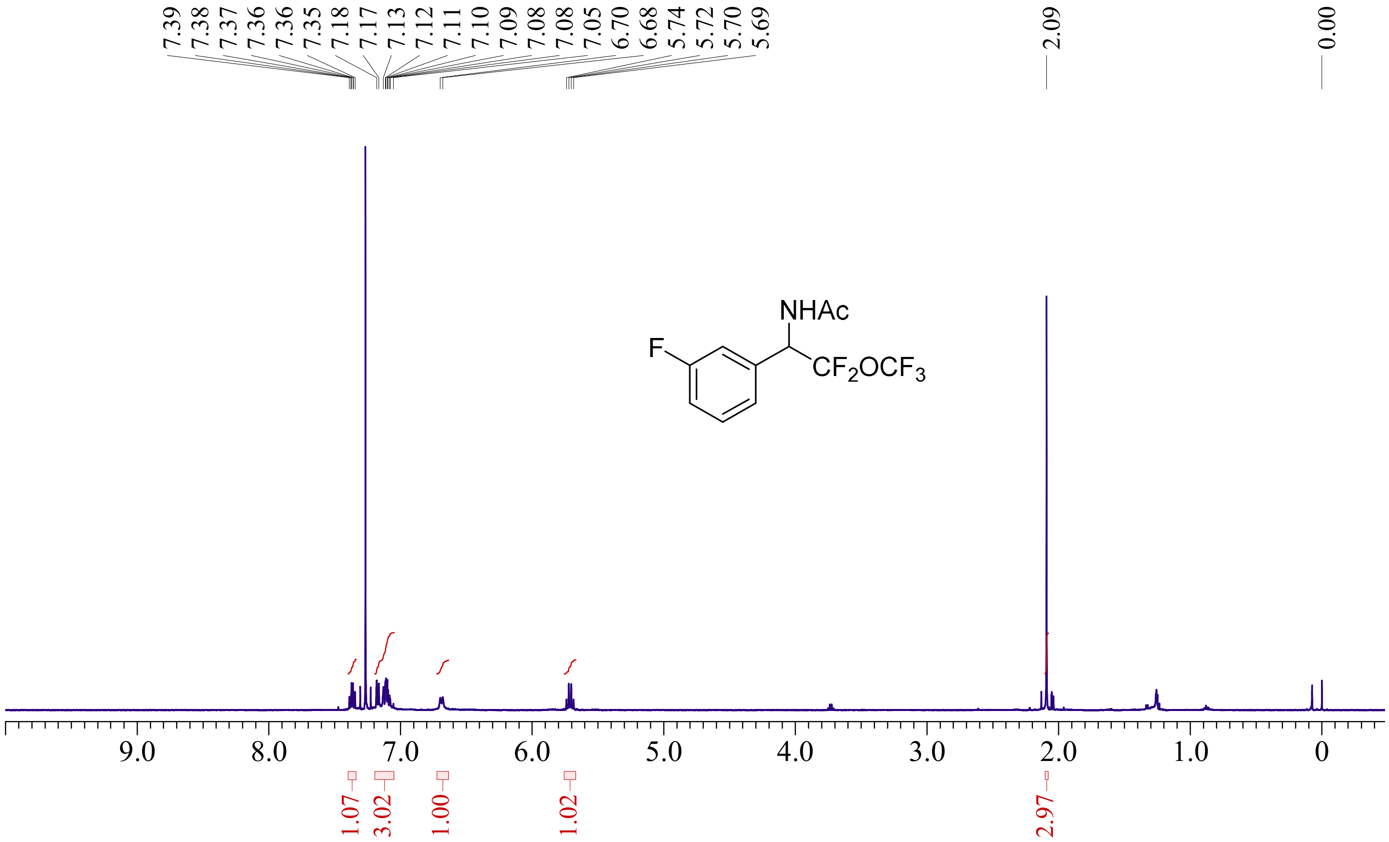


**$^{13}$C NMR** (126 MHz, $CDCl_3$) : **3l**

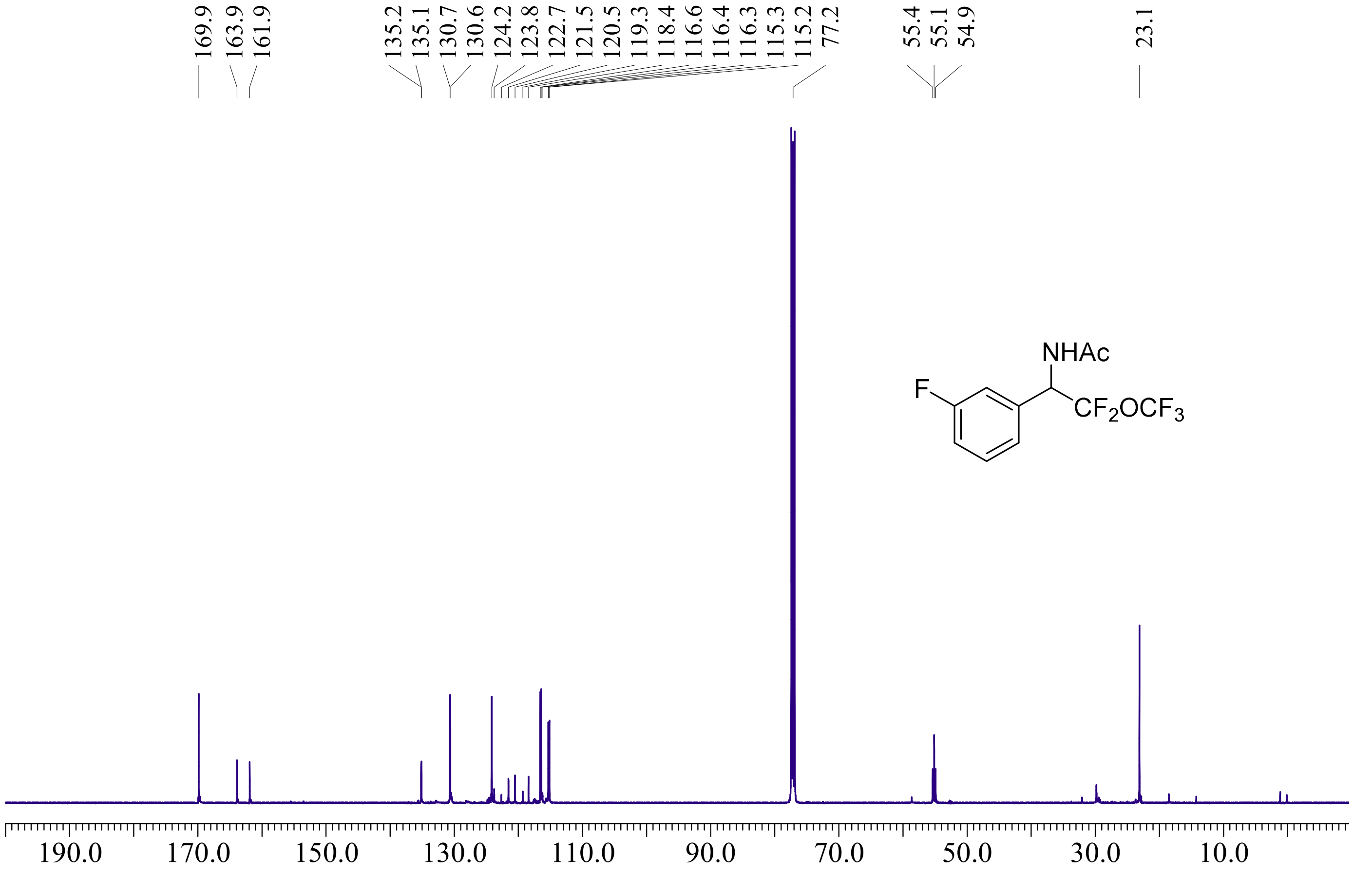

**$^{19}$F NMR** (282 MHz, $CDCl_3$) : **3l**

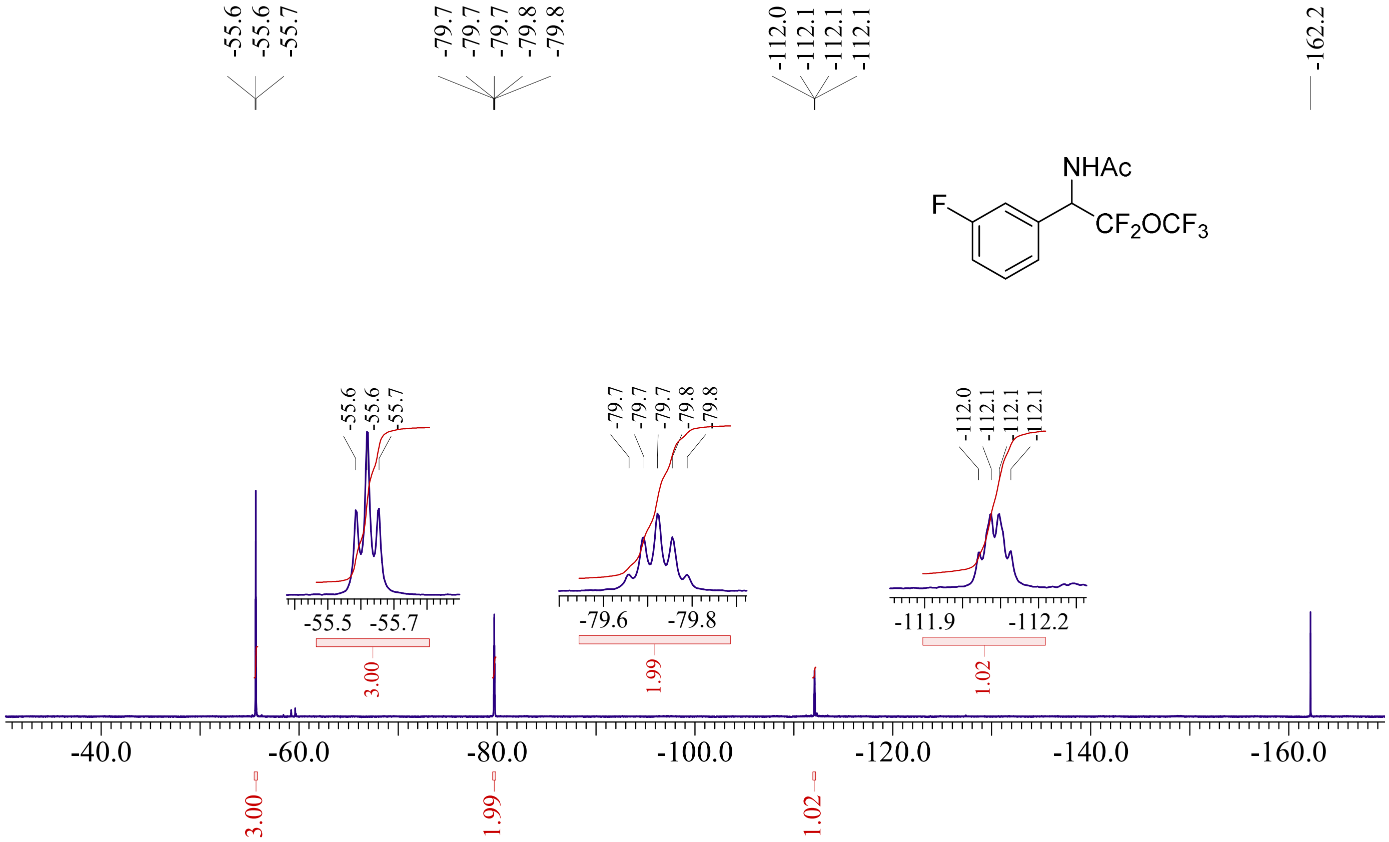


**$^{1}$H NMR** (500 MHz, $CDCl_3$) : **3m**

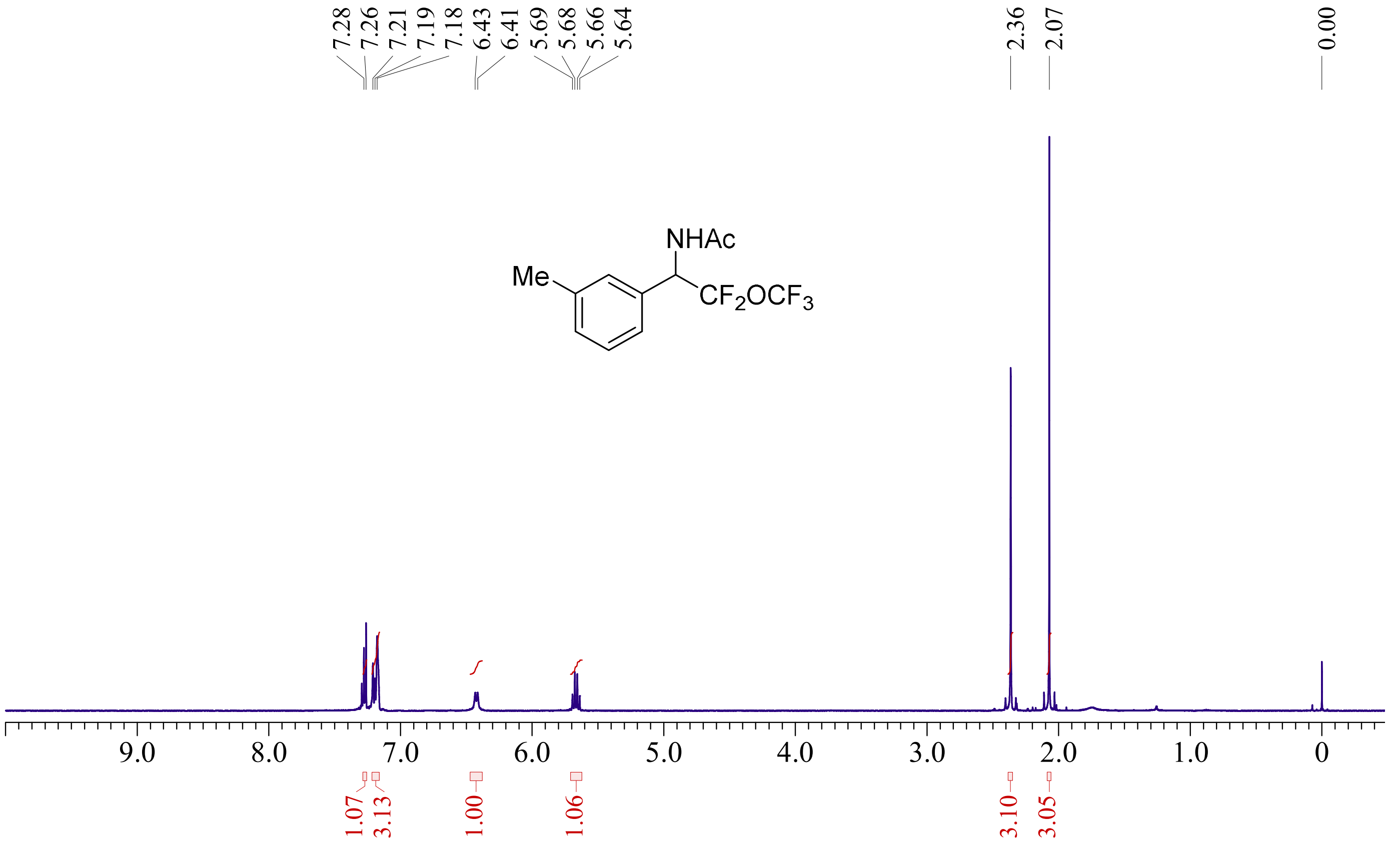

**$^{13}$C NMR** (126 MHz, $CDCl_3$) : **3m**

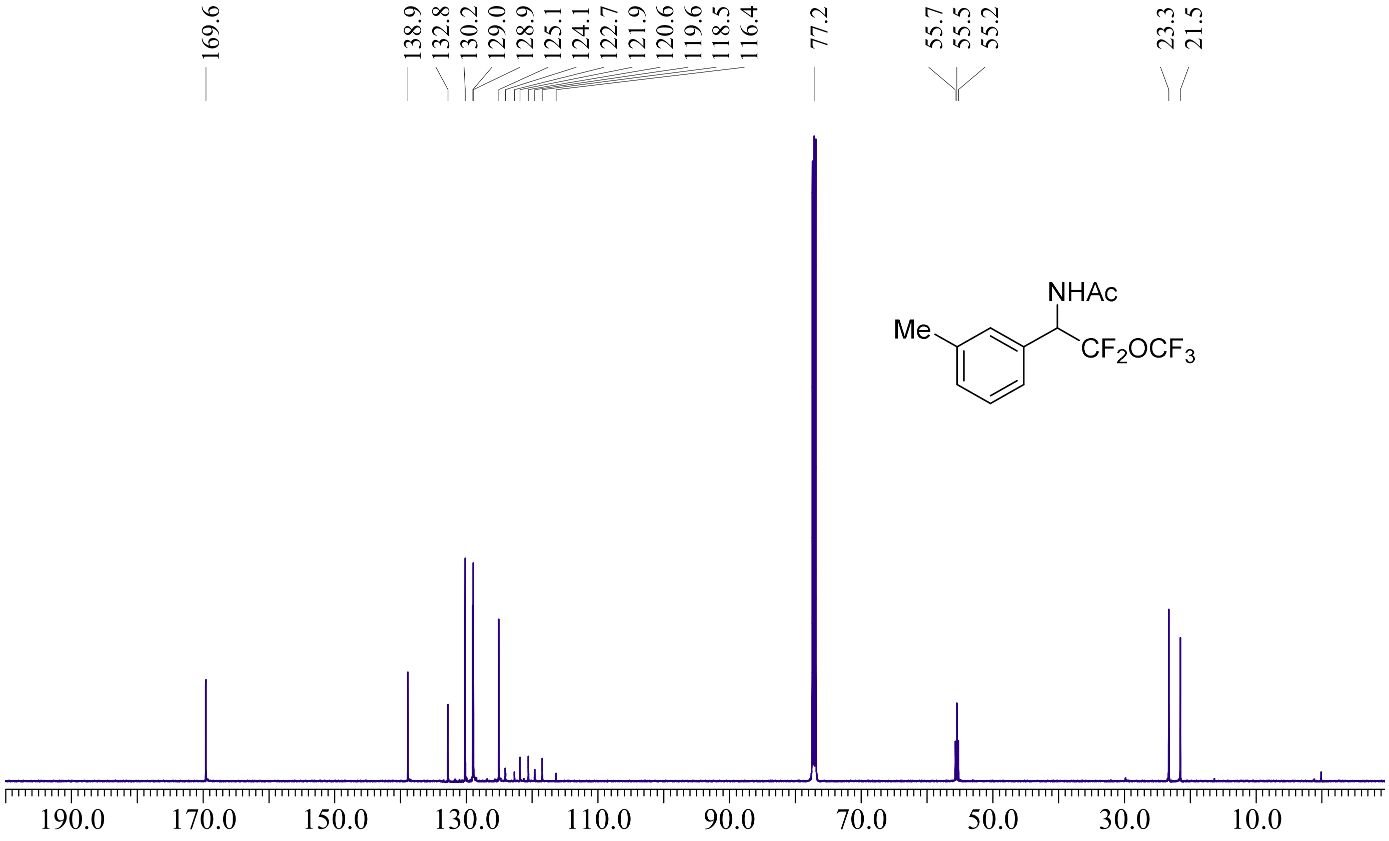


**$^{19}$F NMR** (282 MHz, $CDCl_3$) : **3m**

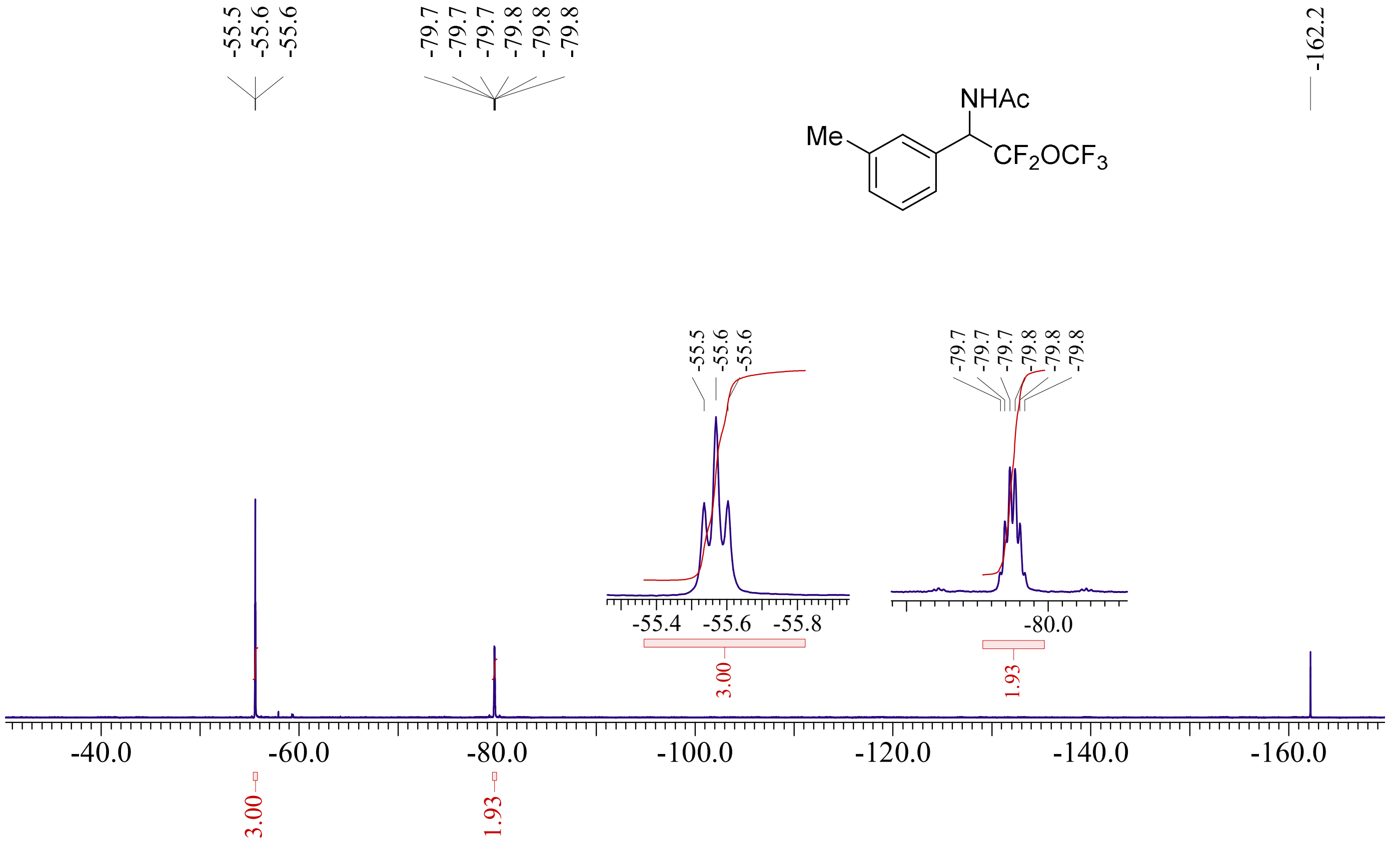

**$^{1}$H NMR** (500 MHz, $CDCl_3$) : **3n**

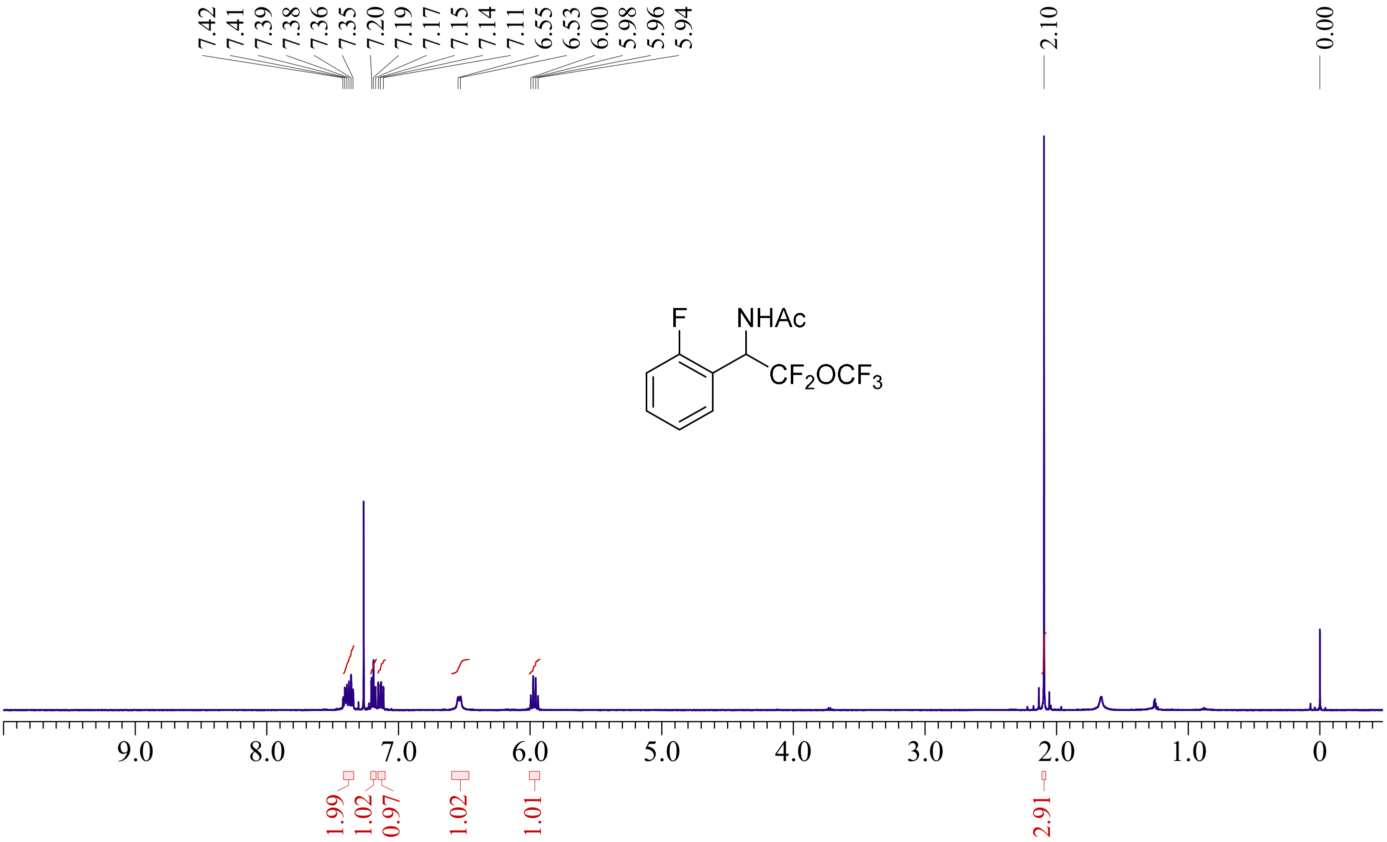


**$^{13}$C NMR** (126 MHz, $CDCl_3$) : **3n**

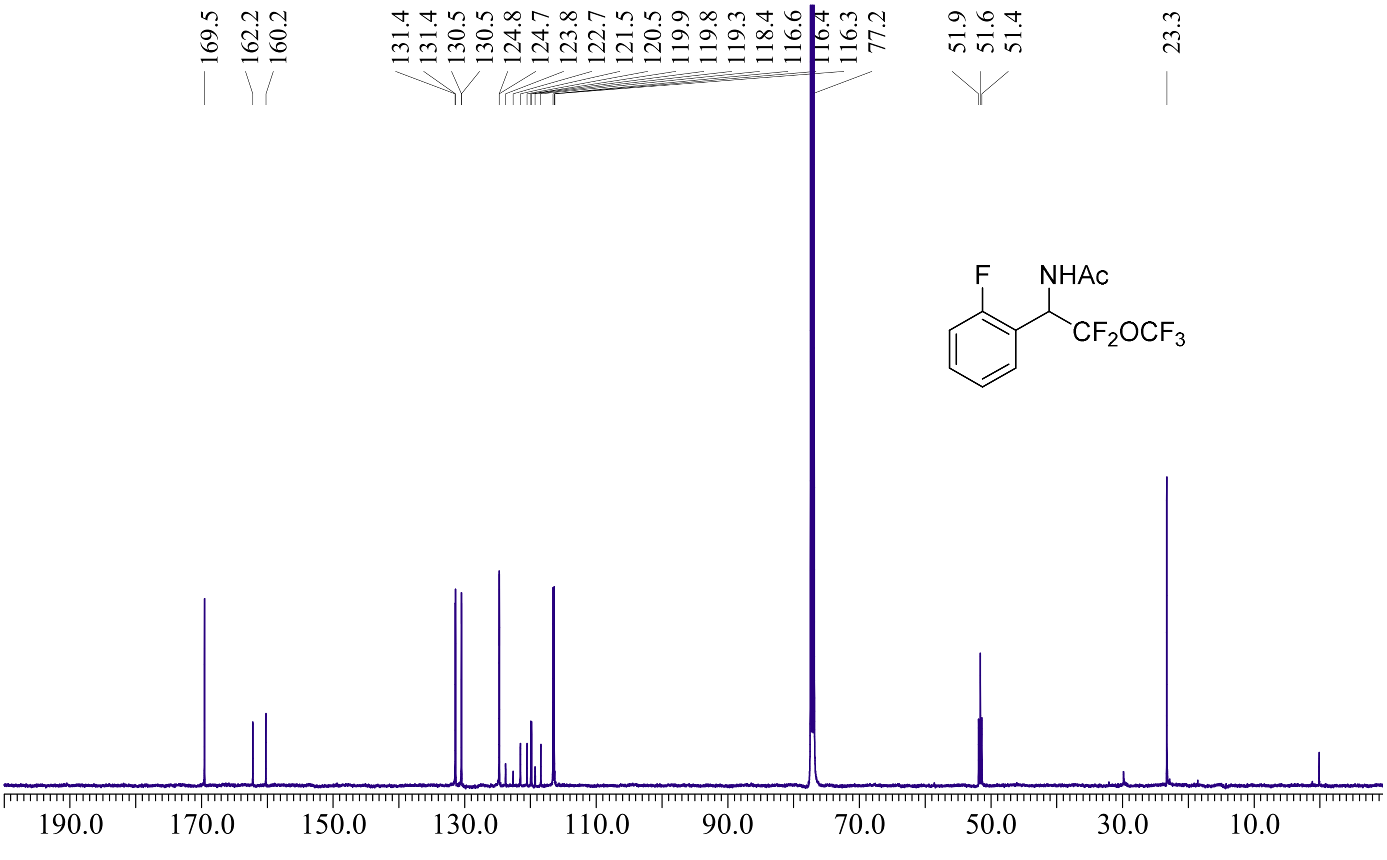

**$^{19}$F NMR** (282 MHz, CDCl3) : **3n**

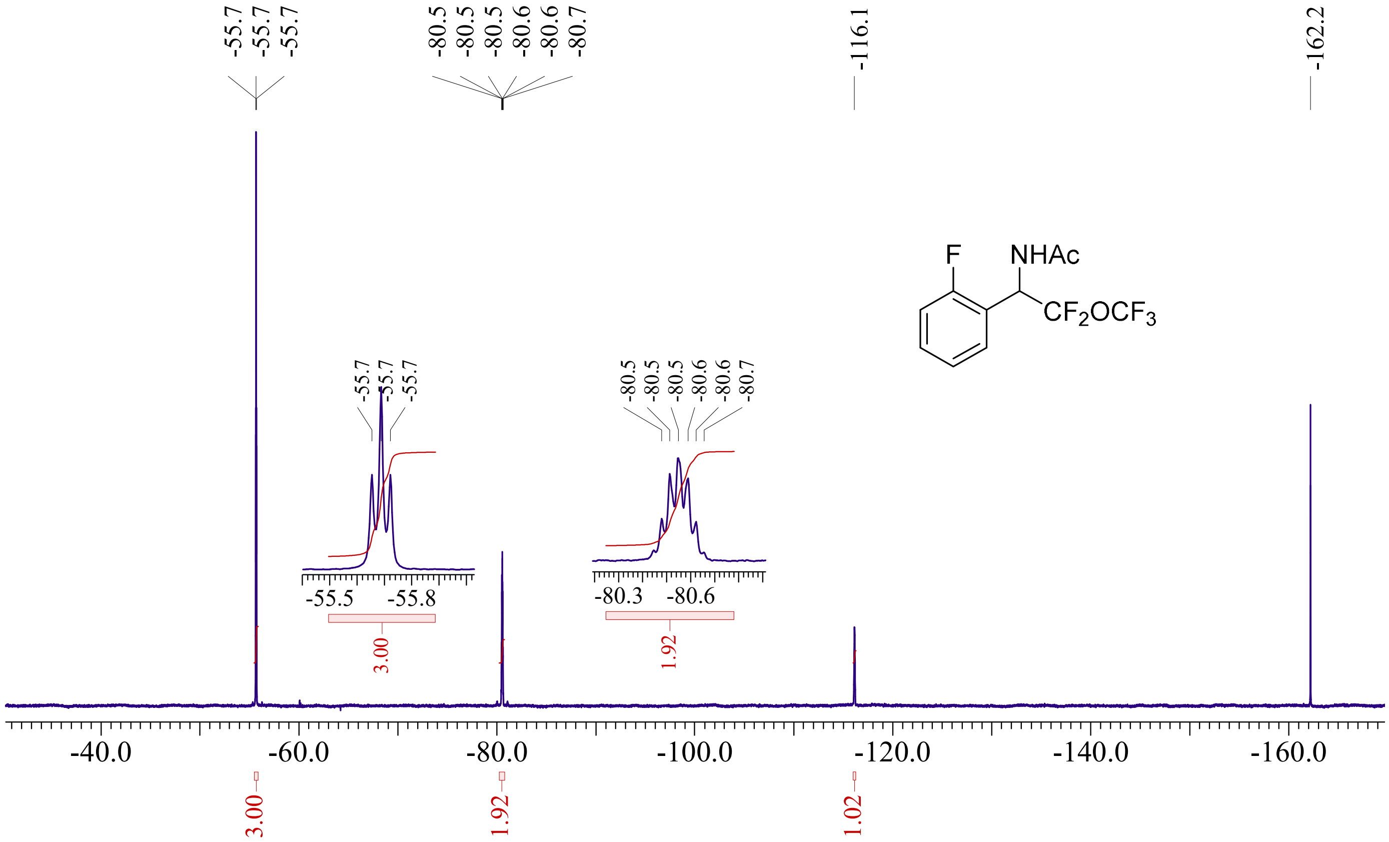


**$^{1}$H NMR** (500 MHz, CDCl3) : **3o**

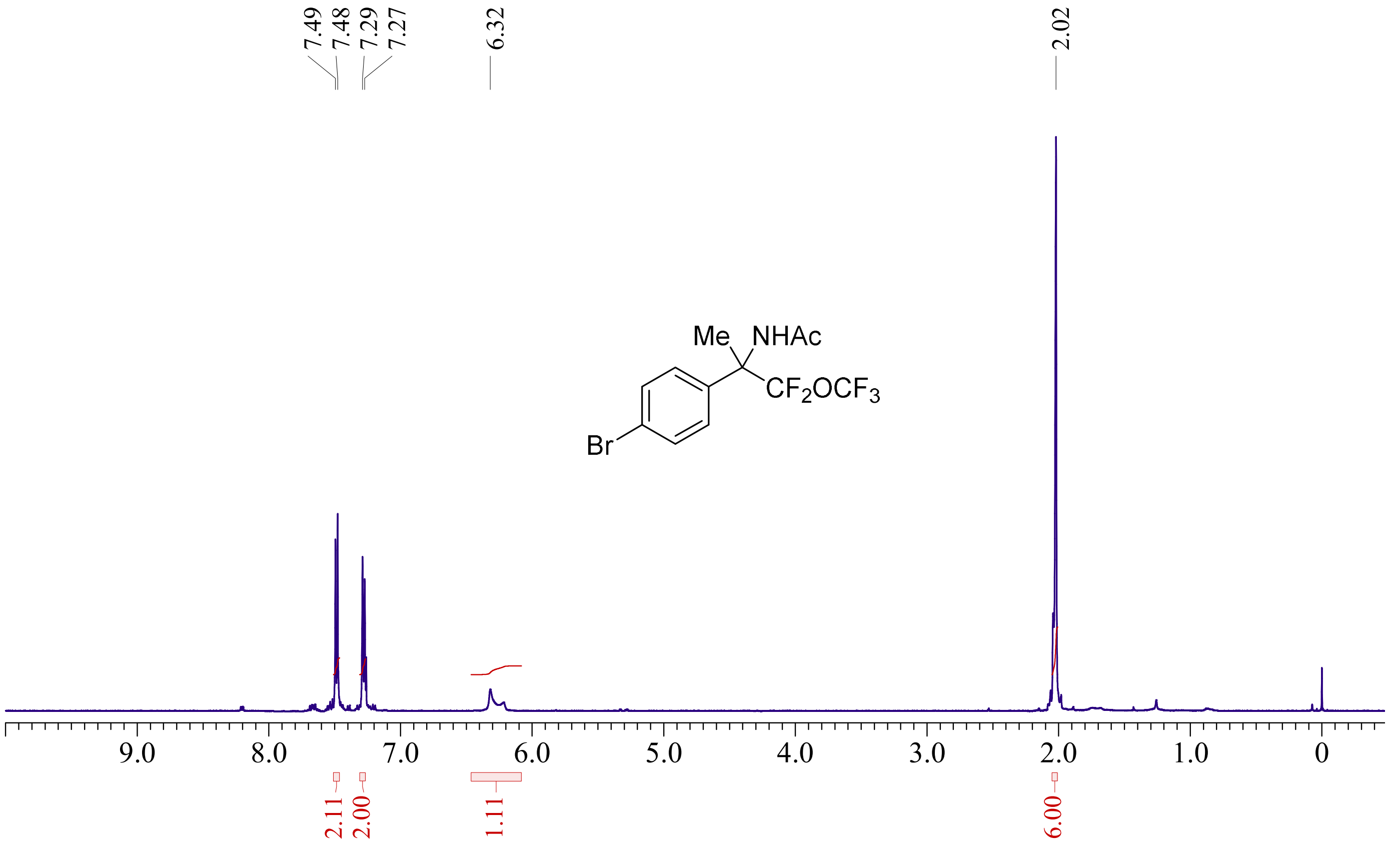

**$^{13}$C NMR** (126 MHz, CDCl$_3$) : **3o**

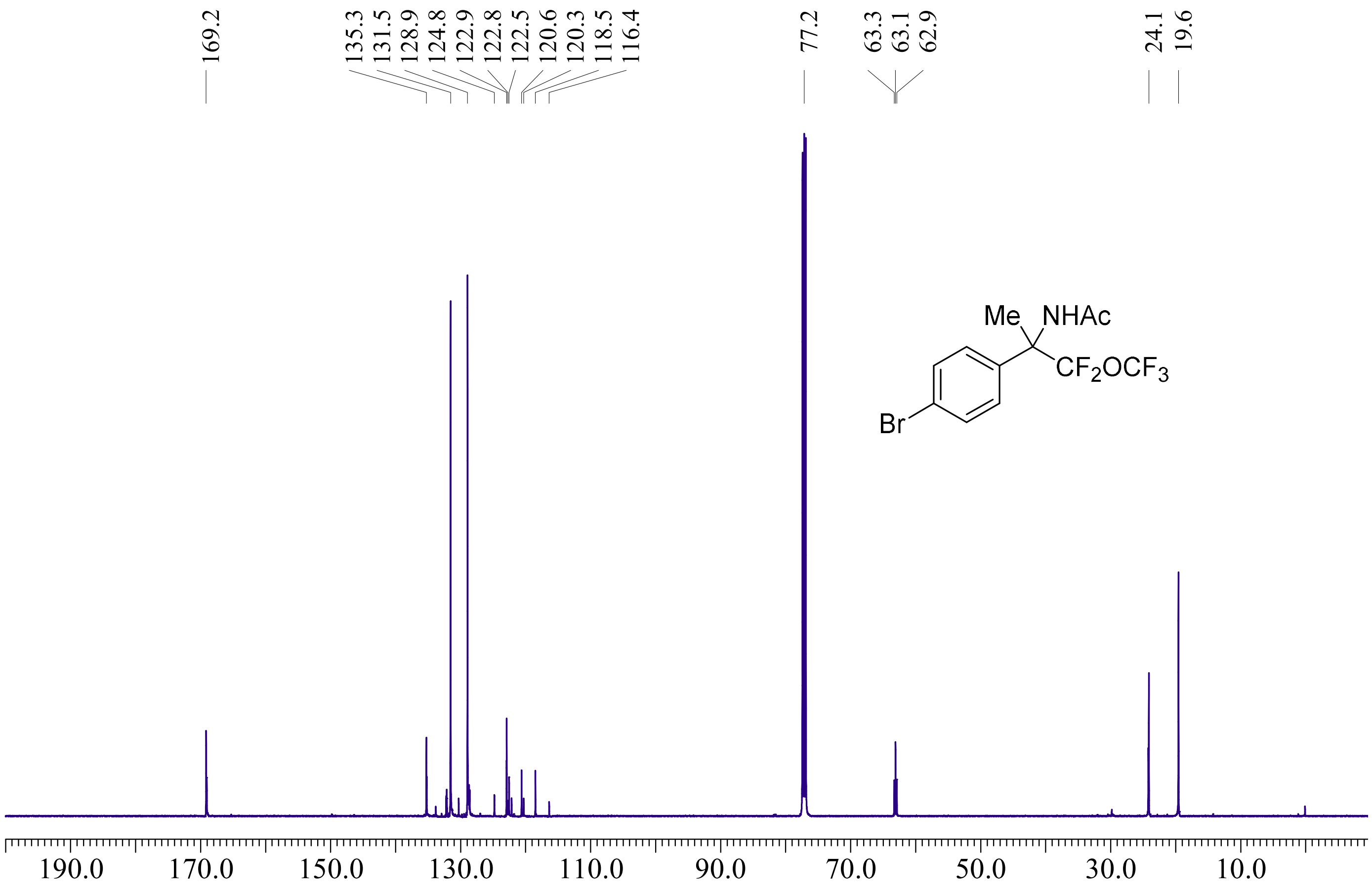


**$^{19}$F NMR** (282 MHz, CDCl$_3$) : **3o**

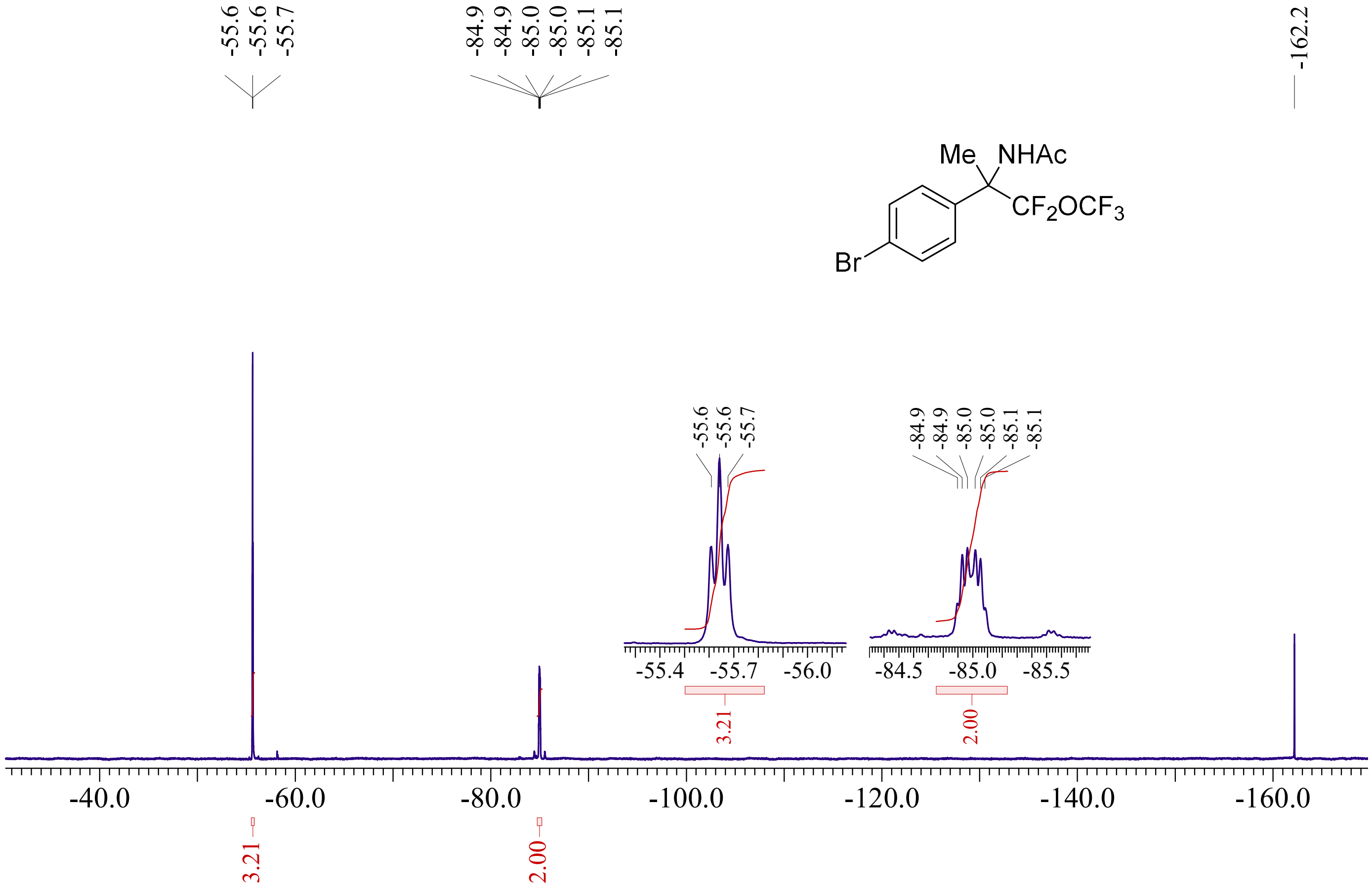

☆

**$^{1}$H NMR** (500 MHz, $CDCl_3$) : **3p**

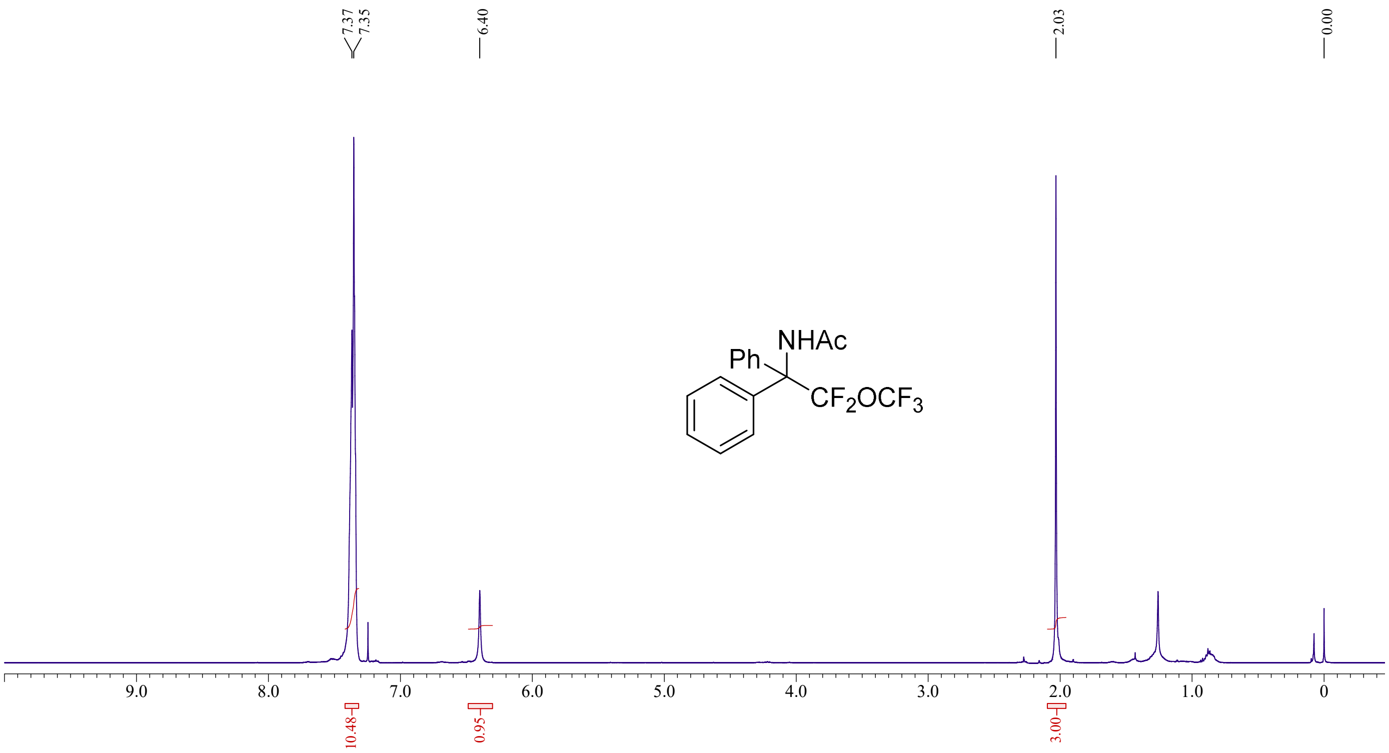


**$^{13}$C NMR** (126 MHz, $CDCl_3$) : **3p**

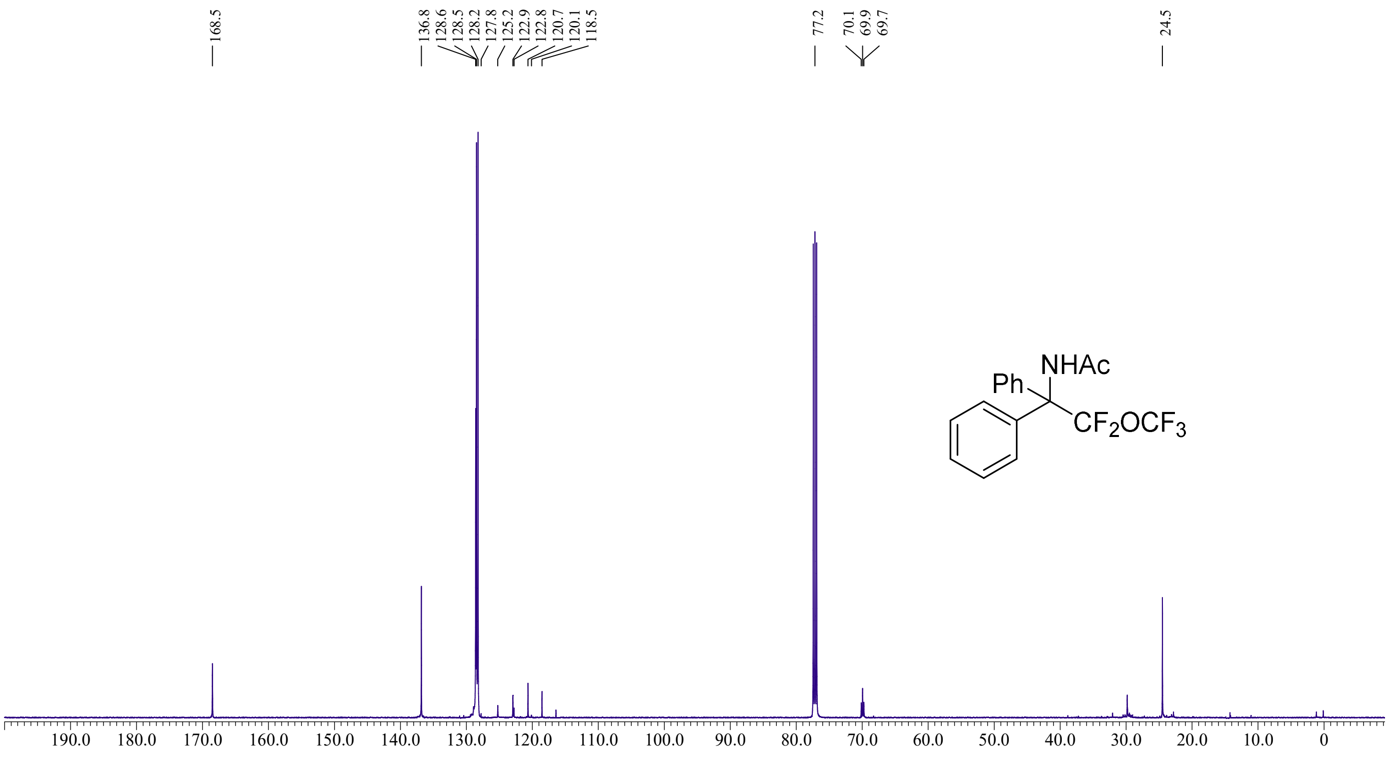

**$^{19}$F NMR** (282 MHz, $CDCl_3$) : **3p**

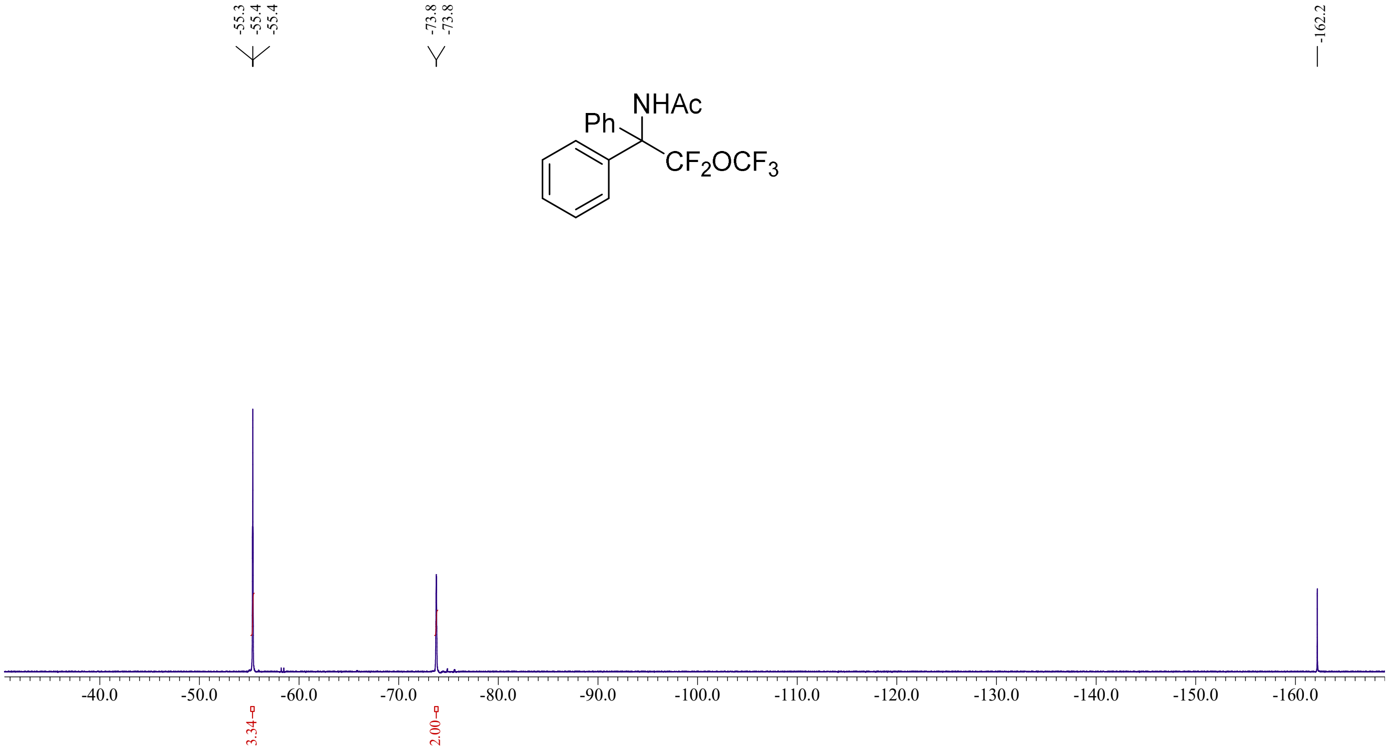


**$^{1}$H NMR** (500 MHz, $CDCl_3$) : **3q**

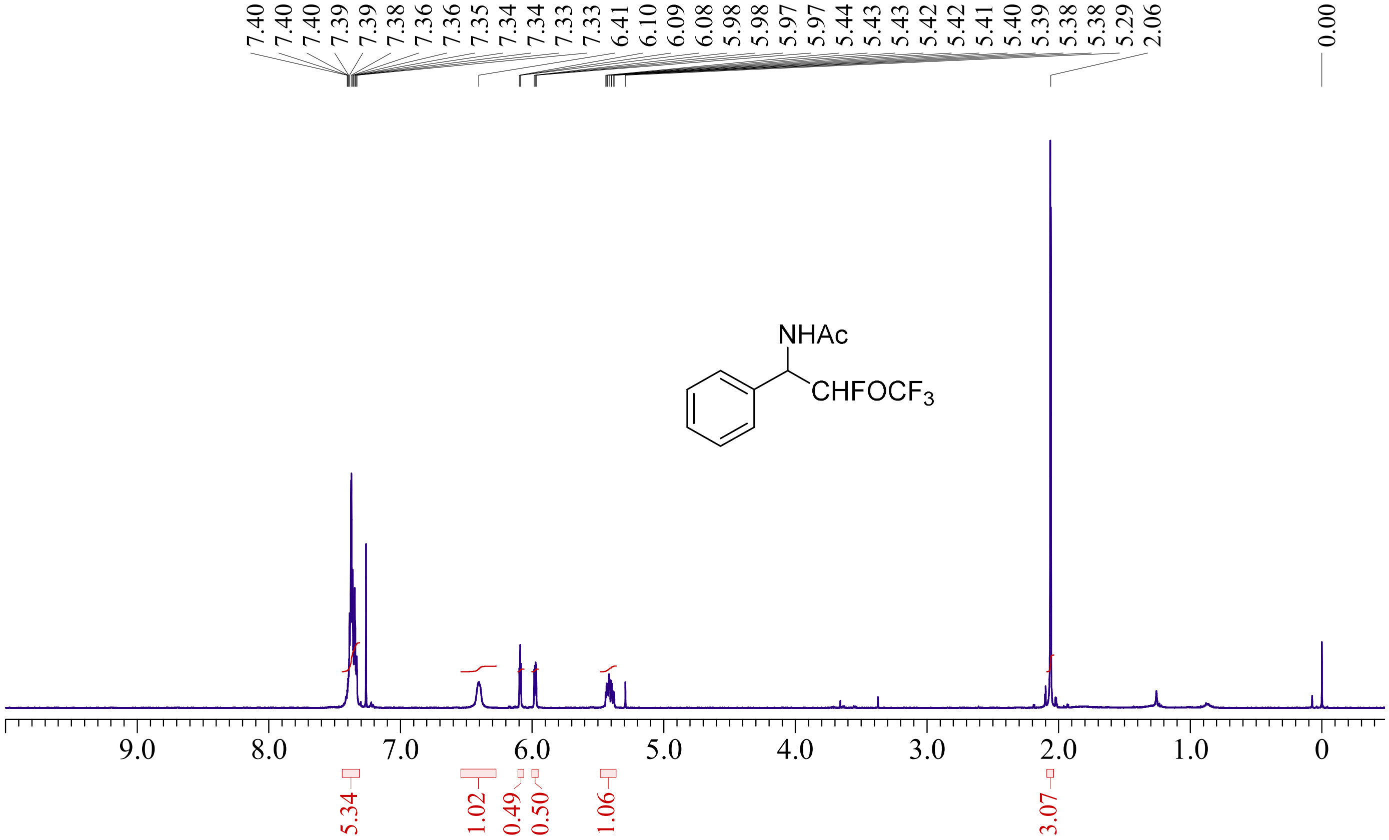

**$^{13}$C NMR** (126 MHz, $CDCl_3$) : **3q**

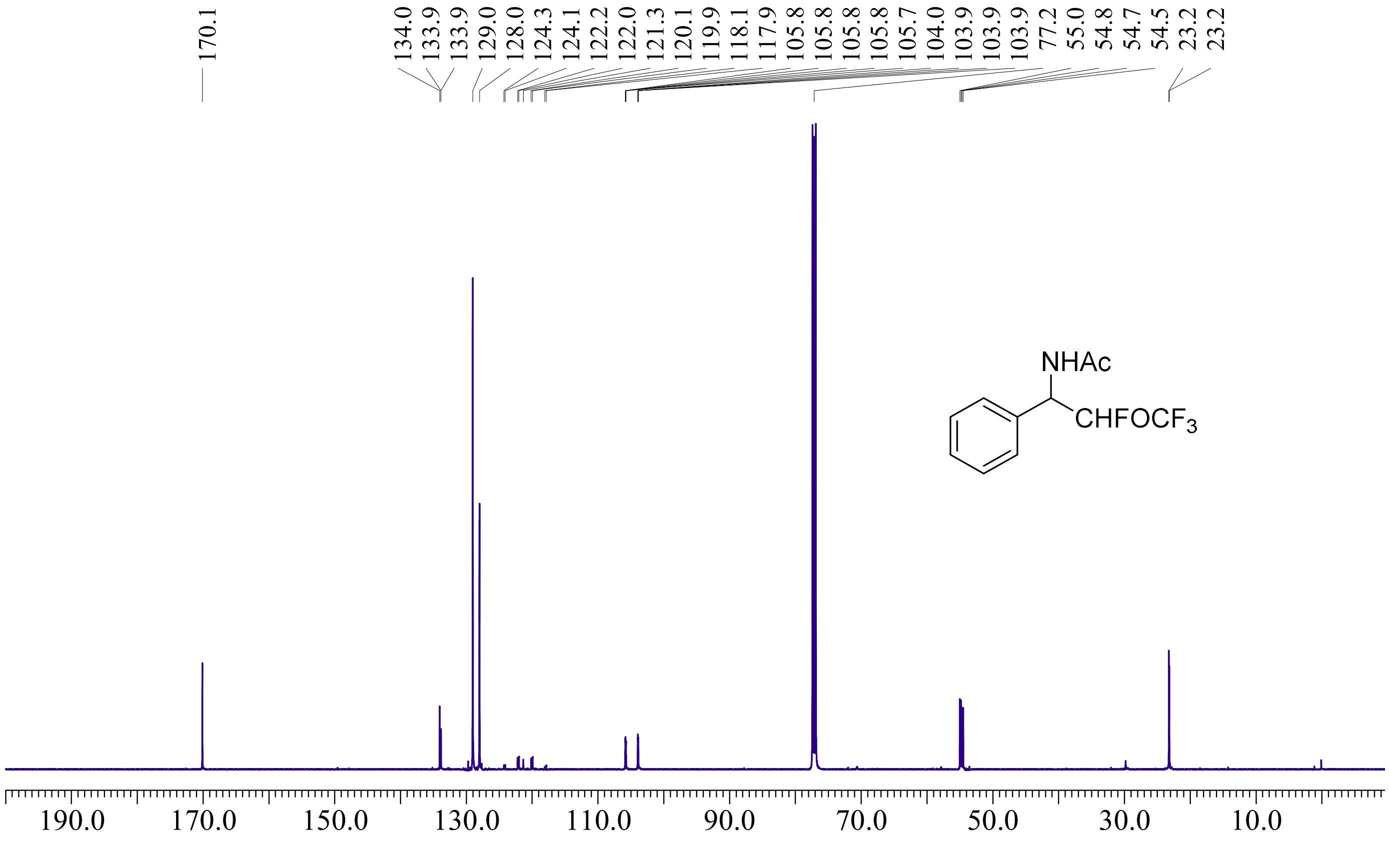


**$^{19}$F NMR** (282 MHz, $CDCl_3$) : **3q**

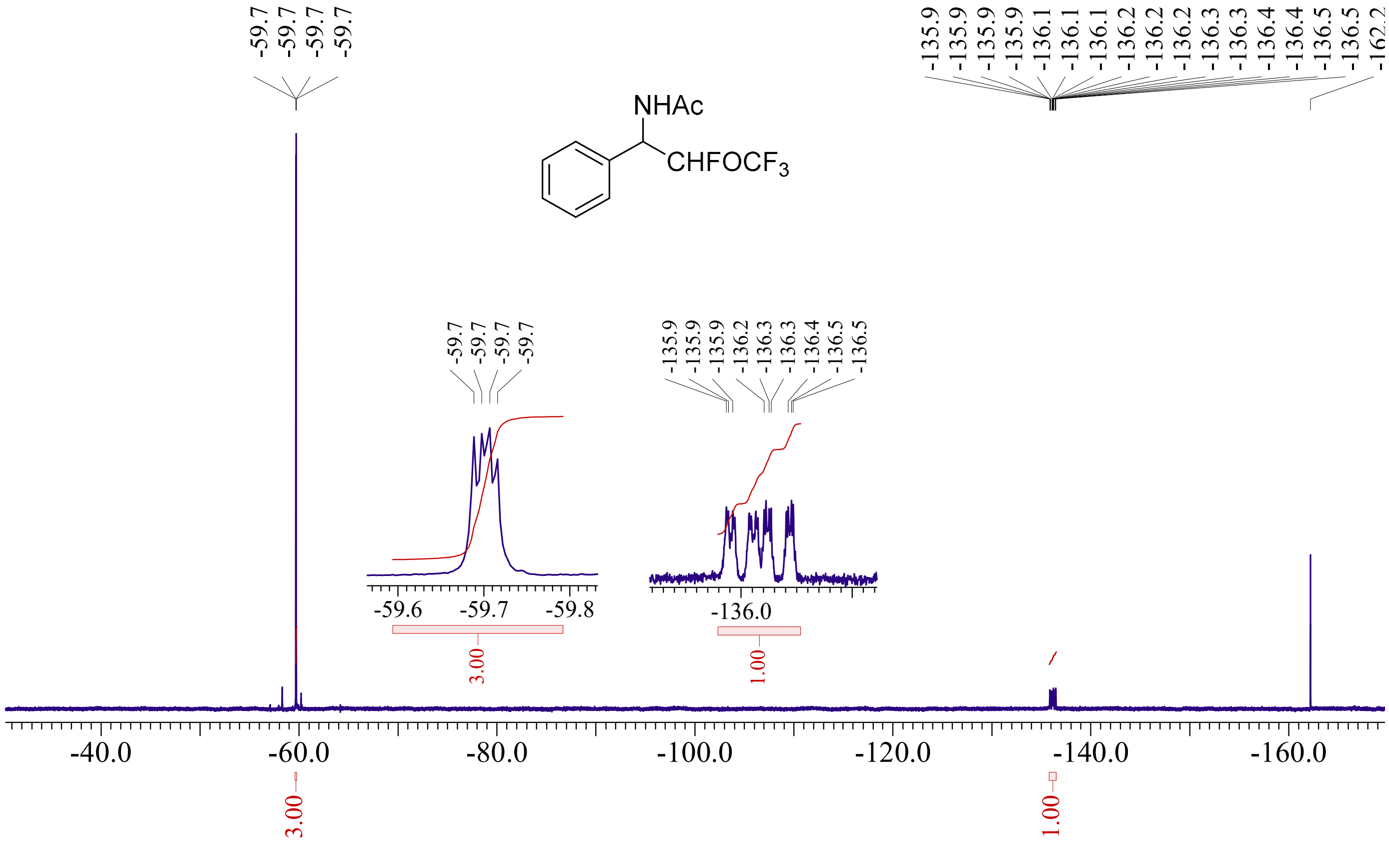

**$^{1}$H NMR** (500 MHz, $CDCl_3$) : **3s**

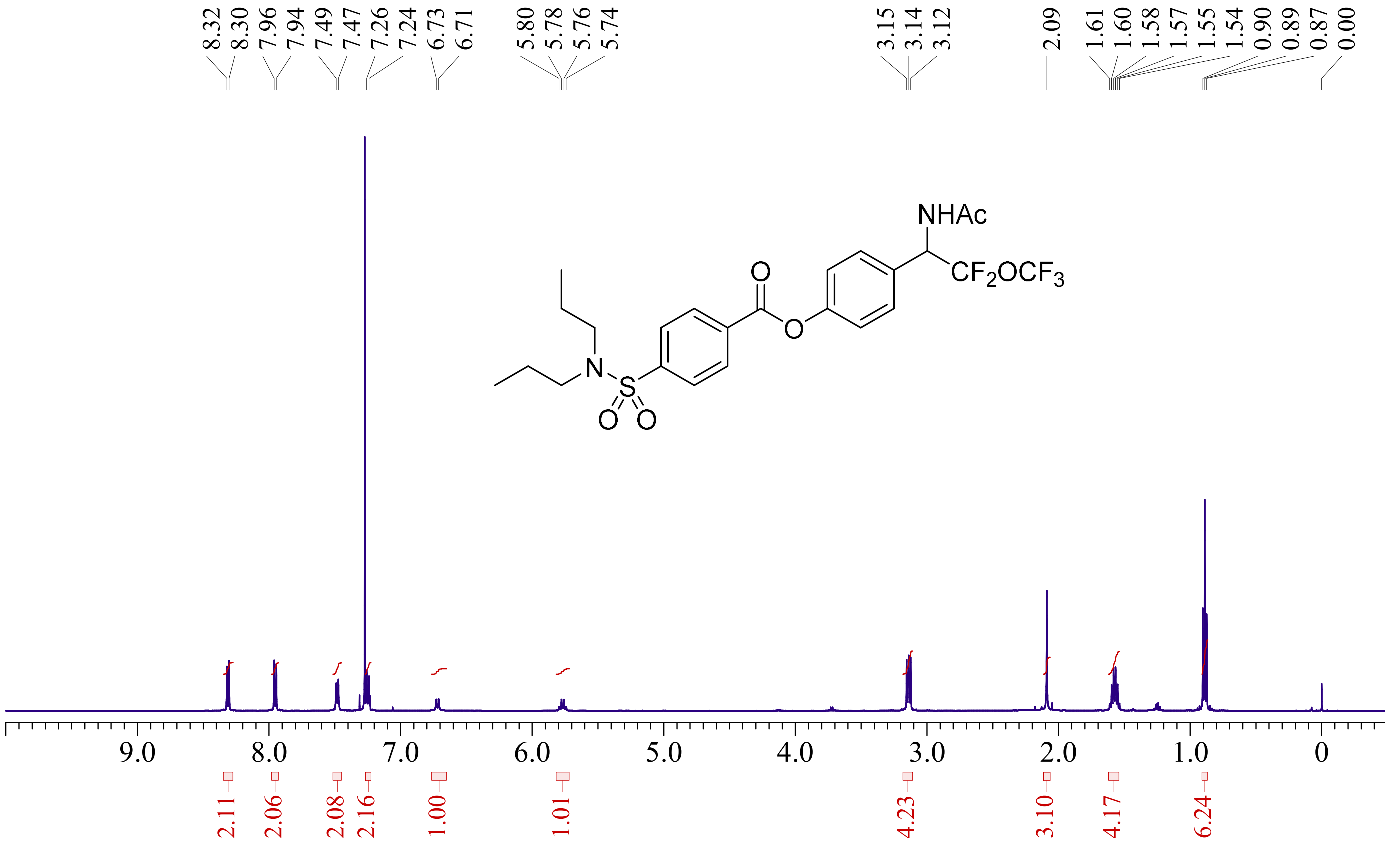


**$^{13}$C NMR** (126 MHz, $CDCl_3$) : **3s**

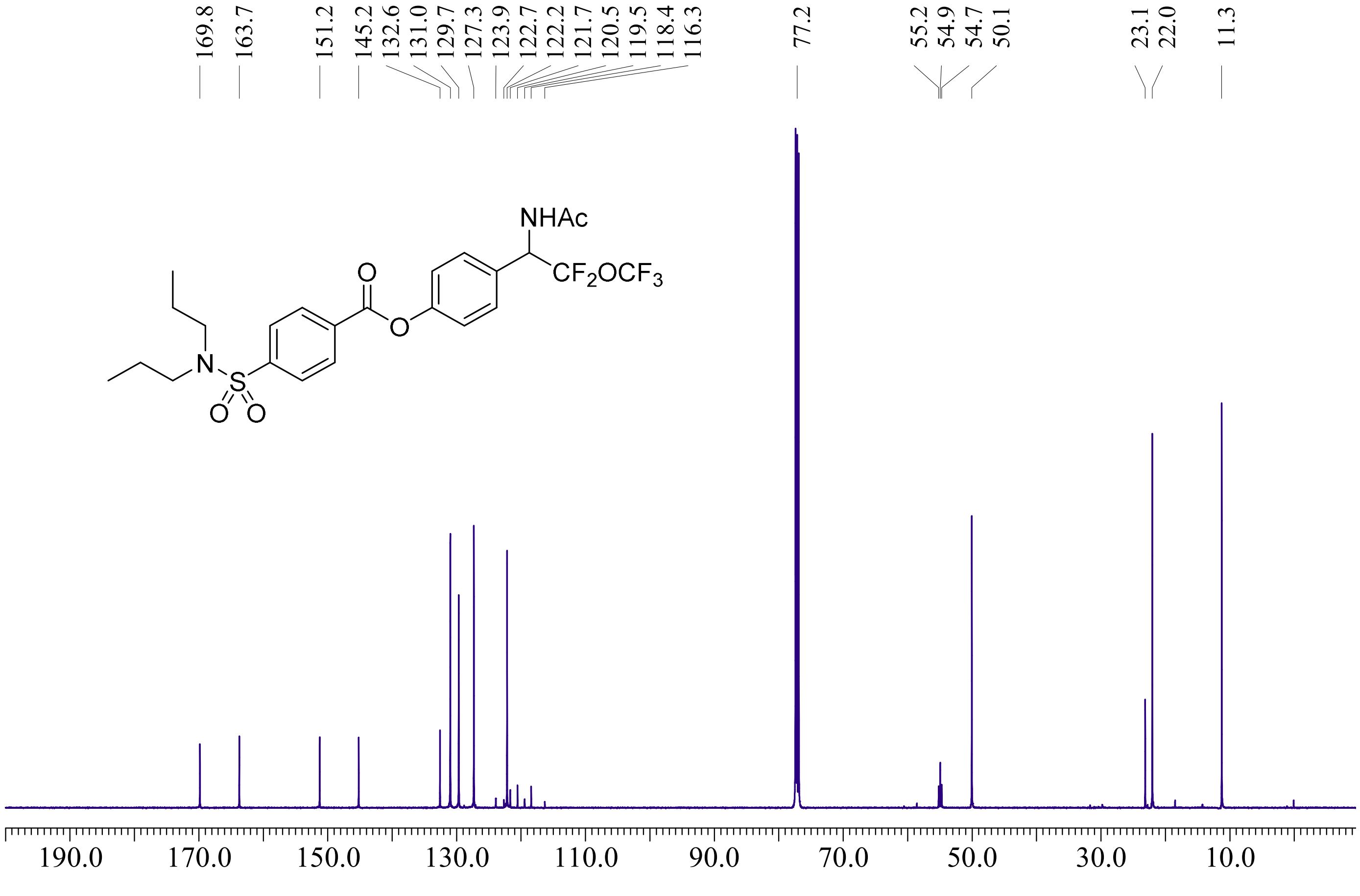

**$^{19}$F NMR** (282 MHz, $CDCl_3$) : **3s**

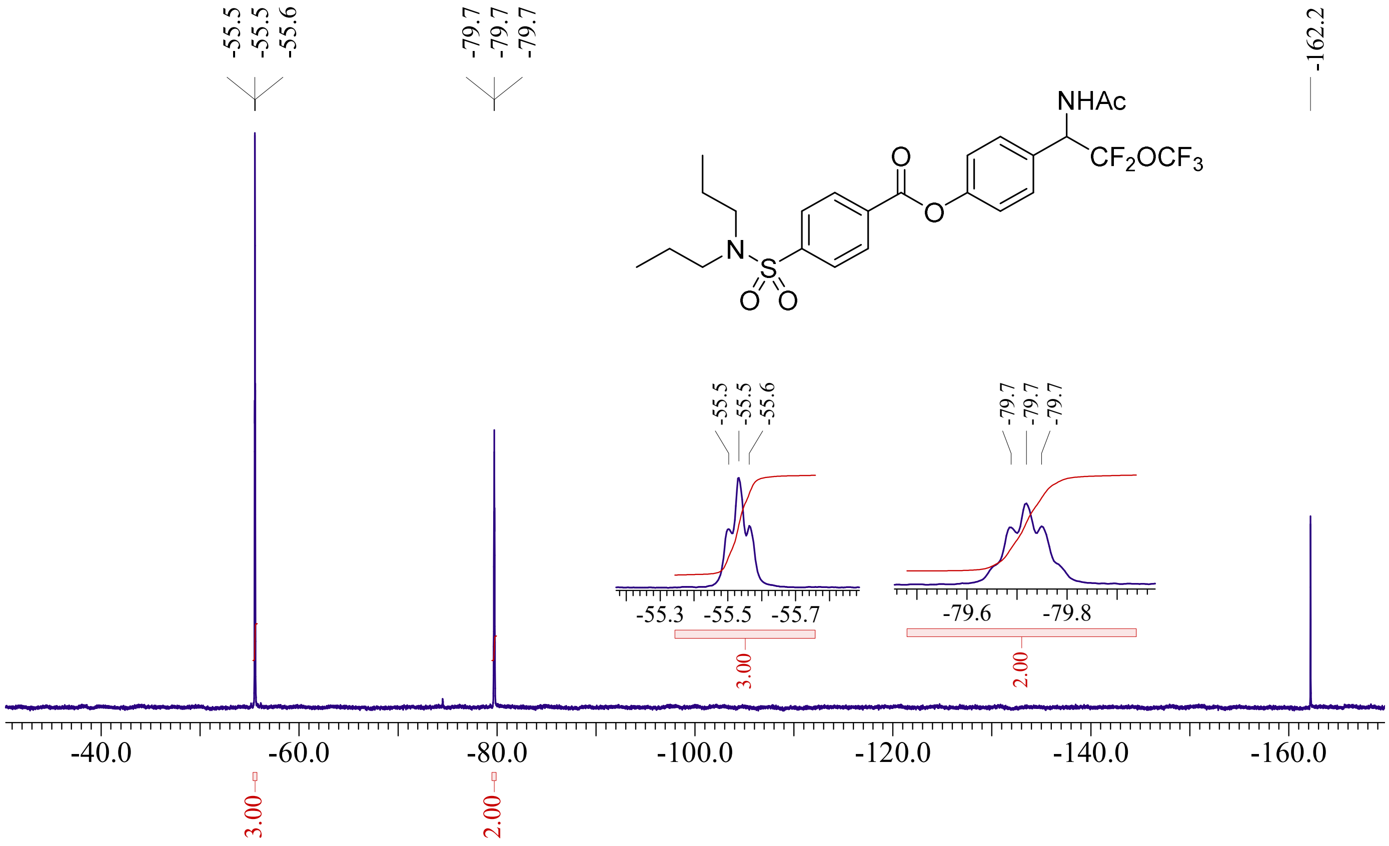


**$^{1}$H NMR** (500 MHz, $CDCl_3$) : **3t**

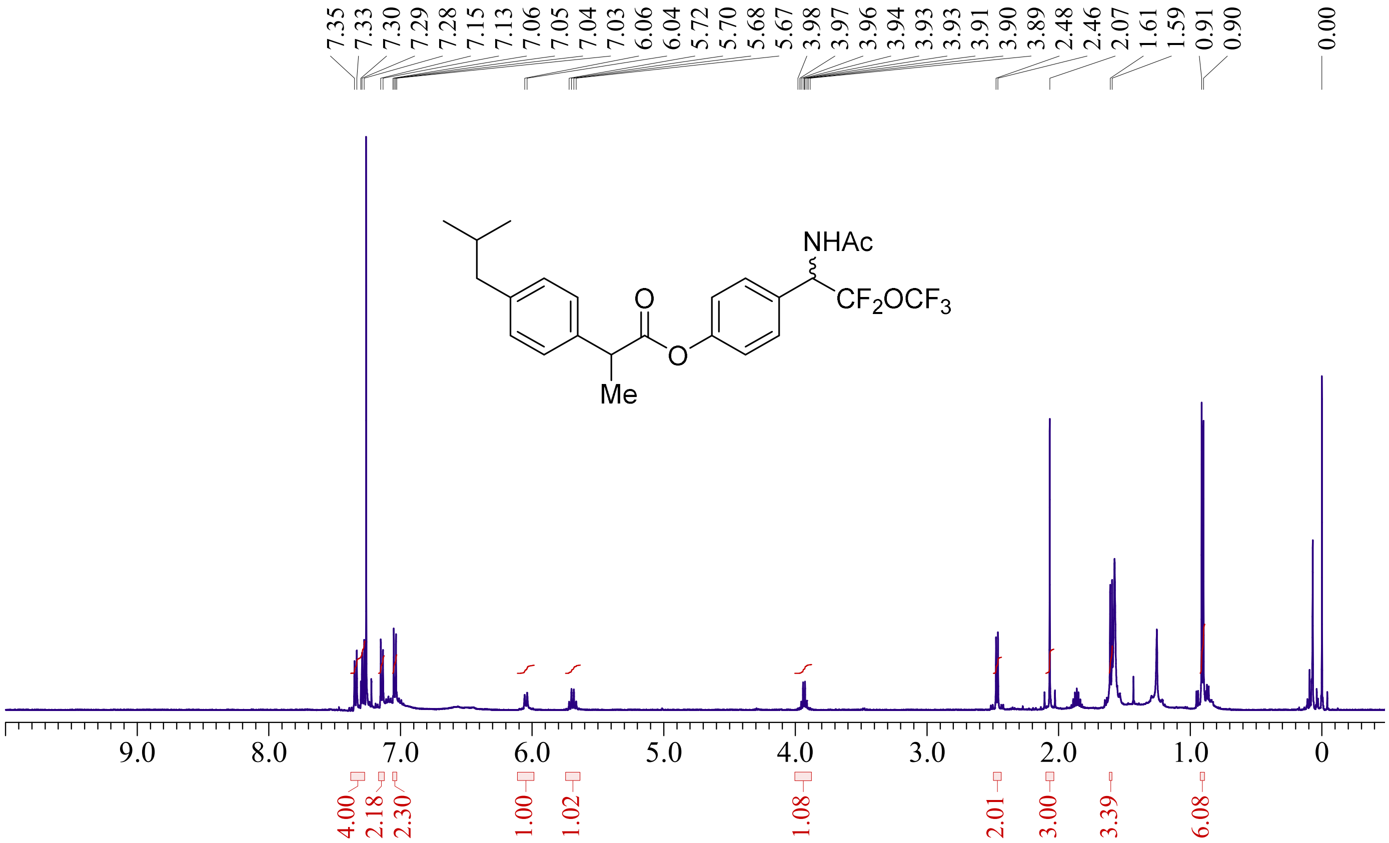

**$^{13}$C NMR** (126 MHz, $CDCl_3$) : **3t**

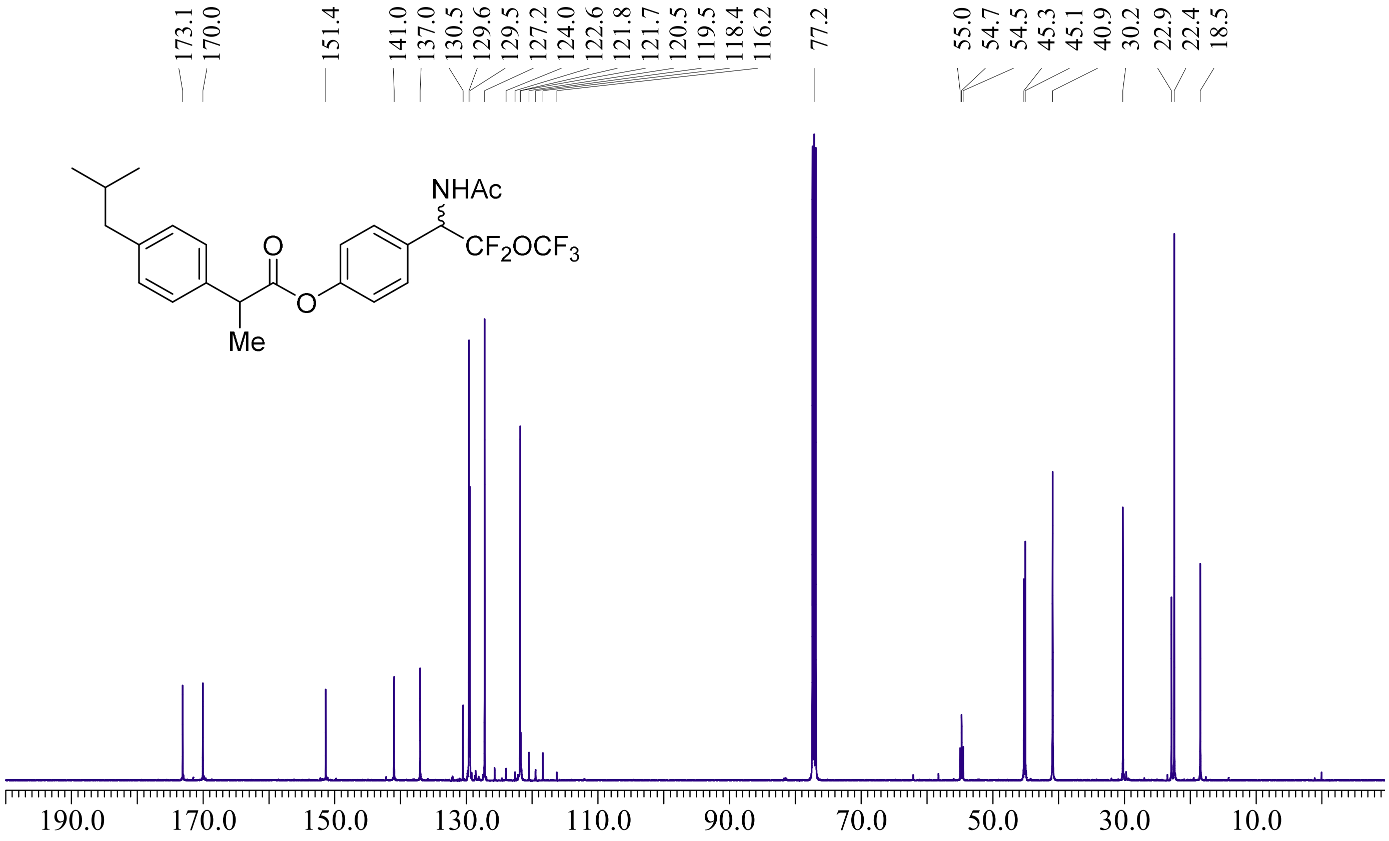


**$^{19}$F NMR** (282 MHz, $CDCl_3$) : **3t**

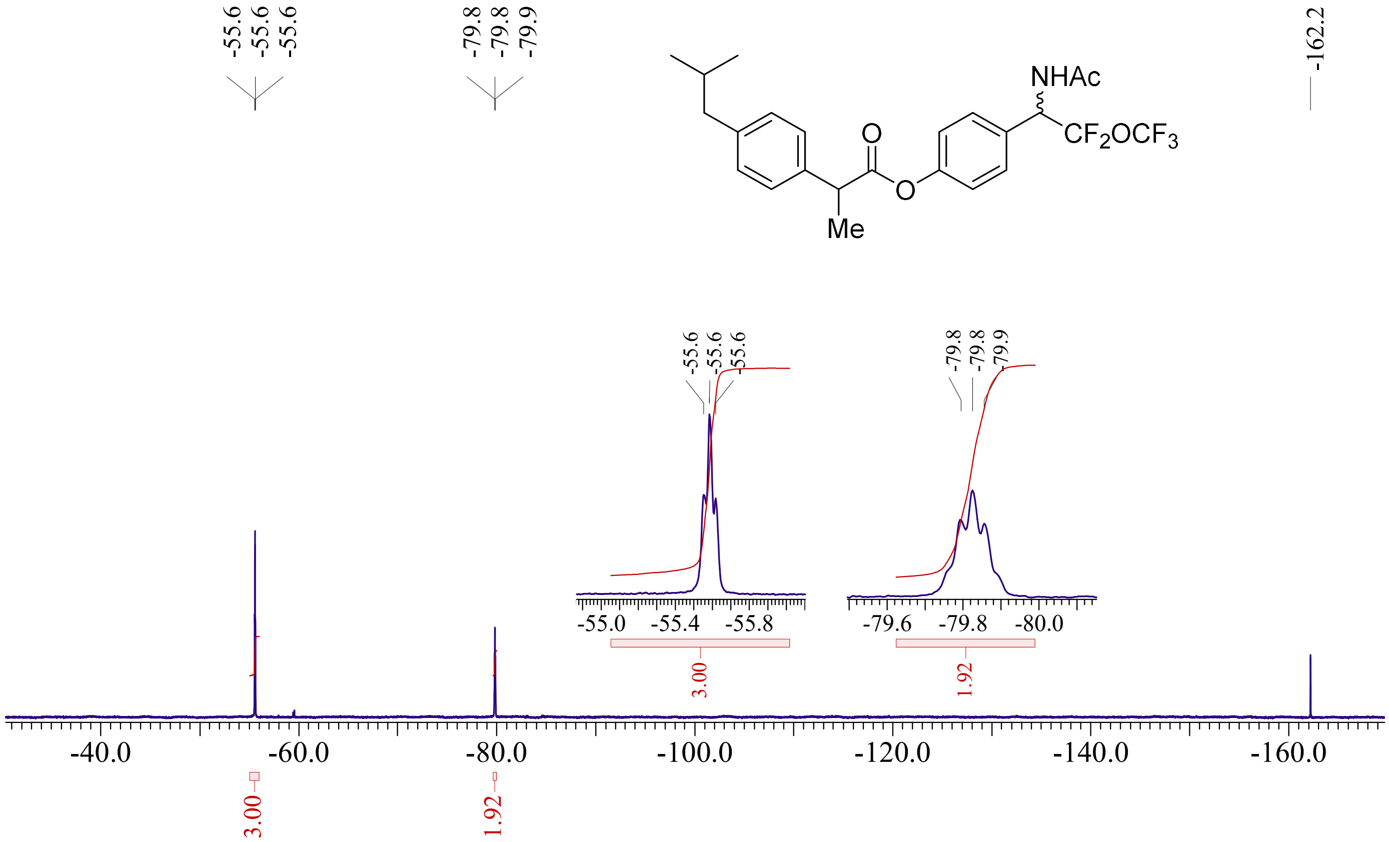

**$^{1}$H NMR** (500 MHz, CDCl3) : **3u**

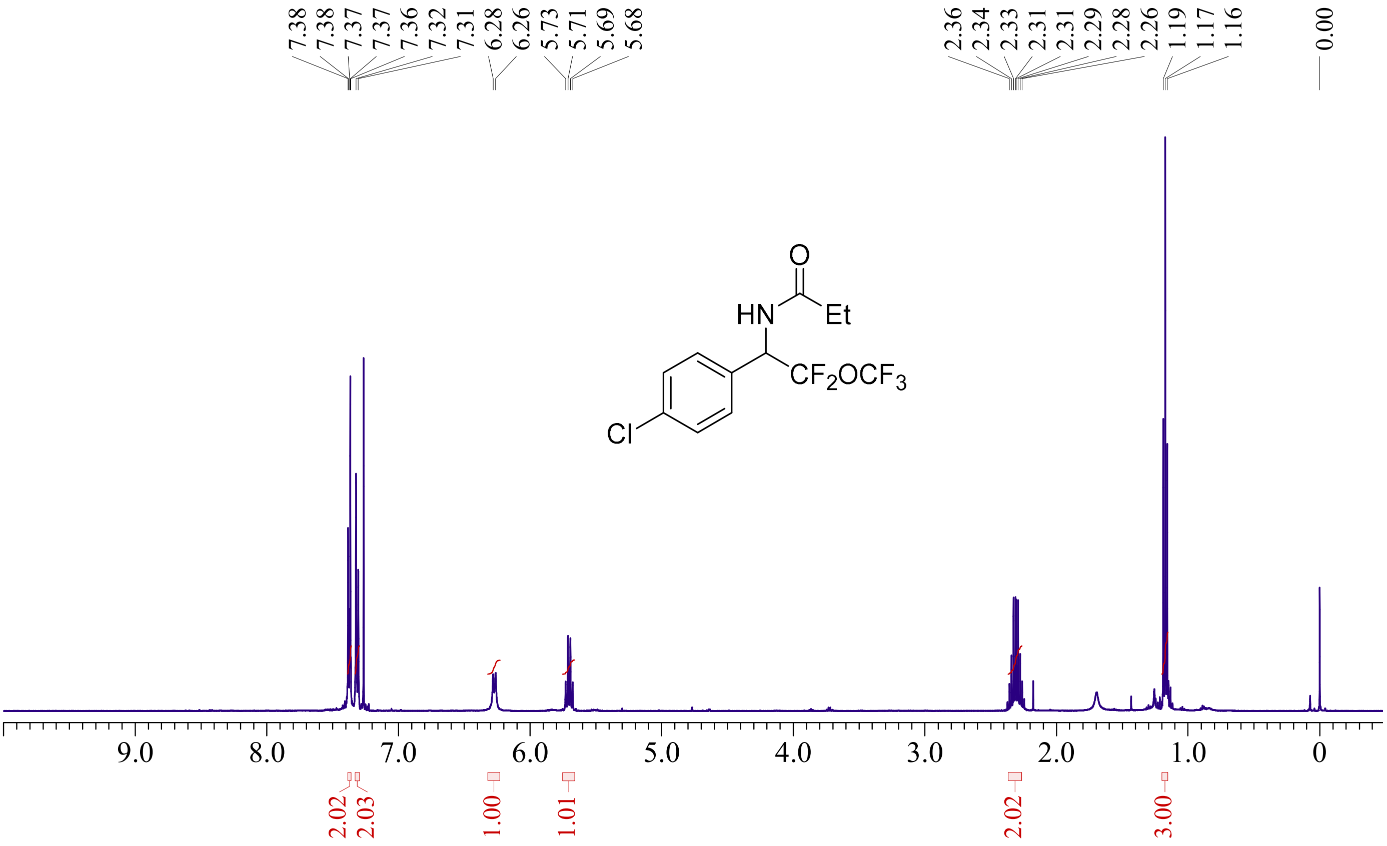


**$^{13}$C NMR** (126 MHz, CDCl3) : **3u**

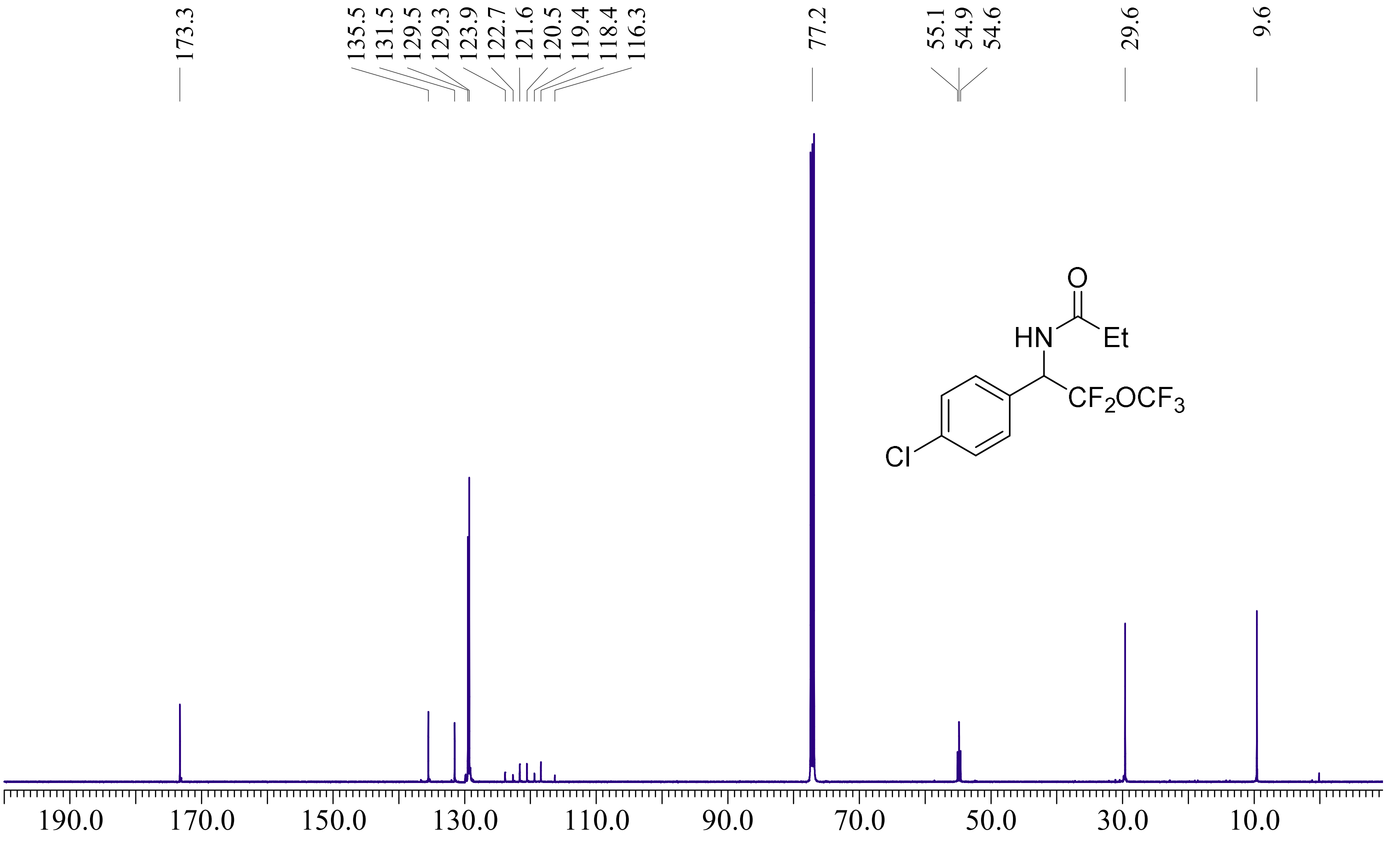

**$^{19}$F NMR** (282 MHz, CDCl$_3$) : **3u**

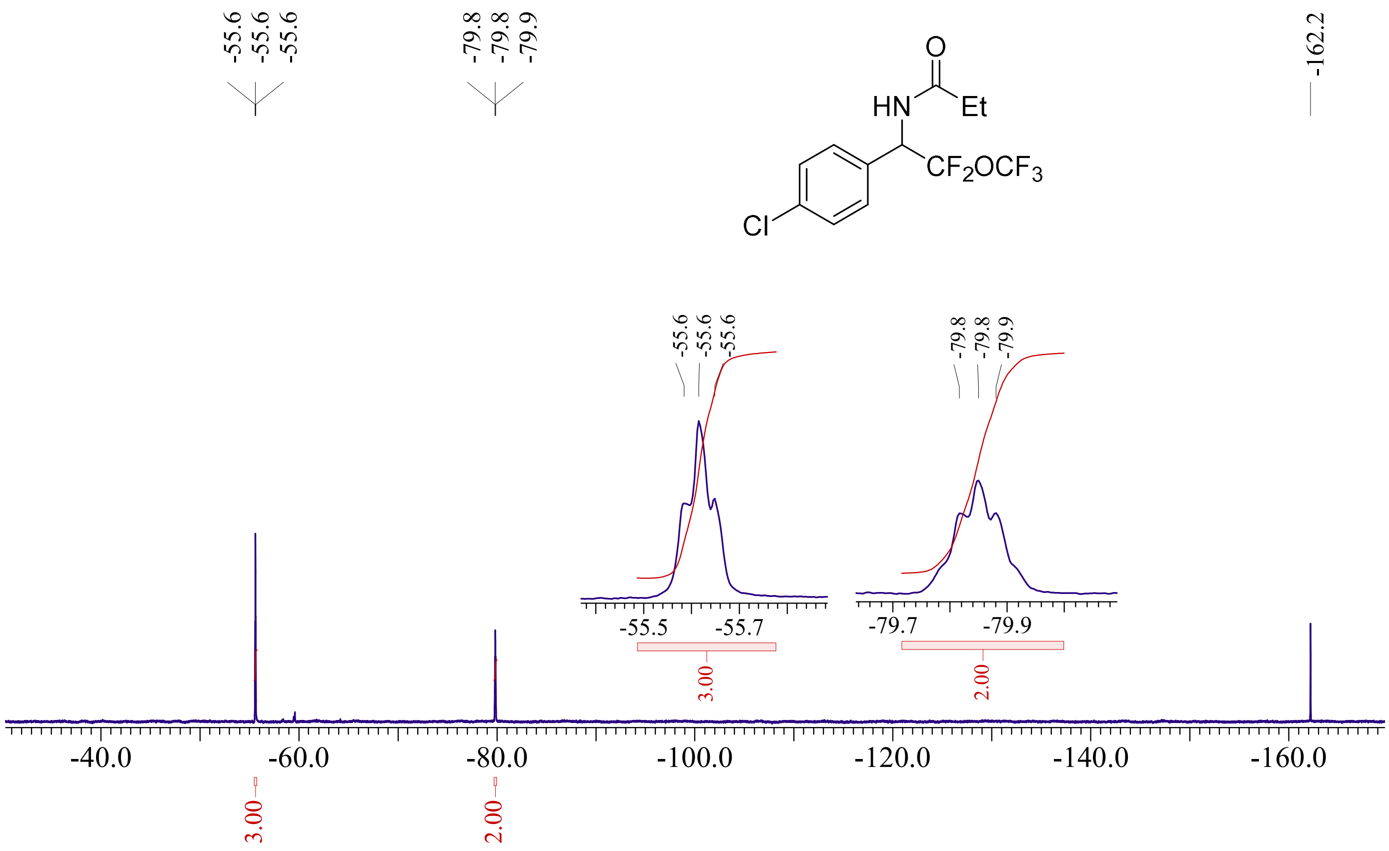

**$^{1}$H NMR** (500 MHz, CDCl$_3$) : **3a-*d*$_3$**

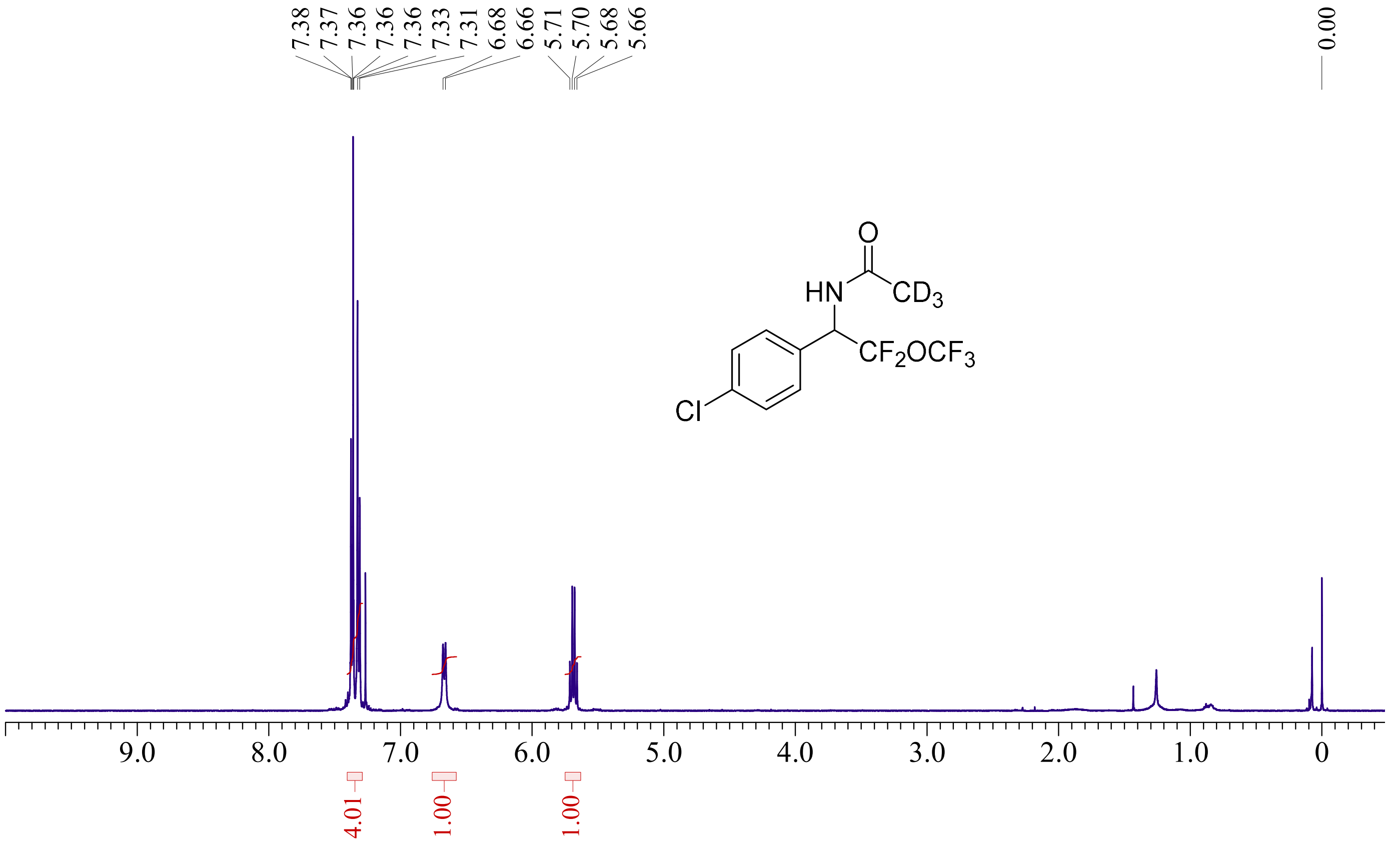

**$^{13}$C NMR** (126 MHz, CDCl$_3$) : **3a-*d*$_3$**

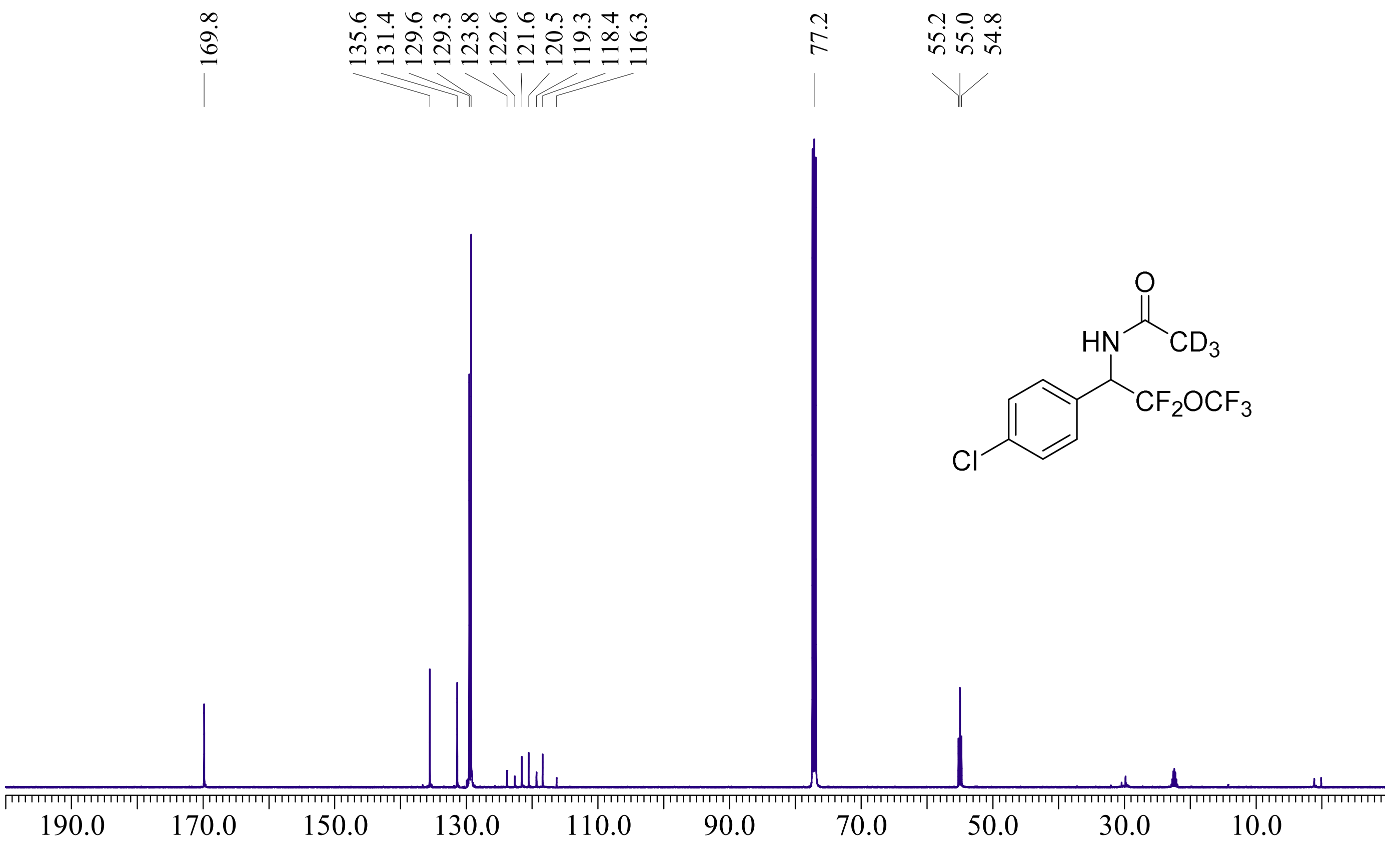


**$^{19}$F NMR** (282 MHz, CDCl$_3$) : **3a-*d*$_3$**

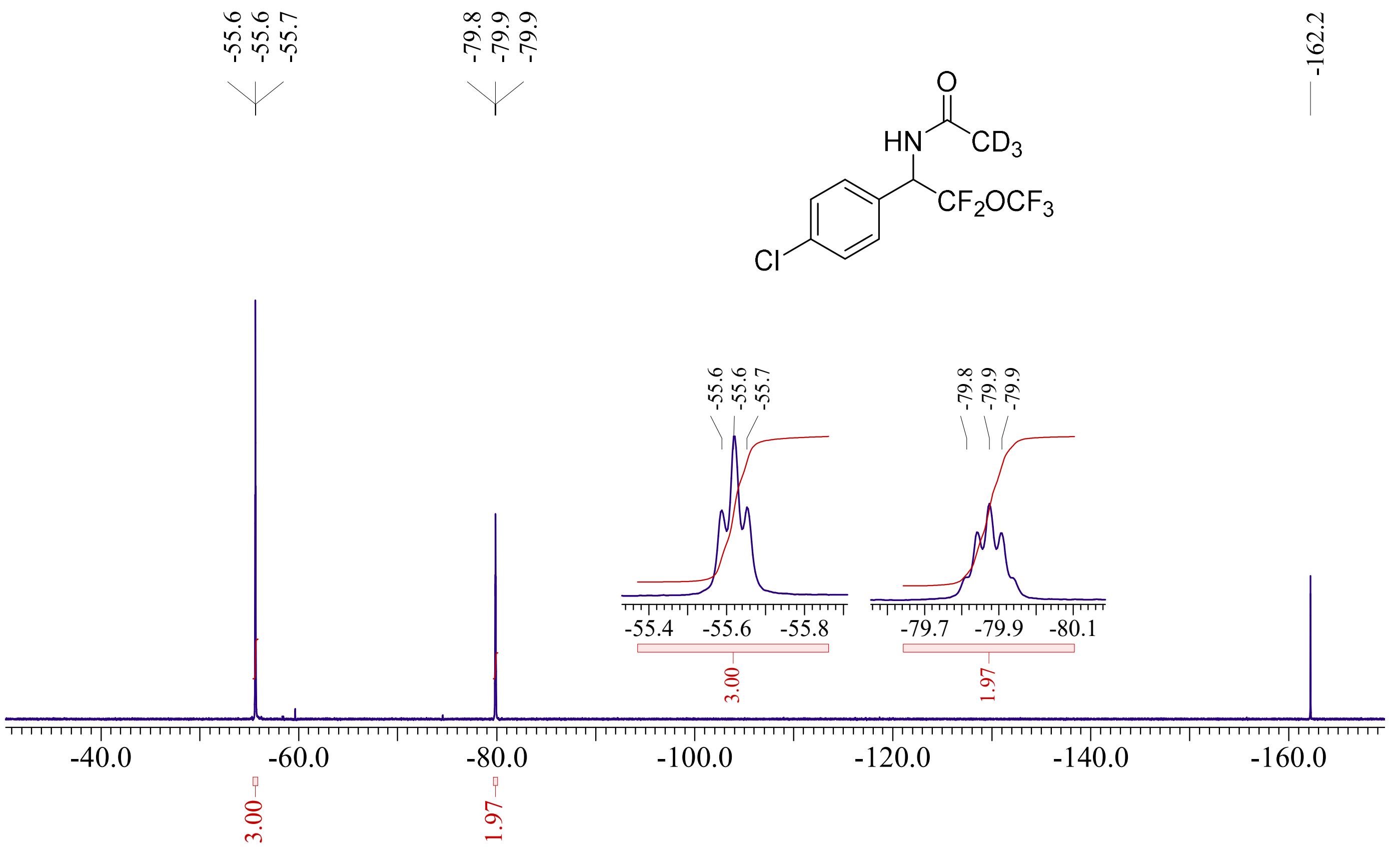

**$^{1}$H NMR** (500 MHz, $CDCl_3$) : **3v**

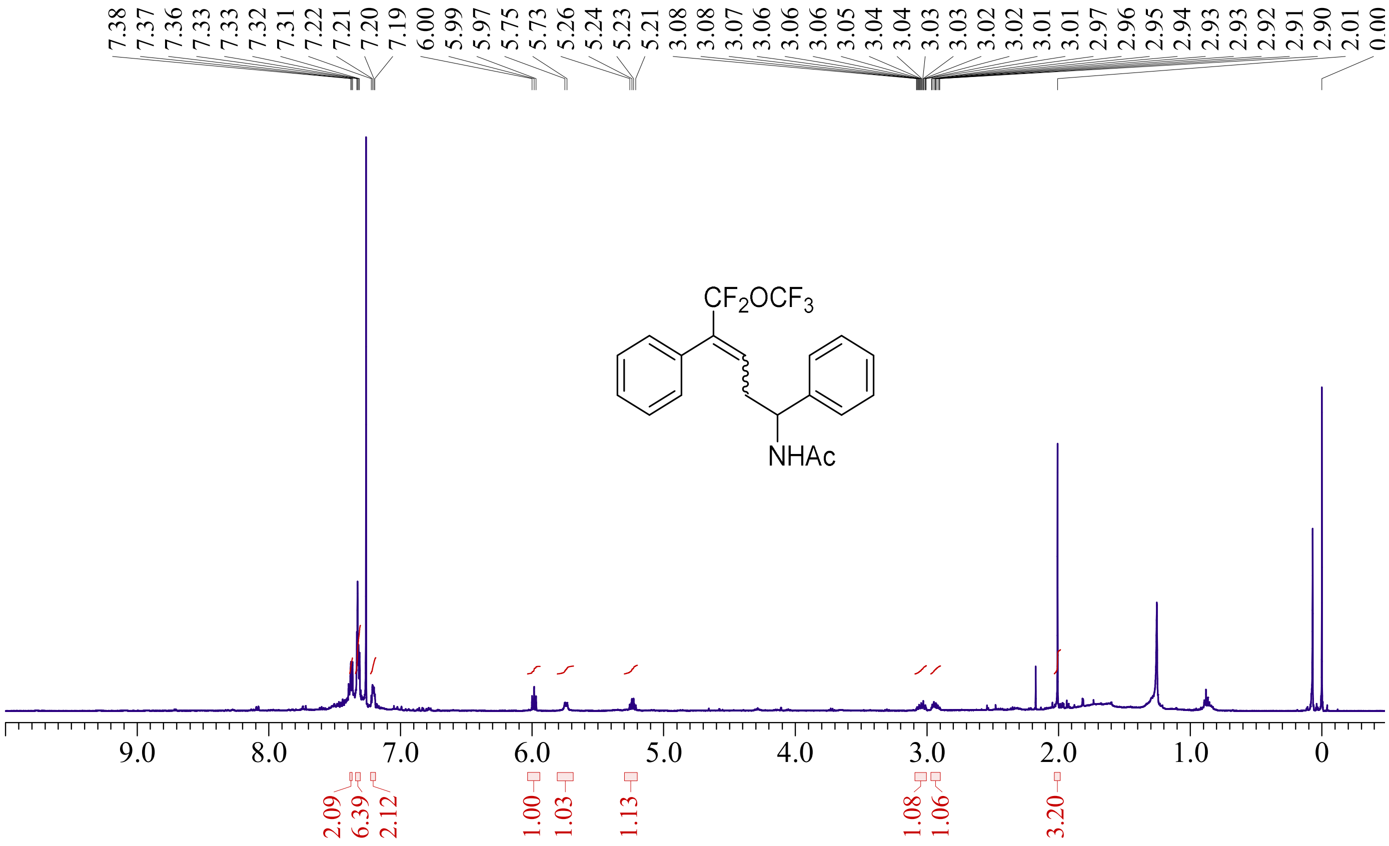


**$^{13}$C NMR** (126 MHz, $CDCl_3$) : **3v**

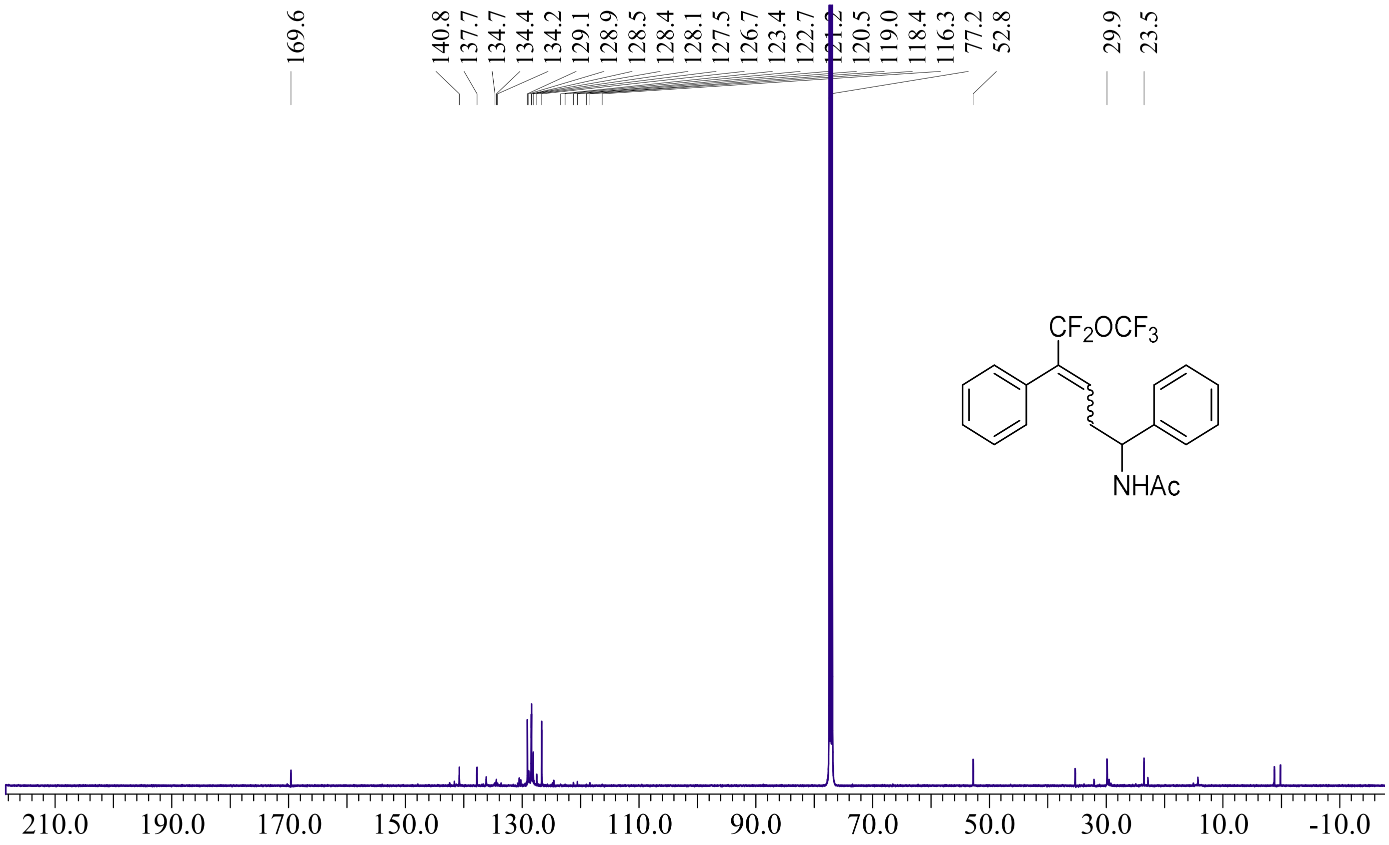

**$^{19}$F NMR** (282 MHz, $CDCl_3$) : **3v**

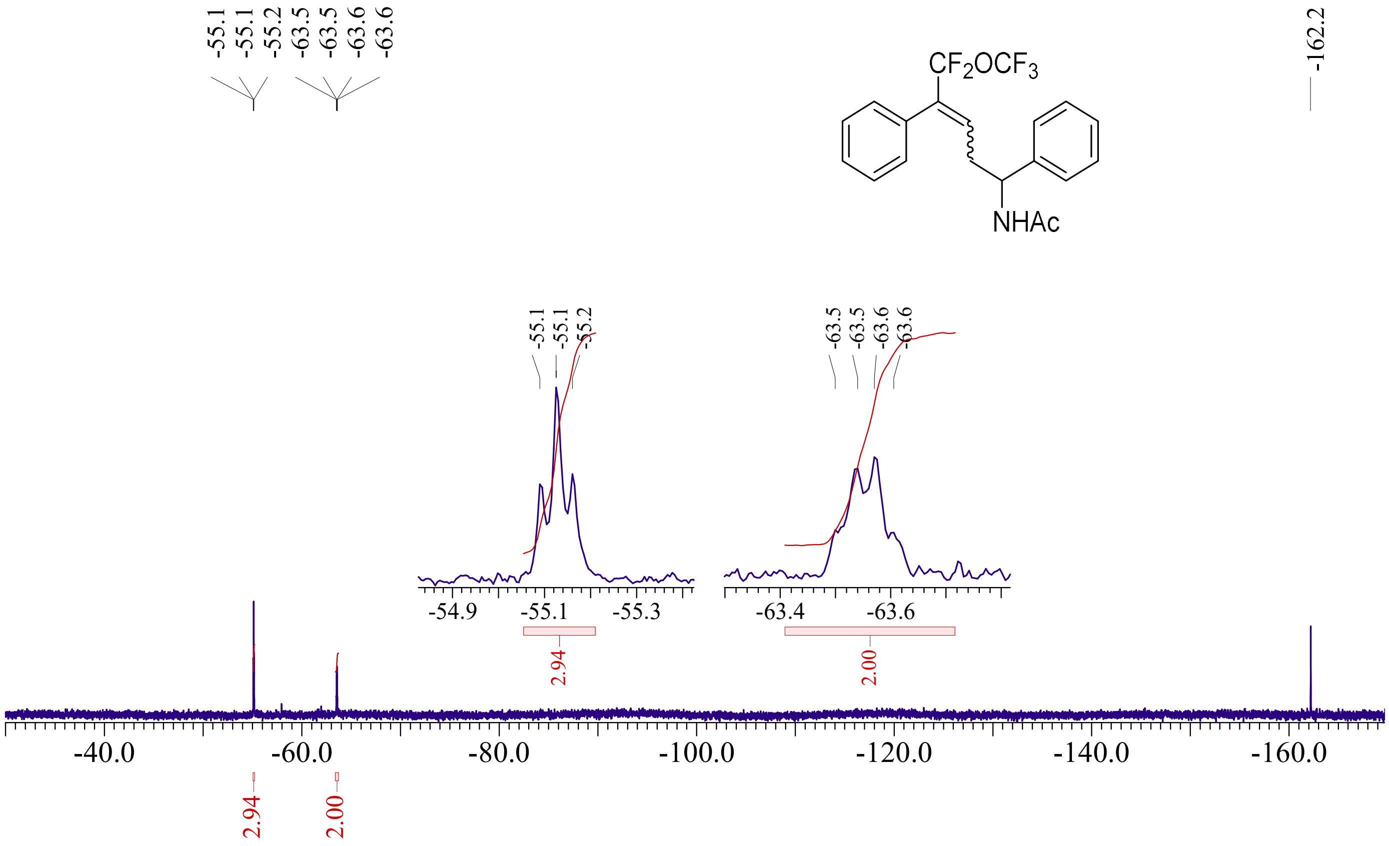


**$^{1}$H NMR** (500 MHz, $CDCl_3$) : **4a**

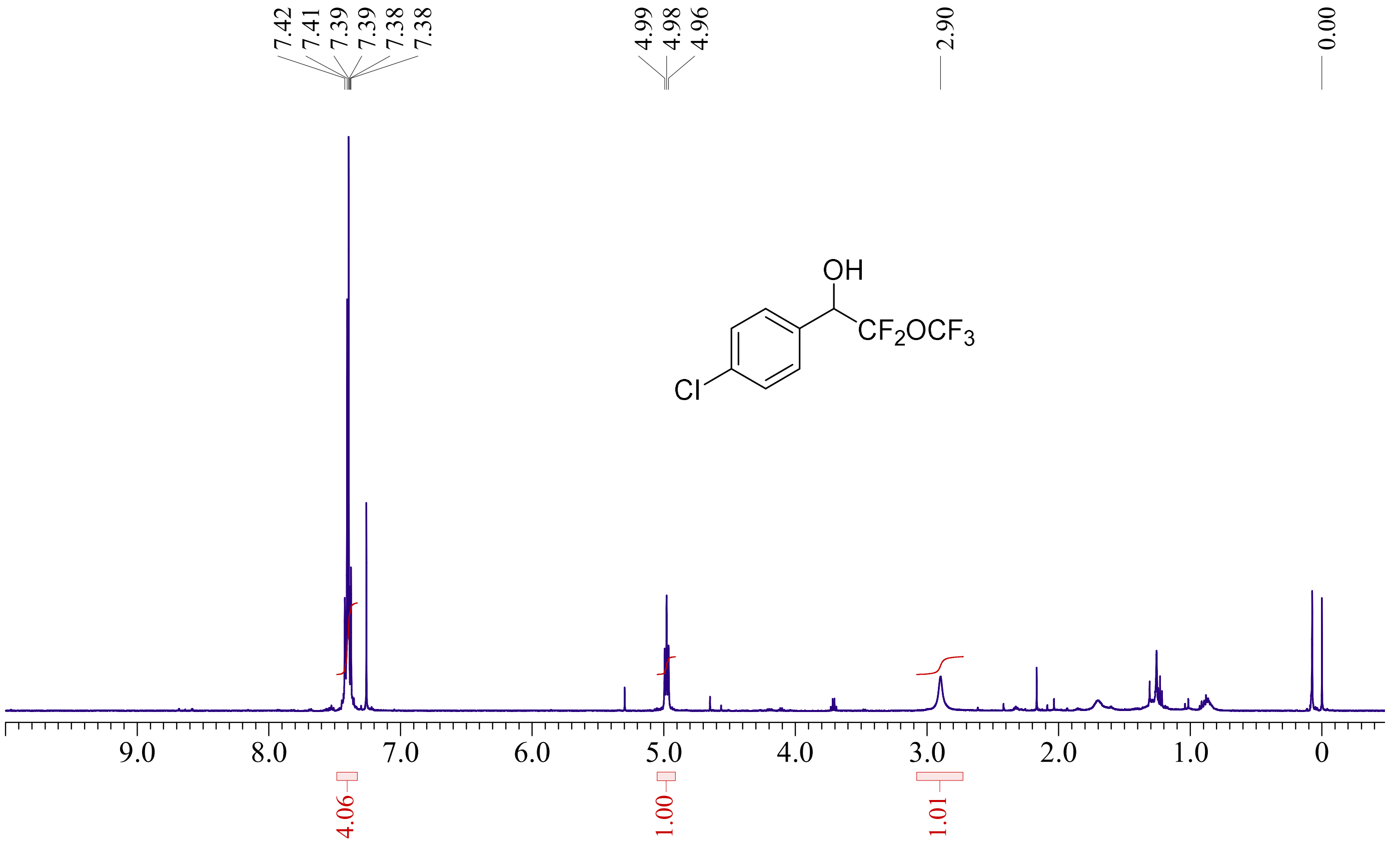

**$^{13}$C NMR** (126 MHz, $CDCl_3$) : **4a**

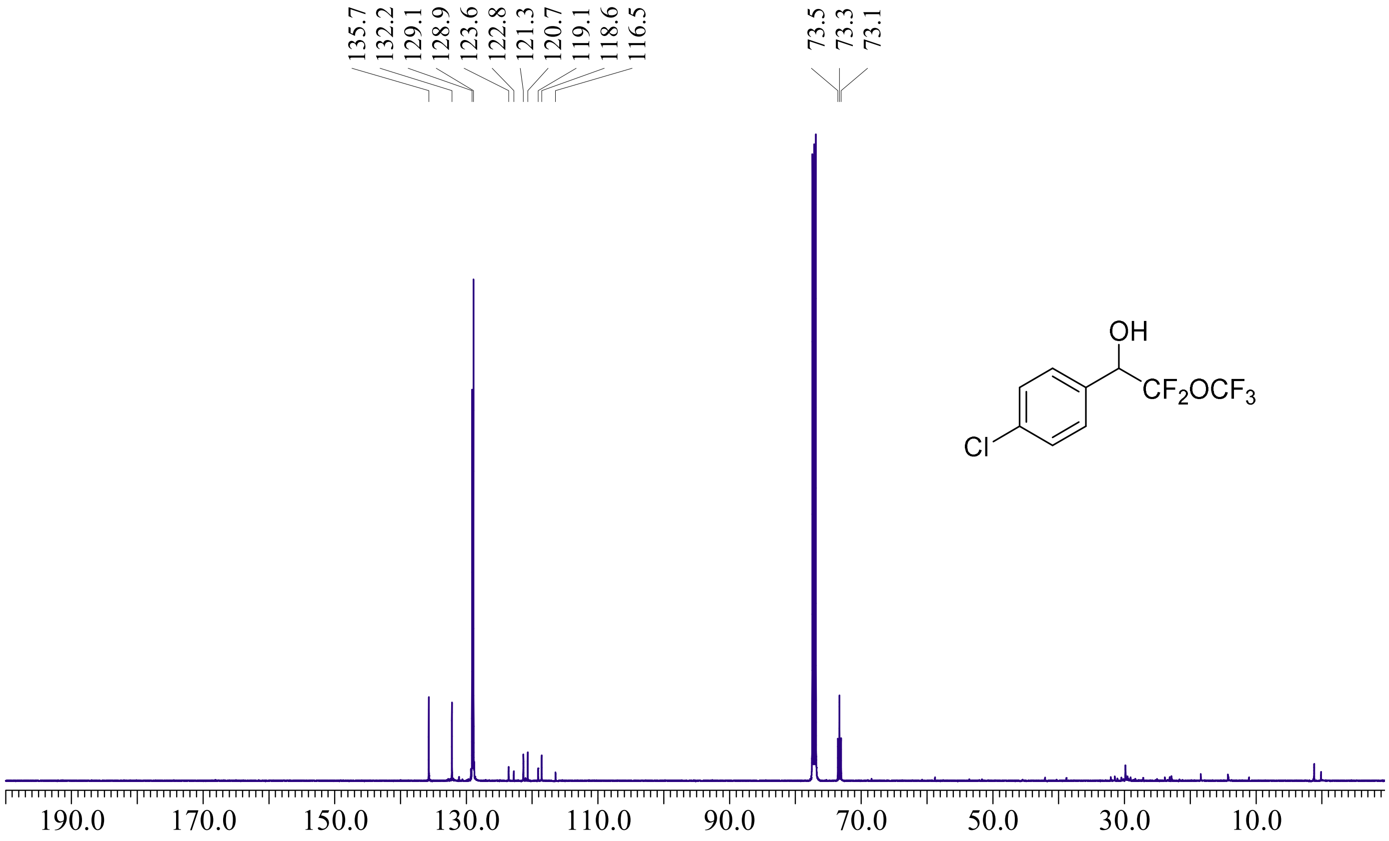


**$^{19}$F NMR** (282 MHz, $CDCl_3$) : **4a**

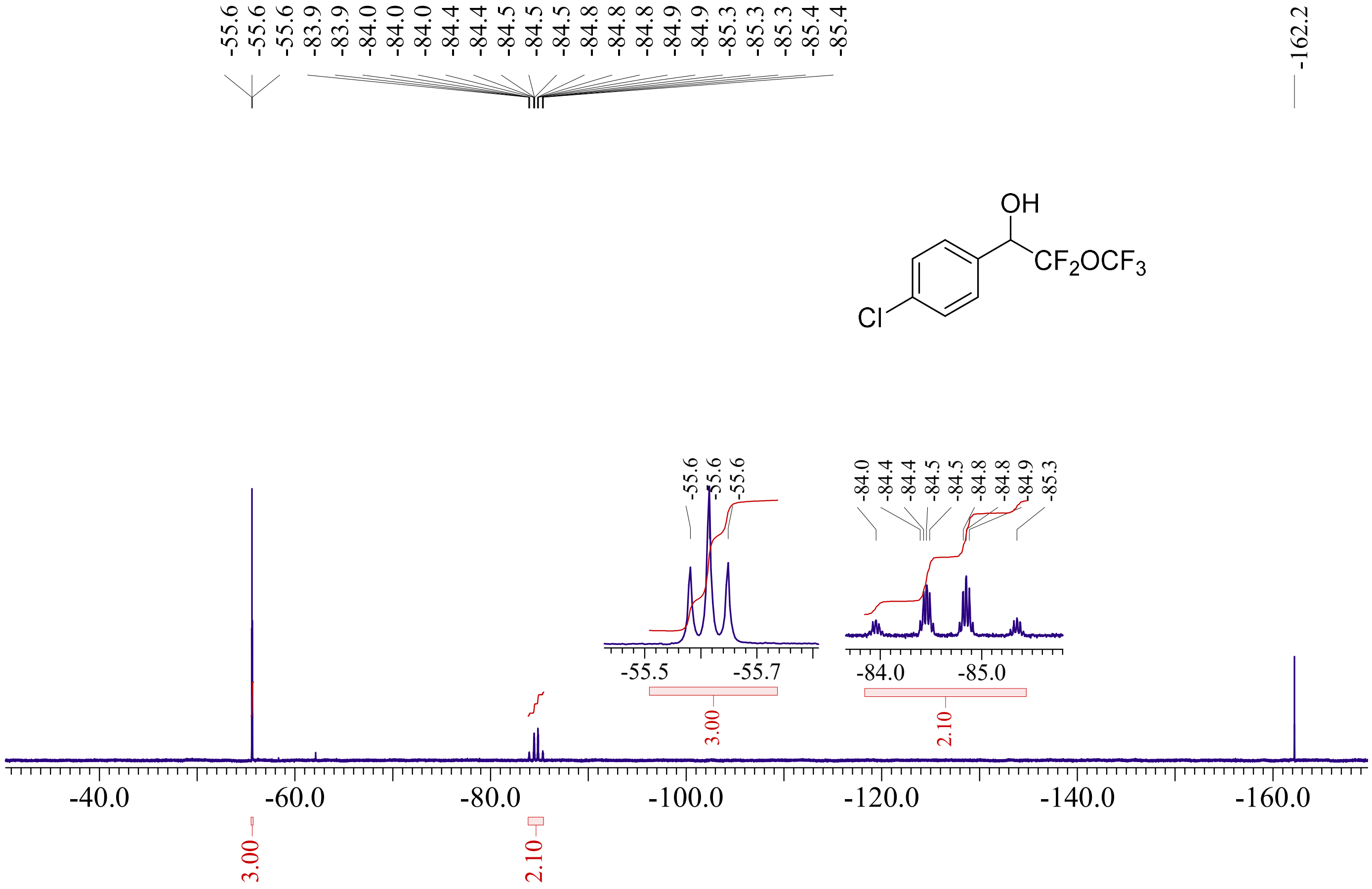

**$^{1}$H NMR** (500 MHz, $CDCl_3$) : **4g**

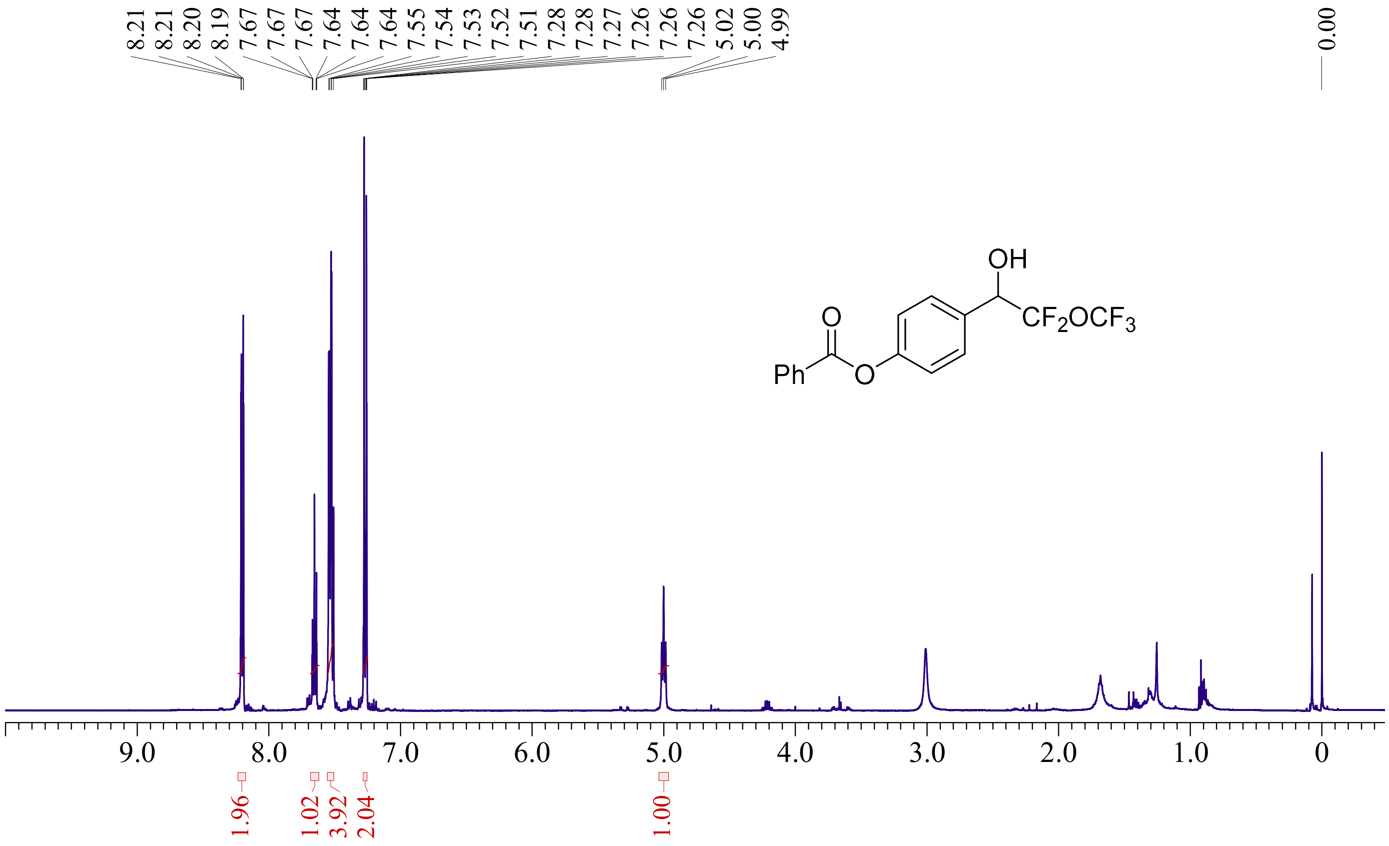

**$^{13}$C NMR** (126 MHz, $CDCl_3$) : **4g**

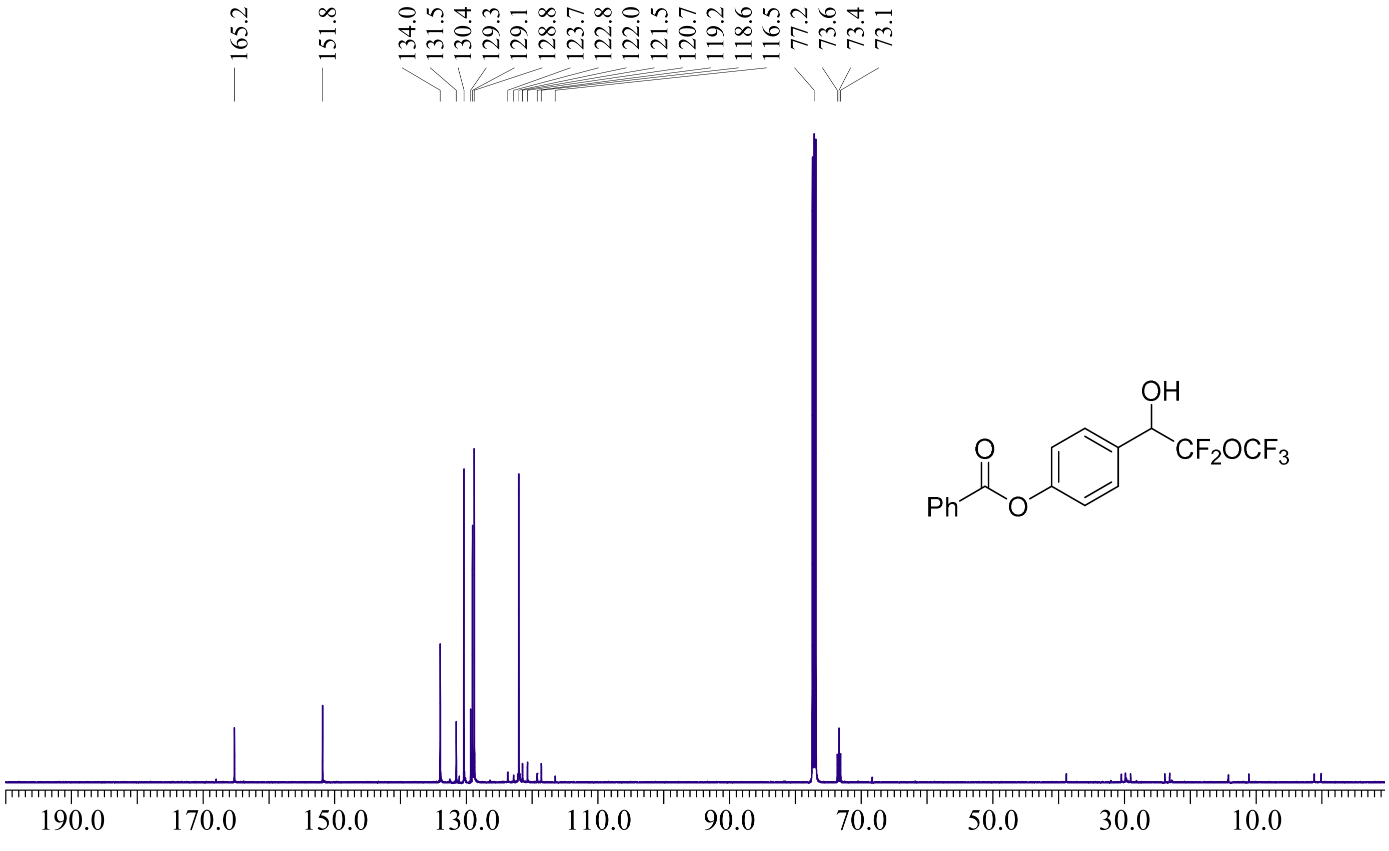

**$^{19}$F NMR** (282 MHz, $CDCl_3$) : **4g**

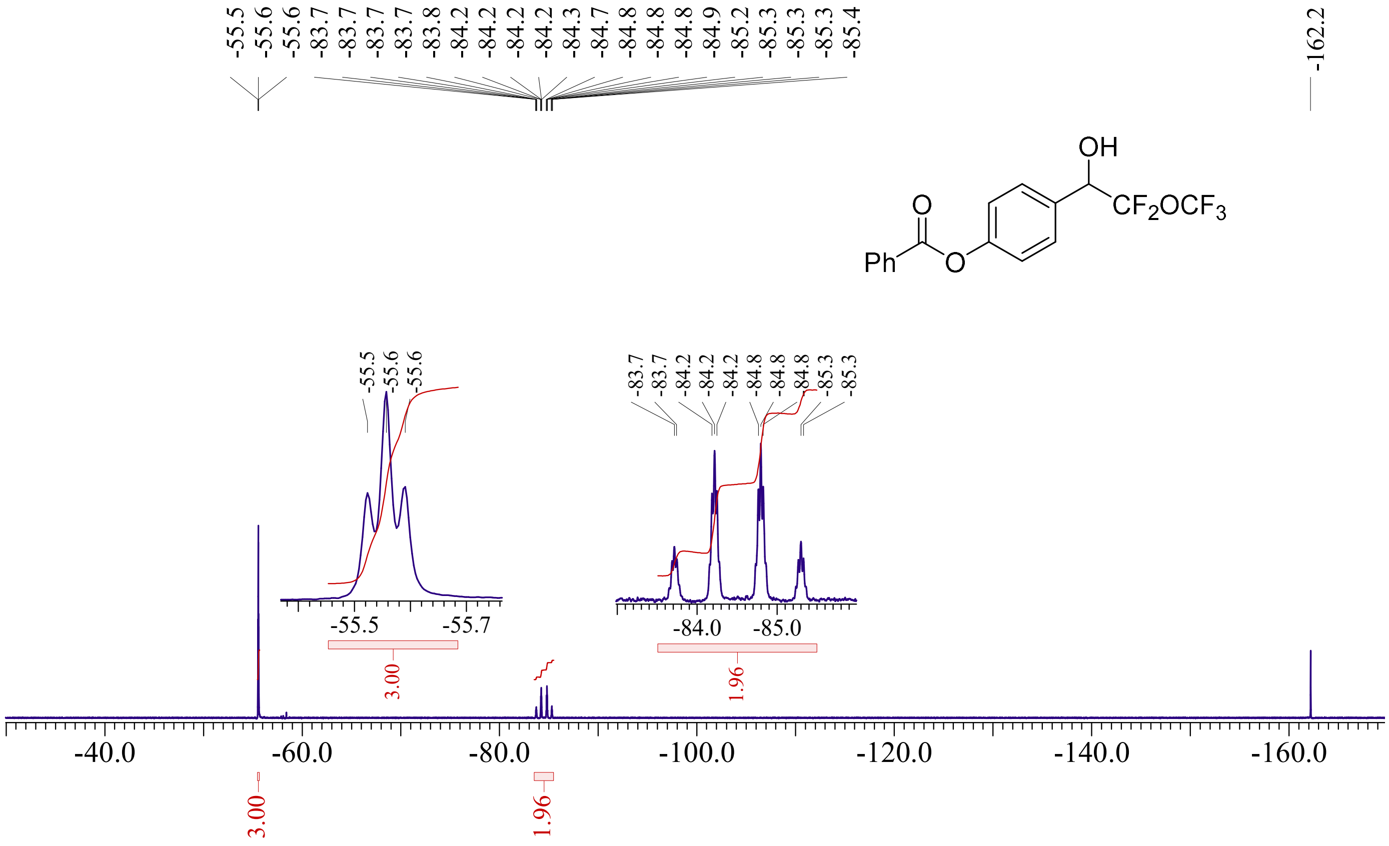

**$^{1}$H NMR** (500 MHz, $CDCl_3$) : **6a**

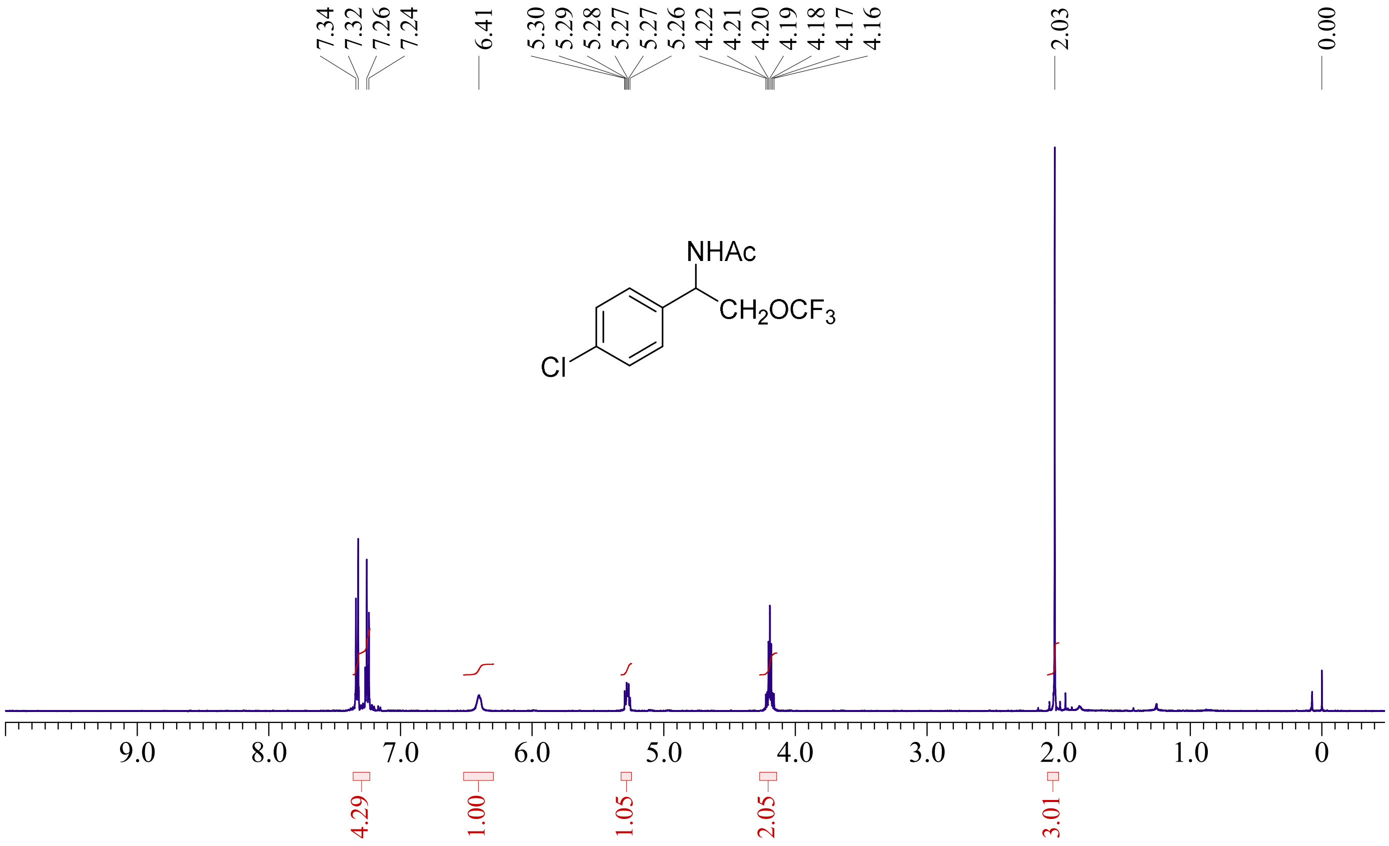

**$^{13}$C NMR** (126 MHz, $CDCl_3$) : **6a**

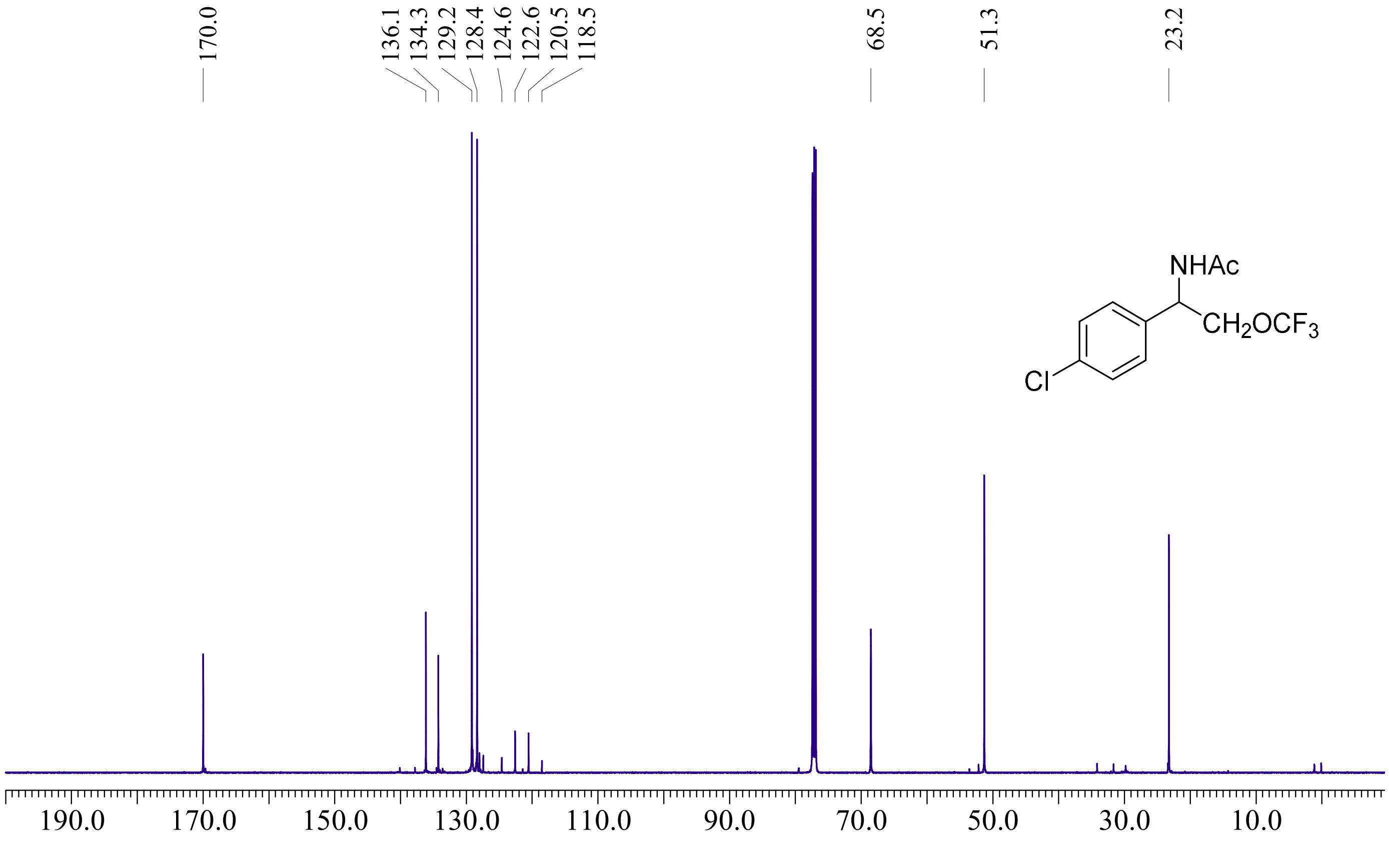


**$^{19}$F NMR** (282 MHz, $CDCl_3$) : **6a**

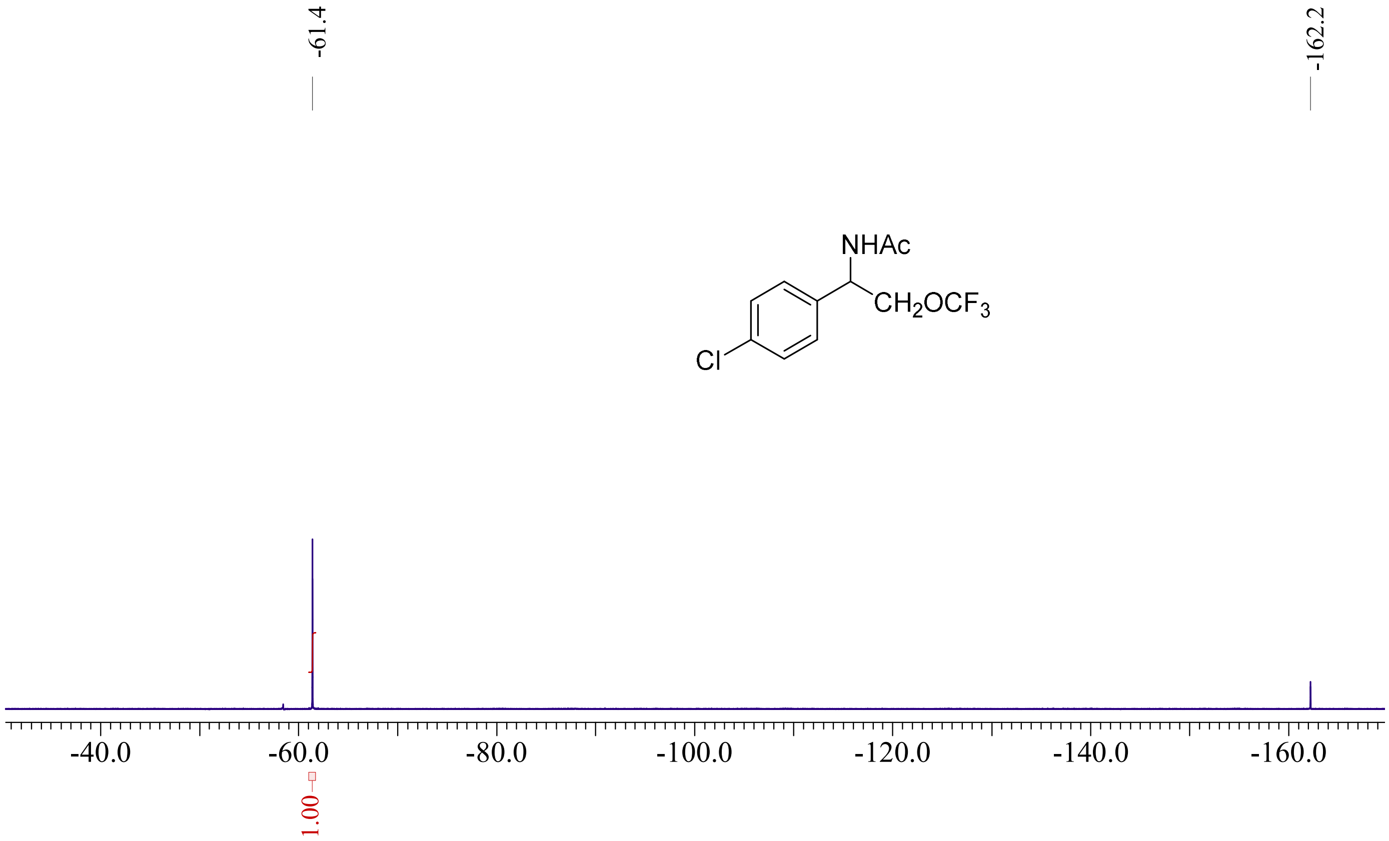

**$^{1}$H NMR** (500 MHz, CDCl3) : **6d**

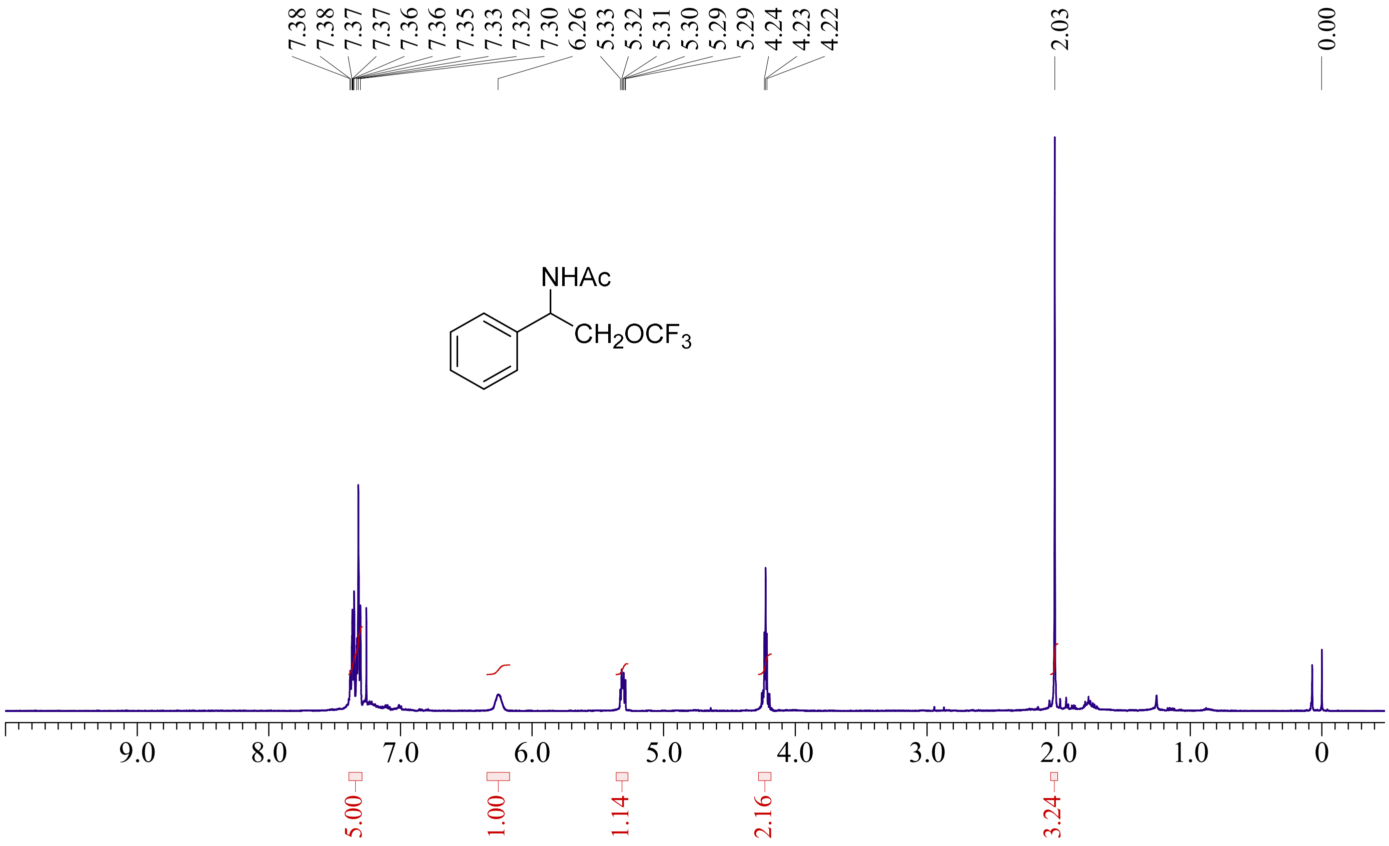


**$^{13}$C NMR** (126 MHz, CDCl3) : **6d**

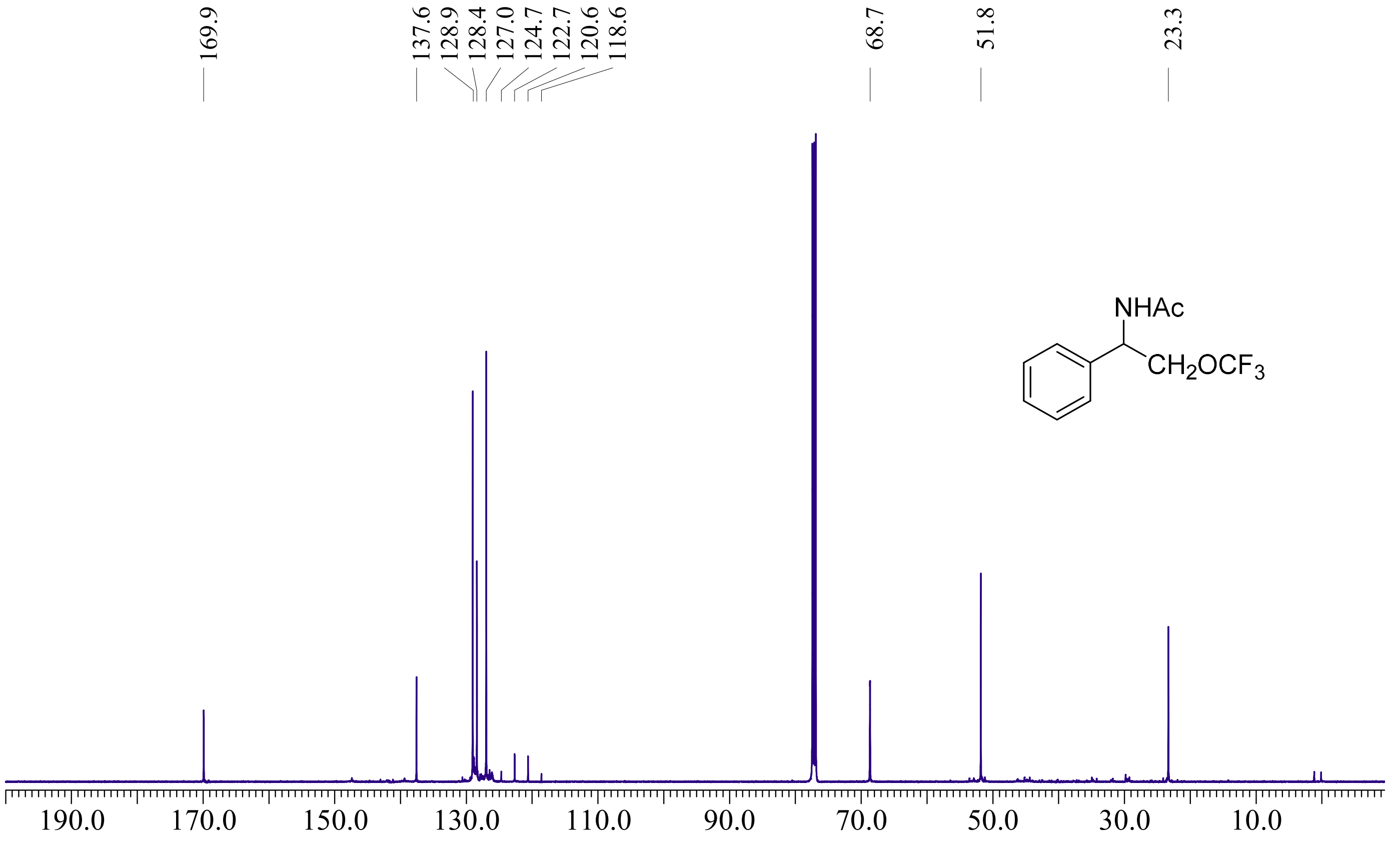

**$^{19}$F NMR** (282 MHz, $CDCl_3$) : **6d**

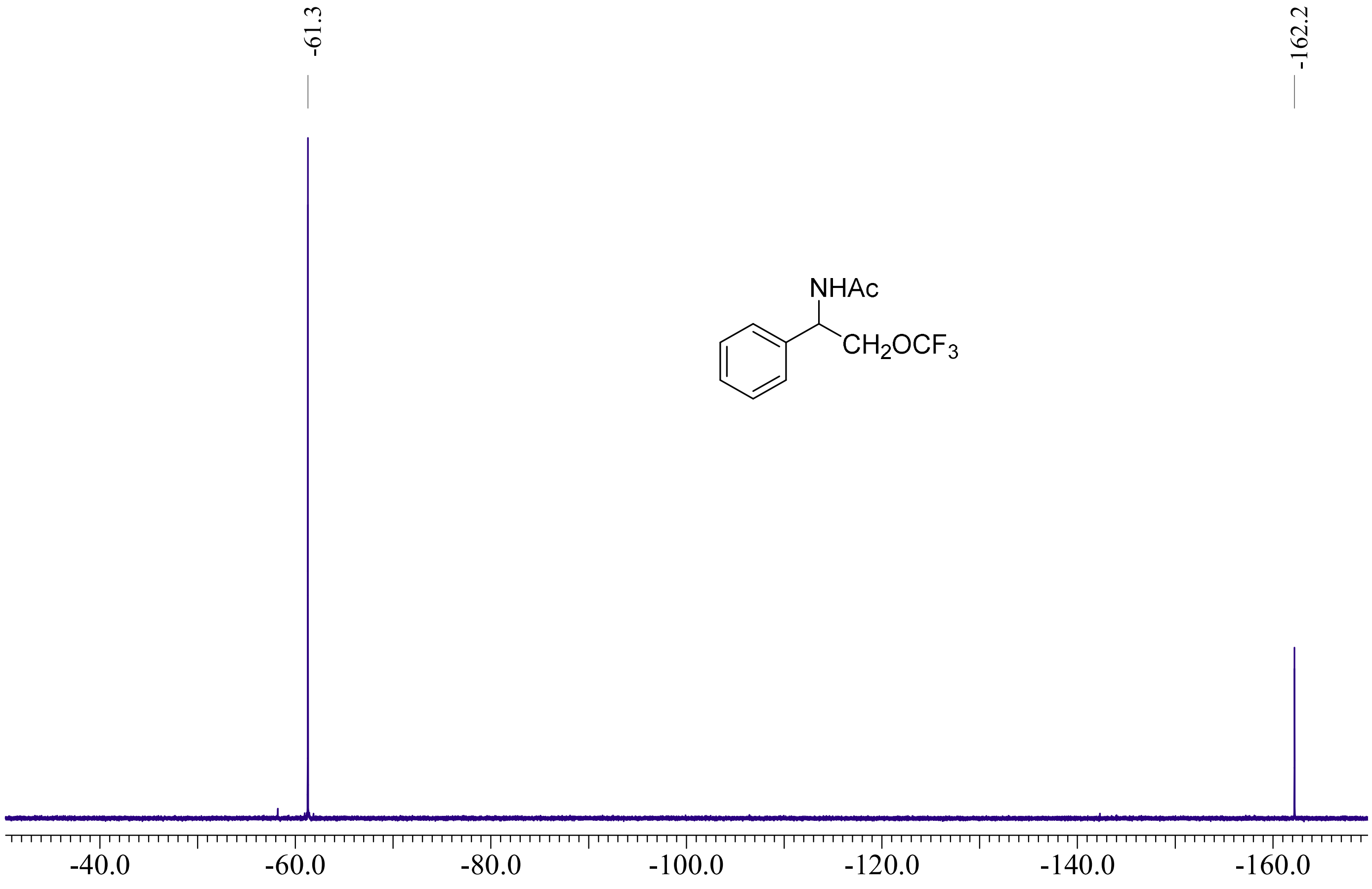


**$^{1}$H NMR** (500 MHz, $CDCl_3$) : **6f**

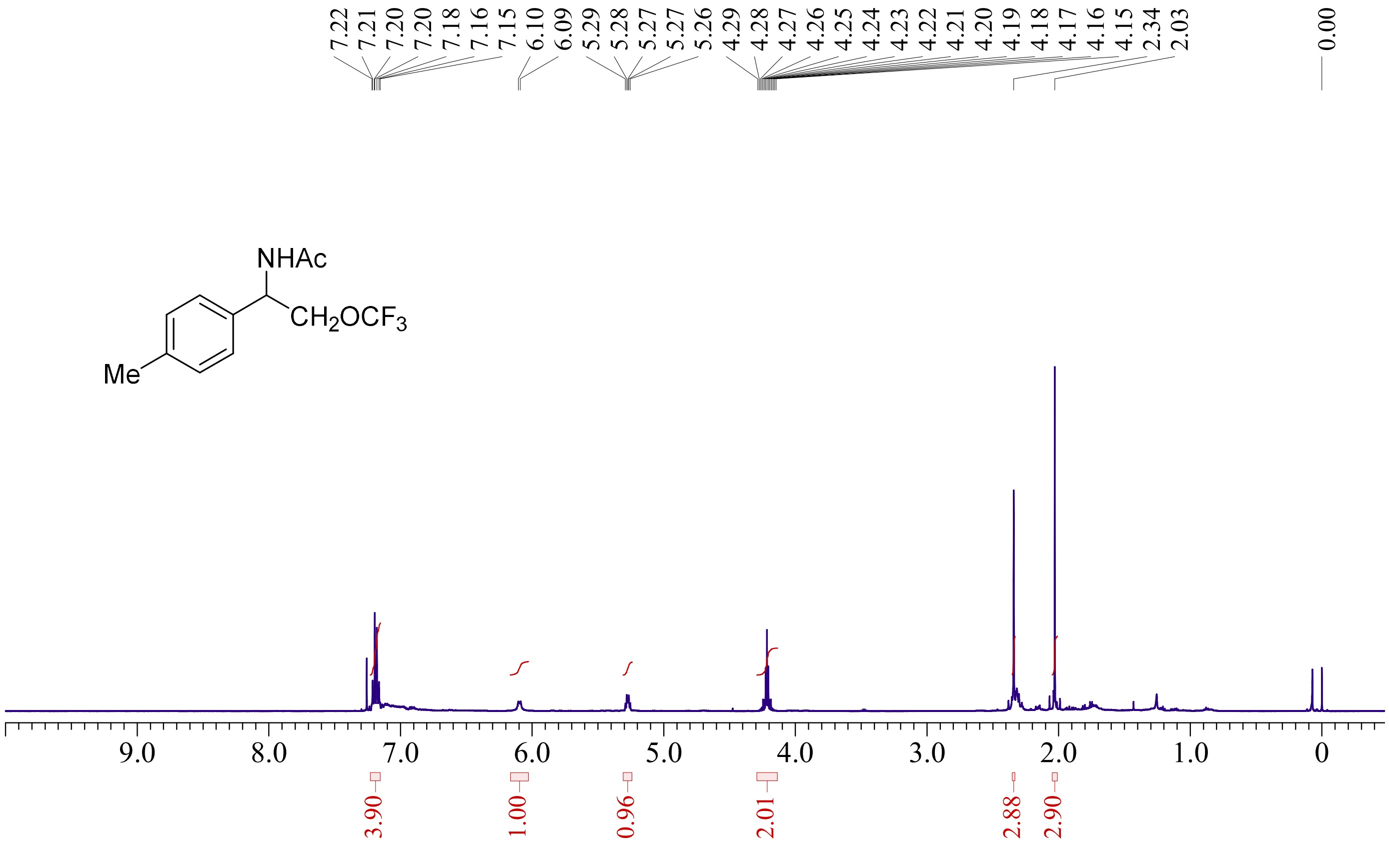

**$^{13}$C NMR** (126 MHz, $CDCl_3$) : **6f**

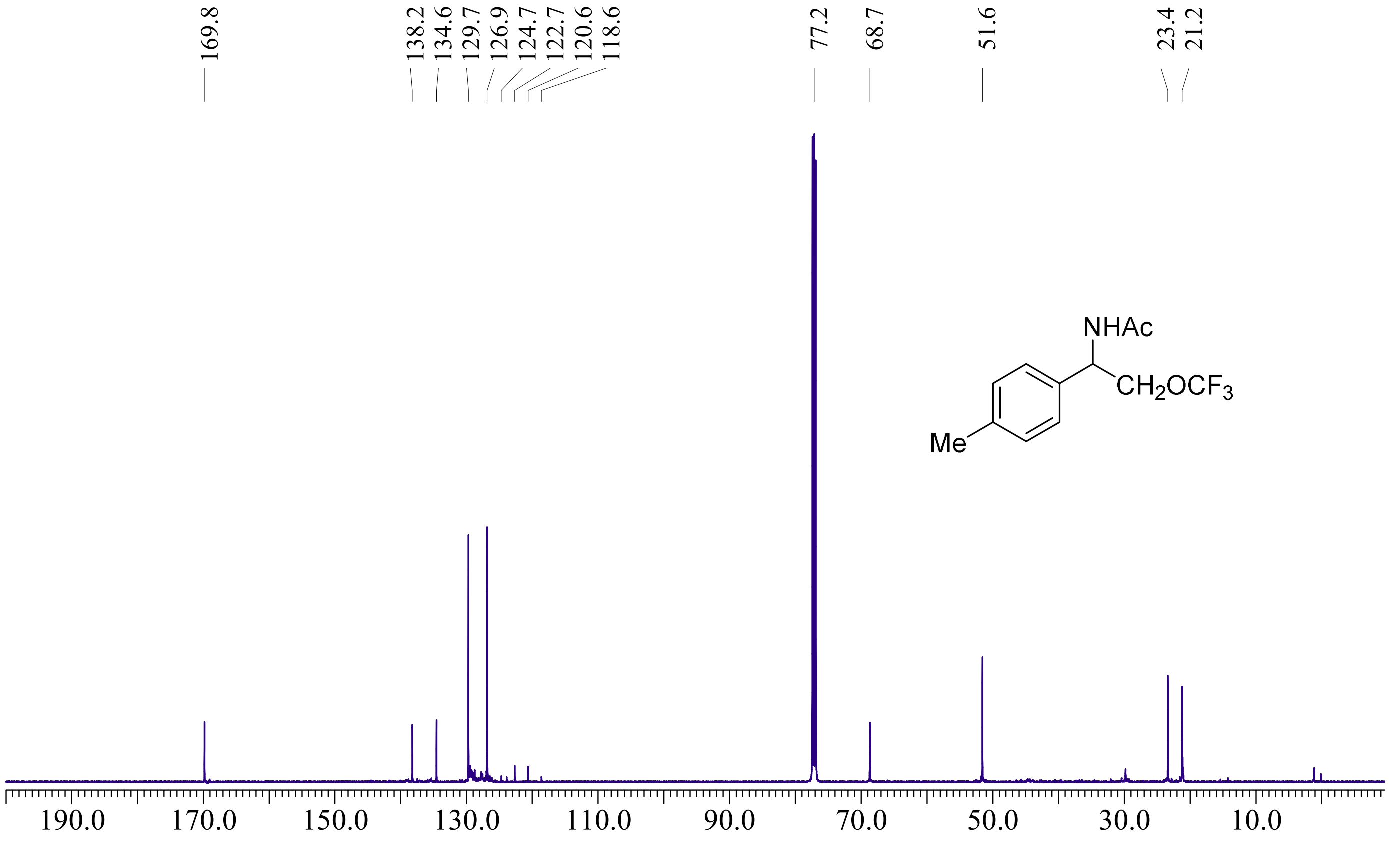


**$^{19}$F NMR** (282 MHz, $CDCl_3$) : **6f**

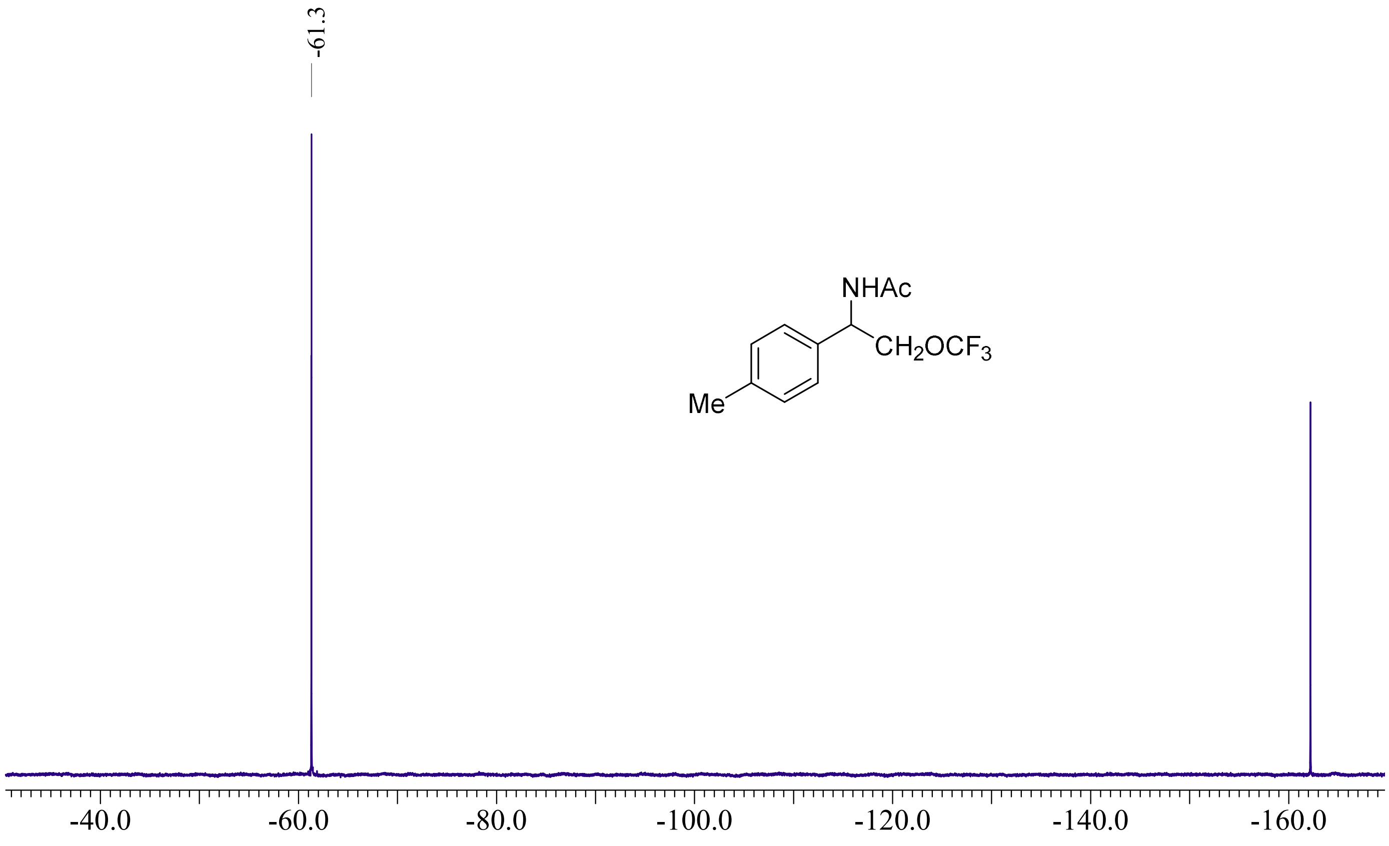

**¹H NMR** (500 MHz, $CDCl_3$) : **6g**

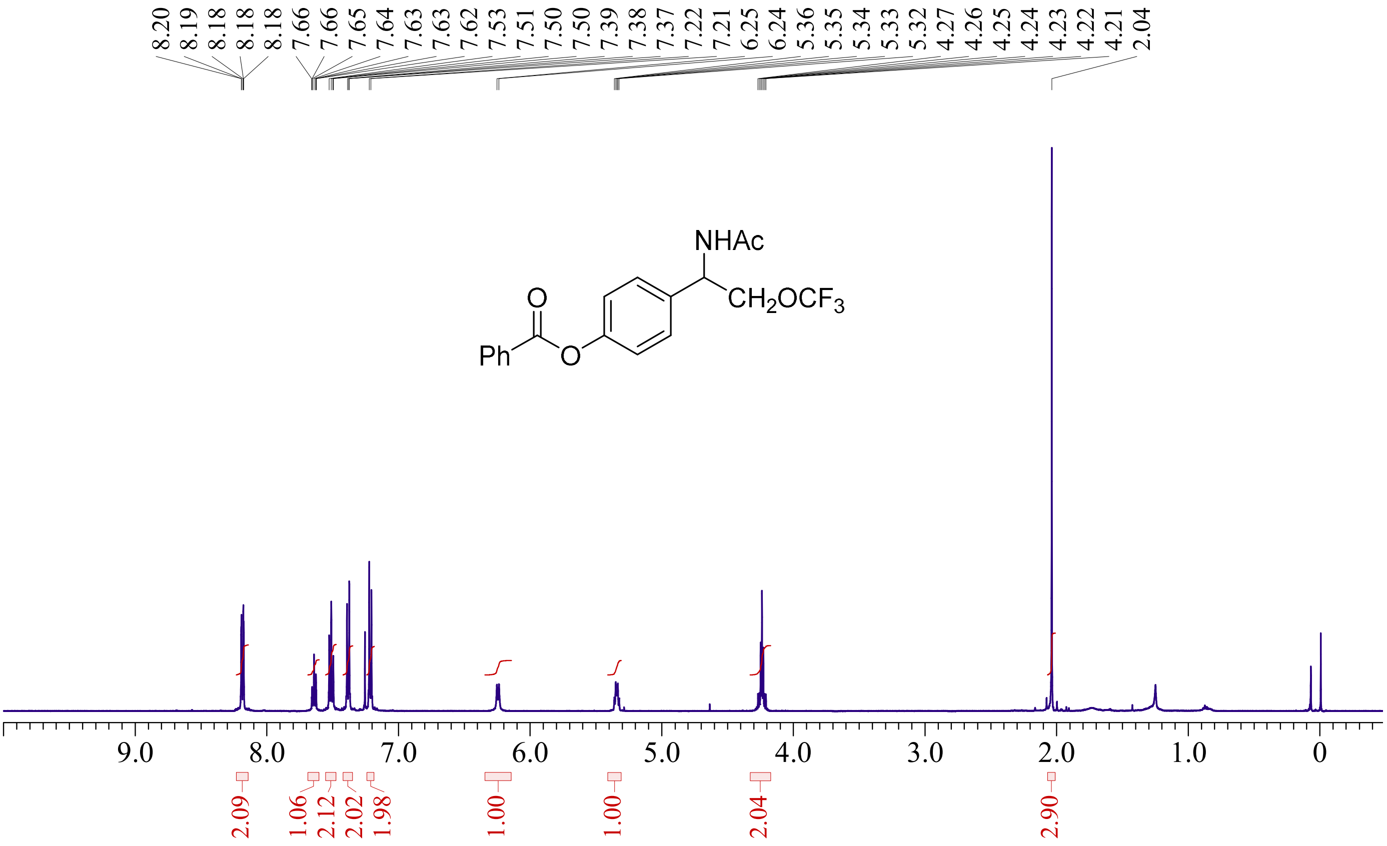


**¹³C NMR** (126 MHz, $CDCl_3$) : **6g**

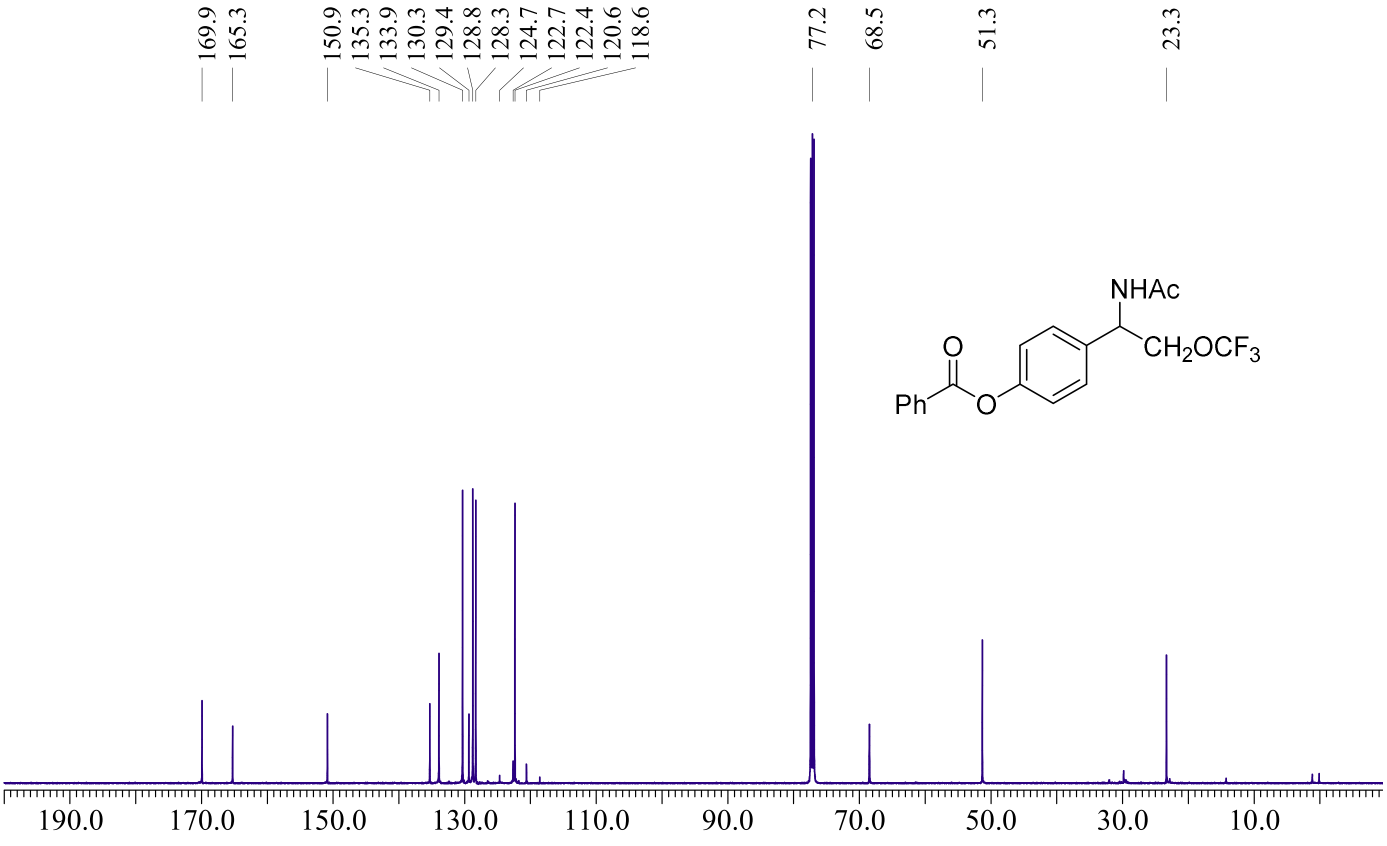

**$^{19}$F NMR** (282 MHz, $CDCl_3$) : **6g**

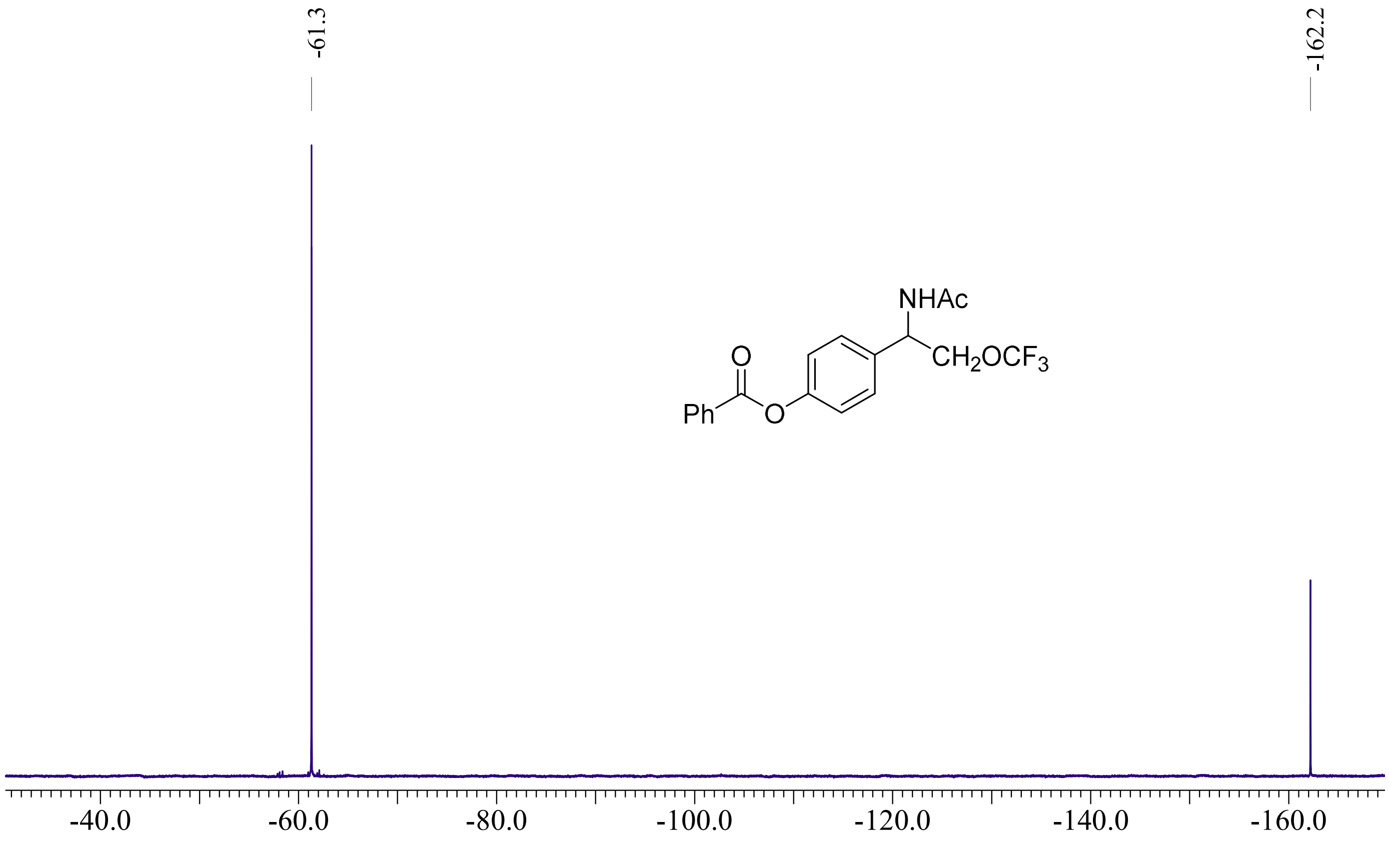


**$^{1}$H NMR** (500 MHz, $CDCl_3$) : **6j**

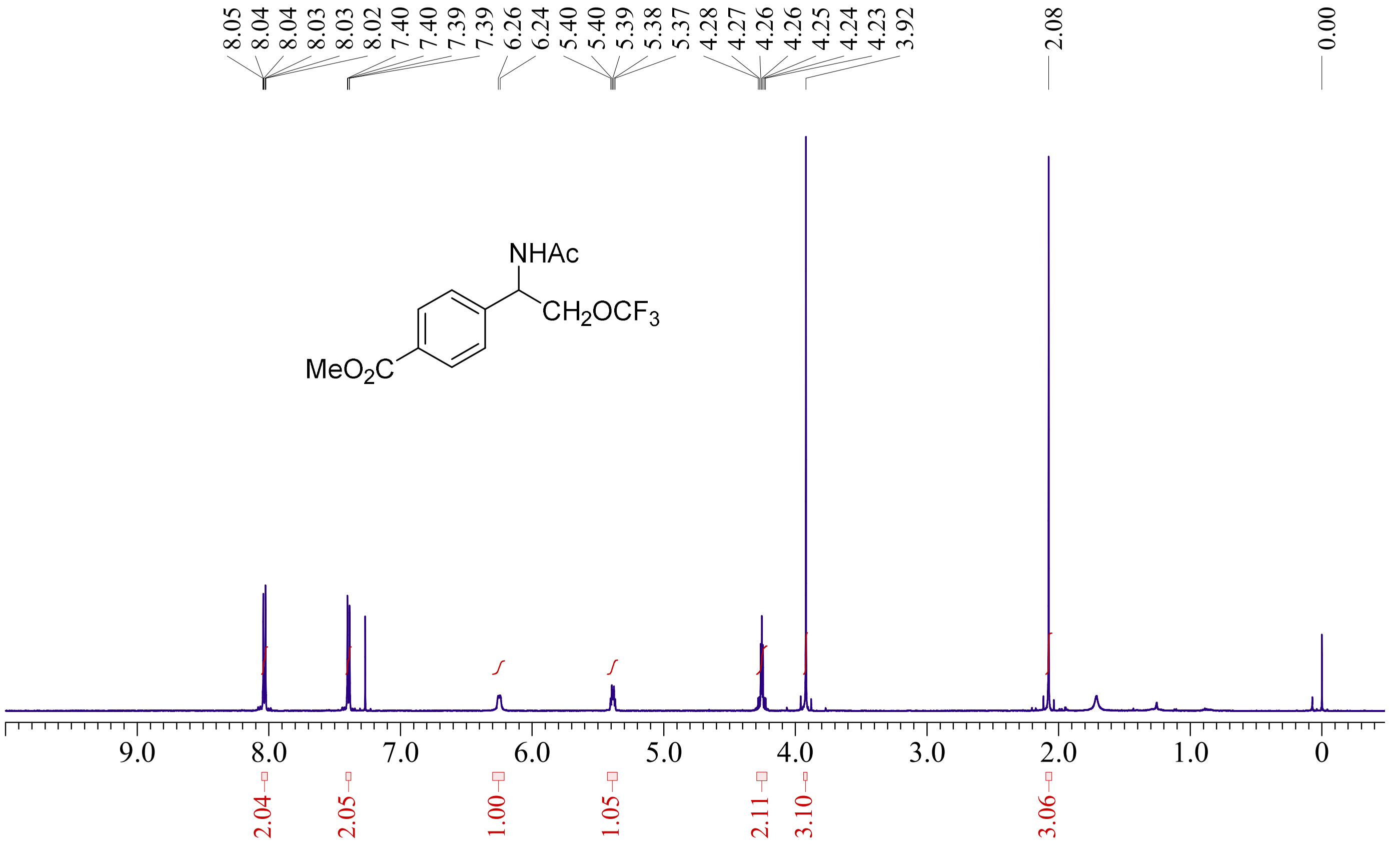

**$^{13}$C NMR** (126 MHz, $CDCl_3$) : **6j**

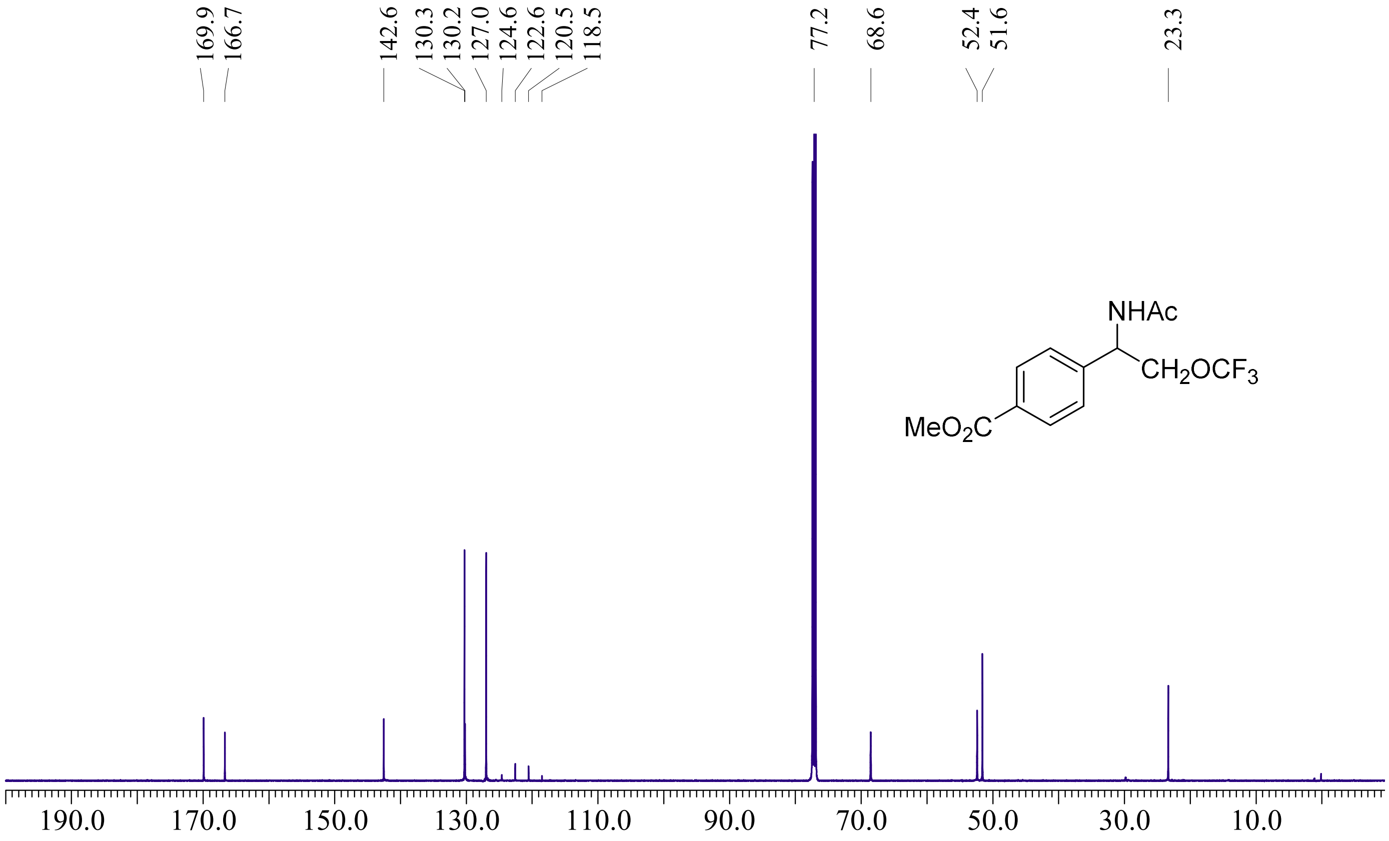


**$^{19}$F NMR** (282 MHz, $CDCl_3$) : **6j**

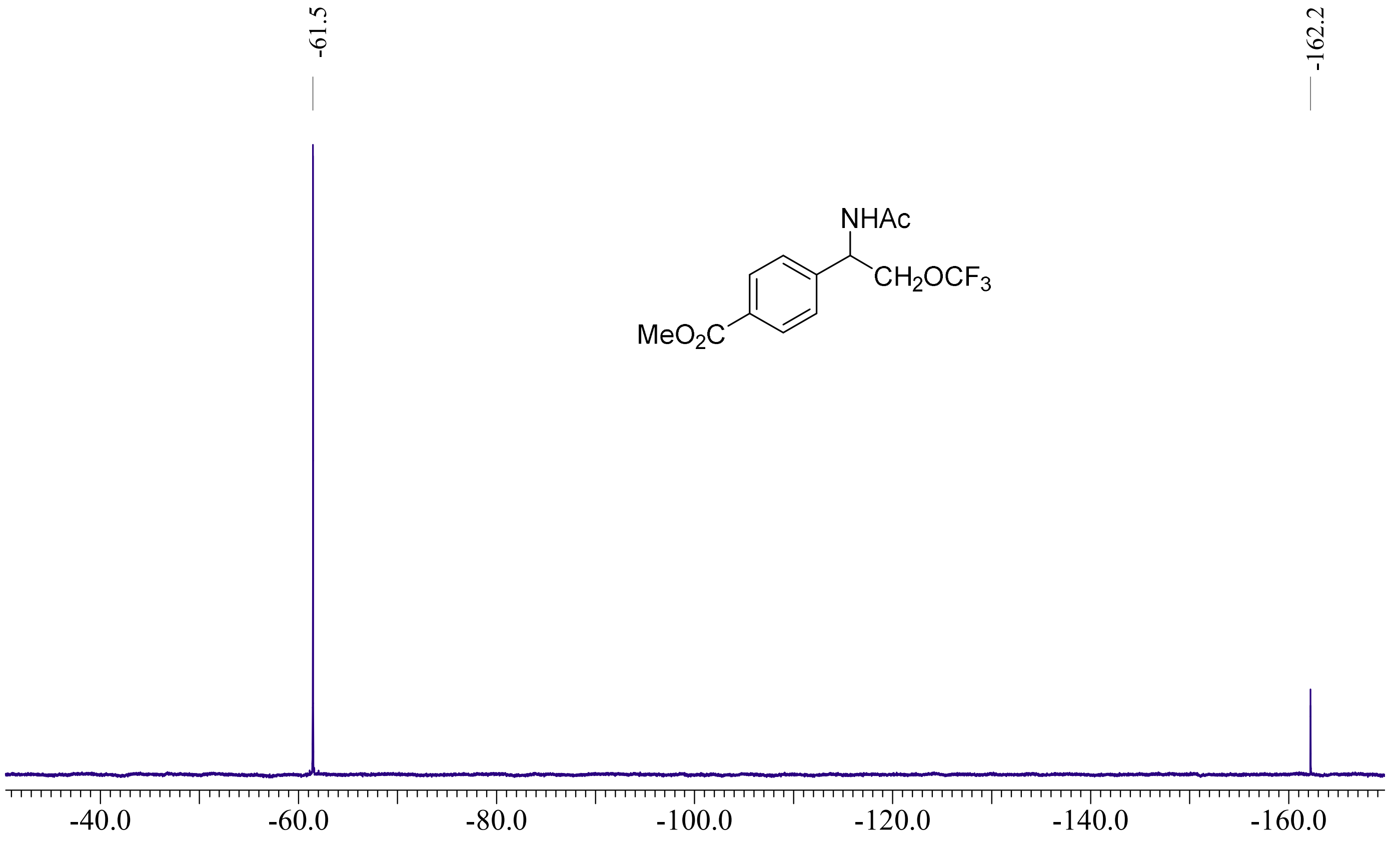

**$^{1}$H NMR** (500 MHz, $CDCl_3$) : **6k**

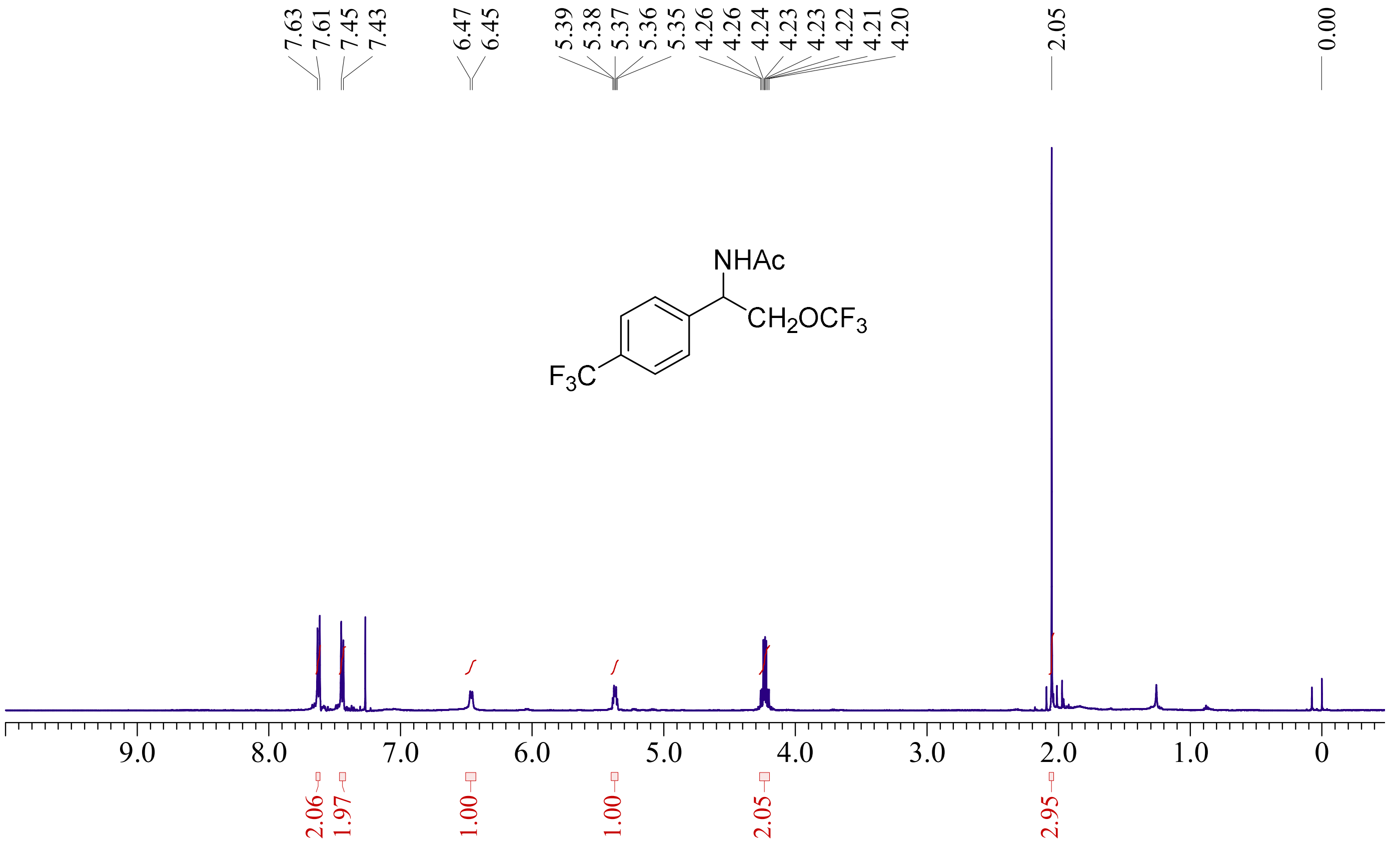


**$^{13}$C NMR** (126 MHz, $CDCl_3$) : **6k**

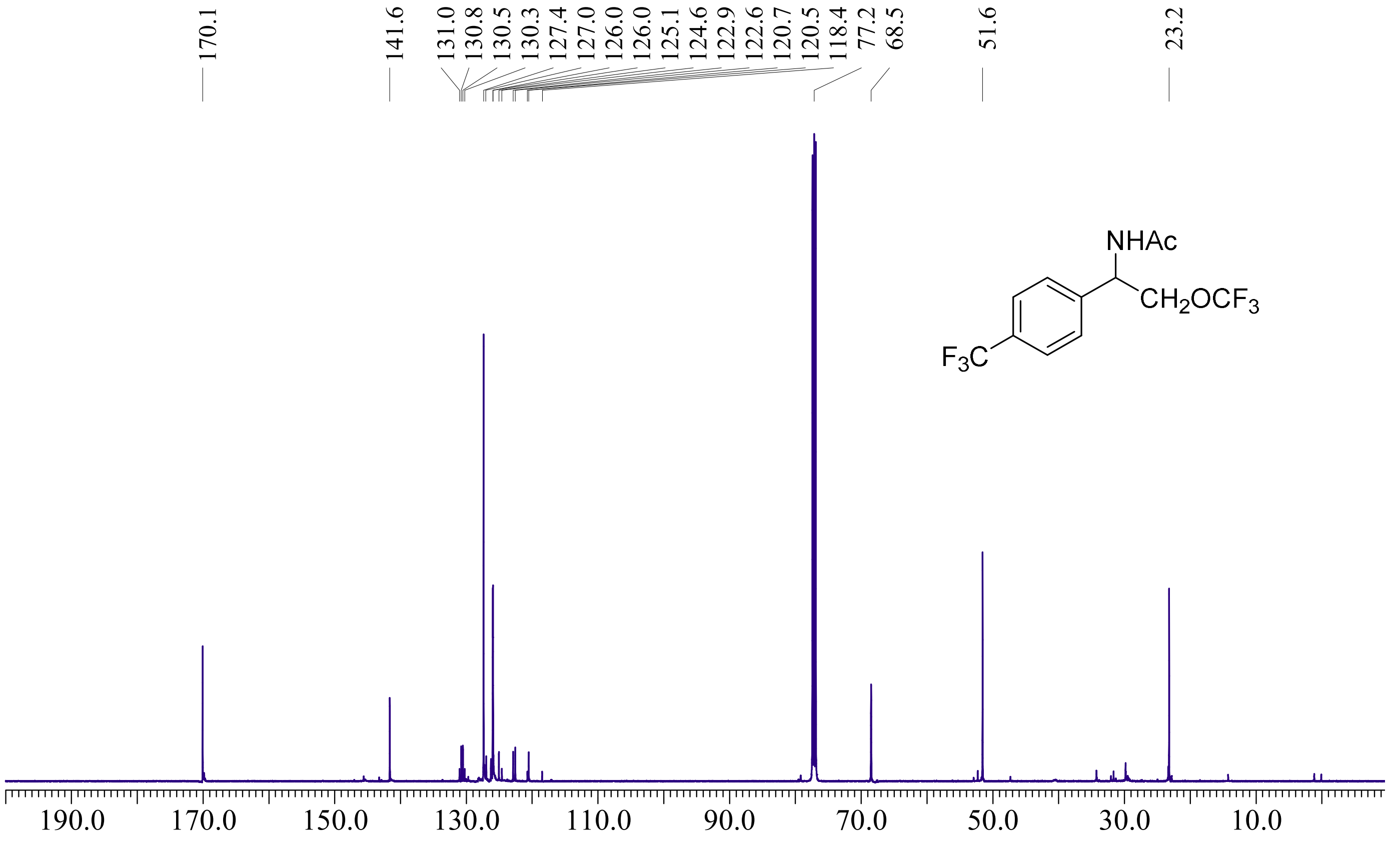

**$^{19}$F NMR** (282 MHz, $CDCl_3$) : **6k**

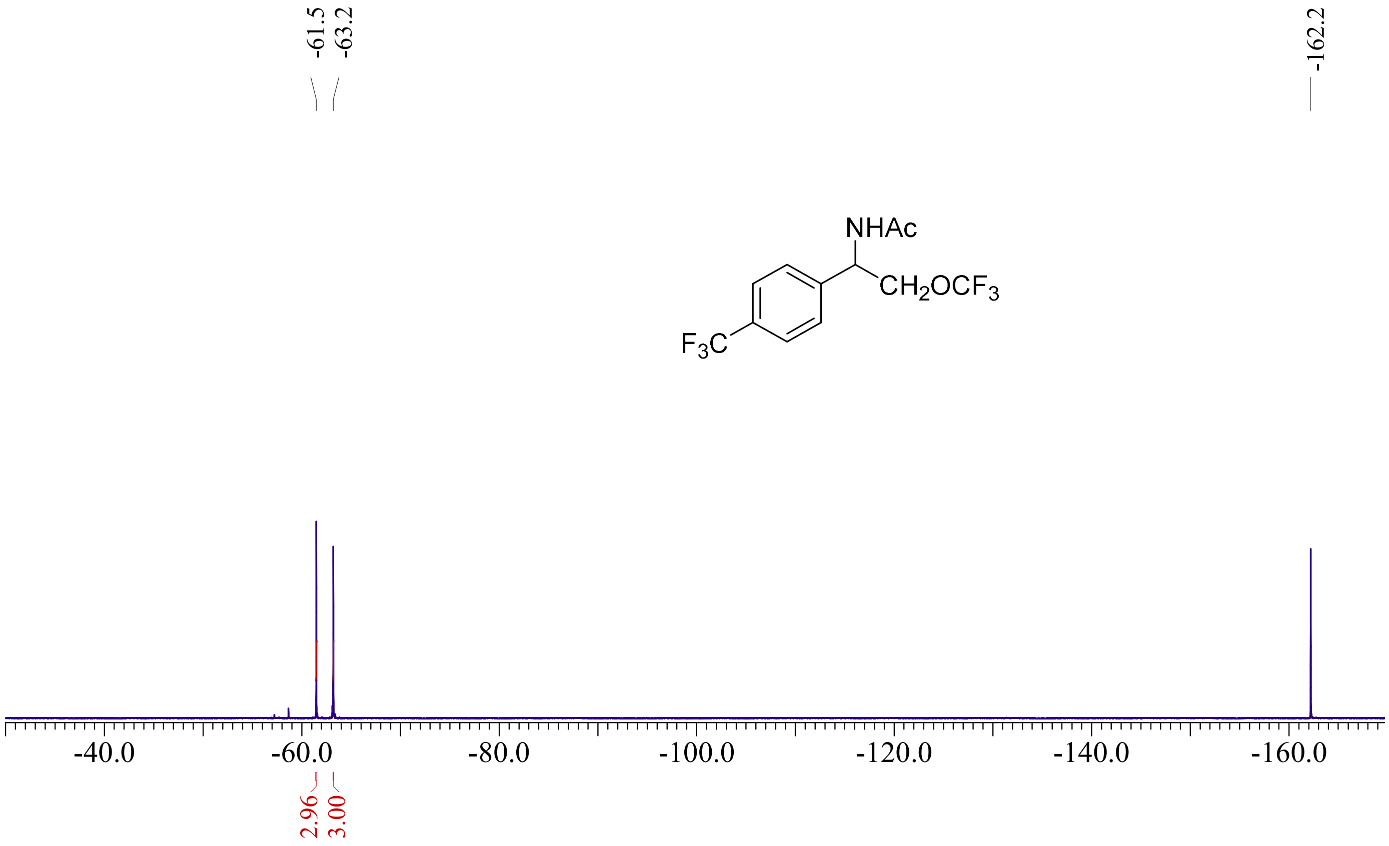


**$^{1}$H NMR** (500 MHz, $CDCl_3$) : **7j**

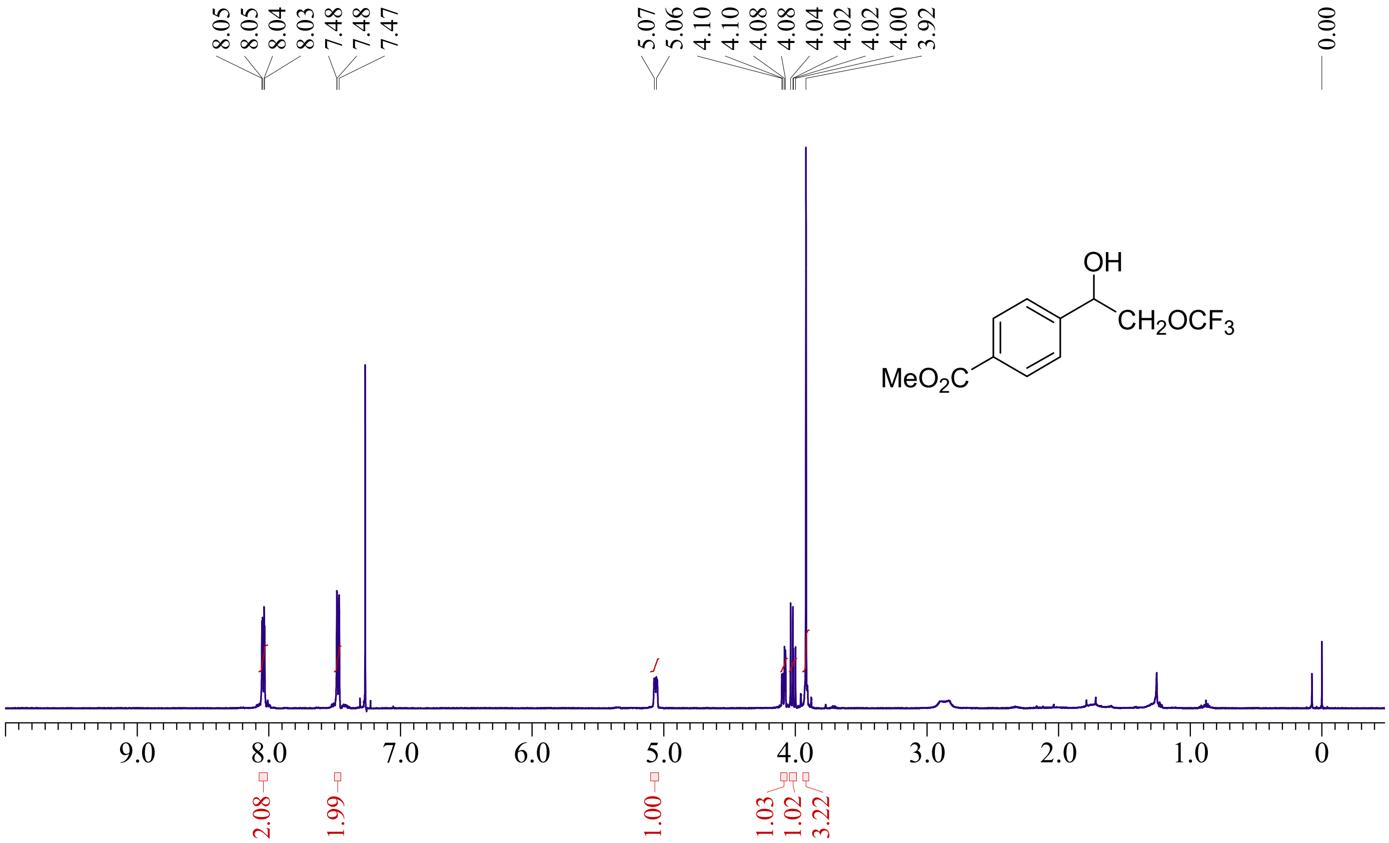

**$^{13}$C NMR** (126 MHz, $CDCl_3$) : **7j**

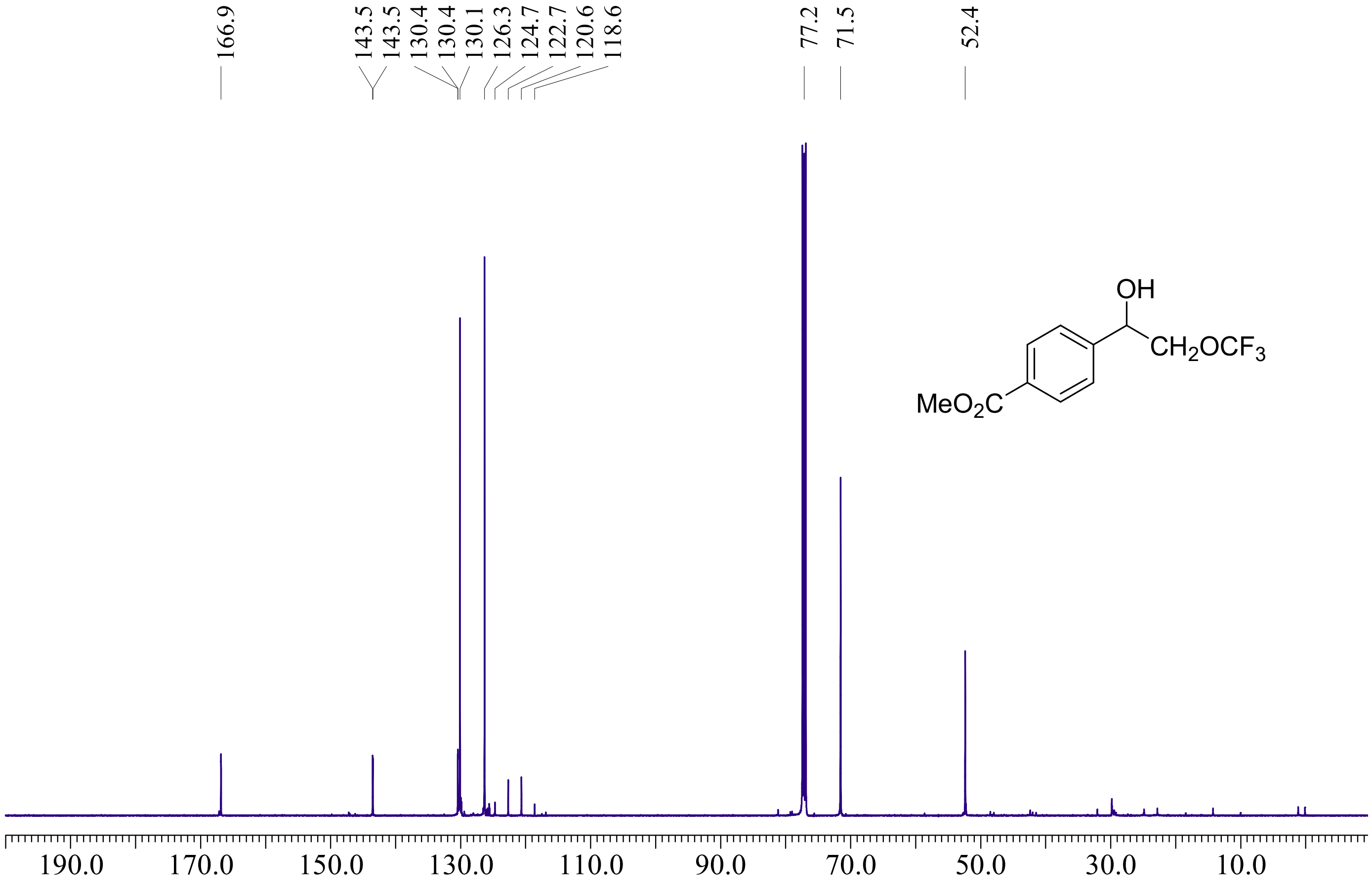


**$^{19}$F NMR** (282 MHz, $CDCl_3$) : **7j**

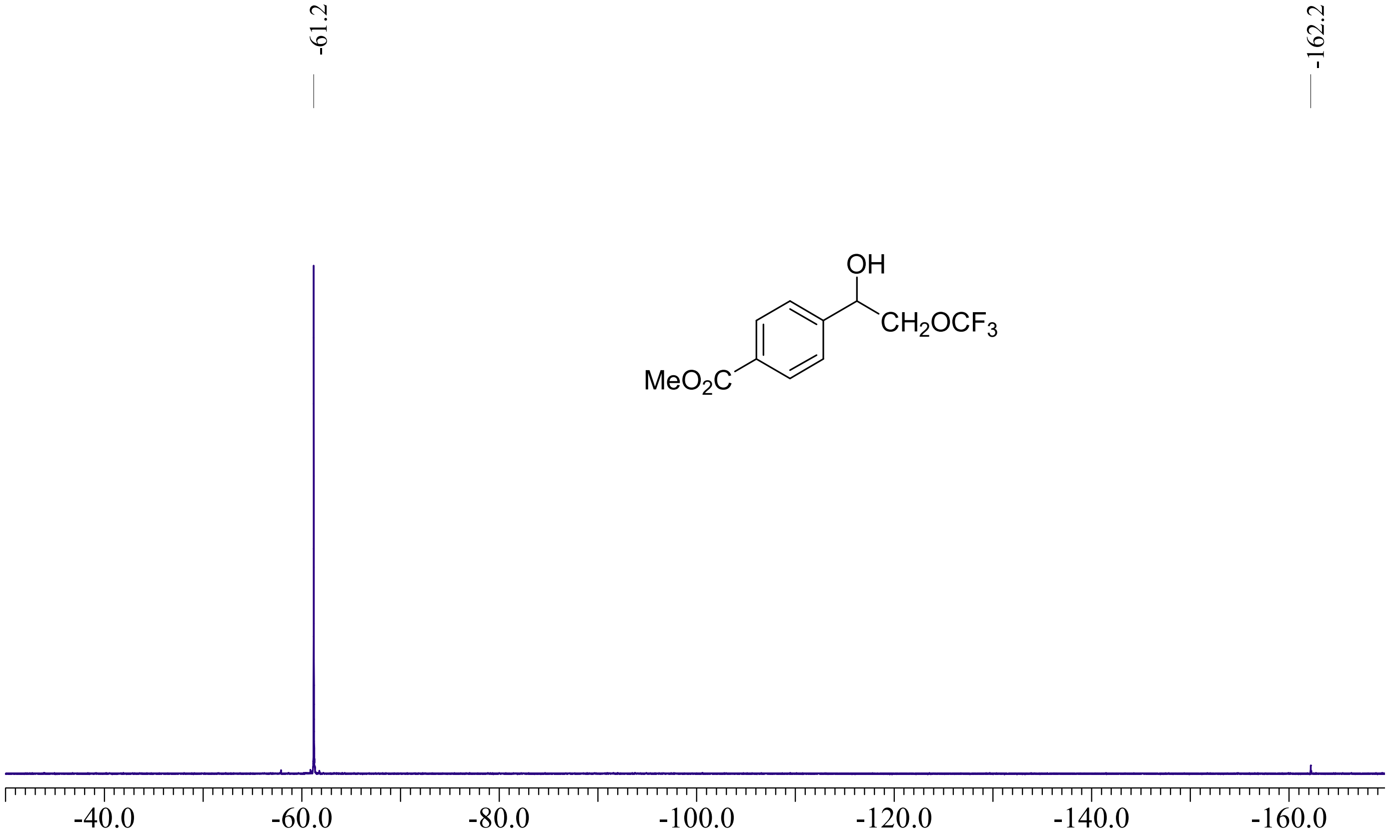

**¹H NMR** (500 MHz, $CDCl_3$) : **8**

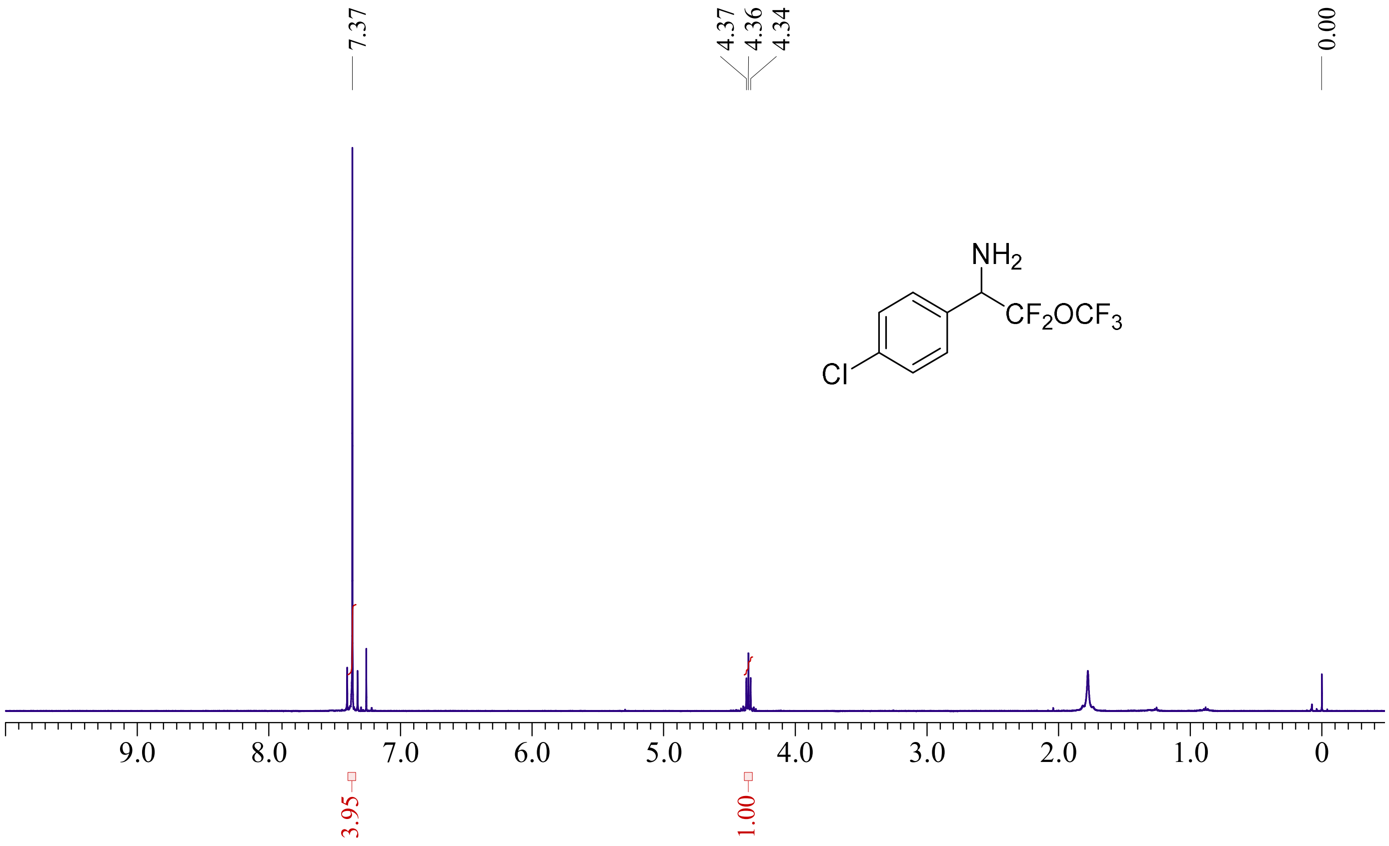


**¹³C NMR** (126 MHz, $CDCl_3$) : **8**

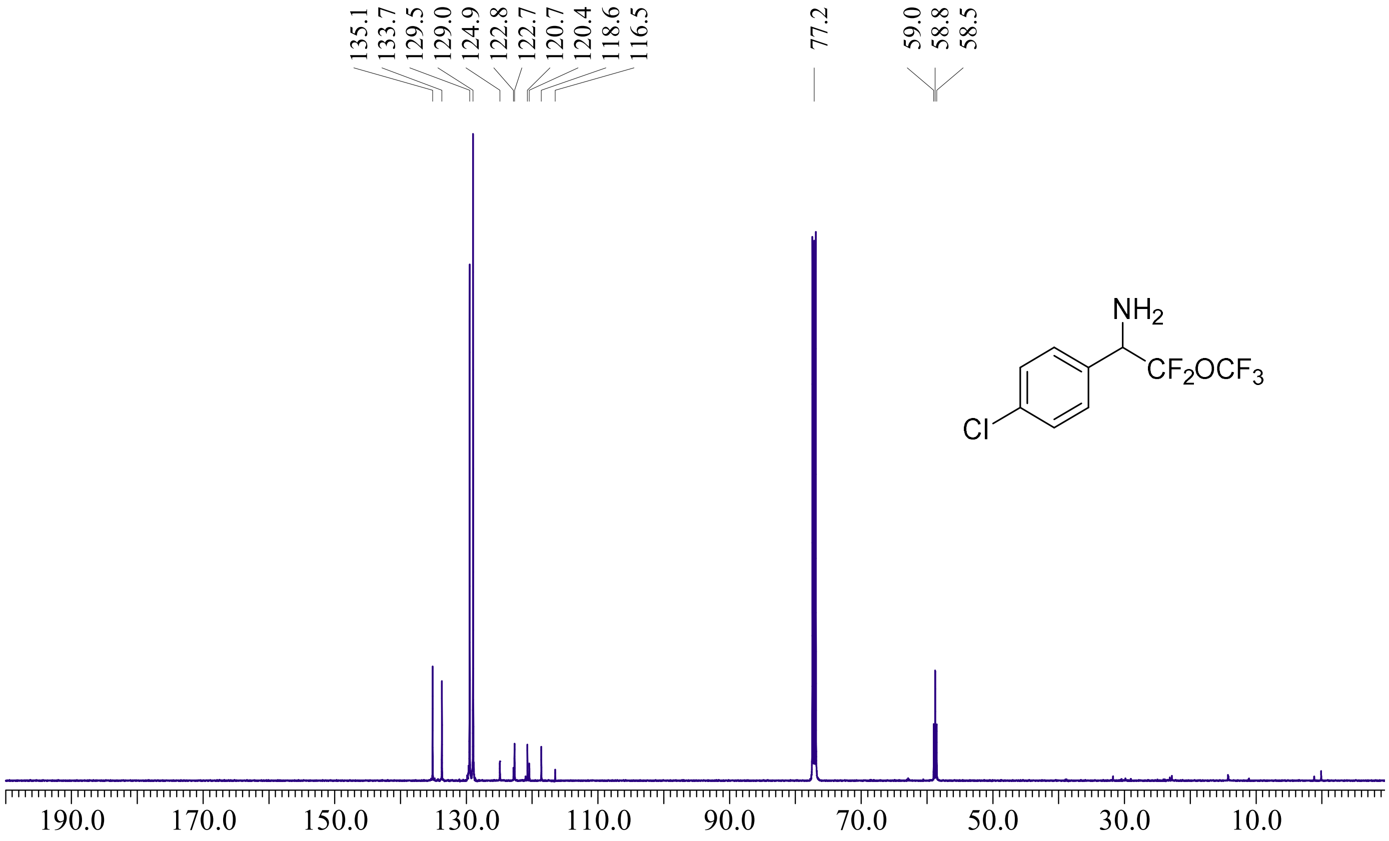

**$^{19}$F NMR** (282 MHz, CDCl3) : **8**

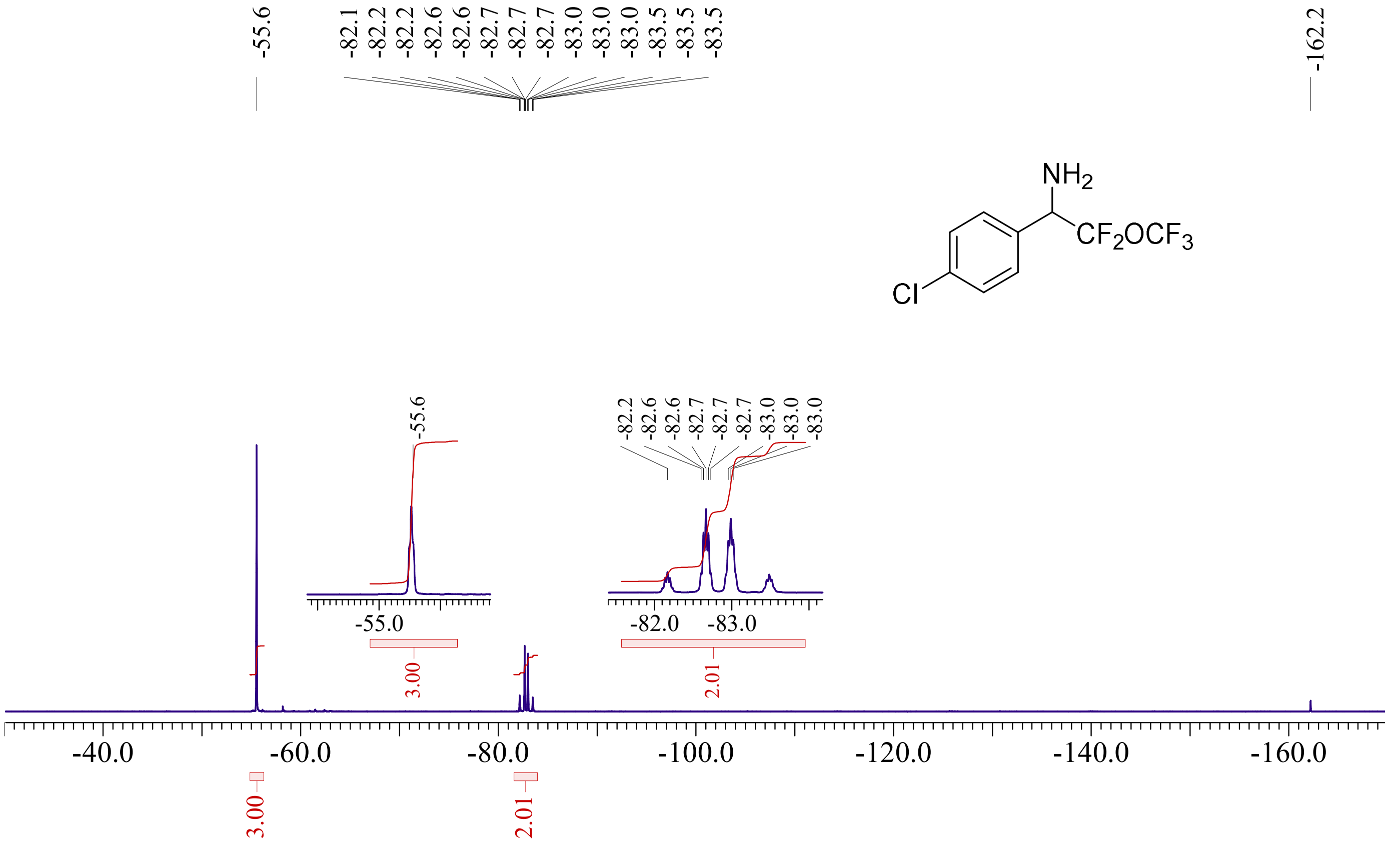


**$^{1}$H NMR** (500 MHz, CDCl3) : **10**

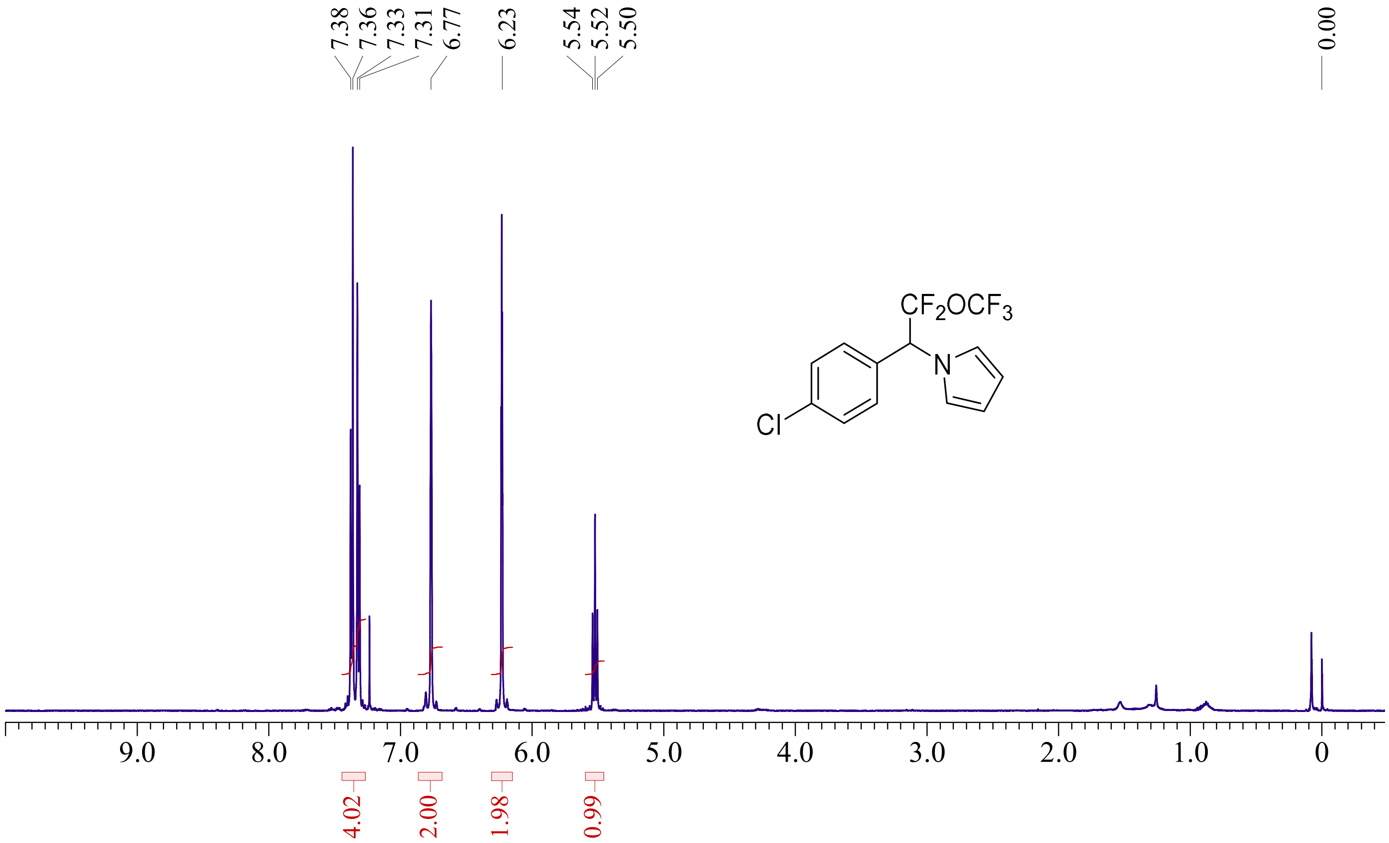

**$^{13}$C NMR** (126 MHz, CDCl$_3$) : **10**

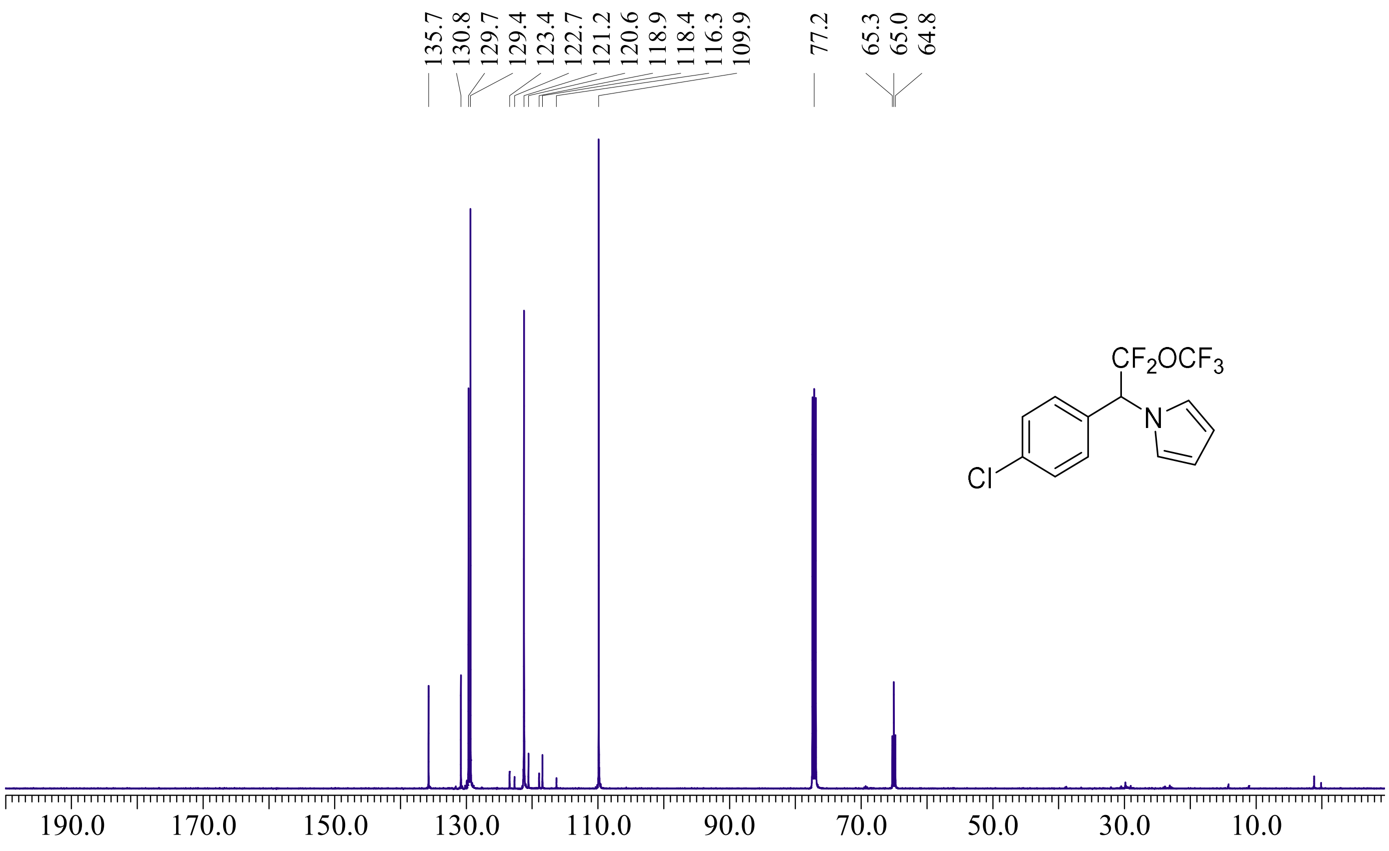


**$^{19}$F NMR** (282 MHz, CDCl$_3$) : **10**

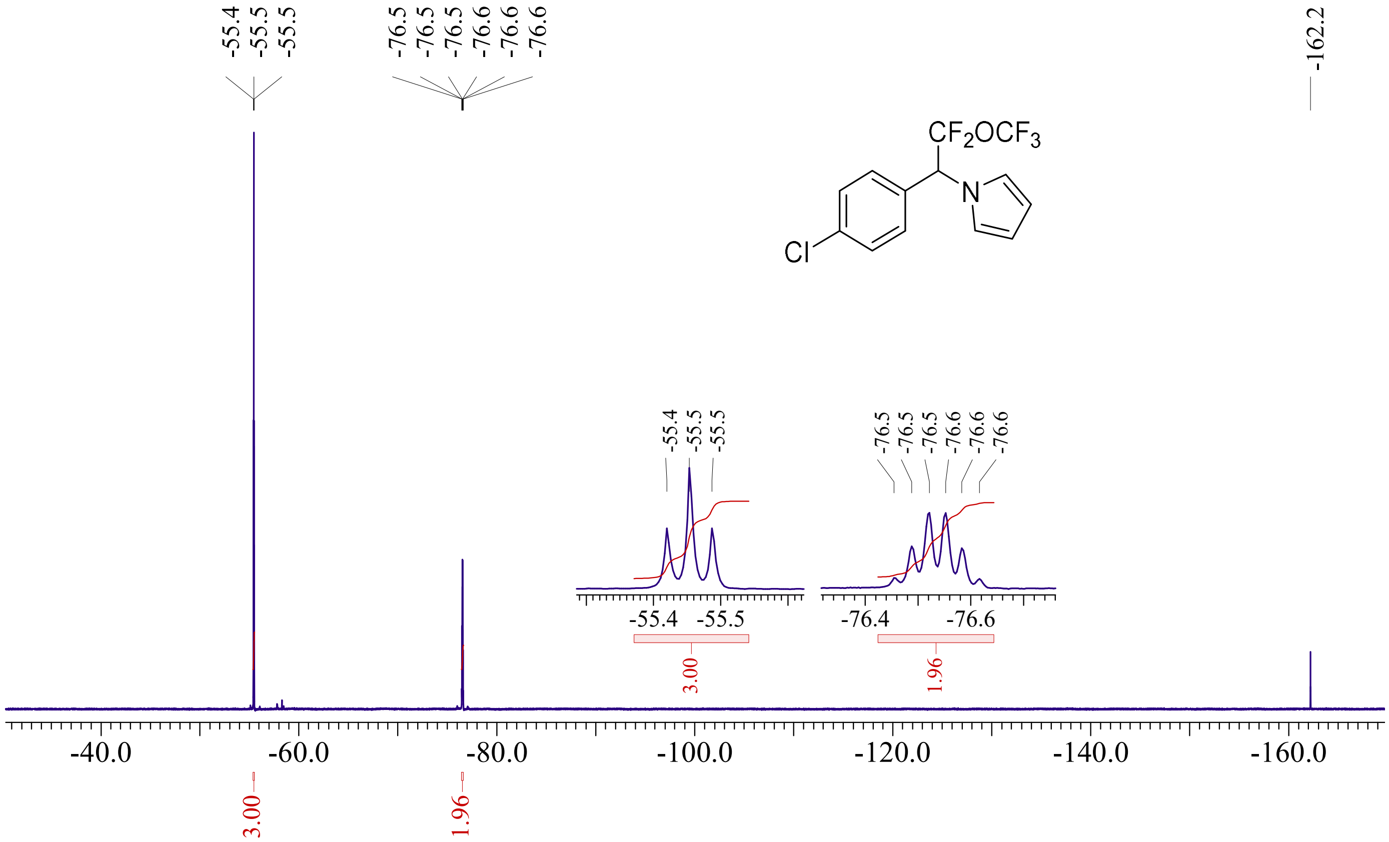

**$^{1}$H NMR** (500 MHz, $CDCl_3$) : **12**

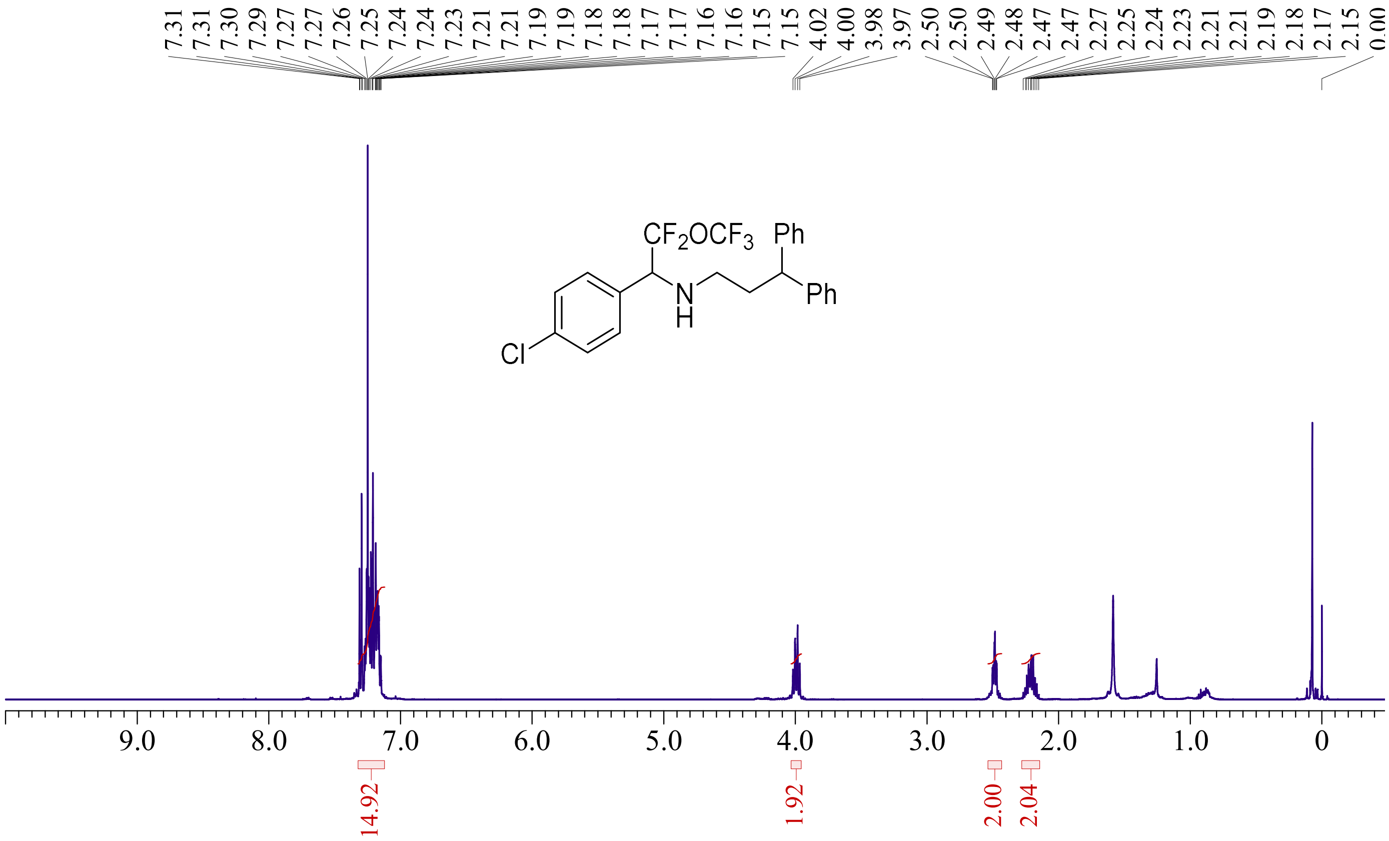


**$^{13}$C NMR** (126 MHz, $CDCl_3$) : **12**

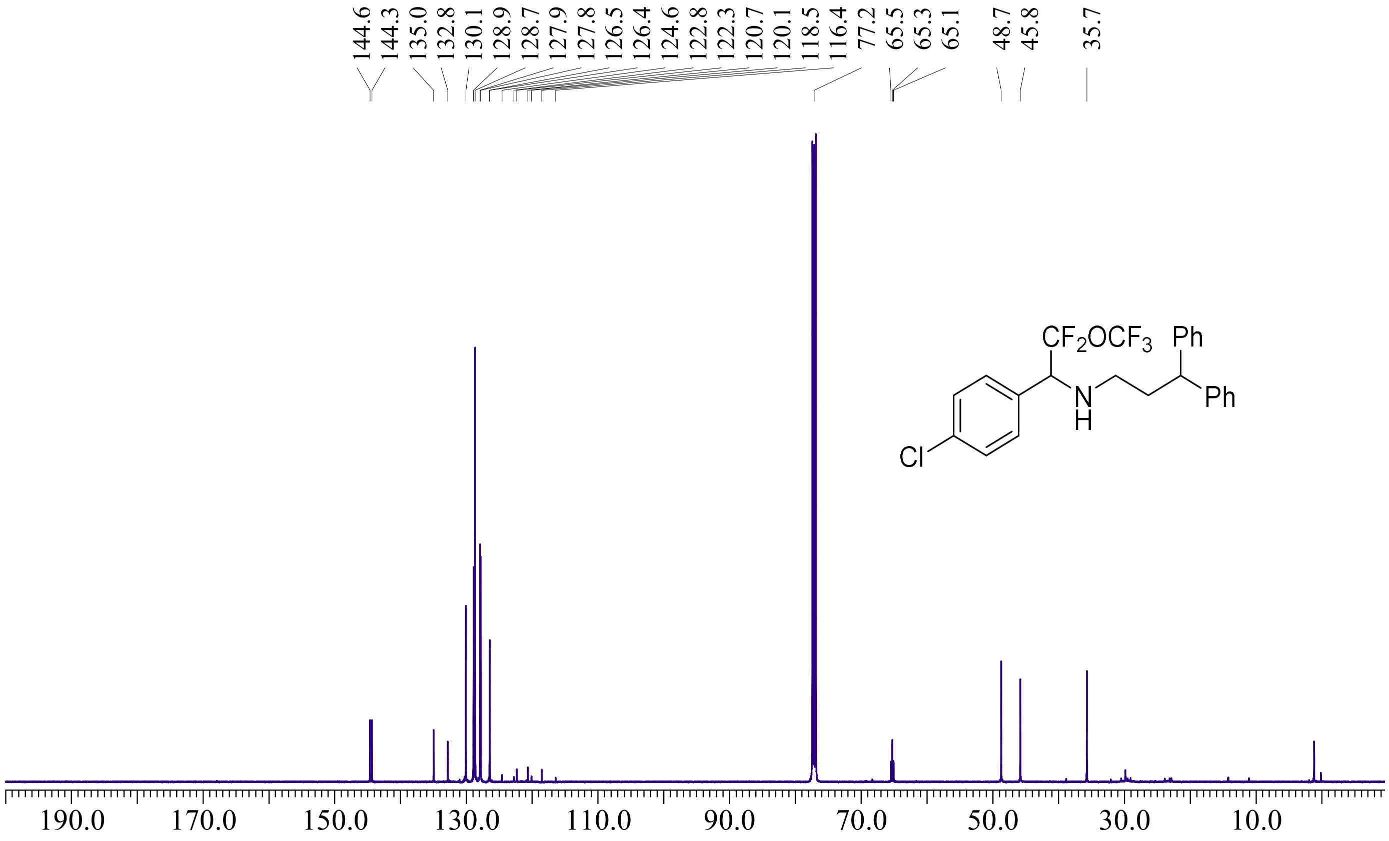

**$^{19}$F NMR** (282 MHz, $CDCl_3$) : **12**

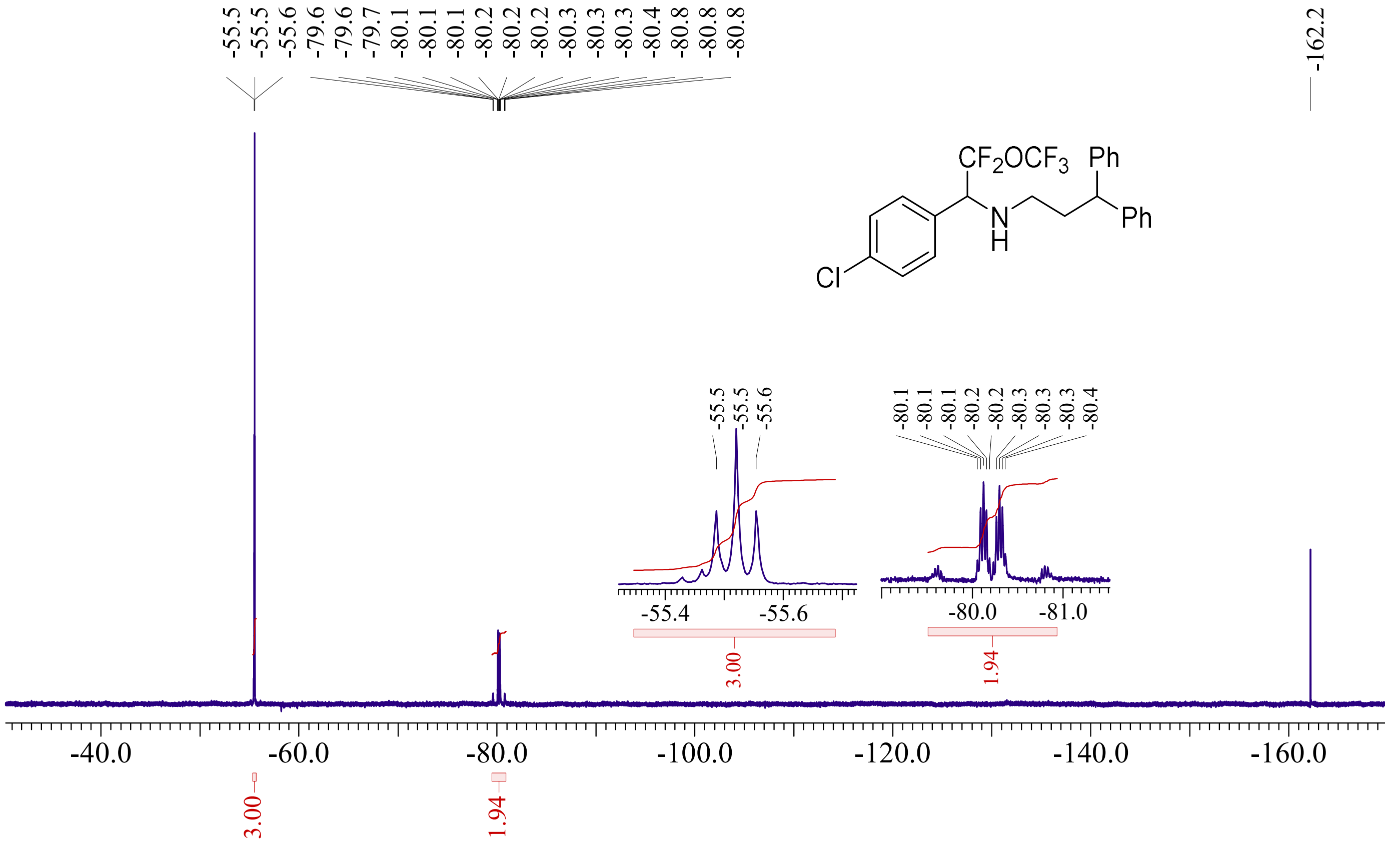


**$^{1}$H NMR** (500 MHz, $CDCl_3$) : **14**

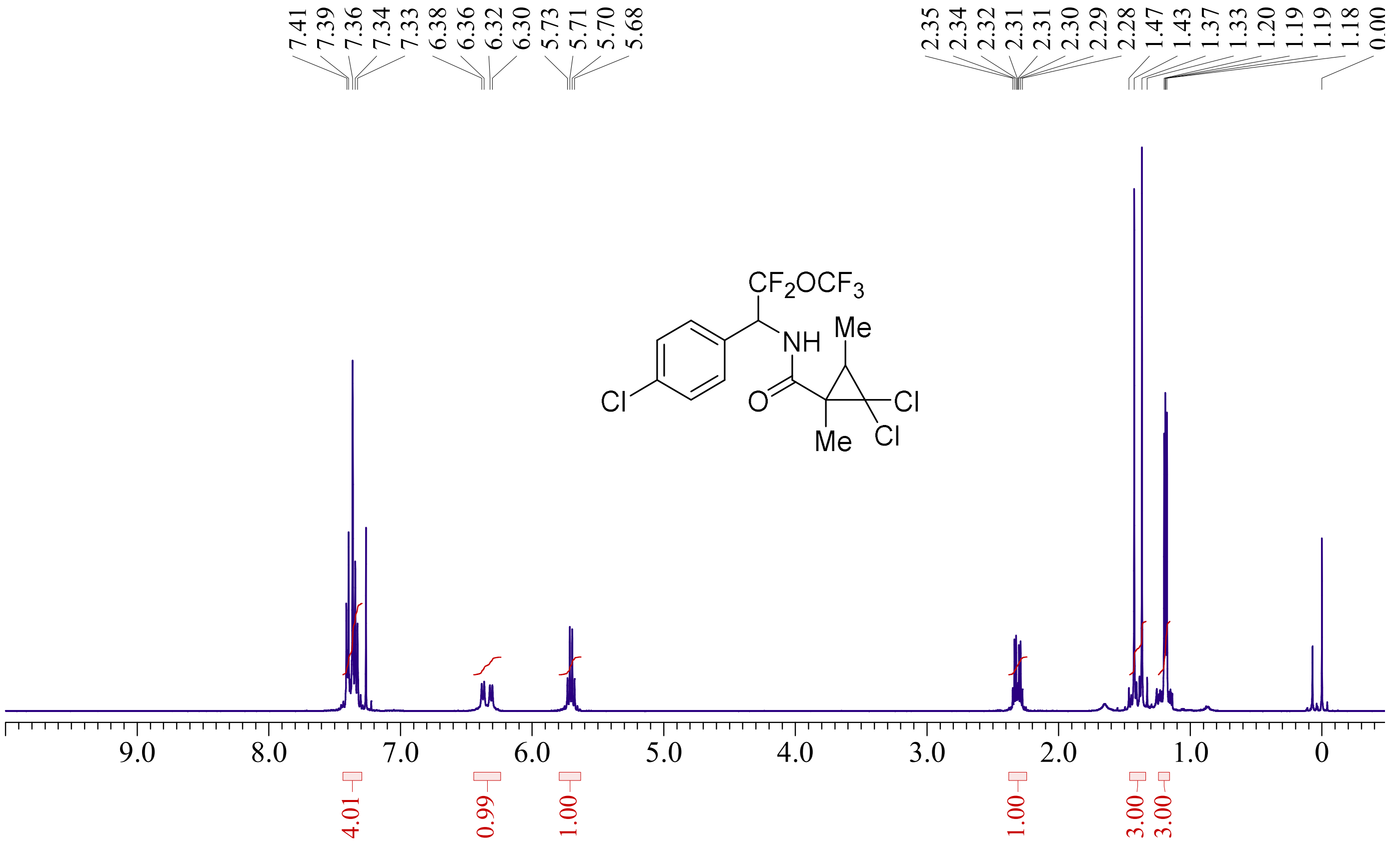

$^{13}$C NMR (126 MHz, $CDCl_3$) : **14**

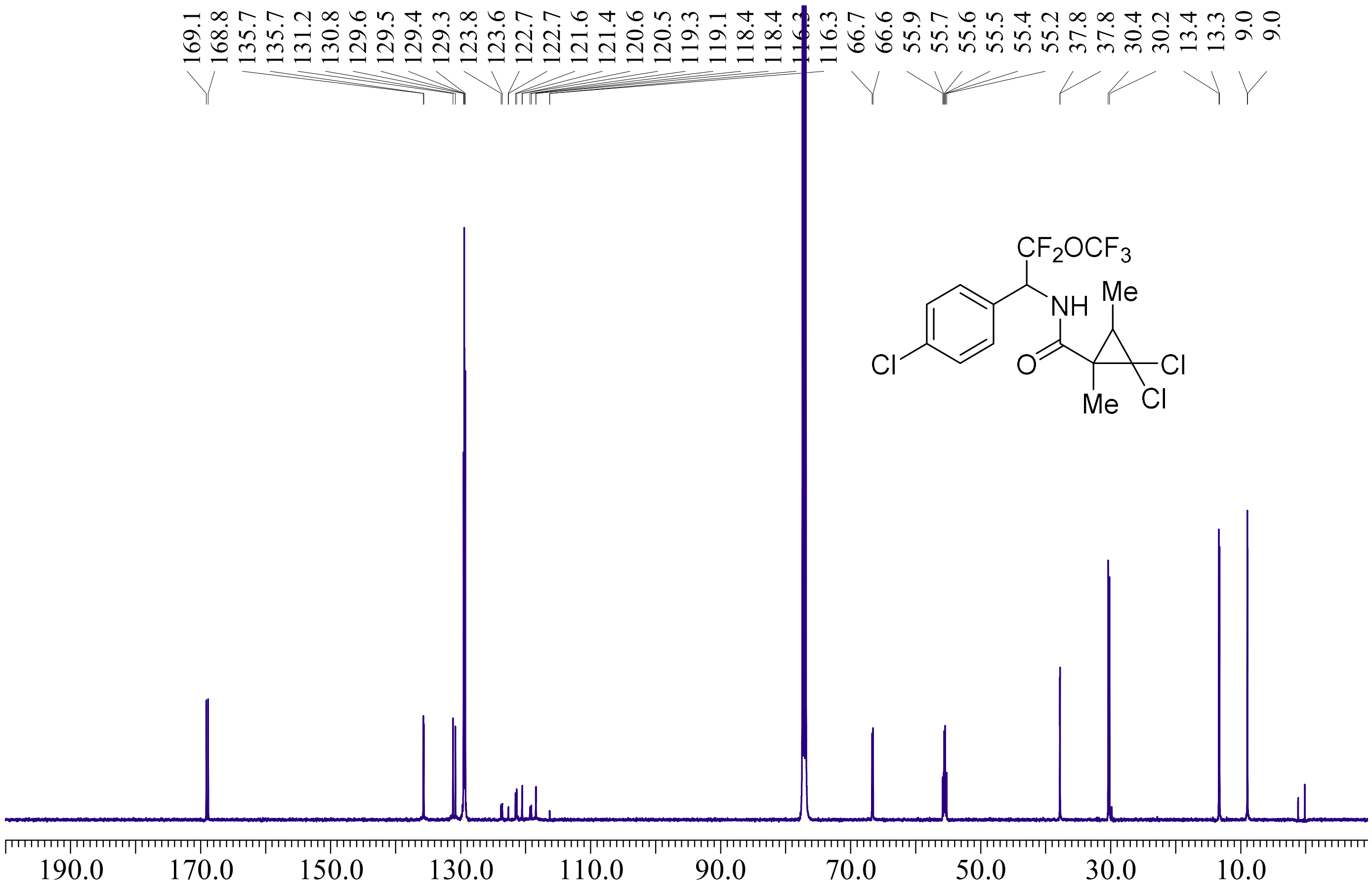


$^{19}$F NMR (282 MHz, $CDCl_3$) : **14**

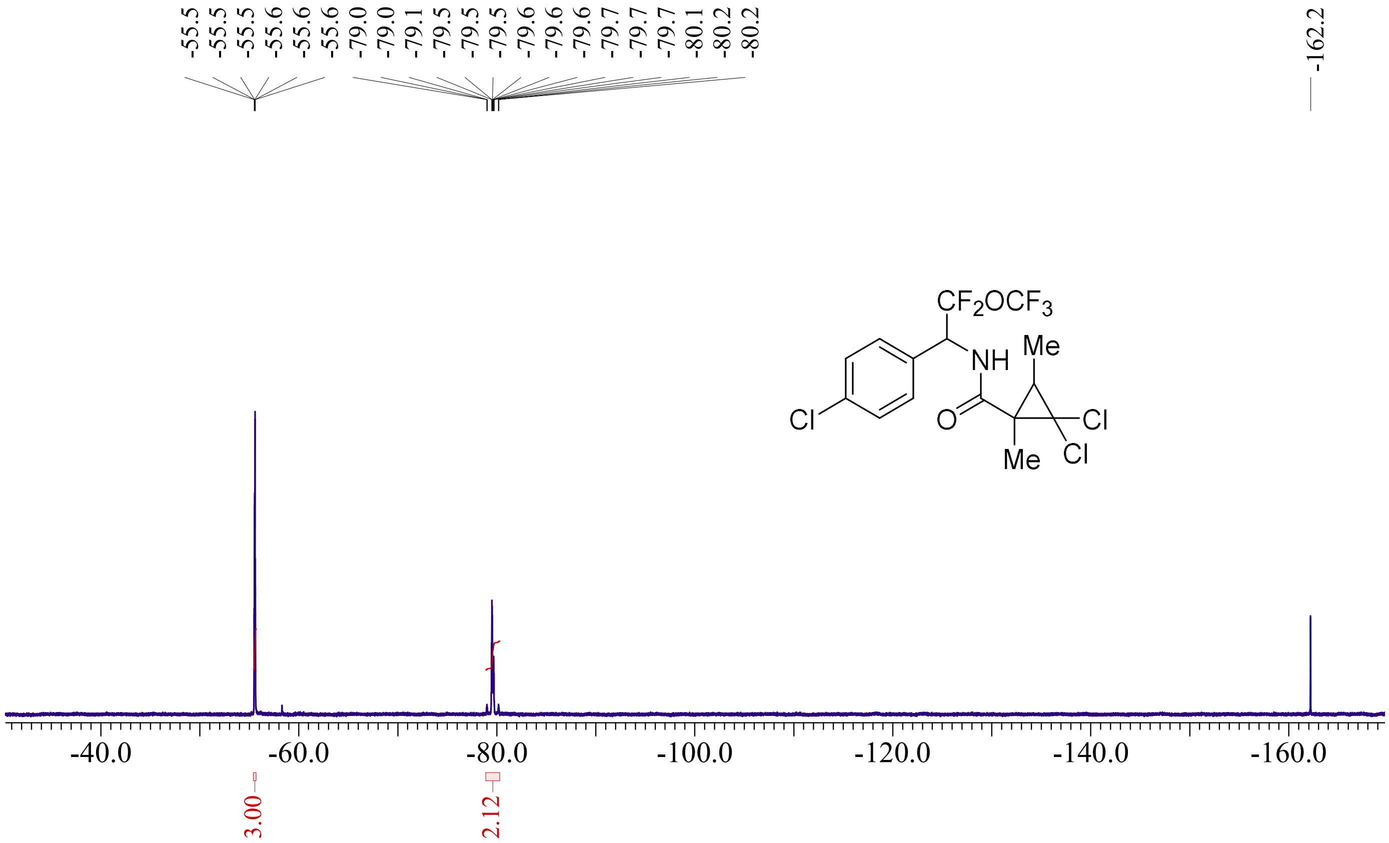

Supplement: SC-016-D4SC07788A-s001 [file SC-016-D4SC07788A-s001.pdf]
